# Combined Transcriptomics and Chemical-Genetics Reveal Molecular Mode of Action of Valproic acid, an Anticancer Molecule using Budding Yeast Model

**DOI:** 10.1038/srep35322

**Published:** 2016-10-13

**Authors:** Upendarrao Golla, Deepthi Joseph, Raghuvir Singh Tomar

**Affiliations:** 1 Department of Biological Sciences, Indian Institute of Science Education and Research (IISER), Bhopal, 462066, India

## Abstract

Valproic acid (VA) is a pharmacologically important histone deacetylase inhibitor that recently garnered attention as an anticancer agent. Since the molecular mechanisms behind the multiple effects of VA are unclear, this study was aimed to unravel the comprehensive cellular processes affected by VA and its molecular targets *in vivo* using budding yeast as a model organism. Interestingly, genome-wide transcriptome analysis of cells treated with VA showed differential regulation of 30% of the genome. Functional enrichment analysis of VA transcriptome evidenced alteration of various cellular processes including cell cycle, cell wall biogenesis, DNA repair, ion homeostasis, metabolism, stress response, transport and ribosomal biogenesis, etc. Moreover, our genetic screening analysis revealed VA molecular targets belonging to oxidative and osmotic stress, DNA repair, cell wall integrity, and iron homeostasis. Further, our results demonstrated the activation of mitogen-activated protein kinases (MAPKs) Hog1 (*p38*) and Slt2 (*p44/42*) upon VA treatment. Our results also exhibited that VA acts through alteration of mitochondrial, ER architecture and functions. Especially, VA effects were neutralized in cells lacking lipid particles. Altogether, our results deciphered the novel molecular insights and mechanistic links to strengthen our knowledge on diverse cellular effects of VA along with its probable therapeutic targets and detoxification approaches.

Eukaryotic cells have their genome in the tightly condensed form of chromatin with the help of basic core histone proteins H3, H4, H2A, and H2B, which were prone to undergo different post-translational modifications (PTMs) such as acetylation, methylation, etc^1^. DNA methylation by DNMTs (DNA Methyltransferases), addition or removal of histone PTMs, and remodeling of chromatin were referred as epigenetic modifications that bring the dynamics in chromatin structure without altering their DNA sequence^2^. Particularly, these modifications provide the ability, mechanisms to respond and assimilate the environmental cues by altering the gene expression and thus functional output^3^. During the past decades, studies have focused on the development of epidrugs such as inhibitors of DNMTs, Histone acetyltransferases (HATs), histone deacetylases (HDACs), Histone methyltransferases (HMTs) for Histone demethylases (HDMs) for the treatment of chronic diseases including cancer. Among epidrugs, HDAC inhibitors (HDACi) are the most advanced, and many of them are in clinical trials for evaluation^4^. Broadly, HDACi are classified based on their structure into cyclic peptides, hydroxamic acids, benzamides, and short-chain fatty acids^5^.

Valproic acid (VA; 2-propylpentanoic acid) is a branched short-chain fatty acid derived from valeric acid (naturally produced by valerian, *Valeriana officinali*s). VA is FDA approved drug, a mood stabilizer that primarily used in the treatment of bipolar disorder and later found effective against epilepsy and several types of seizures, migraine headaches, schizophrenia, clinical depression, anxiety and other neurologic/neurodegenerative disorders. The commercial use of VA in epilepsy and psycho-neurologic disorders credited to its inhibitory effects on Na^+^, Ca^2+^, and voltage-gated K^+^ channels, and VA attenuates the neurotransmitter Gamma Amino Butyrate (GABA) by increasing synthesis, inhibiting its degradation and turnover^6^. Moreover, antimicrobial properties of VA and susceptible interaction with other antibiotics were reported earlier^7^. In addition to its non-oncological clinical uses, recent studies have explored VA as potential anticancer epidrug in alone or combination therapy due to its inhibitory effects on HDACs^8^. Among four classes of HDACs, VA selectively inhibits the catalytic activity of Class I and II HDACs similar to that of other small-chain fatty acids, which are relatively weak HDACi^9^. Till date, there are 723 drugs known to interact with VA and modulate its effects (http://www.drugs.com/).

Despite the diverse therapeutic implications, VA treatment adversely affects embryonic development and also results in teratogenicity, depression, pancreatitis, autism, hyperammonemia, hepatic and hematopoietic damage^6^. Although the HDACs^9^ and glycogen synthase kinase-3 (GSK3)^10^ are the known targets of VA, the molecular mechanisms and genetic targets responsible for multiple effects of VA and its toxicity are unclear. So, the present study aimed to identify the underlying mechanisms of VA induced effects *in vivo* through chemical genetics and global transcriptomics approach using budding yeast *Saccharomyces cerevisiae*, which offers a robust model organism to unravel the conserved molecular targets and mode of action of bioactive molecules, toxicants and pollutants^11^. Interestingly, our transcriptome and genetic screening results revealed diverse cellular processes targeted by VA in addition to new molecular targets that belonging to cell wall biogenesis, metal homeostasis, oxidative stress and DNA repair are essential for mediating its effects and tolerance. Our results also showed that VA activates conserved MAP kinase signaling pathways in yeast. Altogether, our results facilitate us to understand the response of eukaryotic cells to VA *in vivo* and identify plausible diverse molecular mechanisms, targets and its mode of action.

## Results

### Valproic acid exhibits dose-dependent cytotoxicity in budding yeast

**Figure 1:**
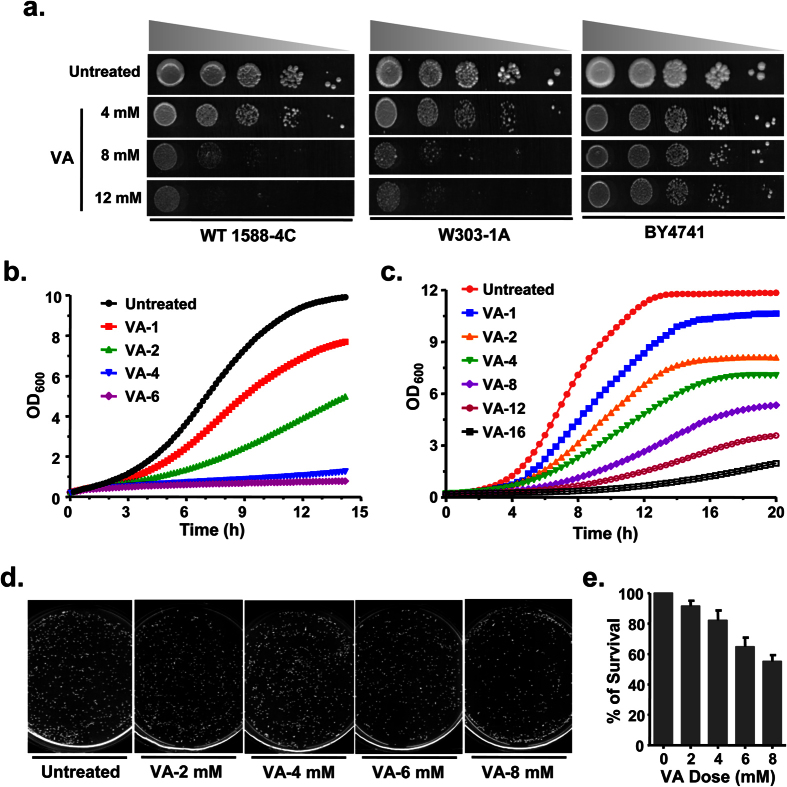
Valproic acid exhibit dose-dependent cytotoxicity in budding yeast. (**a**) Sensitivity to VA varies with the genotype of yeast cells. Ten-fold serially diluted yeast cultures of different wild-type strains (W1588-4C, W303-1A, BY4741) were spotted onto the SC-Agar plates supplemented without or with VA (4, 8 and 12 mM) and imaged after 72 h. (**b**,**c**) Real-time growth analysis of wild-type W1588-4C (**b**) and BY4741 (**c**) cells that were left untreated or treated with indicated doses of VA in SC-liquid media. The growth of yeast cells was monitored for 14 h (**b**) and 20 h (**c**) by recording Optical Density at 600 nm (OD_600_) with a regular interval of 15 min (**b**) and 20 min (**c**) each using plate reader. (**d**,**e**) VA treatment suppressed colony-forming ability of the yeast cells in a dose-dependent fashion. Equal number cells from both untreated and VA treated were spread onto YPD-Agar plates and imaged after 72 h (**d**). The number of colonies in untreated and VA treated plates were quantified and represented as % survival compared to untreated control (**e**).

To identify the optimal dose of VA and pursue further, we performed a growth assay with different commonly used laboratory Wild-type (WT) yeast cells. Interestingly, VA exhibited dose-dependent growth inhibition, but the effective dose varied with different wild-type yeast cells (Fig. 1a). Similarly, our real-time analysis of VA effect in liquid media also showed dose-dependent growth inhibition of 1588-4C (Fig. 1b) and BY4741 (Fig. 1c) yeast cells relatively at lower doses than the solid media. Hence, the dose of VA used in further experiments is entirely dependent on the respective wild-type cells and growth media. To reason the effect of VA on the cell viability, we stained VA treated wild-type (1588-4C) cells with methylene blue (MB), a vital dye that discriminates the metabolically active (unstained) and inactive cells (dark blue stained). As usual, untreated cells (metabolically active) were failed to stain with MB, whereas heat killed cells (metabolically inactive) were stained to dark blue (Fig. S1a). Surprisingly, cells treated with VA for 3 h failed to show MB stained cells, are metabolically active at the highest dose tested (Fig. S1a) and suggests that the growth inhibition by VA might be credited to cell arrest and not cell death. Accordingly, both the untreated and VA treated (for 6 h) wild-type cells grow at the similar rate after withdrawal of VA from growth media (Fig. S1b). Although VA treated cells didn’t stain with MB, our clonogenic assay results showed that the colony forming ability of cells decreased in a dose-dependent manner (Fig. 1d,e). Altogether, our results showed that VA treatment leads to dose-dependent growth inhibition of budding yeast.

### Functional enrichment analysis of valproic acid global transcriptome

**Figure 2:**
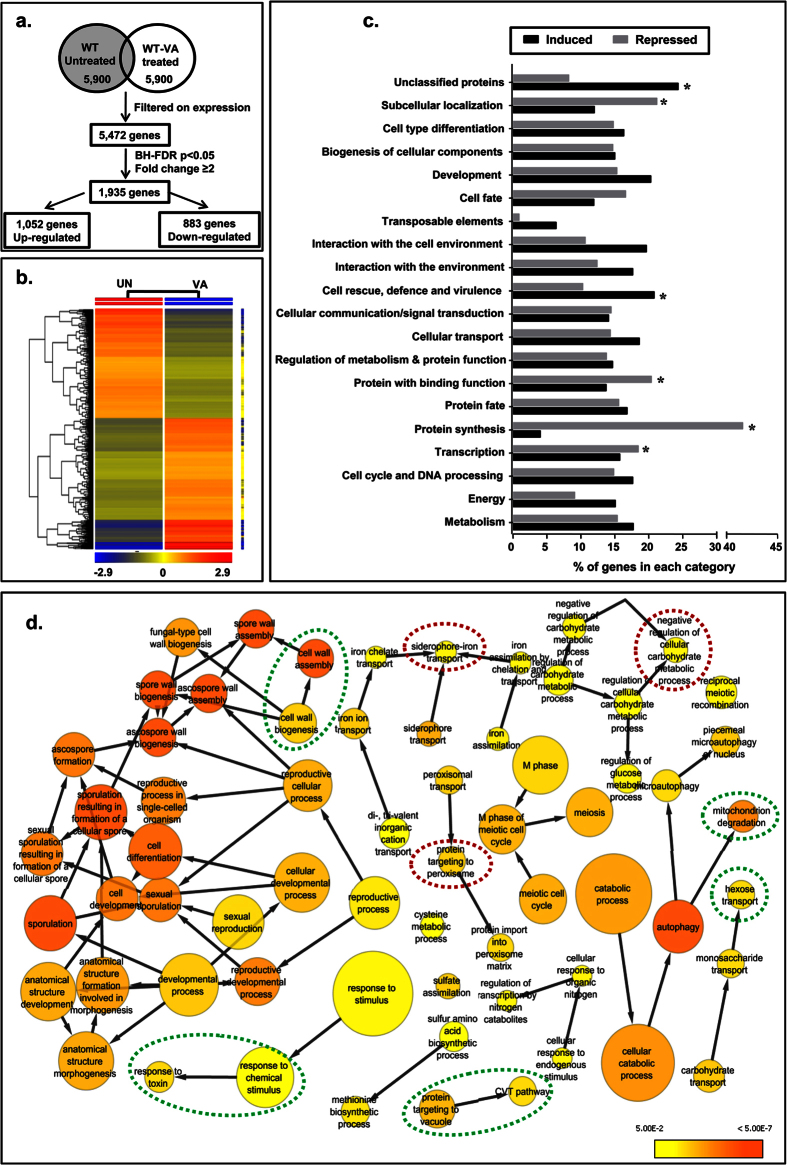
Functional classification and enrichment analysis of the global transcriptome of Valproic acid. (**a**) Experimental design for microarray analysis. A large number of genes were differentially expressed (filtered on expression) upon VA treatment compared to that of untreated cells. BH-FDR stands for Benjamini–Hochberg false discovery rate and *p* < 0.05 was considered statistically significant. (**b**) A hierarchically clustered heatmap is generated using the averaged relative expression (log_2_) values from two independent biological repeats of both untreated (UN) and VA treated, on the scale of red (induced)-blue (repressed). Each column in heatmap represents the normalized average values of untreated (UN) and VA transcriptome, and each row represents the genes (entities). (**c**) Functional classification of VA transcriptome (*p* < 0.05, Fold change ≥2) into the MIPS (Munich International Center for Protein Sequences) categories. The enrichment of each category was represented as % of genes altered upon VA treatment compared to the whole number of genes present in each category. ‘*’ indicates the significantly enriched functional categories. (**d**) Analysis of VA induced (≥2 fold) transcriptome for overrepresented (enriched) biological processes using a Biological Networks Gene Ontology (BiNGO) tool reveal a biological network of significantly enriched Gene Ontology (GO) categories [Hypergeometric test (*p* < 0.05) and corrected for Benjamini-Hochberg False Discovery Rate (FDR)]. Each node in the network represents a term of biological process (cluster of genes) whereas the edges represent the interaction between different nodes (biological processes). The nodes in yellow color show the significant enrichment of term. As the significance (*p*-value) of enrichment increases, the color of node goes from yellow to orange. Node size in the network is relative to the number of nodes with that GO-term in the query set.

Transcriptome profiling offers a platform to understand the global alterations in the mRNA expression upon any stimuli^12^. So in this study, we used microarray analysis to understand the global cellular effects of VA. Based on our clonogenicity and dose-response analysis, 6 mM of VA was found to be effective enough to induce non-lethal growth inhibition and would merely indicate that VA cause cellular stress. Notably, WT (1588-4C) cells treated with a sublethal dose of VA (6 mM) showed 1,935 differentially expressed genes (DEG’s), of which 1,052 genes were induced, and 883 genes were repressed (Fig. 2a; Supplementary Table S3). To confirm the VA transcriptional regulation, we have validated a set of genes by RT-qPCR, and the results were in consistent with that of expression pattern obtained by microarray analysis (Fig. S2). Additionally, our cluster analysis using hierarchical clustering algorithm identified co-expressed DEG’s sets across the different samples (Fig. 2b). Interestingly, promoter analysis of VA induced transcriptome (repressed in untreated cells) against ChromatinDB Chip-chip database indicated that the occupancy of histones was higher, and the permissive PTM marks were depleted at the promoters of induced genes (Fig. S3). To reveal the functional significance of VA transcriptome, we performed functional clustering of DEGs according to MIPS classification and found that the transcripts belonging to protein synthesis, transcription, and sub-cellular localization were significantly repressed whereas that of cell rescue and defense were induced (Fig. 2c). The enrichment analysis of induced transcriptome suggests that VA affects mainly functional categories including transport, transcription, metabolism, DNA repair, and stress response pathways (Table S4). In contrast, enrichment analysis of repressed transcriptome suggests that VA affects protein synthesis and folding, translation, mitotic cell cycle and cell morphogenesis (Table S5).

To gain further insights, Gene Ontology (GO) enrichment analysis was performed with induced transcriptome of VA. We found major GO biological processes including cell wall biogenesis, response to stress, iron homeostasis, transport, mitochondrial biogenesis, metabolism, meiosis, and protein targeting was significantly (*p* ≤ 0.05) overrepresented (Fig. 2d). Additionally, the overrepresented functional categories were identified in VA transcriptome using FungiFun2 application and were also in line with that of analysis using aforementioned tools (Fig. S4). Further, GO enrichment analysis using GOEAST tool led us to gain more insights on different biological processes that were enriched and clustered together functionally in VA transcriptome (Fig. S5). As VA treatment affects many of biological processes, then we reasoned the regulatory associations of VA transcriptome using YEASTRACT tool and identified 73 and 26 transcriptional factors (TFs) present in VA induced and repressed genes respectively along with % of VA transcriptome regulation by each TF (Table S6). The regulatory associations between different TFs and their major target genes were also left us a clue about the possible mode of action of VA (Fig. S6). Taken together, our functional enrichment analysis of transcriptome illustrated that VA treatment leads to cellular injuries and evoke an abundant transcriptional response that belongs to a series of diverse biological processes.

### Valproic acid alters iron homeostasis and requires functional mitochondria for its tolerance

**Figure 3:**
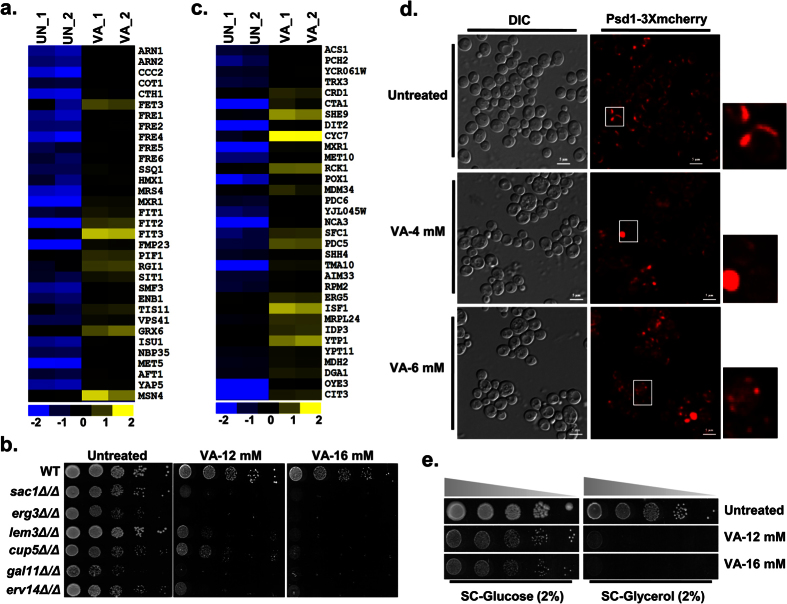
Valproic acid regulates iron homeostasis genes and alters mitochondrial organization. (**a**,**c**) VA exposure leads to upregulation of iron regulon genes involved in iron transport, iron-sulfur cluster assembly and homeostasis (**a**) and mitochondrial organization, biogenesis and functions (**c**). The relative expression values (log_2_) for each gene was illustrated by blue-yellow color scale (yellow color indicates up-regulation whereas blue color for down-regulation) in the heatmap. (**b**) Loss of iron homeostasis genes in yeast exacerbates VA sensitivity. Ten-fold serially diluted Wild-type (BY4743) and iron homeostasis null mutants were spotted onto SC-Agar plates supplemented without or with VA and imaged after 72 h. (**d**) VA treatment alters mitochondrial organization as indicated by Psd1-3Xmcherry (Mitochondria resident) localization. Wild-type cells expressing Psd1-3Xmcherry were left untreated (Control) or treated with VA (6 mM) for 3 h and visualized under Apotome microscope using ‘mCherry’ filter. Scale bar represents 5 μm. Images are representative of at least two independent experiments for each condition. (**e**) VA exposure leads to mitochondrial damage, and thus cells can’t be able to utilize non-fermentable carbon source (glycerol) as a growth media. The growth of cells on medium containing fermentable carbon source (glucose) don’t depend on mitochondrial respiration, and thus VA has less effect on growth compared to that of growth on non-fermentable carbon source (glycerol) containing medium. The growth of Wild-type (BY4741) cells on SC-Agar plates supplemented with Glucose (2%) or Glycerol (2%) as carbon sources was imaged after 72 h.

It is evident from our transcriptional profiling and enrichment analysis that the cells treated with VA showed alteration of iron homeostasis, transport, iron-sulfur (Fe/S) cluster (ISC) assembly genes (Fig. 3a). VA treatment also lead to the upregulation of iron regulatory TFs *AFT1* and *YAP5* (Table S6), which achieves iron homeostasis^13^. Additionally, VA transcriptome signatures showed the induction of genes involved in iron transport (*FIT1/FIT2/FIT3/ARN1/ARN/2/VPS41/SIT1/ENB1*), ferric-chelate reductase (*FRE1/2/4/5/6*), iron ion transmembrane transport (*COT1/FET3/MRS4/SMF3*), and genes involved in iron homeostasis (*CCC2/CTH1/FMP23/HMX1/MSN4/TIS11*) (Fig. 3a). Further, growth assay with iron homeostasis mutants was performed to identify VA genetic targets (Fig. S7). We found *CUP5, ERG3, LEM3, GAL11, ERV14* and *SAC1* null mutants sensitive to VA compared to wild-type (BY4743) cells (Fig. 3b). Under iron-sufficient conditions, iron ions accumulate in mitochondria and synthesize ISC by integrating with sulfur and redox pathways. Accordingly, VA transcriptome showed upregulation of genes involved in ISC binding and assembly (*GRX6/RLI1/NBP35/ISU1/TYW1*) and sulfur metabolism (*MET1/3/5/8/10/14/16/22*), which led us to propose that VA alters iron homeostasis and ISC assembly.

Both iron ions and ISC play very important role in the mitochondrial maintenance^14^. So, we looked into VA transcriptome and found that the genes involved in mitochondrial organization and biogenesis (*SHE9/MDM34/NCA3/RPM2*) were upregulated. Moreover, the genes related to mitochondrial functions such as TCA cycle (*YJL045W/YLR164W/IDP3/MDH2/CIT3*), respiration (*CYC7/AIM33/YTP1*), metabolic process (*ACS1/MET10/POX1/PDC5/MDH2/OYE3*), electron transport, and energy conservation (*TRX3/CYC7/YJL045W/OYE3/ACS1/IDP3*) were induced significantly (Fig. 3c). Then we motivated to look into mitochondrial organization upon VA treatment by using cells harboring Psd1-3Xmcherry. *PSD1* encodes a phosphatidylserine decarboxylase enzyme of the inner mitochondrial membrane that regulates mitochondrial morphology and fusion^15^. Interestingly, the localization of Psd1-3Xmcherry indicates the mitochondrial reorganization upon VA treatment compared to intact mitochondrial morphology and peripheral organization in untreated cells (Fig. 3d). Additionally, VA treated cells restored the normal mitochondrial architecture after withdrawal of VA from the growth media (Fig. S9a). Furthermore, the wild-type cells exhibited higher sensitivity to VA in non-fermentable carbon source (2% Glycerol) containing media, where yeast cells grow only if they have functional mitochondria for respiration compared to glucose-containing media, where cells grow by fermentation and don’t require functional mitochondria (Fig. 3e). Thus, our transcriptional signatures and results indicate that VA mediates its effects through modulation of iron ion homeostasis, ISC assembly, mitochondrial organization and functions.

### Valproic acid treatment induces oxidative stress response, DNA repair genes and degradation of Sml1

**Figure 4:**
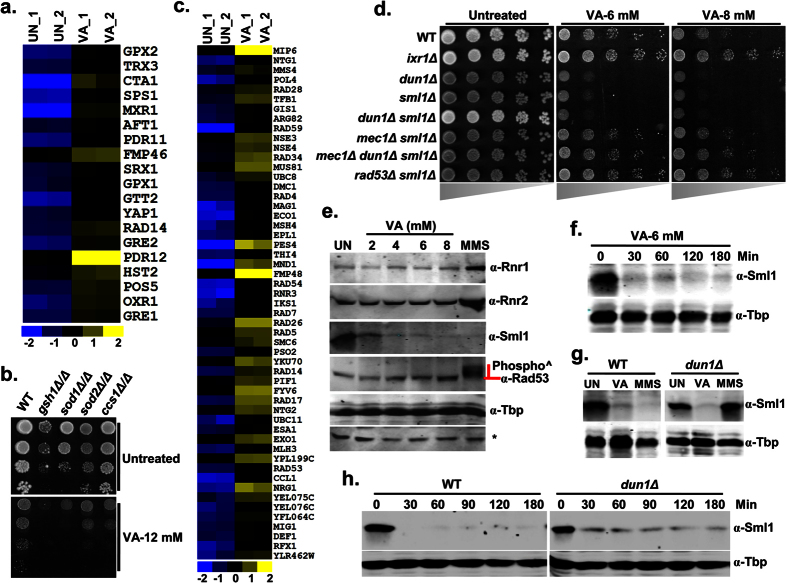
Transcriptional regulation of stress response and DNA damage repair genes by Valproic acid. (**a**,**c**) VA exposure leads to upregulation of oxidative stress response genes (**a**) and DNA damage repair genes (**c**). The relative expression values (log_2_) for each gene was illustrated by blue-yellow color scale (yellow color indicates up-regulation whereas blue color for down-regulation) in the heatmap. (**b**,**d**) Loss of genes involved in redox homeostasis and checkpoint kinase cascade increase VA susceptibility and become sensitive to VA. Ten-fold serially diluted cultures of sensitive antioxidant mutants (**b**) and Mec1-Rad53-Dun1-checkpoint pathway mutants (**d**) along with wild-type (W1588-4C) were spotted onto SC-Agar plates supplemented without or with indicated dose of VA and imaged after 72 h. (**e**–**h**) VA treatment leads to DNA replication stress. VA treated cells were failed to show the hallmarks of DNA damage such as induction of ribonucleotide reductases (Rnr1/2) and Rad53 phosphorylation but showed a drastic decrease in Sml1 levels without depending on Dun1-kinase. Exponentially growing Wild-type W1588-4C (**e**,**f**) alone or along with *dun1*Δ (**g**,**h**) cells were left untreated (UN) or treated with indicated dose of VA (**e**), if not VA (6mM) and MMS (0.03%). The cells were incubated at 30 °C and collected after 3 h (**e**,**g**) or at indicated time intervals (**f**,**h**) after the treatment. Whole-cell TCA protein extracts were analyzed by immunoblotting using indicated antibodies. ‘*’ Represents a non-specific band in the same blot of Rnr2 served as loading control along with Anti-Tbp. Cells treated with a standard DNA damaging agent (MMS) were served as a positive control.

Although VA known to induce the accumulation of reactive oxygen species (ROS) and apoptotic cell death in yeast^16^, transcriptional regulation and the target genes required for VA tolerance were unknown. Here, our VA transcriptome showed upregulation of a considerable number of oxidative stress response genes and suggests the possible alteration of redox homeostasis (Fig. 4a). To identify the target genes required for VA tolerance, we performed a growth assay with null mutants of redox homeostasis genes (Fig. S8). Interestingly, cells lacking cytosolic copper-zinc superoxide dismutase (Sod1), its copper chaperone (Ccs1) involved in oxidative stress protection, and γ-glutamylcysteine synthetase (Gsh1) that catalyzes the first step in glutathione biosynthesis were found sensitive to VA (Fig. 4b). Our results thus suggest the critical role of these antioxidant gene products in providing tolerance against VA induced oxidative stress.

Genomic DNA is the prime target of oxidative damage and thus results in DNA lesions^17^. Although VA induces ROS, the probable DNA lesions and the repair mechanisms were unclear. In our attempt to understand the VA cellular effects by transcription profiling has revealed enrichment (Fig. S5) and upregulation of DNA damage repair genes in VA treated cells (Fig. 4c). The majority of induced DNA repair genes by VA belongs to cell cycle checkpoint (*RAD4/PSO2/RAD14/RAD53/YKU70/RAD17*), non-homologous end joining-NHEJ (*POL4/YKU70/FYV6*), nucleotide-excision repair-NER (*TFB1/RAD34/RAD4/RAD26/RAD14*), base-excision repair-BER (*NTG1/MAG1/NTG2*), mismatch repair-MMR (*MSH4/EXO1/MLH3*), and single-strand annealing-SSA (*RAD59/RAD54*) processes, and thus supports our hypothesis of VA induced DNA damage. In response to DNA damage or DNA replication stress, Mec1/Rad53/Dun1 checkpoint kinase cascade activates several repair mechanisms^18^. To investigate the role of this Mec1/Rad53/Dun1-kinase cascade in providing VA tolerance, we screened the individual or combined deletion mutants of this checkpoint pathway for VA sensitivity. Notably, a null mutant of Dun1-kinase, ribonucleotide reductase inhibitor (*sml1∆*) and the combined *dun1∆sml1∆* mutant showed sensitivity to VA (Fig. 4d). Moreover, the sensitivity of *sml1∆* to VA was neutralized upon the loss of upstream Mec1 and Rad53-checkpoint kinases, thus suggests the role of Sml1 in providing VA tolerance. Additionally, the loss of *IXR1* that encodes an HMG (high mobility group box) domain containing protein resulted in VA resistance (Fig. 4d). These results suggest a prime role of Mec1/Rad53/Dun1-checkpoint cascade in VA tolerance. To check the activation of this kinase cascade by VA, we analyzed hallmarks such as activation of Rad53, induction of ribonucleotide reductases (Rnr1/2/4) and degradation of Sml1 by immunoblotting^19^. Our results showed that VA treatment didn’t induce any of the Rnr’s expression and show detectable Rad53 phosphorylation (Fig. 4e). However, the levels of Sml1 were drastically decreased upon VA treatment and hence indicate the probable induction of DNA replication stress by VA (Fig. 4e,f).

Previous studies have demonstrated that the activation of Dun1 by Mec1 and Rad53-kinases leads to Sml1 protein degradation in S-phase and response to genotoxic stress^19^. To ascertain whether Dun1 participate in VA-mediated Sml1 regulation, we examined Sml1 levels in *dun1*Δ cells after VA treatment. Although untreated and MMS-treated *dun1*Δ cells retain intact Sml1, VA promoted Sml1 degradation in *dun1*Δ cells similar to that of wild-type cells (Fig. 4g) and follows the same kinetics (Fig. 4h). Sml1 levels are diminished upon iron depletion in Dun1 dependent fashion, independent of Mec1 and Rad53-kinases^20^. Thus, Dun1 independent regulation of Sml1 by VA suggests the probable role of Mec1/Rad53-kinases and indicates that the degradation is activated by possible DNA damage/replication stress, and not iron depletion. Altogether, our results demonstrated that VA treatment alters redox homeostasis and leads to genomic instability.

### Valproic acid induces lipid homeostasis, unfolded protein response (UPR) genes and alters endoplasmic reticulum (ER) architecture

**Figure 5:**
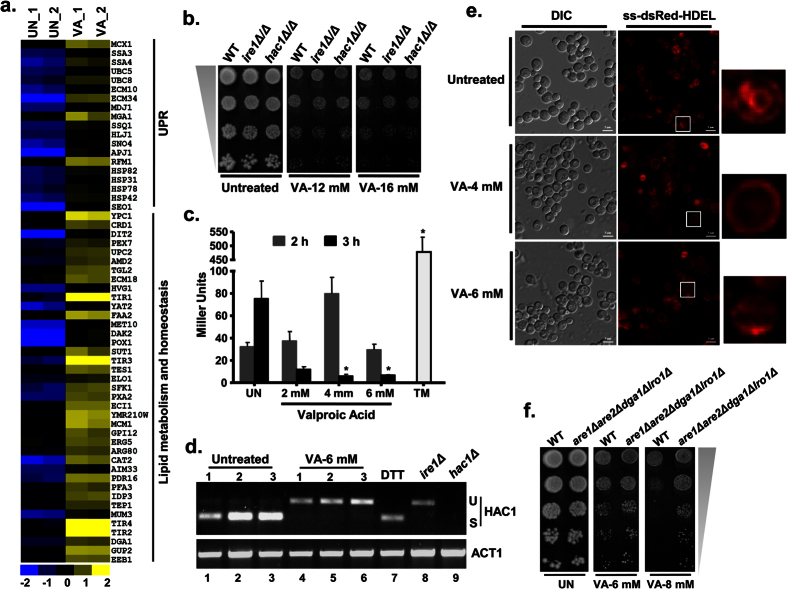
Valproic acid alters Unfolded Protein Response (UPR) and ER organization. (**a**) VA exposure leads to upregulation of UPR and lipid homeostasis genes. (**b**) ER stress regulators Ire1 and Hac1 didn’t have any role in VA tolerance. The growth of wild-type (BY4743), *hac1∆* and *ire1∆* mutants on SC-Agar plates that were supplemented without or with VA was imaged after 72 h. (**c**) VA treated cells were failed to show an increased UPR stress, instead showed a decrease in comparison to the basal levels in untreated cells. The β-galactosidase activity was measured in exponentially growing wild-type (BY4741) cells carrying a 2 μ *UPRE-lacZ* reporter plasmid (*pPW344*), both in absence and presence of VA and represented in terms of Miller Units. Cells treated with tunicamycin-TM (1 μg/ml) for 1 h were used as a positive control. The results were shown as mean ± standard error of mean (n = 2) and *p < 0.05 were considered as significant compared to untreated control (Student’s *t*-test). (**d**) VA treatment leads to accumulation of inactive *HAC1* precursor mRNA and indicates the negative effects of VA on ER stress. Total RNA’s were extracted from exponentially growing cells that were left untreated (control) or treated with VA (6 mM) in triplicate for 3 h and reverse transcribed to cDNA. *HAC1* splicing was tested by PCR using indicated *HAC1* splicing-specific primer (U-unspliced; S-spliced form of *HAC1*). Cells treated with 2mM Dithiothreitol (DTT), an ER stress inducer was served as a positive control whereas *ire1∆* cells were served as a negative control for *HAC1* splicing. *hac1∆* cells were used to check the specificity of the splicing primers used. *ACT1* specific primers served as control. (**e**) VA treatment of wild-type (*GSHY583*) cells leads to perturbation of ER architecture as indicated by altered localization of ss-dsRed-HDEL (ER marker) reporter. Scale bar represents 5μm. Images are representative of at least two independent experiments for each condition. (**f**) A quadruple mutant that is inositol auxotrophic and lacks lipid particles exhibit VA resistance and hints an approach for neutralizing VA toxicity. The growth of wild-type (WT) and quadruple mutant on SC-Agar plates supplemented without or with VA (6 and 8 mM) was imaged after 72 h.

Previously, VA is known to deplete cellular levels of inositol, a precursor of phospholipid biosynthesis and a key regulator of many cellular processes including lipid homeostasis and signal transduction^21^. Consequently, our VA transcriptome also showed enrichment of fatty acid metabolism and lipid/fatty acid transport processes (Fig. S4a). Also, the induction of lipid homeostasis genes belonging to lipid biosynthesis (*CRD1/ELO1/SFK1/ERG5/HES1/DGA1*), lipid/fatty acid metabolism (*FRM2/FAA2/YAT2/POX1/POT1/ECI1/CAT2/YOR059C*), transport (*SEO1/PEX7/YAT2/HVG1/BST1/MET10/PXA2/CAT2/PDR16/CRC1/GUP2*), and unfolded protein response (UPR) stress genes was observed (Fig. 5a). To ascertain the role of Ire1 and Hac1 which primarily involved in the activation of UPR stress response genes in budding yeast^22^, we performed a growth assay with their null mutants and found none of them are required for VA tolerance (Fig. 5b). Next, we measured the activation of UPR upon VA treatment in wild-type (BY4741) cells expressing a *UPRE::lacZ* reporter^23^. As expected, cells treated with a standard ER stress inducer tunicamycin showed 20-fold induction of β-galactosidase activity. In contrast, VA treated cells didn’t show a significant increase in β-galactosidase activity, instead showed a reduction of basal level activity (Fig. 5c). To confirm further, we analyzed *HAC1* splicing that is promoted by Ire1under ER/UPR stress^22^. Cells lacking the splicing factor Ire1 showed inactive *HAC1* precursor mRNA (*HAC1*^*u*^, for unspliced) whereas cells treated with a known ER stress inducer (DTT) showed active mature form (*HAC1*^*s*^, for spliced) of the transcription factor and served as negative and positive controls respectively. Surprisingly, our results showed that VA treated cells exhibited the accumulation of inactive (unspliced) *HAC1* form compared to untreated cells that showed basal level active form (spliced) of *HAC1*, suggests possible negative effects of VA on ER functions (Fig. 5d).

To monitor the VA effects on overall ER architecture, we visualized wild-type cells harbouring ss-dsRed-HDEL reporter (a marker for nuclear and cortical ER) after VA treatment^24^. As usual, dsRed-HDEL reporter was able to localize properly in peripheral ER near the plasma membrane and nuclear ER envelope in untreated cells. In contrast, cells treated with VA showed loss of dsRed-HDEL localization in the nuclear ER and even showed perturbations in the peripheral ER staining (Fig. 5e). This result suggests that VA induced stress lead to altered ER architecture and thus its associated functions including membrane lipid biogenesis. Additionally, the cells treated with VA restore the normal ER architecture after withdrawal of VA from growth media (Fig. S9b). As VA treatment leads to accumulation of neutral lipids and depletion of inositol levels to exert its effects, then we reasoned whether the cells lacking inositol and neutral lipids can tolerate VA induced stress. Surprisingly, a quadruple mutant (*dga1∆lro1∆are1∆are2∆*) that lacks the ability to sustain normal growth on inositol lacking medium (Ino^−^ phenotype) and synthesize neutral lipids triacylglycerols (TAG) and steryl esters (SE)^25^ grows better than wild-type cells in the presence of VA (Fig. 5f), and thus suggests that VA mediate its effects through inositol and neutral lipids (lipid droplets). Taken together, our results revealed that VA treatment alters lipid homeostasis and probably by alteration of ER morphology and functions.

### Valproic acid induces cell wall biogenesis genes and activates Slt2 MAP Kinase of cell wall integrity (CWI) pathway

**Figure 6:**
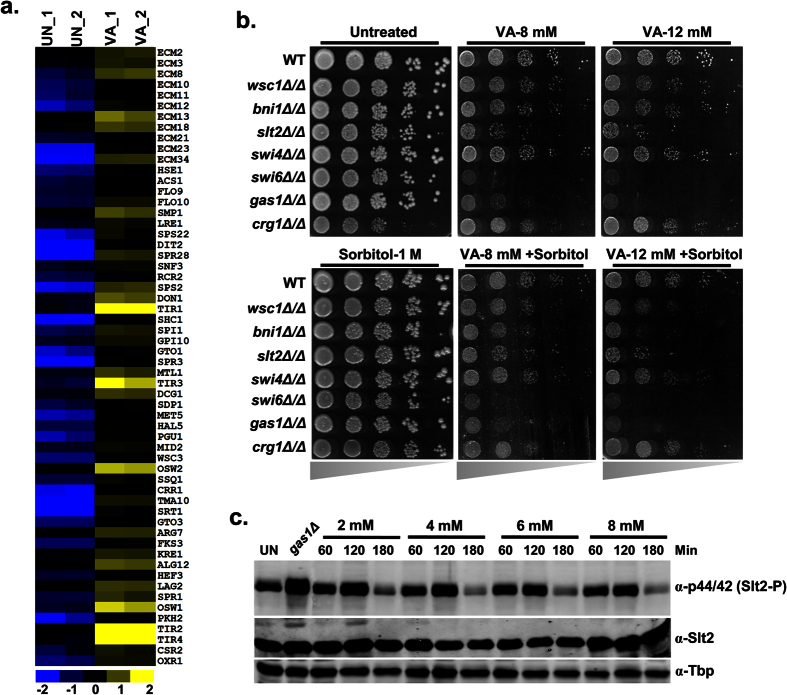
Valproic acid activates Slt2 MAP Kinase of Cell Wall Integrity (CWI) pathway. (**a**) VA exposure leads to transcriptional upregulation of cell wall biogenesis and maintenance genes. The relative expression values (log_2_) for every gene in each replicate was illustrated by blue-yellow color scale (yellow color indicates up-regulation whereas blue color for down-regulation) in the heatmap. (**b**) Loss of CWI pathway components leads to enhanced VA sensitivity, and intact CWI pathway is required for VA tolerance. The growth of wild-type (BY4743) and CWI pathway null mutants on SC-Agar plates supplemented without or with VA (8 and 12 mM), Sorbitol (1M) and both was imaged after 72 h. (**c**) VA treatment leads to the activation of central Slt2 MAPK of CWI pathway and indicates VA induced cell wall damage. Whole-cell TCA protein extracts of wild-type (W1588-4C) cells that were left untreated (UN) or treated with different doses of VA (2, 4, 6 and 8 mM) for indicated time points at 24 °C were analyzed by immunoblotting using indicated antibodies. Anti-Tbp was used as a loading control. *gas1Δ* cells were used as a positive control for Slt2 activation.

Earlier, VA was shown to affect membrane trafficking and cell wall integrity (CWI) in fission yeast^26^. Accordingly, our VA transcriptome also evidenced significant enrichment (Fig. 2d) and upregulation of cell wall biogenesis and assembly genes (Fig. 6a). In budding yeast, CWI pathway contributes to cell wall biogenesis, reorganization, and maintenance through Slt2 MAP Kinase (MAPK) signaling cascade^27^. To investigate the genetic targets required for mediating VA effects, we performed a growth assay with CWI pathway null mutants. Our results showed that CWI pathway mutants including MAPK (*SLT2*), transcriptional factor (*SWI6*), and *GAS1* exhibited considerable sensitivity, whereas sensor (*WSC1*) and effector (*BNI1*) exhibited moderate sensitivity to VA, suggests the essential role of CWI in VA tolerance (Fig. S10). To rescue the VA sensitive CWI pathway mutants, we supplemented the growth medium with sorbitol as an osmotic stabilizer and cell wall protective agent. To our surprise, the sensitivity of CWI mutants didn’t reverse despite the presence of sorbitol in the medium along with VA (Fig. 6b), suggests that the sensitivity of CWI mutants to VA doesn’t credit for osmotic perturbation. The central MAPK Slt2 of CWI pathway gets activated in response to diverse stress conditions and achieves cellular homeostasis^27^. Our results motivated us to ask whether VA activates Slt2 MAPK or not. Although the levels of unphosphorylated Slt2 (Mpk1) were unaltered, the abundance of Phospho-Slt2 (Anti-p44/42) was increased upon VA treatment for 2 h compared to untreated cells and later decreased (Fig. 6c). Altogether, our results demonstrated that VA treatment induces cell wall biogenesis genes and activates Slt2 MAPK of CWI pathway.

### Valproic acid activates Hog1 (p38) MAP Kinase of high osmolarity glycerol (HOG) pathway

**Figure 7:**
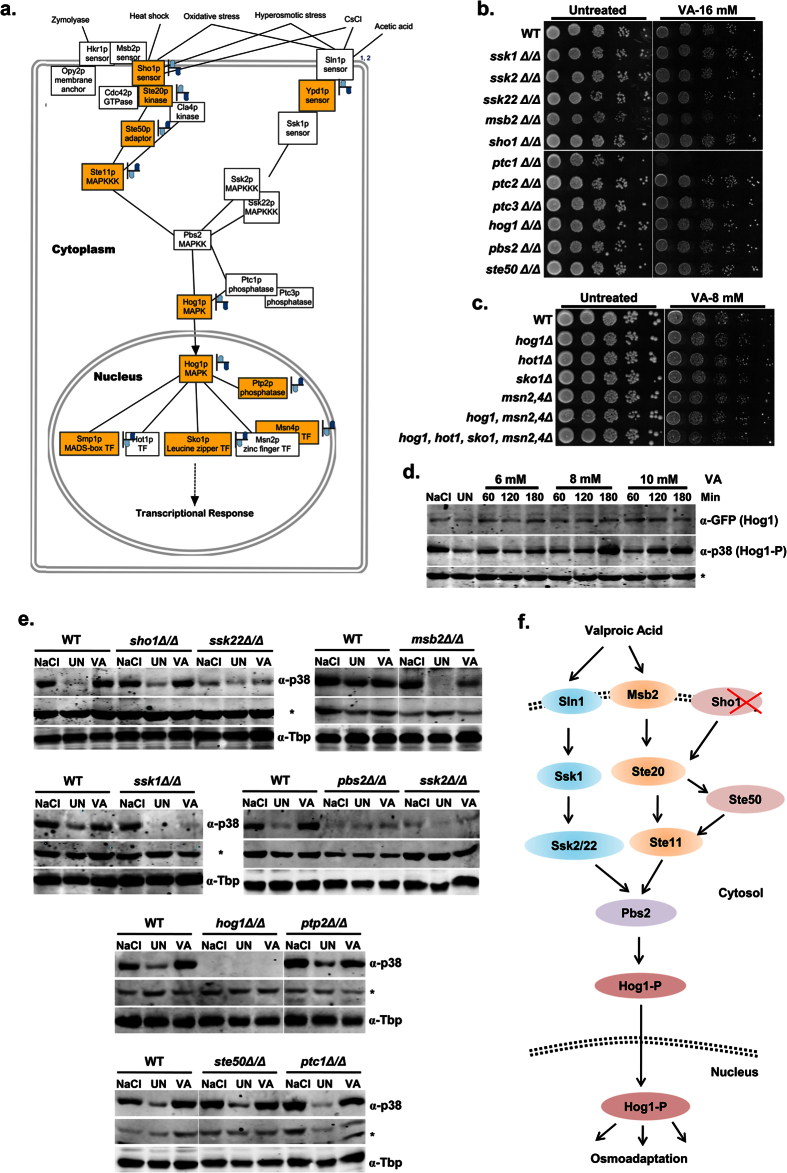
Valproic acid activates p38 MAP Kinase of High Osmolarity Glycerol (HOG) pathway. (**a**) Enrichment of HOG pathway in VA transcriptome and the pathway map showing the components that were differentially regulated upon VA treatment in ‘yellow’ color. (**b**,**c**) Loss of *PTC1*, a phosphatase of Hog1 MAP kinase leads to VA sensitivity. The growth of wild-type (BY4743), HOG pathway single null mutants (**b**) and combined null mutants generated in *EY0690* wild-type (see Table S1) (**c**) on SC-Agar plates supplemented without or with indicated doses of VA was imaged after 72 h. (**d**,**e**) VA treatment leads to the activation of central Hog1 MAPK of HOG pathway that responds to osmotic stress through Sln1 and not Sho1 branch. Whole-cell TCA protein extracts from Wild-type cells expressing GFP-tagged Hog1 (*EY2335*) (**d**) or wild-type BY4743 cells along with individual HOG pathway null mutants (**e**) that were left untreated (UN) or treated with mentioned doses of VA for indicated time points (**d**) or 8 mM of VA for 3 h (**e**) at 27 °C were analyzed by immunoblotting using indicated antibodies. ‘*’ Represents a non-specific band in the same blot of *p38* served as loading control along with Anti-Tbp. Cells treated with a standard osmolyte NaCl (0.8 M) for 30 min were used as a positive control for Hog1 (*p38*) activation. (**f**) Proposed HOG pathway model that is functional during VA exposure for activating the Hog1 (p38) MAP kinase. The HOG pathway consists of two upstream osmosensing branches (Sln1/Msb2 and Sho1) each with a downstream MAPK cascade (Ssk2/Ssk22 and Ste11 MAPKKKs, Pbs2 MAPKK, and Hog1 MAPK). Activation of the Hog1 by VA leads to its rapid translocation into the nucleus, which in turn activates the expression of stress-responsive genes via several transcription factors.

Valproic acid activates *p38* MAPK pathway that regulates stress-responsive genes in human cell line^28^. In yeast, Hog1 (homolog of mammalian *p38*) also encodes an MAPK that integrated into HOG pathway, which activates in response to hyperosmotic stress^29^. Interestingly, our pathway mapping analysis revealed that different components of HOG pathway including sensors (*SHO1/STE20/YPD1*), transducers (*STE50/STE11/HOG1*), and target TFs (*MSN4/SMP1/SKO1*) were differentially regulated in VA transcriptome (Fig. 7a). To identify the VA genetic targets, we then screened individual null mutants (Fig. 7b) and combined null mutants (Fig. 7c) of HOG pathway for VA sensitivity. Surprisingly, we found none of the critical HOG pathway mutants exhibited sensitivity to VA except *ptc1∆* (Fig. 7b). *PTC1* encodes a type-2C protein phosphatase (*PP2C*) that dephosphorylates Hog1 and inactivates osmosensing MAPK cascade once the cells achieve equilibration to the hyperosmotic environment^30^. Although Hog1 is not essential for survival against VA induced stress, we reasoned whether VA activates Hog1 (p38) MAPK similar to that of mammalian cells or not. To check the same, we have analyzed the activation of GFP-tagged Hog1 in wild-type yeast cells by immunoblotting. Remarkably, cells treated with VA (8 and 10 mM) showed activation of Hog1 as indicated by the increased levels of phosphorylated Hog1 compared to its unphosphorylated form (Fig. 7d). Our results were in consistent with the earlier findings that showed p38 activation in mammalian cell lines by VA to elicit its effects^28^.

As HOG pathway is a signaling cascade, activation of central Hog1 MAPK engages several upstream kinases and sensors to mediate hyperosmotic stress response^29^. Further to investigate the upstream targets required for Hog1 activation in response to VA, we analyzed the Hog1 phosphorylation levels in single null mutants of HOG pathway (Fig. 7e). Interestingly, *ssk1∆* and *msb2∆* cells were defective in Hog1 activation upon VA treatment, while they exhibited normal phosphorylation upon NaCl (standard osmolyte) treatment (Fig. 7e). Our findings suggest the primary role of these specific sensors (Ssk1, Msb2) and not Sho1 in optimal Hog1 activation against VA induced stimuli. Moreover, cells lacking the transducers (*ssk2∆, ssk22∆, pbs2∆*) also failed to activate Hog1 upon treatment with VA and NaCl, while others (*ste50∆, ptc1∆, ptp2∆*) showed normal Hog1 activation. Thus, our results identified the critical upstream regulators required for Hog1 activation in yeast cells upon VA treatment (Fig. 7f). Altogether, our results indicate that VA activates *p38* (Hog1) and hence induce osmotic and other stress response genes.

## Discussion

In this study, we investigated the molecular mechanisms that VA perturbs to mediate its effects by global transcriptional profiling and target genes that are required for VA tolerance by chemical genetics approach using budding yeast as a model organism. Eukaryotic cells evoke a series of responses to cope up with the environmental cues by regulating a specific set of genes^31^. Indeed, cells treated with VA also evidenced differential regulation of approx 30% of the genome. We performed enrichment analysis of pathways for upregulated and downregulated genes separately, which is more powerful than that of all DEGs together for finding significant functional pathways^32^. VA exhibits its effects by altering several cellular processes and functions as evidenced by our GO enrichment analysis (Figs 2 and S4–5). VA treatment leads to growth arrest in G1-phase of the cell cycle through activation of cyclin-dependent kinase inhibitors and thus apoptosis of human cancer cells^33^. Transcriptional upregulation of G1- and G1/S-phase transition genes of mitotic cell cycle (Fig. S5) and dose-dependent growth inhibition (Fig. 1) suggests that induction of apoptosis in *S. cerevisiae* by VA possibly mediated through G1-phase arrest^16^.

Oxidative process mediated production of ROS is a prominent characteristic of apoptosis and thus cell death. VA induced apoptosis in yeast also features increased production of ROS^16^. Moreover, genetic targets identified through our screening indicate the critical role of intracellular levels of reduced glutathione (GSH), an essential antioxidant in tolerating VA mediated oxidative stress (Fig. 4b). Our results further substantiated earlier reports indicating that VA treatment leads to depletion of GSH levels^34^, a probable mechanism for VA induced oxidative stress. Oxidative stress by increased ROS production primarily by the mitochondrial respiratory chain and the ER UPR machinery finally leads to cell death in yeast^35^. In earlier, VA was known to alter iron metabolism^36^, mitochondrial biogenesis and respiratory functions^37^, and mitochondrial epigenetics^38^. Functional mitochondria are the source of ISCs and heme–prosthetic groups and thus crucial for iron homeostasis^39^. Accordingly, our transcriptional signatures also detailed the effects of VA on iron homeostasis, mitochondrial functions and mitochondrial architecture (Fig. 3). Our findings were in line with that of earlier studies showing VA effects on mitochondrial functions in *S. cerevisiae*^40^ and fission yeast^41^.

Both ER and mitochondria interact physiologically and functionally, including Ca^2+^ signaling, energy metabolism, and lipid exchange for cell survival. In this study, our results indicated that VA treatment leads to redistribution, alteration of ER architecture (Fig. 5e) and induction of UPR stress response genes in agreement with its effects on mitochondrial functions (Fig. 3). These findings are in line with earlier studies showing that VA regulates UPR stress^42^ and targets protein secretory functions of ER for exhibiting its effects^40^. At higher doses, VA treated yeast cells show morphological features similar to that of autophagic cell death (ACD)^43^, an integral response of a cell to ER stress. Thus, it’s plausible that VA incites ACD in yeast by modulating ER stress. In addition to protein folding and maturation, ER is also a major site for lipid biosynthesis and metabolism^44^. Depletion of intracellular inositol levels^21^ and accumulation of neutral lipids by VA to induce ACD^45^ also indicate its effects on lipid homeostasis. Neutralization of VA effects on a quadruple null mutant that is inositol auxotrophic and lacks the lipid particles hints a mechanism to detoxify VA, needs further studies to check its effectiveness in presence or combination with inhibitors of lipid droplet formation.

Enhanced ROS accumulation damages cellular macromolecules and leads to genomic instability. In eukaryotes, cellular checkpoints monitor the genome integrity and accordingly activate the genotoxic stress responses and DNA damage-induced apoptosis in complex organisms^46^. Intriguingly, transcriptional signatures of DNA repair (NHEJ, NER, BER, MMR, and SSA) genes and prime role of Mec1/Rad53/Dun1-kinase checkpoint components in VA tolerance indicates the probable DNA replication stress (Fig. 4). The resistance of *IXR1* null mutant which encodes an HMG domain-containing protein to VA is in accordance with an earlier study that shows VA exacerbate innate immune responses to endotoxin by enhancing the release of High-mobility group box-1 (HMGB1)^47^. Altogether, our results demonstrated that VA mediates its toxicity through alteration of genomic stability, redox and lipid homeostasis, ER and mitochondrial functions.

In response to a stress condition, eukaryotic cells coordinate multiple protective effects through a cascade of signaling events for acclimatization and transcription of condition-specific stress defence genes^31^. Of particular importance are the MAPK pathways well characterized in mammals and conserved in other organisms including *D. melanogaster*, *S. pombe,* and *S. cerevisiae*^29^. Strikingly, VA treatment leads to the activation of Hog1 MAPK and renders HOG pathway to be considered as its one of functional targets (Fig. 7d). In *S. cerevisiae*, the activation of Hog1 regulated by any of Sln1 and Sho1 branches, which converges on MAPK kinase Pbs2^29^. As indicated by our results, VA markedly acts through a Sln1 branch (Fig. 7e,f), which has a far more prominent role in the regulation of HOG pathway in response to osmotic changes than Sho1 branch^48^. It was evident from prior studies that both osmotic stress and cell wall damage are related very closely, and also the interplay between HOG and CWI pathways was well observed earlier^49^. Activation of Slt2 (*p44/42*), a central MAPK of CWI pathway in addition to that of Hog1 indicates the possible deleterious effects of VA on cell wall (Fig. 6c). Our results are in agreement with earlier findings that showed alteration of membrane trafficking and cell wall integrity by VA in fission yeast^26^. In budding yeast, both HOG and CWI pathways get activated in response to a variety of stimuli including ER stress, oxidative stress, osmotic changes and cold/heat stress^50^. Thus, it is reasonable to propose that the activation of HOG and CWI pathways in response to VA might not only due to osmotic stress, but also oxidative stress (Fig. 4), ER stress (Fig. 5), and needs further insight analysis of VA effects on different cellular processes and pathways.

**Figure 8:**
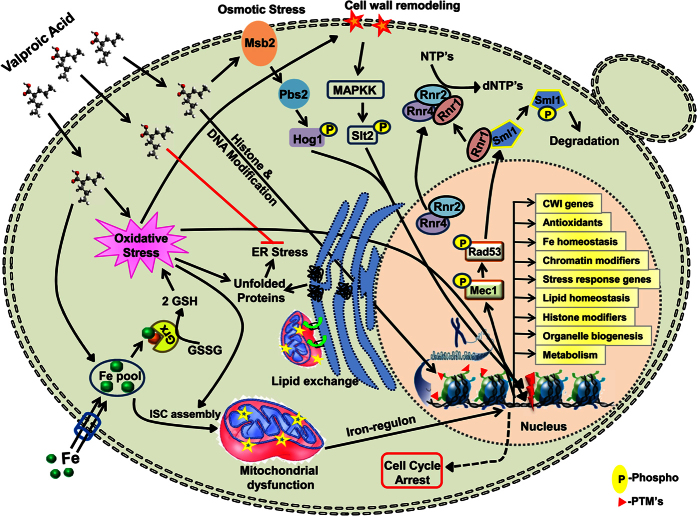
A proposed working model is depicting the molecular mode of action and transcriptional regulation of VA in budding yeast. VA alters cellular iron ion homeostasis, which is an essential element for the maintenance of iron-sulfur clusters in mitochondria. In corroboration, VA treated cells exhibited reorganization of mitochondrial structures and decreased functions of mitochondria as evidenced by enhanced VA toxicity on non-fermentable carbon source medium. Also, Iron is an essential cofactor for Grx (Glutaredoxin), which converts GSSG to reduced glutathione (GSH), which is an essential antioxidant that scavenges cellular ROS and thus achieves cellular redox homeostasis. Notably, the sensitivity of antioxidant mutants suggests that VA treatment leads to oxidative stress in accordance with earlier findings. Alteration in redox homeostasis leads to genomic instability. Our results also suggest that VA treatment leads to genomic stress that resulted in degradation of Sml1, a negative regulator of RNR complex and hence dNTP synthesis. We have also evidenced that VA interferes with ER organization and thus resulted in transcriptional regulation of lipid homeostasis and suppression of unfolded protein response. Moreover, major MAP Kinases Hog1 (*p38*) of Osmotic stress and Slt2 (*p44/42*) of CWI pathway that responds to diverse stress conditions also got activated upon VA exposure and thus leads to transcriptional induction of stress-responsive genes and CWI genes. As VA is HDAC inhibitor, the entire transcriptional regulation of genes of diverse cellular pathways by VA might be credited to its effects through alteration of histone and DNA modifications.

In conclusion, we have illustrated a model describing key findings of this study obtained through chemical genetics and transcriptomics approaches that led us to understand the molecular mode of action of VA in budding yeast (Fig. 8). Taken together, our detailed transcriptomics and genetic screening findings shed light on effects of VA on different organelles and diverse cellular processes that aid in understanding the molecular mechanisms of action including antimicrobial effects and detoxification approaches of VA, a pharmacologically important anticancer molecule. The extensive conservation of basic cellular processes, biochemical pathways and signaling mechanisms in yeast favours the need of further studies in eukaryotic mammalian models to understand the VA mediated diverse effects.

A summary of this paper was presented at the 27^th^ International Conference on Yeast Genetics and Molecular Biology (ICYGMB), September 2015^51^,^52^.

## Methods

### Yeast Strains, Chemicals, Growth Media, and Growth Conditions

The *Saccharomyces cerevisiae* strains used in this study were listed in Supplementary Table S1. The sodium salt of Valproic acid (P4543; ≥98%) was procured from Sigma-Aldrich India. Media components and all other reagents used in this study were of molecular biology grade and purchased from Sigma–Aldrich India, Merck India, Himedia India, GE Healthcare India, Invitrogen India, New England BioLabs and Thermo Scientific India. Unless stated otherwise, all yeast strains used in this study were grown at 30 °C in standard Synthetic Complete (SC) liquid media containing 2% glucose. SC liquid media was prepared by mixing all amino acids, yeast nitrogen base (YNB) and ammonium sulfate (AS) together by following a standard protocol (Yeast Protocols Handbook, Clontech Laboratories, Inc.). For solid agar media, 2% Bacto-agar was used in addition to SC media components.

### Growth Sensitivity and Growth Curve Assays

Effect of VA on the growth of yeast cells was examined by using growth sensitivity assay as described previously^53^. In brief, 3 μl of each ten-fold serially diluted wild-type (WT) and mutant cultures was spotted onto solid SC-agar plates supplemented with 2%-glucose or 2%-glycerol without (control) or with the addition of VA at indicated concentrations. All the plates were incubated at 30 °C and growth was recorded after 72 h using HP scanner.

For growth curve analysis, exponentially growing yeast cells were left untreated (control) or treated with indicated concentrations of VA and seeded in a 96-well cell culture plate (SPL Life Sciences Ltd.) in triplicate. Growth curves were constructed for each treatment from averaged values of optical density (OD_600_) measured at a regular interval of time for indicated period using plate reader (Eon™ Microplate Spectrophotometer).

### Clonogenic Assay

For testing colony forming ability of yeast cells after VA treatment, clonogenic assay was performed as described previously^54^. Exponentially growing wild-type (1588-4C) yeast cells in SC liquid media were left untreated (control) or treated with VA (2, 4, 6, and 8 mM) for 3 h at 30 °C. Theoretically, an equal number of cells (normalized by absorbance) from each treatment were spread onto standard YPDA (1% yeast extract, 2% Bacto peptone, 2% Dextrose, and 2% bacto-agar) plates and incubated at 30 °C for 72 h. The number of colonies from two independent experiments were counted in each plate and represented as average % survival relative to untreated control.

### Total RNA Isolation and Global Transcriptome Analysis

The exponentially growing wild-type yeast (1588-4C) cells were left untreated or treated with VA (6 mM) for 3 h at 30 °C in SC liquid media and then cells were harvested. Total RNA was isolated by heat/freeze Phenol method as described earlier^55^ and assessed the integrity and quality of RNA by absorbance (260/280), Formaldehyde Agarose (FA)-gel electrophoresis, and by Agilent 2100 Bioanalyzer (Agilent Technologies, CA) before proceeding with microarray analysis. These DNA-free RNA samples from two independent biological repeats of each untreated and VA treated cells were processed at iLife Discoveries (Gurgaon, India) for gene expression profiling using Affymetrix platform as mentioned previously^55^. Briefly, biotinylated complementary RNA (cRNA) was prepared from 6 μg of total RNA using *in vitro* transcription (IVT) reaction and then hybridized to Yeast Genome 2.0 GeneChip Arrays (GPL2529) at 45 °C for 16 h at 60 rpm according to standard Affymetrix protocol. Afterward, GeneChips were washed, stained in the Fluidics Station 450 (Affymetrix) and scanned using the GeneArray 3000 7G microarray scanner. The data sets were extracted from all CEL (raw intensity) files and submitted to NCBI’s Gene Expression Omnibus (GEO) repository with a GEO Series accession number of GSE62400 (http://www.ncbi.nlm.nih.gov/geo/query/acc.cgi?&acc=GSE62400).

The raw signals (CEL) in each array were processed for background adjustment, normalization followed by log transformation and summarization of probe sets using RMA (Robust Multi-array Average) algorithm in GeneSpring GX 12.6 expression analysis software (Agilent Technologies, CA). Differentially expressed genes (DEG’s) whose expression altered (induced or repressed) significantly by two-fold (≥2) in VA treatment compared to untreated were determined by moderated t-test (p ≤ 0.05) and considered for functional analysis. The averaged data was normalized by experiments and clustered by hierarchical clustering (Euclidean distance). Functional classification, Gene Ontology (GO) enrichment and pathway mapping analysis of DEG’s were performed using standard tools as described earlier^55^ and the details for the same were provided in the Supplementary Information.

### Reverse Transcriptase PCR (RT*-*PCR) and quantitative PCR (qPCR)

The exponentially growing wild-type yeast (1588-4C) cells were left untreated or treated with VA (6 mM) for 3 h at 30 °C in SC liquid media and then cells were harvested. Total RNA was isolated by heat/freeze Phenol method as described earlier^55^. 1 μg of DNA-free RNA was reverse transcribed to cDNA as per method supplied by iScript cDNA Synthesis Kit (Bio-Rad, India). Then PCR reaction was performed with primers of *HAC1-S* and *ACT1* (See Table S2 for F/R primers). The PCR amplicon products were electrophoresed, stained with ethidium bromide, visualized and photographed. A representative image from at least two independent experiments for each condition was shown. For validation of microarray data, each qPCR reactions for selected genes were performed at least in duplicate on ABI-7300 RT-PCR with Sequence Detection System v1.4 (Applied Biosystems, CA) according to the conditions and protocol provided with VeriQuest SYBR Green qPCR Master Mix (Affymetrix, CA). Relative expression values (fold change) were calculated according to the ΔΔC_*T*_ method^56^ using actin (*ACT1)* as a reference. Gene-specific primers used in this study were listed in Supplementary Table S2.

### Preparation of Protein Extracts and Immunoblotting Analysis

Exponentially growing yeast wild-type or mutant cells were left untreated (control) or treated with specified dose of VA, incubated at indicated temperatures and harvested. Whole cell protein extracts were prepared by using 20% Trichloroacetic acid (TCAA) precipitation method as described previously^57^. The protein extracts were resolved by electrophoresis on an SDS-polyacrylamide gel and stained with 0.5% of Coomassie Brilliant Blue R-250 (CBBR). Immunoblotting analysis of protein extracts was performed by following a standard protocol. Briefly, the extracts were transferred to nitrocellulose membranes that were then blocked for 45 min using blocking buffer (2.5% Bovine serum albumin in TBST; TBS containing 0.05% Tween-20) followed by incubation with primary antibodies for 90 min. After washing with TBST, the membranes were incubated with relevant secondary antibodies such as IRDye^®^ 800CW Goat anti-Rabbit IgG or anti-Mouse IgG (1:15,000, LI-COR Biosciences) for 45 min. Blots were scanned by using Odyssey infrared imager (LI-COR Biosciences). Anti-Rad53 western signals were detected by chemiluminescence using Fuji gel-dock system (LAS-4000 mini). Following primary antibodies were used: Anti-Rnr1 (Agrisera, ASO9 576), Anti-Rnr2 (Agrisera, ASO9 575), Anti-Sml1 (Agrisera, AS10 847), Anti-Rad53 (Santa Cruz Biotechnology Inc., SC-6749), Anti-GFP (Sigma, G1544), Phospho-p38 MAPK antibody (Cell Signaling, 9211), Anti-Mpk1 (Santa Cruz Biotechnology Inc., SC-6803), Phospho-p44/42 MAPK antibody (Cell Signaling, 4370). Polyclonal antibody against recombinant yeast Tbp protein was raised in rabbit. All primary and secondary antibodies were diluted in blocking buffer. The representative images obtained from at least two independent experiments were shown.

### Microscopy

To investigate the effects of VA on mitochondria and ER, we analyzed yeast cells harboring Psd1-3Xmcherry (mitochondrial resident) or ss-dsRed-HDEL reporter (stains cortical and nuclear ER) by ApoTome microscopy^24^. A single colony of these strains was inoculated in 5 ml SC-liquid media and grown overnight at 30 °C. Cells were transferred to fresh media and cultured till exponential phase, left untreated or treated with VA (4 and 6 mM). After 3 h treatment with VA, the cells were washed, resuspended at an equal OD600 of 0.8 in fresh SC-liquid media and then allowed to grow further at 30 °C for 3 h. The cells were visualized just after 3 h treatment with VA and removal of VA from growth media using 63x oil-immersion objective lens of ZEISS ApoTome.2 microscope provided with an appropriate filter. Images were processed via ZEN-2012 (Blue Edition) software.

### *β*-Galactosidase Activity Assay

The *β*-galactosidase assay was performed to monitor the expression levels of *UPRE-lacZ* reporter gene as described previously^58^. Briefly, 0.2 ml of a fresh overnight culture of wild-type (BY4741) yeast cells carrying 2 μ *UPRE-lacZ* reporter plasmid (pPW344; gifted by Laran T. Jensen)^23^ was inoculated into 5 ml of fresh SC liquid media lacking Uracil and incubated at 30 °C. Exponentially growing cells (OD_600_: 0.8–1) were then left untreated or treated with indicated doses of VA (2, 4, 6 mM) and tunicamycin (1 μg/ml). Tunicamycin-treated cells were served as a positive control, harvested after 1 h incubation. Untreated and VA treated cells were harvested after 2 h and 3 h of incubation. Before harvesting the cells, the cell density at OD_600_ was determined. The β-gal activity was measured by permeabilizing cells in 1 ml of Z buffer using ONPG as substrate at room temperature. The galactosidase activity was expressed in terms of Miller units as described earlier^59^. The results are the average values from two independent experiments each performed in triplicate.

## Additional Information

**How to cite this article**: Golla, U. *et al.* Combined Transcriptomics and Chemical-Genetics Reveal Molecular Mode of Action of Valproic acid, an Anticancer Molecule using Budding Yeast Model. *Sci. Rep.* **6**, 35322; doi: 10.1038/srep35322 (2016).

## Supplementary Material

Supplementary Information
