# Supplementary material for: Combined Transcriptomics and Chemical-Genetics Reveal Molecular Mode of Action of Valproic acid, an Anticancer Molecule using Budding Yeast Model
Source: Sci Rep. 2016 Oct 13;6:35322. doi: 10.1038/srep35322 (PMC5062167; doi:10.1038/srep35322)
Supplement: Supplementary Information [file srep35322-s1.pdf]

Supporting Information

Combined Transcriptomics and Chemical-Genetics Reveal Molecular Mode of Action of Valproic acid, an Anticancer Molecule using Budding Yeast Model

[1]Upendarrao Golla, [1]Deepthi Joseph, and [1]Raghuvir Singh Tomar*

[1]Department of Biological Sciences, Indian Institute of Science Education and Research (IISER), Bhopal-462066, India

Contents of Supporting Information

Number of pages: 145

Figures: S1-S10

Tables: S1-S6

***Correspondence to be addressed:** Raghuvir S. Tomar, Associate Professor, Department of Biological Sciences, Indian Institute of Science Education and Research, Bhopal–462066, India. Tel.: +91-755- 6692560; Fax: +91-755- 4092392; Email: rst@iiserb.ac.in

SUPPLEMENTARY METHODS:

Cell Viability Assay: The viability of yeast cells after VA treatment was assessed by using methylene blue (vital dye) staining method as described earlier[1]. Exponentially growing wild-type (1588-4C) yeast cells were left untreated (control) or treated with VA (2, 4, 6, and 8 mM) for 3 h at 30 ^{o}C. A fraction of cells from each treatment were stained with 3.7% buffered methylene blue solution. Heat killed (70 ^{o}C/15 min) cells were served as a positive control. Cells stained dark blue were considered as metabolically inactive or dead. The staining of yeast cells was recorded by using a LEICA DM500 microscope (installed with LAS EZ V1.7.0 software) at 400X total magnification.

Functional Enrichment Analysis of VA Transcriptome: The DEG's were systematically classified into MIPS (Munich Information Center for Protein Sequences) functional categories using Functional Catalogue Database (FunCatDB; available at http://mips.helmholtz-muenchen.de/funcatDB/)[2], and evaluated for enrichment of functional gene clusters using a web-based tool, FunSpec (http://funspec.med.utoronto.ca)[3]. Moreover, GO clustering and enrichment analysis was performed using Biological Networks Gene Ontology (BiNGO) tool (http://apps.cytoscape.org/apps/bingo)[4] and Gene Ontology Enrichment Analysis Software Toolkit (GOEAST, available at http://omicslab.genetics.ac.cn/GOEAST/)[5] respectively. Significantly enriched GO categories were represented in the form of an interactive hierarchy using Cytoscape software[6]. Additionally, functional enrichment analysis of DEG's was carried out using FunCat classification in FungiFun2 tool (https://elbe.hki-jena.de/fungifun/fungifun.php)[7]. The transcriptional factors (TF's) present in DEG's were identified and ranked (based on % of transcriptome regulation) using YEASTRACT tool (http://www.yeastract.com/)[8]. Also the enrichment of chromatin features in up-regulated genes in VA transcriptome was analyzed by using ChromatinDB database (http://www.bioinformatics2.wsu.edu/ChromatinDB)[9].

SUPPLEMENTARY FIGURES:

Figure S1:

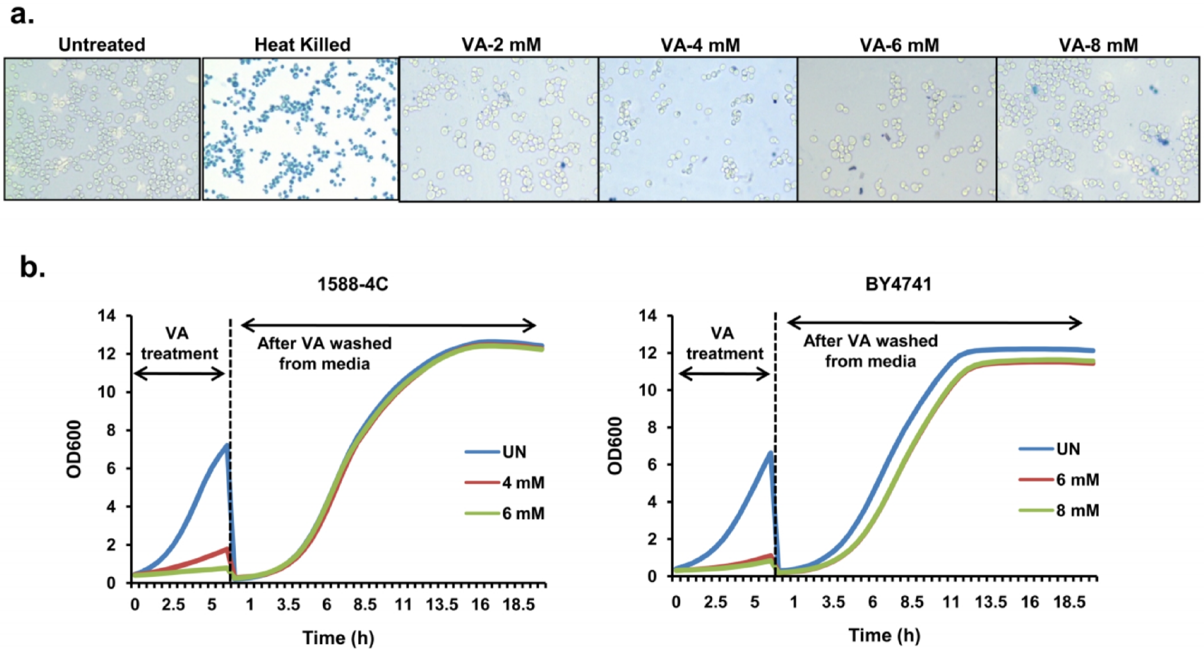

Figure S1: Acute exposure of Valproic acid (VA) reversibly inhibits growth and didn't affect the cell viability. (**a**) Exponentially growing wild-type (1588-4C) yeast cells in SC-liquid media were left untreated (Control) or treated with 2, 4, 6 and 8 mM of VA for 3 h at 30 ^{o}C. The cell viability was assessed by staining with 0.3% buffered methylene blue (vital dye) and visualized under light microscope (400X). Untreated and heat-killed yeast cells were served as negative and positive control respectively. (**b**) Exponentially growing wild-type (1588-4C and BY4741) yeast cells were left untreated (UN) or treated with indicated VA doses and the growth was recorded for 6 h at 30 ^{o}C. After VA treatment for 6 h, the cells were washed, resuspended (indicated by dotted vertical line in graph) in fresh SC-liquid media at an equal OD600 of 0.3 and the growth was monitored at a regular interval of time for 20 h using plate reader. The averaged (n=3) OD values were used to construct the growth curves.

Figure S2:

a.

Gene	Fold Change in Microarray	Regulation	Fold Change in qPCR Validation
SPS100	13.89735	up	20.79565
DMC1	2.196286	up	14.81898
SPO20	9.99191	up	13.85551
SPO13	2.285883	up	13.62829
DIT2	9.863262	up	13.60817
SPS1	4.064818	up	13.21419
DIT1	4.654425	up	12.86247
WSC3	3.557586	up	11.50155
FRE1	3.084817	up	9.108165
FET3	3.646724	up	8.520987
GIT1	-7.169636	down	0.348942
HOG1	-2.525147	down	0.669683
YLR194C	1	none	0.958176

b.

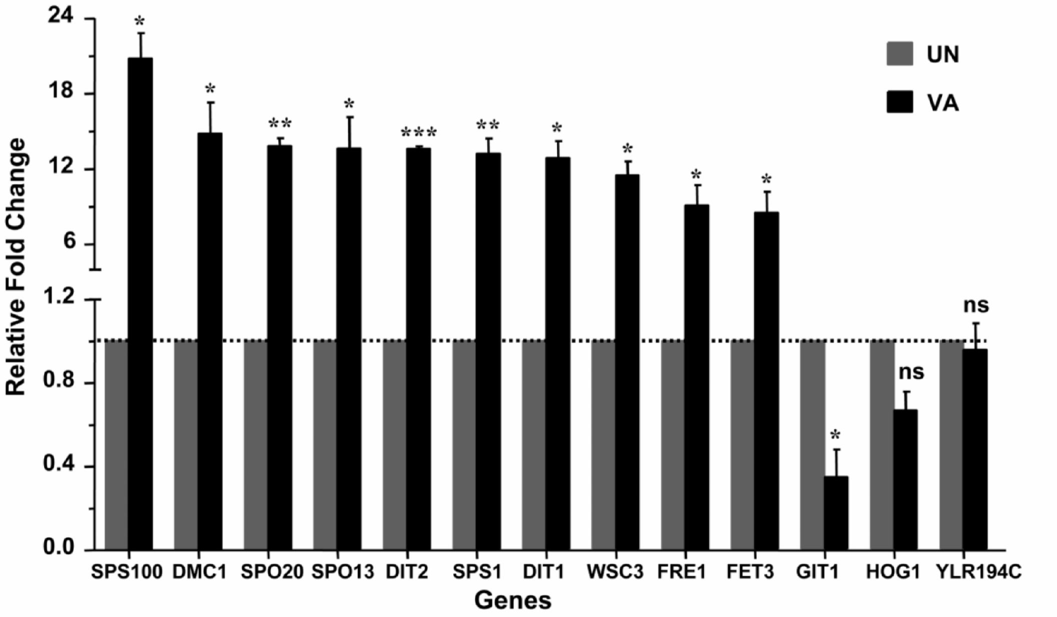

Figure S2: Validation of VA Transcriptome by RT-qPCR. (a) The table shows the list of genes selected for validation along with their relative fold change and regulation (up or down) in VA transcriptome. These selected genes represent diverse cellular processes including HOG pathway (*HOG1*), CWI pathway (*SPS100, WSC3*), heavy metal homeostasis (*FRE1*), Detoxification (*DIT2*), Meiosis (*DMC1, DIT1, SPS1, SPO13,* and *SPO20*), lipid/fatty acid transport (*GIT1*), Receptor sensor and morphogenesis (*WSC3*), Iron ion binding and transport (*FRE1* and *FET3*). (**b**) RT-qPCR. Total RNA was isolated from exponentially growing cells that were left untreated or treated with VA (6 mM) for 3 h and reverse transcribed to cDNA. The relative expression (fold change) values of selected genes were represented as Mean±SEM (n=3). *p<0.05, **p<0.01, ***p<0.001 were considered significant when compared to untreated (UN) control by Student's t-test.

Figure S3:

a.

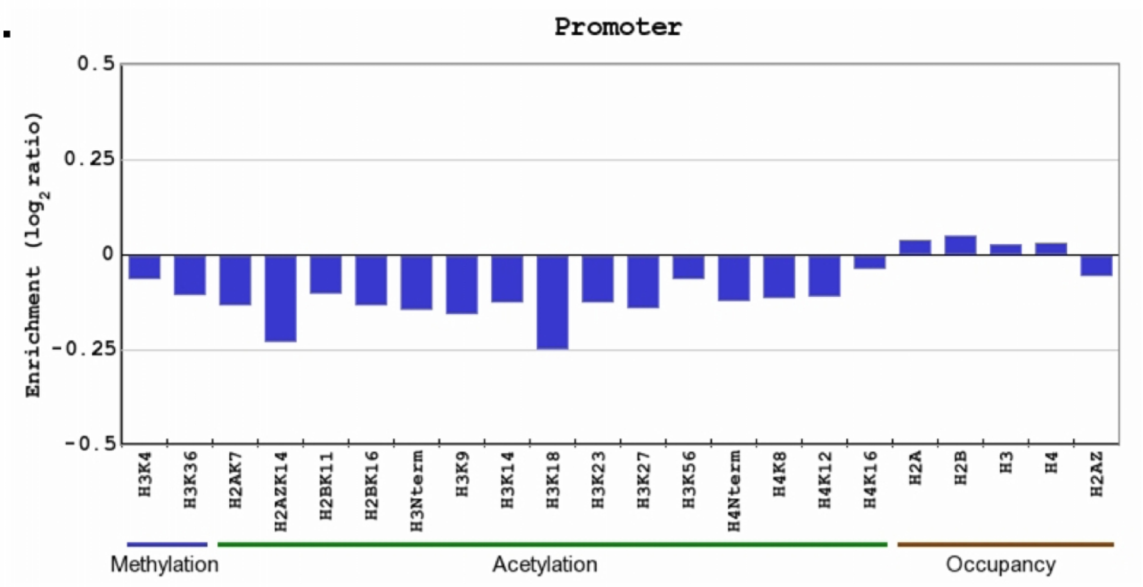

b.

Chromatin Enrichment/Depletion

Promoter

Histone Modification	Rank Percentile	Trend	P-value
H3K4 Di-Methylation	46.4 %	**Depleted**	$< 10^{-3}$
H3K36 Di-Methylation	44.7 %	**Depleted**	$< 10^{-6}$
H2AK7 Acetylation	39.6 %	**Depleted**	$< 10^{-9}$
H2AZK14 Acetylation	41.1 %	**Depleted**	$< 10^{-11}$
H2BK11 Acetylation	40.6 %	**Depleted**	$< 10^{-8}$
H2BK16 Acetylation	39.6 %	**Depleted**	$< 10^{-10}$
H3Nterm Acetylation	39.1 %	**Depleted**	$< 10^{-25}$
H3K9 Acetylation	38.5 %	**Depleted**	$< 10^{-12}$
H3K14 Acetylation	39.3 %	**Depleted**	$< 10^{-10}$
H3K18 Acetylation	35.3 %	**Depleted**	$< 10^{-19}$
H3K23 Acetylation	40.1 %	**Depleted**	$< 10^{-9}$
H3K27 Acetylation	40.8 %	**Depleted**	$< 10^{-8}$
H3K56 Acetylation	44.4 %	**Depleted**	$< 10^{-6}$
H4Nterm Acetylation	38.7 %	**Depleted**	$< 10^{-27}$
H4K8 Acetylation	40.3 %	**Depleted**	$< 10^{-8}$
H4K12 Acetylation	39.8 %	**Depleted**	$< 10^{-9}$
H4K16 Acetylation	47.2 %	none	0.0800
H2A Occupancy	54.5 %	**Enriched**	$< 10^{-4}$
H2B Occupancy	56.6 %	**Enriched**	$< 10^{-10}$
H3 Occupancy	55.8 %	**Enriched**	$< 10^{-7}$
H4 Occupancy	56.0 %	**Enriched**	$< 10^{-8}$
H2AZ Occupancy	43.5 %	**Depleted**	$< 10^{-7}$

Figure S3: Genes induced by VA treatment have enhanced histone occupancy and reduced active histone post-translational modifications (PTMs) at promoter. (**a**) Levels of Histone occupancy and histone PTMs in the promoter regions of 1,052 genes induced by VA (i.e., genes that are repressed in untreated cells) were analysed (normalized by nucleosome occupancy levels) by using the web-based ChromatinDB database. (**b**) Statistical analysis of the data shown in above indicating the trend (enriched or depleted) of histone occupancy and PTMs.

Figure S4:

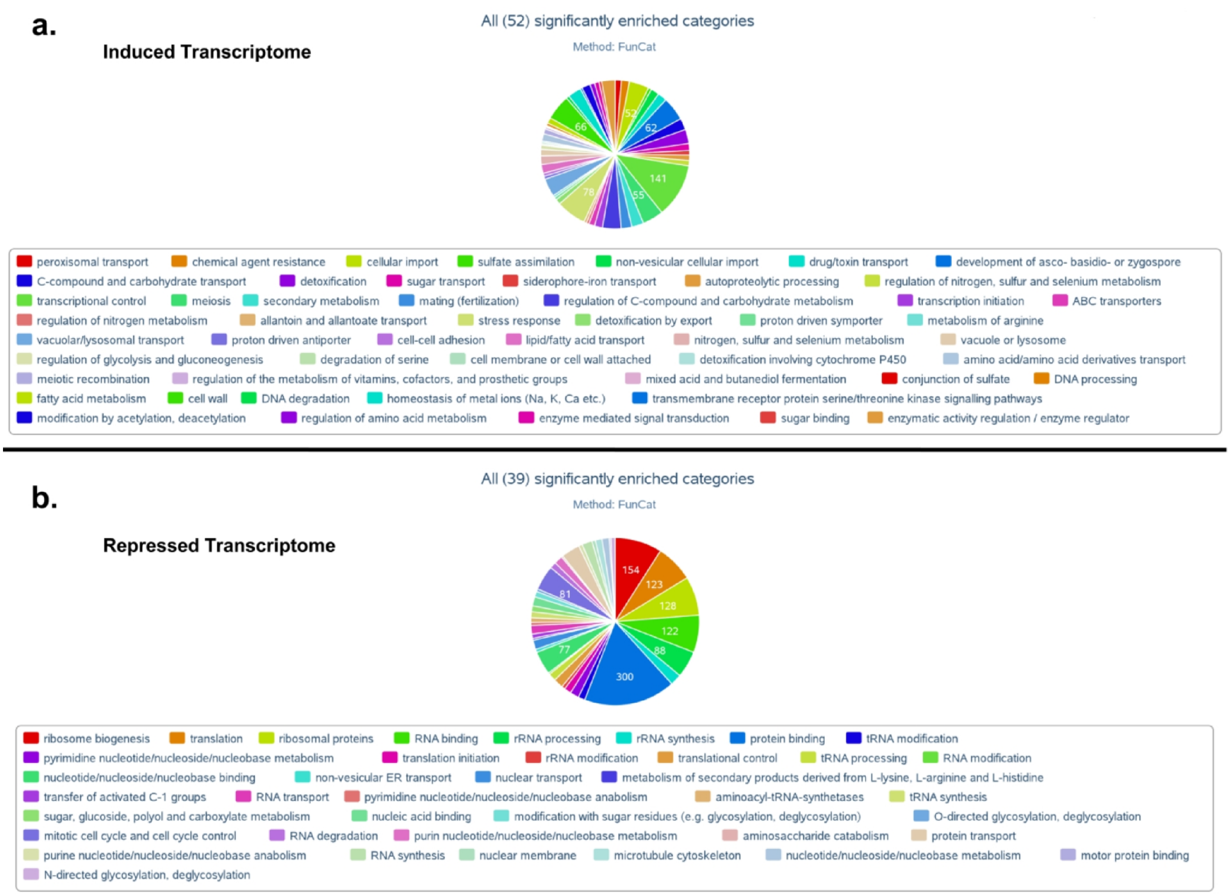

Figure S4: Functional enrichment analysis of Valproic acid transcriptome. The pie chart shows significantly enriched (over-represented) functionally annotated gene ontology (GO) processes in VA induced (**a**) and repressed (**b**) transcriptome. The analysis was performed on FungiFun tool with default significance of p<0.05.

Figure S5:

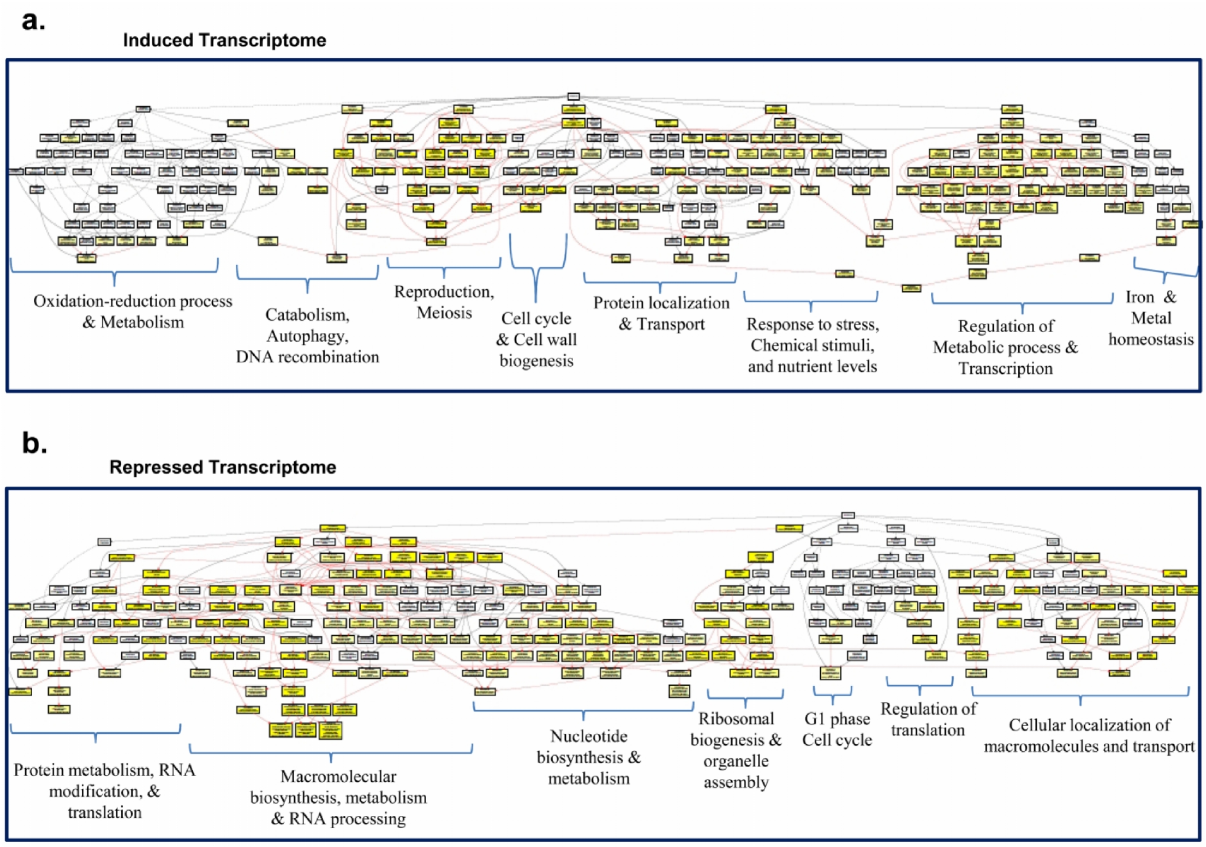

Figure S5: Gene Ontology (GO) enrichment analysis of VA transcriptome. Significantly Induced (**a**) and repressed (**b**) transcriptome (>2 fold) of VA was analyzed using GOEAST tool (default settings) to determine significantly enriched biological process. The hierarchical network of entire set of GO terms (biological processes) represented by each box. The intensity of yellow color represents the significance of GO term enrichment. The black arrows represent the connections among different processes, whereas red colored arrows specifically represents the connections among only enriched GO terms.

Figure S6:

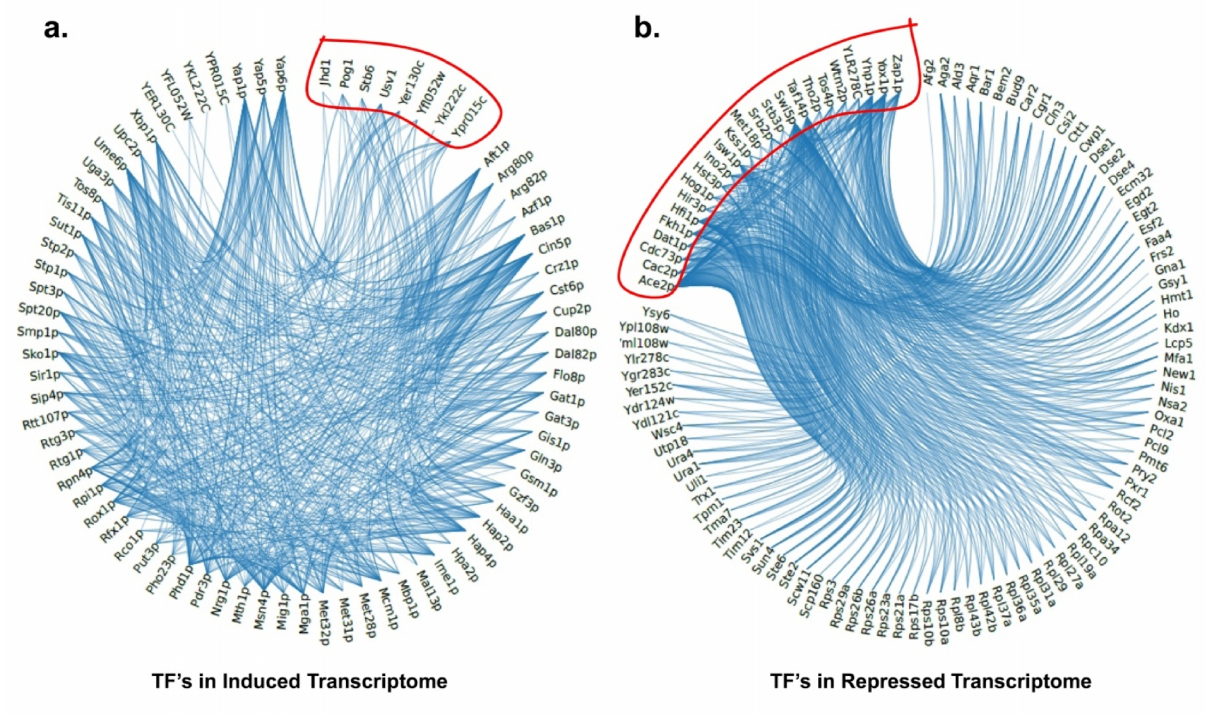

Figure S6: Transcriptional factors (TFs) were regulated by Valproic acid treatment. The transcriptional factors (TFs) that potentially involved in induced (**a**) and repressed (**b**) transcriptome of VA in yeast were identified by using YEASTRACT tool. The target genes of most TFs in VA transcriptome in common were highlighted in red colored box and the network between the target genes and TFs was shown by blue colored lines.

Figure S7:

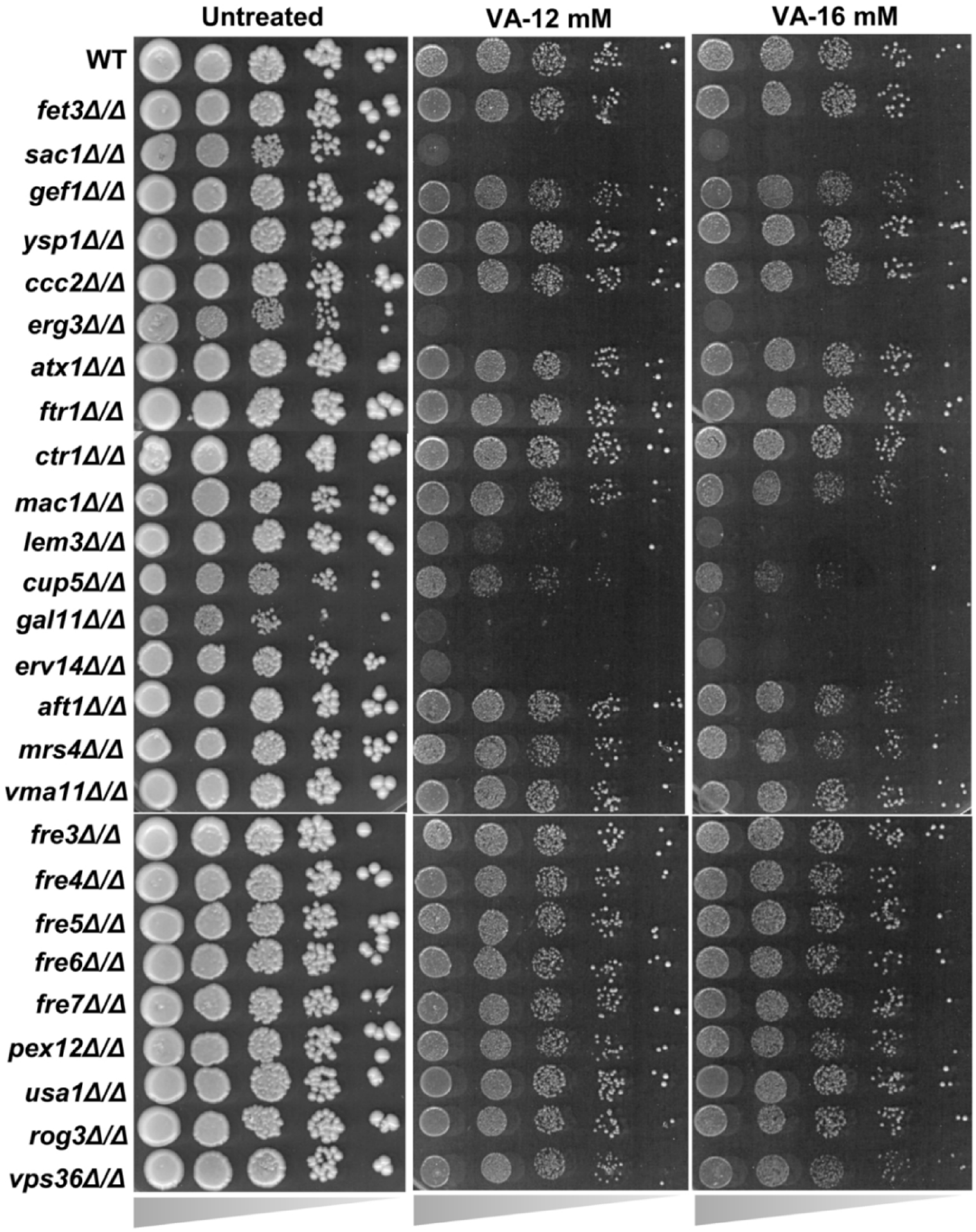

Figure S7: Screening of iron homeostasis mutants for tolerance against Valproic acid. 3 μL of ten-fold serially diluted cultures of iron homeostasis mutants was spotted onto SC-Agar plates supplemented without or with VA (12 and 16 mM). The plates were imaged after 72 h incubation at 30 ºC.

Figure S8:

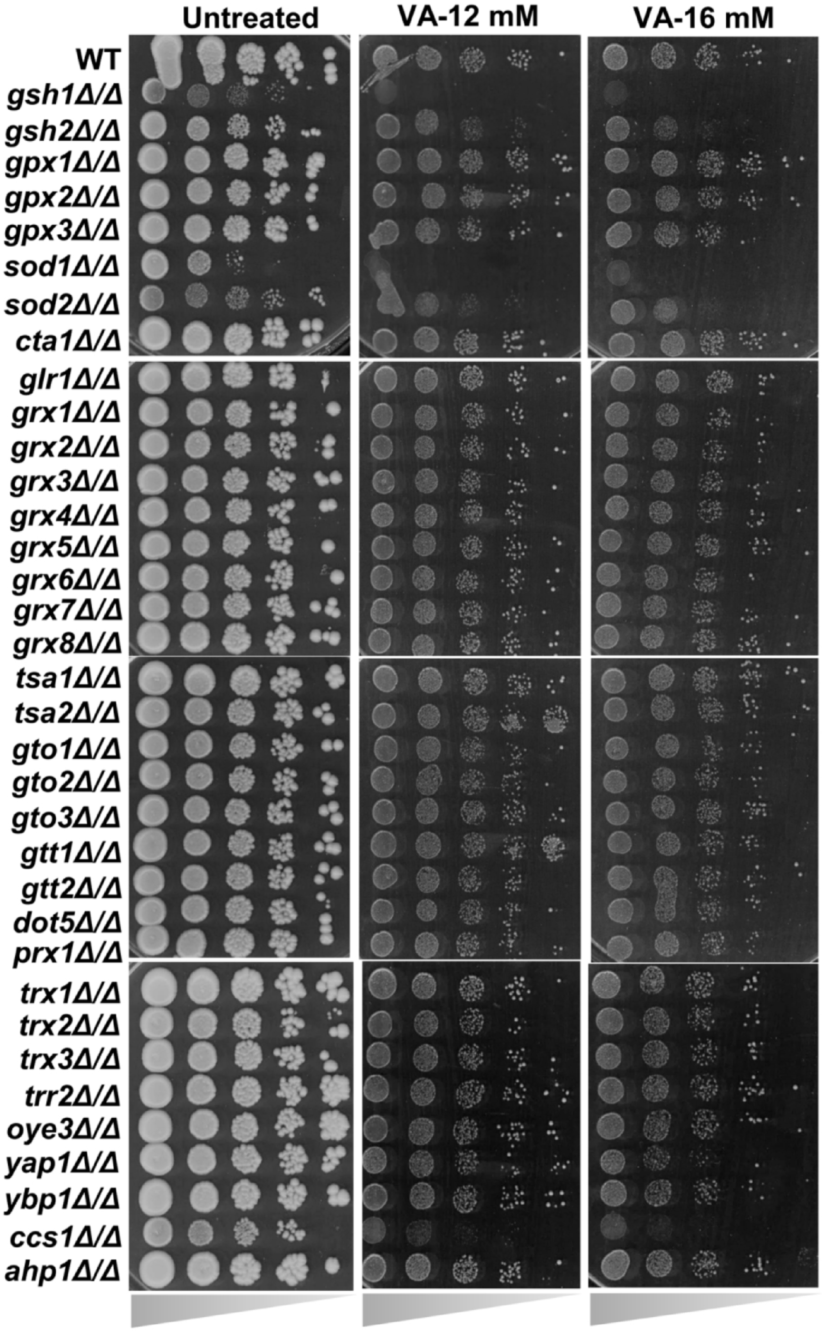

Figure S8: Screening of antioxidant mutants for tolerance against Valproic acid. 3 µL of ten-fold serially diluted cultures of antioxidant mutants was spotted onto SC-Agar plates supplemented without or with VA (12 and 16 mM). The plates were imaged after 72 h incubation at 30 ^{o}C.

Figure S9:

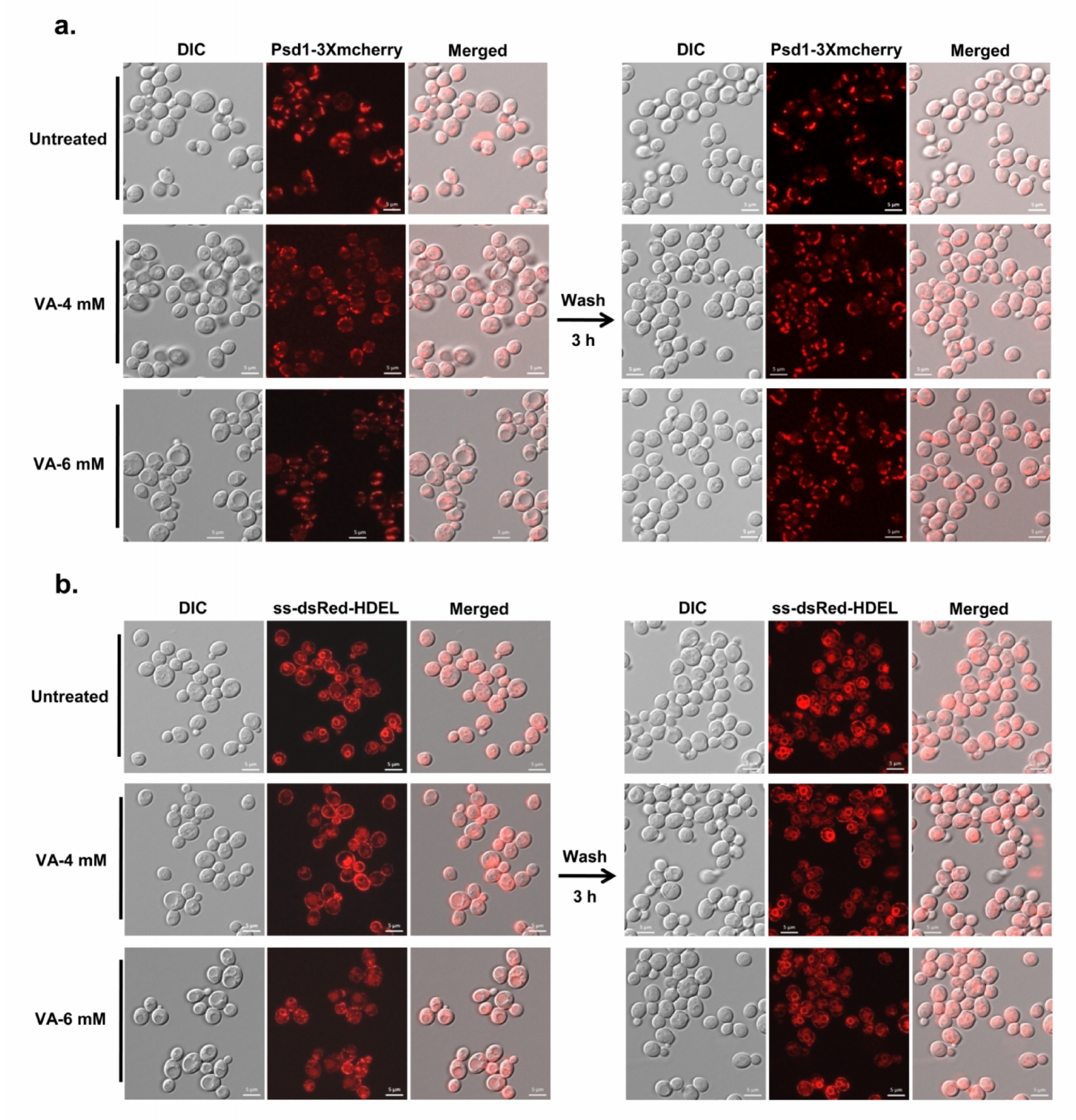

Figure S9: Acute exposure to Valproic acid induces reversible reorganization of mitochondria and ER. The wild-type cells harbouring a mitochondrial resident Psd1-3Xmcherry (**a**) or ER marker ss-dsRed-HDEL reporter (**b**) were grown to exponential phase and then left untreated or treated with VA (4 and 6 mM) for 3 h. All the cells were washed and resuspended at an equal OD600 of 0.8 in fresh SC liquid media and allowed to grow at 30 ^{o}C for 3 h. The cells were visualized under Apotome microscope using 'mCherry' filter just after 3 h VA treatment and removal of VA from growth media. Scale bar represents 5 µm.

Figure S10:

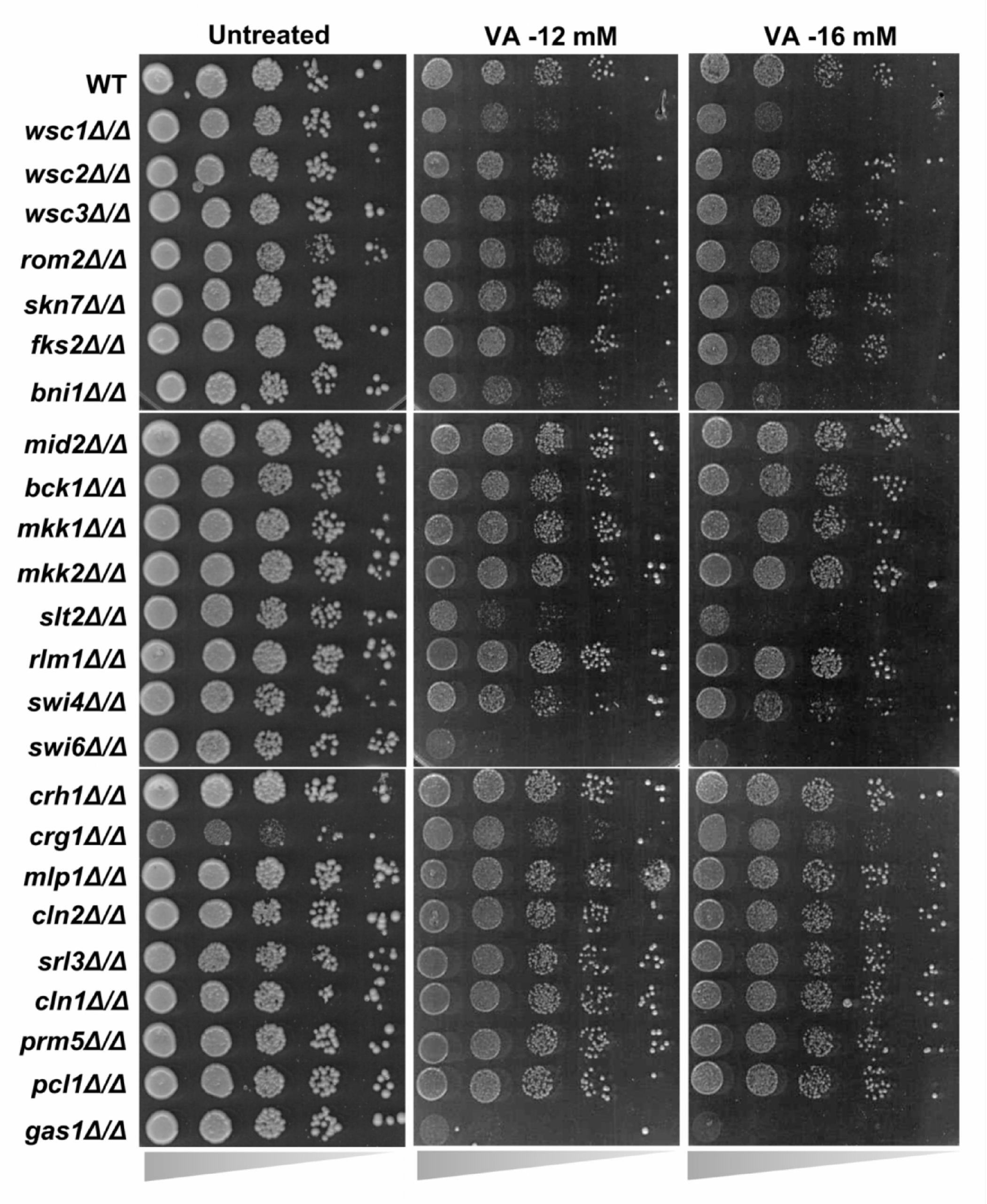

Figure S10: Intact Cell Wall Integrity (CWI) pathway required for Valproic acid tolerance. 3 μL of ten-fold serially diluted cultures of CWI pathway mutants was spotted onto SC-Agar plates supplemented without or with VA (12 and 16 mM). The plates were imaged after 72 h incubation at 30 °C.

SUPPLEMENTARY TABLES:

Table S1: List of yeast strains used in this study

No.	Strain Name	Genotype	Mutation	Source/Lab (Reference)
1	W15884C	*MATa ade2-1 can1-100 his3-11,15 leu2-3, 112 trp1-1 ura3-1 RAD5+*	WT	Andrei Chabes[10]
2	TOY510	*MATa dun1Δ::KanMX6*	*dun1Δ*	Andrei Chabes
3	TOY588	*MATa ade3::HISG dun1Δ::KanMX6 sml1Δ::HIS3*	*dun1Δ, sml1Δ*	Andrei Chabes
4	TOY603	*MATa ade3::HISG ixr1Δ::TRP1*	*ixr1Δ*	Andrei Chabes
5	TOY711	*MATa mec1Δ::TRP1 sml1Δ::HIS3*	*mec1Δ, sml1Δ*	Andrei Chabes
6	TOY722	*MATa sml1Δ::HIS3*	*sml1Δ*	Andrei Chabes
7	TOY774	*MATa dun1Δ::ura3Δ::LEU2 mec1Δ::TRP1 sml1Δ::HIS3 RNR3-Ha::KanMX6*	*dun1Δ, mec1Δ sml1Δ,* RNR3-Ha	Andrei Chabes
8	TOY782	*MATa rad53Δ::HphMX4 sml1Δ::HIS3 RNR3-Ha::KanMX6*	*rad53Δ, sml1Δ,* RNR3-Ha	Andrei Chabes
9	EY0690	*MATa his3-11,15; leu2-3,112; ura3-1; trp1-1; can1-100 ADE2 GAL+ psi+*	WT	Erin K. O'Shea[11]
10	EY2335	Isogenic to EY0690, *Hog1-GFP::His Nhp6a-RFP::KanMX6*	*Hog1-GFP, Nhp6a-RFP*	Erin K. O'Shea
11	EY2318	Isogenic to EY0690, *hog1::URA MATa*	*hog1Δ*	Erin K. O'Shea
12	EY2296	Isogenic to EY0690, *hot1::HIS3 MATa*	*hot1Δ*	Erin K. O'Shea
13	EY2308	Isogenic to EY0690, *sko1::LEU2 MATa*	*sko1Δ*	Erin K. O'Shea
14	EY2293	Isogenic to EY0690, *msn2::LEU2 msn4::HIS3 MATa*	*msn2Δ, msn4Δ*	Erin K. O'Shea
15	EY2322	Isogenic to EY0690, *hog1::URA msn2::LEU2 msn4::HIS3 MATa*	*hog1Δ, msn2Δ, msn4Δ*	Erin K. O'Shea
16	EY2328	Isogenic to EY0690, *sko1::URA hot1::G418 hog1::URA msn2::LEU2 msn4::HIS3 MATa*	*sko1Δ, hot1Δ, hog1Δ, msn2Δ, msn4Δ*	Erin K. O'Shea
17	BY4742	*MATα his3Δ1;leu2Δ0;lys2Δ0;ura3Δ0*	WT	Axel Mogk[12]
18	GSHY583	Isogenic to BY4742; *ss-dsRed-HDEL::natNT1*	*ss-dsRed-HDEL*	Axel Mogk
19	GSHY619	Isogenic to BY4742; *Fas2-GFP::KanMX4;Psd1-3Xmcherry::hphNT1*	*Psd1-mcherry, Fas2-GFP*	Axel Mogk
20	BY4741	*MATa his3Δ0;leu2Δ0;met15Δ0;ura3Δ0*	Wild-type (WT)	Axel Mogk
21	W303-1A	*MATa ade2-1; his3-11,15; leu2-3,112; ura3-1; trp1-1; can1-100*	Wild-type (WT)	David Shore[13]
22	SCY62	*MAT*a *his3-11,15; leu2-3,112; ura3-1; trp1-1; can1-100 ADE2*	Wild-type (WT)	Sten Stymne[14]
23	H1246	*MATα are1Δ::HIS3 are2Δ::LEU2 dga1Δ::KanMX4 lro1Δ::TRP1 ADE2*	*are1Δare2Δdga1Δlro1Δ*	Sten Stymne
24	BY4743	*MATa/α his3Δ1/his3Δ1 leu2Δ0/leu2Δ0 LYS2/lys2Δ0 met15Δ0/MET15 ura3Δ0/ura3Δ0*	Wild-type (WT)	Yeast Knockout Collection-Open Biosystems(YKO-OB)
25	*hac1Δ/hac1Δ*	Isogenic to BY4743; *hac1Δ::KanMX4*	*hac1Δ*	YKO-OB
26	*ire1Δ/ire1Δ*	Isogenic to BY4743; *ire1Δ:: KanMX4*	*ire1Δ*	YKO-OB
27	*gsh1Δ/gsh1Δ*	Isogenic to BY4743; *gsh1Δ:: KanMX4*	*gsh1Δ*	YKO-OB
28	*gsh2Δ/gsh2Δ*	Isogenic to BY4743; *gsh2Δ:: KanMX4*	*gsh2Δ*	YKO-OB
29	*gpx1Δ/gpx1Δ*	Isogenic to BY4743; *gpx1Δ*:: *KanMX4*	*gpx1Δ*	YKO-OB
30	*gpx2Δ/gpx2Δ*	Isogenic to BY4743; *gpx2Δ*:: *KanMX4*	*gpx2Δ*	YKO-OB
31	*gpx3Δ/gpx3Δ*	Isogenic to BY4743; *gpx3Δ*:: *KanMX4*	*gpx3Δ*	YKO-OB
32	*sod1Δ/sod1Δ*	Isogenic to BY4743; *sod1Δ*:: *KanMX4*	*sod1Δ*	YKO-OB
33	*sod2Δ/sod2Δ*	Isogenic to BY4743; *sod2Δ*:: *KanMX4*	*sod2Δ*	YKO-OB
34	*cta1Δ/cta1Δ*	Isogenic to BY4743; *cta1Δ*:: *KanMX4*	*cta1Δ*	YKO-OB
35	*glr1Δ/glr1Δ*	Isogenic to BY4743; *glr1Δ*:: *KanMX4*	*glr1Δ*	YKO-OB
36	*grx1Δ/grx1Δ*	Isogenic to BY4743; *grx1Δ*:: *KanMX4*	*grx1Δ*	YKO-OB
37	*grx2Δ/grx2Δ*	Isogenic to BY4743; *grx2Δ*:: *KanMX4*	*grx2Δ*	YKO-OB
38	*grx3Δ/grx3Δ*	Isogenic to BY4743; *grx3Δ*:: *KanMX4*	*grx3Δ*	YKO-OB
39	*grx4Δ/grx4Δ*	Isogenic to BY4743; *grx4Δ*:: *KanMX4*	*grx4Δ*	YKO-OB
40	*grx5Δ/grx5Δ*	Isogenic to BY4743; *grx5Δ*:: *KanMX4*	*grx5Δ*	YKO-OB
41	*grx6Δ/grx6Δ*	Isogenic to BY4743; *grx6Δ*:: *KanMX4*	*grx6Δ*	YKO-OB
42	*grx7Δ/grx7Δ*	Isogenic to BY4743; *grx7Δ*:: *KanMX4*	*grx7Δ*	YKO-OB
43	*grx8Δ/grx8Δ*	Isogenic to BY4743; *grx8Δ*:: *KanMX4*	*grx8Δ*	YKO-OB
44	*tsa1Δ/tsa1Δ*	Isogenic to BY4743; *tsa1Δ*:: *KanMX4*	*tsa1Δ*	YKO-OB
45	*tsa2Δ/tsa2Δ*	Isogenic to BY4743; *tsa2Δ*:: *KanMX4*	*tsa2Δ*	YKO-OB
46	*gto1Δ/gto1Δ*	Isogenic to BY4743; *gto1Δ*:: *KanMX4*	*gto1Δ*	YKO-OB
47	*gto2Δ/gto2Δ*	Isogenic to BY4743; *gto2Δ*:: *KanMX4*	*gto2Δ*	YKO-OB

48	*gto3Δ/gto3Δ*	Isogenic to BY4743; *gto3Δ*:: *KanMX4*	*gto3Δ*	YKO-OB
49	*gtt1Δ/gtt1Δ*	Isogenic to BY4743; *gtt1Δ*:: *KanMX4*	*gtt1Δ*	YKO-OB
50	*gtt2Δ/gtt2Δ*	Isogenic to BY4743; *gtt2Δ*:: *KanMX4*	*gtt2Δ*	YKO-OB
51	*dot5Δ/dot5Δ*	Isogenic to BY4743; *dot5Δ*:: *KanMX4*	*dot5Δ*	YKO-OB
52	*prx1Δ/prx1Δ*	Isogenic to BY4743; *prx1Δ*:: *KanMX4*	*prx1Δ*	YKO-OB
53	*trx1Δ/trx1Δ*	Isogenic to BY4743; *trx1Δ*:: *KanMX4*	*trx1Δ*	YKO-OB
54	*trx2Δ/trx2Δ*	Isogenic to BY4743; *trx2Δ*:: *KanMX4*	*trx2Δ*	YKO-OB
55	*trx3Δ/trx3Δ*	Isogenic to BY4743; *trx3Δ*:: *KanMX4*	*trx3Δ*	YKO-OB
56	*trr2Δ/trr2Δ*	Isogenic to BY4743; *trr2Δ*:: *KanMX4*	*trr2Δ*	YKO-OB
57	*oye3Δ/oye3Δ*	Isogenic to BY4743; *oye3Δ*:: *KanMX4*	*oye3Δ*	YKO-OB
58	*yap1Δ/yap1Δ*	Isogenic to BY4743; *yap1Δ*:: *KanMX4*	*yap1Δ*	YKO-OB
59	*ybp1Δ/ybp1Δ*	Isogenic to BY4743; *ybp1Δ*:: *KanMX4*	*ybp1Δ*	YKO-OB
60	*ccs1Δ/ccs1Δ*	Isogenic to BY4743; *ccs1Δ*:: *KanMX4*	*ccs1Δ*	YKO-OB
61	*ahp1Δ/ahp1Δ*	Isogenic to BY4743; *ahp1Δ*:: *KanMX4*	*ahp1Δ*	YKO-OB
62	*ssk1Δ/ssk1Δ*	Isogenic to BY4743; *ssk1Δ*:: *KanMX4*	*ssk1Δ*	YKO-OB
63	*ssk2Δ/ssk2Δ*	Isogenic to BY4743; *ssk2Δ*:: *KanMX4*	*ssk2Δ*	YKO-OB
64	*ssk22Δ/ssk22Δ*	Isogenic to BY4743; *ssk22Δ*:: *KanMX4*	*ssk22Δ*	YKO-OB
65	*msb2Δ/msb2Δ*	Isogenic to BY4743; *msb2Δ*:: *KanMX4*	*msb2Δ*	YKO-OB
66	*sho1Δ/sho1Δ*	Isogenic to BY4743; *sho1Δ*:: *KanMX4*	*sho1Δ*	YKO-OB
67	*ptc1Δ/ptc1Δ*	Isogenic to BY4743; *ptc1Δ*:: *KanMX4*	*ptc1Δ*	YKO-OB
68	*ptc2Δ/ptc2Δ*	Isogenic to BY4743; *ptc2Δ*:: *KanMX4*	*ptc2Δ*	YKO-OB
69	*ptc3Δ/ptc3Δ*	Isogenic to BY4743; *ptc3Δ*:: *KanMX4*	*ptc3Δ*	YKO-OB
70	*hog1Δ/hog1Δ*	Isogenic to BY4743; *hog1Δ*:: *KanMX4*	*hog1Δ*	YKO-OB
71	*pbs2Δ/pbs2Δ*	Isogenic to BY4743; *pbs2Δ*:: *KanMX4*	*pbs2Δ*	YKO-OB
72	*ste50Δ/ste50Δ*	Isogenic to BY4743; *ste50Δ*:: *KanMX4*	*ste50Δ*	YKO-OB
73	*wsc1Δ/wsc1Δ*	Isogenic to BY4743; *wsc1Δ*:: *KanMX4*	*wsc1Δ*	YKO-OB
74	*wsc2Δ/wsc2Δ*	Isogenic to BY4743; *wsc2Δ*:: *KanMX4*	*wsc2Δ*	YKO-OB
75	*wsc3Δ/wsc3Δ*	Isogenic to BY4743; *wsc3Δ*:: *KanMX4*	*wsc3Δ*	YKO-OB
76	*rom2Δ/rom2Δ*	Isogenic to BY4743; *rom2Δ*:: *KanMX4*	*rom2Δ*	YKO-OB
77	*skn7Δ/skn7Δ*	Isogenic to BY4743; *skn7Δ*:: *KanMX4*	*skn7Δ*	YKO-OB
78	*fks2Δ/fks2Δ*	Isogenic to BY4743; *fks2Δ*:: *KanMX4*	*fks2Δ*	YKO-OB
79	*bni1Δ/bni1Δ*	Isogenic to BY4743; *bni1Δ*:: *KanMX4*	*bni1Δ*	YKO-OB
80	*mid2Δ/mid2Δ*	Isogenic to BY4743; *mid2Δ*:: *KanMX4*	*mid2Δ*	YKO-OB
81	*bck1Δ/bck1Δ*	Isogenic to BY4743; *bck1Δ*:: *KanMX4*	*bck1Δ*	YKO-OB
82	*mkk1Δ/mkk1Δ*	Isogenic to BY4743; *mkk1Δ*:: *KanMX4*	*mkk1Δ*	YKO-OB
83	*mkk2Δ/mkk2Δ*	Isogenic to BY4743; *mkk2Δ*:: *KanMX4*	*mkk2Δ*	YKO-OB
84	*slt2Δ/slt2Δ*	Isogenic to BY4743; *slt2Δ*:: *KanMX4*	*slt2Δ*	YKO-OB
85	*rlm1Δ/rlm1Δ*	Isogenic to BY4743; *rlm1Δ*:: *KanMX4*	*rlm1Δ*	YKO-OB
86	*swi4Δ/swi4Δ*	Isogenic to BY4743; *swi4Δ*:: *KanMX4*	*swi4Δ*	YKO-OB
87	*swi6Δ/swi6Δ*	Isogenic to BY4743; *swi6Δ*:: *KanMX4*	*swi6Δ*	YKO-OB
88	*crh1Δ/chr1Δ*	Isogenic to BY4743; *chr1Δ*:: *KanMX4*	*crh1Δ*	YKO-OB
89	*crg1Δ/crg1Δ*	Isogenic to BY4743; *crg1Δ*:: *KanMX4*	*crg1Δ*	YKO-OB
90	*mlp1Δ/mlp1Δ*	Isogenic to BY4743; *mlp1Δ*:: *KanMX4*	*mlp1Δ*	YKO-OB
91	*cln1Δ/cln1Δ*	Isogenic to BY4743; *cln1Δ*:: *KanMX4*	*cln1Δ*	YKO-OB
92	*cln2Δ/cln2Δ*	Isogenic to BY4743; *cln2Δ*:: *KanMX4*	*cln2Δ*	YKO-OB
93	*srl3Δ/srl3Δ*	Isogenic to BY4743; *srl3Δ*:: *KanMX4*	*srl3Δ*	YKO-OB
94	*prm5Δ/prm5Δ*	Isogenic to BY4743; *prm5Δ*:: *KanMX4*	*prm5Δ*	YKO-OB
95	*pcl1Δ/pcl1Δ*	Isogenic to BY4743; *pcl1Δ*:: *KanMX4*	*pcl1Δ*	YKO-OB
96	*gas1Δ/gas1Δ*	Isogenic to BY4743; *gas1Δ*:: *KanMX4*	*gas1Δ*	YKO-OB
97	*fet3Δ/fet3Δ*	Isogenic to BY4743; *fet3Δ*:: *KanMX4*	*fet3Δ*	YKO-OB
98	*sac1Δ/sac1Δ*	Isogenic to BY4743; *sac1Δ*:: *KanMX4*	*sac1Δ*	YKO-OB
99	*gef1Δ/gef1Δ*	Isogenic to BY4743; *gef1Δ*:: *KanMX4*	*gef1Δ*	YKO-OB
100	*ysp1Δ/ysp1Δ*	Isogenic to BY4743; *ysp1Δ*:: *KanMX4*	*ysp1Δ*	YKO-OB
101	*ccc2Δ/ccc2Δ*	Isogenic to BY4743; *ccc2Δ*:: *KanMX4*	*ccc2Δ*	YKO-OB
102	*erg3Δ/erg3Δ*	Isogenic to BY4743; *erg3Δ*:: *KanMX4*	*erg3Δ*	YKO-OB
103	*atx1Δ/atx1Δ*	Isogenic to BY4743; *atx1Δ*:: *KanMX4*	*atx1Δ*	YKO-OB
104	*ftr1Δ/ftr1Δ*	Isogenic to BY4743; *ftr1Δ*:: *KanMX4*	*ftr1Δ*	YKO-OB
105	*ctr1Δ/ctr1Δ*	Isogenic to BY4743; *ctr1Δ*:: *KanMX4*	*ctr1Δ*	YKO-OB
106	*mac1Δ/mac1Δ*	Isogenic to BY4743; *mac1Δ*:: *KanMX4*	*mac1Δ*	YKO-OB
107	*lem3Δ/lem3Δ*	Isogenic to BY4743; *lem3Δ*:: *KanMX4*	*lem3Δ*	YKO-OB
108	*cup5Δ/cup5Δ*	Isogenic to BY4743; *cup5Δ*:: *KanMX4*	*cup5Δ*	YKO-OB
109	*gal11Δ/gal11Δ*	Isogenic to BY4743; *gal11Δ*:: *KanMX4*	*gal11Δ*	YKO-OB
110	*erv14Δ/erv14Δ*	Isogenic to BY4743; *erv14Δ*:: *KanMX4*	*erv14Δ*	YKO-OB
111	*aft1Δ/aft1Δ*	Isogenic to BY4743; *aft1Δ*:: *KanMX4*	*aft1Δ*	YKO-OB

112	*mrs4Δ/mrs4Δ*	Isogenic to BY4743; *mrs4Δ*:: *KanMX4*	*mrs4Δ*	YKO-OB
113	*vma11Δ/vma11Δ*	Isogenic to BY4743; *vma11Δ*:: *KanMX4*	*vma11Δ*	YKO-OB
114	*fre3Δ/fre3Δ*	Isogenic to BY4743; *fre3Δ*:: *KanMX4*	*fre3Δ*	YKO-OB
115	*fre4Δ/fre4Δ*	Isogenic to BY4743; *fre4Δ*:: *KanMX4*	*fre4Δ*	YKO-OB
116	*fre5Δ/fre5Δ*	Isogenic to BY4743; *fre5Δ*:: *KanMX4*	*fre5Δ*	YKO-OB
117	*fre6Δ/fre6Δ*	Isogenic to BY4743; *fre6Δ*:: *KanMX4*	*fre6Δ*	YKO-OB
118	*fre7Δ/fre7Δ*	Isogenic to BY4743; *fre7Δ*:: *KanMX4*	*fre7Δ*	YKO-OB
119	*pex12Δ/pex12Δ*	Isogenic to BY4743; *pex12Δ*:: *KanMX4*	*pex12Δ*	YKO-OB
120	*usa1Δ/usa1Δ*	Isogenic to BY4743; *usa1Δ*:: *KanMX4*	*usa1Δ*	YKO-OB
121	*rog3Δ/rog3Δ*	Isogenic to BY4743; *rog3Δ*:: *KanMX4*	*rog3Δ*	YKO-OB
122	*vps36Δ/vps36Δ*	Isogenic to BY4743; *vps36Δ*:: *KanMX4*	*vps36Δ*	YKO-OB

Table S2: List of gene specific primers used in this study

S.No.	Gene	Primer Sequence (5'→ 3')
1	ACT1	**F:** CCTTCTGTTTTGGGTTTGGA
		**R:** CGGTGATTTCCTTTTGCATT
2	DIT1	**F:** TGACGCCATTGGCTTTTGTG
		**R:** TGCTTTCTCAAACGACCTGC
3	DIT2	**F:** ATTAGCGCGTATGGTGCAGT
		**R:** TCCAAGGGCAACCTGTGAAA
4	DMC1	**F:** TGCGGTAGAGGTGAACTAAGC
		**R:** TCCTTGTTGCTGACGCATGT
5	FET3	**F:** TGGTCACGGACTTGACGAAG
		**R:** CACGGTCATTTCGTGGTCCT
6	FRE1	**F:** GGGTCTCGTCTTCTTCTGGG
		**R:** GACCCTTGCCTCGAGTTGTA
7	GIT1	**F:** ATCGGTTCTGTAGTAGGCG
		**R:** TTACCAGTCCAGCCATTGG
8	HOG1	**F:** GGATGCCTTGGCTCATCCTT
		**R:** TGGTCATCAAACGTGGCAGA
9	SPO13	**F:** ACAAACAATAGAGCGCCCCT
		**R:** TCAGGAACGTTGGGTTTCCA
10	SPO20	**F:** TCGCCACCATAATCAGCACC
		**R:** CGAACCGACAGTTTGGGTGA
11	SPS1	**F:** GGTCCTTGGGTATCACCACC
		**R:** AGTTATATGCCGATGGCCGA
12	SPS100	**F:** TTGGTTGTTGTCAGCTCTGGT
		**R:** TTTGCGCTTGGATTGGTGAC
13	WSC3	**F:** TCCAGTCTGCTGGACTGTCT
		**R:** AGGCCAGCCAGAGCATTTAG
14	YLR194C	**F:** GCCGCTGCTCAAAAAGACTC
		**R:** GGAGCCACAGCATAGGTGTT
15	ACT1 (RT)	**F:** CACCCTGTTCTTTTGACTGAAGC
		**R:** TACCGGCAGATTCCAAACCC
16	HAC1-S (RT)	**F:** TAGAGGGATTTCCAGAGCACG
		**R:** TCATTGAAGTGATGAAGAAATC

'**F**' denotes 'Forward' whereas '**R**' denotes 'Reverse' primer. '**RT**' denotes the set of primers used in semi-quantitative Reverse transcription-PCR (RT-PCR).

Table S3: Complete list of differentially expressed genes (fold-change≥2, moderated t-test BH-FDR corrected p≤0.05) in *Saccharomyces cerevisiae* (1588-4C) cells upon Valproic acid (6 mM) treatment for 3 h relative to untreated (control) cells.

Probe Set ID	Gene Symbol	Systematic Name	Gene Title	Fold-change (Treated / Untreated)	Log Fold-change	Regulation
1770587_at	OCD2	YLR307C-A		141.8389	7.148109	up
1770513_at	SRL4	YPL033C	Protein of unknown function; involved in regulation of dNTP production; null mutant suppresses the lethality of lcd1 and rad53 mutations;	135.0563	7.077417	up
1779413_at	MET2	YNL277W	L-homoserine-O-acetyltransferase, catalyzes the conversion of homoserine to O-acetyl homoserine which is the first step of the methionine biosynthetic pathway	70.89468	6.147605	up
1775364_at	HSP30	YCR021C	Hydrophobic plasma membrane localized, stress-responsive protein that negatively regulates the H(+)-ATPase Pma1p; induced by heat shock, ethanol treatment, weak organic acid, glucose limitation, and entry into stationary phase	36.9923	5.209153	up
1769750_at	MET32	YDR253C	Zinc-finger DNA-binding protein, involved in transcriptional regulation of the methionine biosynthetic genes, similar to Met31p	36.95904	5.207855	up
1777707_at	FMP23	YBR047W	Putative protein of unknown function; proposed to be involved in iron or copper homeostasis; the authentic, non-tagged protein is detected in highly purified mitochondria in high-throughput studies	34.71117	5.117328	up
1771460_at	RSB1	YOR049C	Suppressor of sphingoid long chain base (LCB) sensitivity of an LCB-lyase mutation; putative integral membrane transporter or flippase that may transport LCBs from the cytoplasmic side toward the extracytoplasmic side of the membrane	34.25942	5.098429	up
1769998_at		YLR346C		28.35421	4.825491	up
1780106_at		YAL018C		25.13218	4.651464	up
1778735_at		YGR035C		25.11883	4.650697	up
1778706_at	ATO2	YNR002C	Putative transmembrane protein involved in export of ammonia, a starvation signal that promotes cell death in aging colonies; phosphorylated in mitochondria; member of the TC 9.B.33 YaaH family; homolog of Ady2p and Y. lipolytica Gpr1p	23.88405	4.577976	up
1776304_at	CTA1	YDR256C	Catalase A, breaks down hydrogen peroxide in the peroxisomal matrix formed by acyl-CoA oxidase (Pox1p) during fatty acid beta-oxidation	21.6436	4.435868	up
1771165_x_at	HXT13	YEL069C	Hexose transporter, induced in the presence of non-fermentable carbon sources, induced by low levels of glucose, repressed by high levels of glucose	21.08122	4.397886	up
1771695_at	CIT3	YPR001W	Dual specificity mitochondrial citrate and methylcitrate synthase; catalyzes the condensation of acetyl-CoA and oxaloacetate to form citrate and that of propionyl-CoA and oxaloacetate to form 2-methylcitrate	19.93721	4.317391	up
1773748_at	MUP3	YHL036W	Low affinity methionine permease, similar to Mup1p	19.69616	4.299842	up
1777878_at	SPR28	YDR218C	Sporulation-specific homolog of the yeast CDC3/10/11/12 family of bud neck microfilament genes; meiotic septin expressed at high levels during meiotic divisions and ascospore formation	19.23296	4.265509	up
1773674_at		YGR273C		18.99485	4.247537	up
1779524_at	HXT11	YOL156W	Putative hexose transporter that is nearly identical to Hxt9p, has similarity to major facilitator	18.91695	4.241608	up

			superfamily (MFS) transporters and is involved in pleiotropic drug resistance			
1778079_at	BTN2	YGR142W	v-SNARE binding protein that facilitates specific protein retrieval from a late endosome to the Golgi; modulates arginine uptake, possible role in mediating pH homeostasis between the vacuole and plasma membrane H(+)-ATPase	18.51249	4.210427	up
1775628_at	GAL7	YBR018C	Galactose-1-phosphate uridyl transferase, synthesizes glucose-1-phosphate and UDP-galactose from UDP-D-glucose and alpha-D-galactose-1-phosphate in the second step of galactose catabolism	18.42949	4.203944	up
1770548_at		YMR279C		18.41727	4.202988	up
1777388_at	STR3	YGL184C	Peroxisomal cystathionine beta-lyase, converts cystathionine into homocysteine; may be redox regulated by Gto1p	18.10341	4.17819	up
1777513_at	SPO21	YOL091W	Component of the meiotic outer plaque of the spindle pole body, involved in modifying the meiotic outer plaque that is required prior to prospore membrane formation	18.02834	4.172195	up
1777122_at		YOL014W		17.70987	4.146482	up
1774778_at	CRC1	YOR100C	Mitochondrial inner membrane carnitine transporter, required for carnitine-dependent transport of acetyl-CoA from peroxisomes to mitochondria during fatty acid beta-oxidation	17.65045	4.141633	up
1776680_at	SPG1	YGR236C	Protein required for survival at high temperature during stationary phase; not required for growth on nonfermentable carbon sources; the authentic, non-tagged protein is detected in highly purified mitochondria in high-throughput studies	17.49224	4.128643	up
1772126_at	FIG1	YBR040W	Integral membrane protein required for efficient mating; may participate in or regulate the low affinity Ca2+ influx system, which affects intracellular signaling and cell-cell fusion during mating	17.04606	4.091366	up
1774863_at		YLR012C		16.97031	4.084941	up
1773970_s_at	HXT9	YJL219W	Putative hexose transporter that is nearly identical to Hxt9p, has similarity to major facilitator superfamily (MFS) transporters and is involved in pleiotropic drug resistance, expression of HXT9 is regulated by transcription factors Pdr1p and Pdr3p	15.91665	3.992465	up
1770897_x_at	PAU5	YFL020C	Member of the seripauperin multigene family encoded mainly in subtelomeric regions; induced during alcoholic fermentation; induced by low temperature and also by anaerobic conditions; negatively regulated by oxygen and repressed by heme	15.66697	3.969654	up
1778154_at	IRC18	YJL037W	Putative protein of unknown function; expression induced in respiratory-deficient cells and in carbon-limited chemostat cultures; similar to adjacent ORF, YJL038C; null mutant displays increased levels of spontaneous Rad52p foci	15.63058	3.9663	up
1773507_at		YOR214C		15.61436	3.964802	up
1778197_at	MND1	YGL183C	Protein required for recombination and meiotic nuclear division; forms a complex with Hop2p, which is involved in chromosome pairing and repair of meiotic double-strand breaks	15.37916	3.942905	up
1774858_at	PES4	YFR023W	Poly(A) binding protein, suppressor of DNA polymerase epsilon mutation, similar to Mip6p	15.07384	3.913975	up
1774240_at	SPR3	YGR059W	Sporulation-specific homolog of the yeast CDC3/10/11/12 family of bud neck microfilament genes; septin protein involved in sporulation; regulated by ABFI	14.80559	3.88807	up
1778218_at	TMA10	YLR327C	Protein of unknown function that associates with	14.54762	3.862711	up

			ribosomes; putative homolog of the F1F0-ATPase synthase regulator Stf2p			
1777095_at	RNP1	YLL046C	Ribonucleoprotein that contains two RNA recognition motifs (RRM)	14.50052	3.858033	up
1774777_at	NQM1	YGR043C	Transaldolase of unknown function; transcription is repressed by Mot1p and induced by α-factor & during diauxic shift	14.43586	3.851585	up
1776583_at	FIT2	YOR382W	Mannoprotein that is incorporated into the cell wall via a glycosylphosphatidylinositol (GPI) anchor, involved in the retention of siderophore-iron in the cell wall	14.38634	3.846628	up
1773670_at	TIR4	YOR009W	Cell wall mannoprotein of the Srp1p/Tip1p family of serine-alanine-rich proteins; expressed under anaerobic conditions and required for anaerobic growth; transcription is also induced by cold shock	14.32154	3.840115	up
1778804_at	TIR1	YER011W	Cell wall mannoprotein of the Srp1p/Tip1p family of serine-alanine-rich proteins; expression is downregulated at acidic pH and induced by cold shock and anaerobiosis; abundance is increased in cells cultured without shaking	13.998	3.807149	up
1771373_at	SPS100	YHR139C	Protein required for spore wall maturation; expressed during sporulation; may be a component of the spore wall; expression also induced in cells treated with the mycotoxin patulin	13.89735	3.796738	up
1771645_at	SUE1	YPR151C	Mitochondrial protein required for degradation of unstable forms of cytochrome c	13.83355	3.7901	up
1780163_at	MET28	YIR017C	Basic leucine zipper (bZIP) transcriptional activator in the Cbf1p-Met4p-Met28p complex, participates in the regulation of sulfur metabolism	13.57923	3.76333	up
1777667_s_at	DSF1	YEL070W	Deletion suppressor of mpt5 mutation	12.89941	3.689234	up
1771091_at	DAN1	YJR150C	Cell wall mannoprotein with similarity to Tir1p, Tir2p, Tir3p, and Tir4p; expressed under anaerobic conditions, completely repressed during aerobic growth	12.86931	3.685863	up
1777222_at	YSW1	YBR148W	Protein required for normal prospore membrane formation; interacts with Gip1p, which is the meiosis-specific regulatory subunit of the Glc7p protein phosphatase; expressed specifically in spores and localizes to the prospore membrane	12.62159	3.657821	up
1780089_at	MAM1	YER106W	Monopolin, kinetochore associated protein involved in chromosome attachment to meiotic spindle	12.51825	3.645961	up
1772536_at	ECM34	YHL043W	Putative protein of unknown function; member of the DUP380 subfamily of conserved, often subtelomerically-encoded proteins	12.51389	3.645459	up
1778374_at		YBL086C		11.9101	3.574114	up
1772511_at	SNZ1	YMR096W	Protein involved in vitamin B6 biosynthesis; member of a stationary phase-induced gene family; coregulated with SNO1; interacts with Sno1p and with Yhr198p, perhaps as a multiprotein complex containing other Snz and Sno proteins	11.86926	3.569159	up
1778649_at	SPO74	YGL170C	Component of the meiotic outer plaque of the spindle pole body, involved in modifying the meiotic outer plaque that is required prior to prospore membrane formation	11.84725	3.566481	up
1779091_at		YLR031W		11.02049	3.462117	up
1775215_at		YJL213W		10.95338	3.453305	up
1769531_at		YGL138C		10.8746	3.442891	up
1773109_at	DAL80	YKR034W	Negative regulator of genes in multiple nitrogen degradation pathways; expression is regulated by nitrogen levels and by Gln3p; member of the GATA-binding family, forms homodimers and heterodimers with Deh1p	10.62492	3.40938	up

1777561_at	FMP43	YGR243W	Highly conserved subunit of the mitochondrial pyruvate carrier; a mitochondrial inner membrane complex comprised of Fmp37p/Mpc1p and either Mpc2p or Fmp43p/Mpc3p mediates mitochondrial pyruvate uptake; more highly expressed in glucose-containing minimal medium than in lactate-containing medium; expression regulated by osmotic and alkaline stresses	10.57517	3.402609	up
1774855_at	SPS2	YDR522C	Protein expressed during sporulation, redundant with Sps22p for organization of the beta-glucan layer of the spore wall; S. pombe ortholog is a spore wall component	10.51076	3.393795	up
1770437_at		YPR015C		10.4611	3.386962	up
1772358_at		YKL070W		10.45984	3.386788	up
1774829_at	TIR3	YIL011W	Cell wall mannoprotein of the Srp1p/Tip1p family of serine-alanine-rich proteins; expressed under anaerobic conditions and required for anaerobic growth	10.43597	3.383492	up
1772214_at		YGL015C		10.38576	3.376535	up
1771274_at	MEK1	YOR351C	Meiosis-specific serine/threonine protein kinase, functions in meiotic checkpoint, promotes recombination between homologous chromosomes by suppressing double strand break repair between sister chromatids	10.33239	3.369102	up
1772880_at	SNO1	YMR095C	Protein of unconfirmed function, involved in pyridoxine metabolism; expression is induced during stationary phase; forms a putative glutamine amidotransferase complex with Snz1p, with Sno1p serving as the glutaminase	10.25187	3.357815	up
1776813_at	REC8	YPR007C	Meiosis-specific component of sister chromatid cohesion complex; maintains cohesion between sister chromatids during meiosis I; maintains cohesion between centromeres of sister chromatids until meiosis II; homolog of S. pombe Rec8p	10.16504	3.345544	up
1779924_at		YER053C-A		10.04841	3.328895	up
1778644_at	SPO20	YMR017W	Meiosis-specific subunit of the t-SNARE complex, required for prospore membrane formation during sporulation; similar to but not functionally redundant with Sec9p; SNAP-25 homolog	9.99191	3.320761	up
1773315_at	AAD6	YFL056C	Putative aryl-alcohol dehydrogenase; involved in oxidative stress response; similar to P. chrysosporium aryl-alcohol dehydrogenase; expression induced in cells treated with the mycotoxin patulin	9.966057	3.317023	up
1774960_at	NCA3	YJL116C	Protein that functions with Nca2p to regulate mitochondrial expression of subunits 6 (Atp6p) and 8 (Atp8p) of the Fo-F1 ATP synthase; member of the SUN family; expression induced in cells treated with the mycotoxin patulin	9.886726	3.305493	up
1774988_at	DIT2	YDR402C	N-formyltyrosine oxidase, sporulation-specific microsomal enzyme involved in the production of N,N-bisformyl dityrosine required for spore wall maturation, homologous to cytochrome P-450s	9.863262	3.302065	up
1774137_at	CYC7	YEL039C	Cytochrome c isoform 2, expressed under hypoxic conditions; electron carrier of the mitochondrial intermembrane space that transfers electrons from ubiquinone-cytochrome c oxidoreductase to cytochrome c oxidase during cellular respiration	9.693608	3.277034	up
1778622_at	UGA4	YDL210W	Permease that serves as a gamma-aminobutyrate (GABA) transport protein involved in the utilization of GABA as a nitrogen source; catalyzes the transport of putrescine and delta-aminolevulinic acid (ALA); localized to the vacuolar membrane	9.664695	3.272724	up
1775444_at	MPC54	YOR177C	Component of the meiotic outer plaque, a	9.572617	3.258913	up

			membrane-organizing center which is assembled on the cytoplasmic face of the spindle pole body during meiosis II and triggers the formation of the prospore membrane; potential Cdc28p substrate			
1771806_at		YDR374C		9.348806	3.224782	up
1778358_at	HES1	YOR237W	Protein implicated in the regulation of ergosterol biosynthesis; one of a seven member gene family with a common essential function and non-essential unique functions; similar to human oxysterol binding protein (OSBP)	9.347069	3.224514	up
1775331_at	OYE3	YPL171C	Conserved NADPH oxidoreductase containing flavin mononucleotide (FMN), homologous to Oye2p with different ligand binding and catalytic properties; has potential roles in oxidative stress response and programmed cell death	9.319854	3.220307	up
1770162_x_at	PAU24	YBR301W	Cell wall mannoprotein with similarity to Tir1p, Tir2p, Tir3p, and Tir4p; member of the seripauperin multigene family encoded mainly in subtelomeric regions; expressed under anaerobic conditions, completely repressed during aerobic growth	9.282548	3.214521	up
1776064_at	JEN1	YKL217W	Monocarboxylate/proton symporter of the plasma membrane; transport activity is dependent on the pH gradient across the membrane; mediates high-affinity uptake of carbon sources lactate, pyuvate, and acetate, and also of the micronutrient selenite, whose structure mimics that of monocarboxylates; expression and localization are tightly regulated, with transcription repression, mRNA degradation, and protein endocytosis and degradation all occurring in the presence of glucose	9.126696	3.190093	up
1772874_at	TIR2	YOR010C	Putative cell wall mannoprotein of the Srp1p/Tip1p family of serine-alanine-rich proteins; transcription is induced by cold shock and anaerobiosis	9.113366	3.187984	up
1774699_at	PRR2	YDL214C	Serine/threonine protein kinase that inhibits pheromone induced signalling downstream of MAPK, possibly at the level of the Ste12p transcription factor; mutant has increased aneuploidy tolerance	9.102143	3.186206	up
1776156_at	BDH2	YAL061W	Putative medium-chain alcohol dehydrogenase with similarity to BDH1; transcription induced by constitutively active PDR1 and PDR3	9.073404	3.181644	up
1770886_at		YLR030W		8.990564	3.168412	up
1769854_at	CRF1	YDR223W	Transcriptional corepressor involved in repression of ribosomal protein (RP) gene transcription via the TOR signaling pathway which promotes accumulation of Crf1p in the nucleus; role in repression of RP genes varies by strain	8.964276	3.164187	up
1769832_at		YNR062C		8.830218	3.142449	up
1778632_at	MET1	YKR069W	S-adenosyl-L-methionine uroporphyrinogen III transmethylase, involved in the biosynthesis of siroheme, a prosthetic group used by sulfite reductase; required for sulfate assimilation and methionine biosynthesis	8.788337	3.13559	up
1777389_at	BSC5	YNR069C	Protein of unknown function; shows homology with N-terminal end of Bul1p; ORF exhibits genomic organization compatible with a translational readthrough-dependent mode of expression; readthrough expression includes YNR068C and the locus for this readthrough is termed BUL3; Bul3p is involved in ubiquitin-mediated sorting of plasma membrane proteins; readthrough and shortened forms of Bul3p interact with Rsp5p differently in vitro	8.774368	3.133295	up

1773630_at	PFS1	YHR185C	Sporulation protein required for prospore membrane formation at selected spindle poles, ensures functionality of all four spindle pole bodies during meiosis II; not required for meiotic recombination or meiotic chromosome segregation	8.485487	3.084997	up
1779364_at	RTC2	YBR147W	Protein of unknown function; mutant produces large lipid droplets, is resistant to fluconazole, has decreased levels of rDNA transcription, growth defects on minimal media, and suppresses cdc13-1; detected in highly purified mitochondria; similar to a G-protein coupled receptor from S. pombe	8.435046	3.076396	up
1770592_at	SIP4	YJL089W	C6 zinc cluster transcriptional activator that binds to the carbon source-responsive element (CSRE) of gluconeogenic genes; involved in the positive regulation of gluconeogenesis; regulated by Snf1p protein kinase; localized to the nucleus	8.330377	3.058382	up
1769938_at	RMA1	YKL132C	Putative dihydrofolate synthetase; has similarity to Fol3p and to E. coli folylpolyglutamate synthetase/dihydrofolate synthetase; the authentic, non-tagged protein is detected in highly purified mitochondria in high-throughput studies	8.300662	3.053227	up
1773522_at	SPO23	YBR250W	Protein of unknown function; associates with meiosis-specific protein Spo1p	8.292163	3.051749	up
1777445_at		YAR068W		8.291054	3.051555	up
1779517_at	MCH2	YKL221W	Protein with similarity to mammalian monocarboxylate permeases, which are involved in transport of monocarboxylic acids across the plasma membrane; mutant is not deficient in monocarboxylate transport	8.265275	3.047063	up
1772958_at	FMP48	YGR052W	Putative protein of unknown function; the authentic, non-tagged protein is detected in highly purified mitochondria in high-throughput studies; induced by treatment with 8-methoxypsoralen and UVA irradiation	8.262063	3.046502	up
1779806_at	OSW1	YOR255W	Protein involved in sporulation; required for the construction of the outer spore wall layers; required for proper localization of Spo14p	8.227968	3.040536	up
1779892_at	MET16	YPR167C	3'-phosphoadenylsulfate reductase, reduces 3'-phosphoadenylyl sulfate to adenosine-3',5'-bisphosphate and free sulfite using reduced thioredoxin as cosubstrate, involved in sulfate assimilation and methionine metabolism	8.15694	3.028028	up
1779267_at	SHC1	YER096W	Sporulation-specific activator of Chs3p (chitin synthase III), required for the synthesis of the chitosan layer of ascospores; has similarity to Skt5p, which activates Chs3p during vegetative growth; transcriptionally induced at alkaline pH	8.114808	3.020557	up
1776384_at	YCT1	YLL055W	High-affinity cysteine-specific transporter with similarity to the Dal5p family of transporters; green fluorescent protein (GFP)-fusion protein localizes to the endoplasmic reticulum; YCT1 is not an essential gene	8.031644	3.005695	up
1772534_s_at		YAR066W		8.029957	3.005392	up
1774177_at		YNL144C		7.97073	2.994712	up
1771288_at		YGR053C		7.965531	2.993771	up
1770137_at		YML083C		7.929831	2.98729	up
1774377_at	CAT2	YML042W	Carnitine acetyl-CoA transferase present in both mitochondria and peroxisomes, transfers activated acetyl groups to carnitine to form acetylcarnitine which can be shuttled across membranes	7.925409	2.986486	up
1776865_at		YKL071W		7.897911	2.981471	up
1773545_at	SPG4	YMR107W	Protein required for survival at high temperature during stationary phase; not required for growth on	7.808433	2.965033	up

			nonfermentable carbon sources			
1773675_at	MXR1	YER042W	Methionine-S-sulfoxide reductase, involved in the response to oxidative stress; protects iron-sulfur clusters from oxidative inactivation along with MXR2; involved in the regulation of lifespan	7.782894	2.960307	up
1773890_at	APJ1	YNL077W	Putative chaperone of the HSP40 (DNAJ) family; overexpression interferes with propagation of the [Psi+] prion; the authentic, non-tagged protein is detected in highly purified mitochondria in high-throughput studies	7.764587	2.956909	up
1771435_at	NRG1	YDR043C	Transcriptional repressor that recruits the Cyc8p-Tup1p complex to promoters; mediates glucose repression and negatively regulates a variety of processes including filamentous growth and alkaline pH response	7.751461	2.954468	up
1771951_at		YJL144W		7.745742	2.953404	up
1771364_at		YHR140W		7.419619	2.891345	up
1775765_at	SRT1	YMR101C	Cis-prenyltransferase involved in synthesis of long-chain dolichols (19-22 isoprene units; as opposed to Rer2p which synthesizes shorter-chain dolichols); localizes to lipid bodies; transcription is induced during stationary phase	7.279238	2.863787	up
1773135_at	LEE1	YPL054W	Zinc-finger protein of unknown function	7.211071	2.850214	up
1780022_at		YLR053C		7.190612	2.846115	up
1779354_at	DTR1	YBR180W	Putative dityrosine transporter, required for spore wall synthesis; expressed during sporulation; member of the major facilitator superfamily (DHA1 family) of multidrug resistance transporters	7.049381	2.817497	up
1777923_at		YDR042C		7.014614	2.810364	up
1779914_at		YHL044W		7.009156	2.809241	up
1775742_at		YNR068C		7.004328	2.808247	up
1774074_s_at	VBA3	YCL069W	Permease of basic amino acids in the vacuolar membrane///Putative transporter of the Major Facilitator Superfamily (MFS); proposed role as a basic amino acid permease based on phylogeny	6.991324	2.805566	up
1775697_at		YNR064C		6.98171	2.80358	up
1774937_at	PDC5	YLR134W	Minor isoform of pyruvate decarboxylase, key enzyme in alcoholic fermentation, decarboxylates pyruvate to acetaldehyde, regulation is glucose- and ethanol-dependent, repressed by thiamine, involved in amino acid catabolism	6.84818	2.775721	up
1772831_at	ROG3	YFR022W	Protein that binds the ubiquitin ligase Rsp5p via its 2 PY motifs; has similarity to Rod1p; mutation suppresses the temperature sensitivity of an mck1 rim11 double mutant; proposed to regulate the endocytosis of plasma membrane proteins	6.836533	2.773265	up
1779211_at	JLP1	YLL057C	Fe(II)-dependent sulfonate/alpha-ketoglutarate dioxygenase, involved in sulfonate catabolism for use as a sulfur source; contains sequence that resembles a J domain (typified by the E. coli DnaJ protein); induced by sulphur starvation	6.759275	2.756869	up
1770957_at		YOR378W		6.717981	2.748028	up
1779423_at	SSP2	YOR242C	Sporulation specific protein that localizes to the spore wall; required for sporulation at a point after meiosis II and during spore wall formation; SSP2 expression is induced midway in meiosis	6.70132	2.744445	up
1775810_at		YNL194C		6.609577	2.724558	up
1773143_at	LOH1	YJL038C	Protein of unknown function with proposed roles in maintenance of genome integrity and also in spore wall assembly; induced during sporulation; repressed during vegetative growth by Sum1p and	6.558062	2.71327	up

			Hst1p; sequence similar to IRC1			
1778076_at		YBR284W		6.516462	2.704089	up
1774511_at	AAD4	YDL243C	Putative aryl-alcohol dehydrogenase; involved in oxidative stress response; similar to P. chrysosporium aryl-alcohol dehydrogenase; expression induced in cells treated with the mycotoxin patulin	6.49897	2.700211	up
1779567_at	CDA2	YLR308W	Chitin deacetylase, together with Cda1p involved in the biosynthesis ascospore wall component, chitosan; required for proper rigidity of the ascospore wall	6.444572	2.688085	up
1773662_at		YGR066C		6.420388	2.682661	up
1777476_at	CUR1	YPR158W	Sorting factor, central regulator of spatial protein quality control; physically and functionally interacts with chaperones to promote sorting and deposition of misfolded proteins into cytosolic compartments; involved in destabilization of [URE3] prions; similar in sequence to Btn2p	6.391959	2.676258	up
1777731_at	IST3	YIR005W	Component of the U2 snRNP, required for the first catalytic step of splicing and for spliceosomal assembly; interacts with Rds3p and is required for Mer1p-activated splicing	6.368421	2.670936	up
1778391_at	AZR1	YGR224W	Plasma membrane transporter of the major facilitator superfamily, involved in resistance to azole drugs such as ketoconazole and fluconazole	6.318686	2.659625	up
1770566_at	CIN5	YOR028C	Basic leucine zipper (bZIP) transcription factor of the yAP-1 family; physically interacts with the Tup1-Cyc8 complex and recruits Tup1p to its targets; mediates pleiotropic drug resistance and salt tolerance; nuclearly localized under oxidative stress and sequestered in the cytoplasm by Lot6p under reducing conditions	6.304175	2.656308	up
1773608_at	CDA1	YLR307W	Chitin deacetylase, together with Cda2p involved in the biosynthesis ascospore wall component, chitosan; required for proper rigidity of the ascospore wall	6.271396	2.648787	up
1777850_at		YNL097C-A		6.259052	2.645944	up
1770751_at		YPL272C		6.255528	2.645132	up
1778312_at	PHD1	YKL043W	Transcriptional activator that enhances pseudohyphal growth; physically interacts with the Tup1-Cyc8 complex and recruits Tup1p to its targets; regulates expression of FLO11, an adhesin required for pseudohyphal filament formation; similar to StuA, an A. nidulans developmental regulator; potential Cdc28p substrate	6.174079	2.626224	up
1777424_at	YHI9	YHR029C	Protein of unknown function; null mutant is defective in unfolded protein response; possibly involved in a membrane regulation metabolic pathway; member of the PhzF superfamily, though most likely not involved in phenazine production	6.14491	2.619392	up
1770834_at	MIF2	YKL089W	Kinetochore protein with homology to human CENP-C, required for structural integrity of the spindle during anaphase spindle elongation, interacts with histones H2A, H2B, and H4, phosphorylated by Ipl1p	6.142591	2.618847	up
1775251_at	MIP6	YHR015W	Putative RNA-binding protein, interacts with Mex67p, which is a component of the nuclear pore involved in nuclear mRNA export	6.09913	2.608604	up
1779706_at	PDR3	YBL005W	Transcriptional activator of the pleiotropic drug resistance network, regulates expression of ATP-binding cassette (ABC) transporters through binding to cis-acting sites known as PDREs (PDR responsive elements); post-translationally up-regulated in cells lacking a functional	6.089464	2.606315	up

			mitochondrial genome			
1773716_at	SPS22	YCL048W	Protein of unknown function, redundant with Sps2p for the organization of the beta-glucan layer of the spore wall	6.075986	2.603119	up
1775702_at		YMR018W		6.065654	2.600663	up
1770303_at	SUL1	YBR294W	High affinity sulfate permease of the SulP anion transporter family; sulfate uptake is mediated by specific sulfate transporters Sul1p and Sul2p, which control the concentration of endogenous activated sulfate intermediates	6.046926	2.596202	up
1778233_at	MMP1	YLL061W	High-affinity S-methylmethionine permease, required for utilization of S-methylmethionine as a sulfur source; has similarity to S-adenosylmethionine permease Sam3p	6.031263	2.59246	up
1775041_at	JID1	YPR061C	Probable Hsp40p co-chaperone, has a DnaJ-like domain and appears to be involved in ER-associated degradation of misfolded proteins containing a tightly folded cytoplasmic domain; inhibits replication of Brome mosaic virus in S. cerevisiae	6.019674	2.589685	up
1778153_at		YHR022C		6.01152	2.58773	up
1780101_at	IME2	YJL106W	Serine/threonine protein kinase involved in activation of meiosis, associates with Ime1p and mediates its stability, activates Ndt80p; IME2 expression is positively regulated by Ime1p	6.007551	2.586777	up
1772730_at	CRR1	YLR213C	Putative glycoside hydrolase of the spore wall envelope; required for normal spore wall assembly, possibly for cross-linking between the glucan and chitosan layers; expressed during sporulation	6.007377	2.586735	up
1773046_at	SLZ1	YNL196C	Sporulation-specific protein with a leucine zipper motif	6.001396	2.585298	up
1778200_at		YER187W		5.994986	2.583756	up
1772193_at		YLR297W		5.994423	2.583621	up
1775757_at	RAD59	YDL059C	Protein involved in the repair of double-strand breaks in DNA during vegetative growth via recombination and single-strand annealing; anneals complementary single-stranded DNA; homologous to Rad52p	5.985963	2.581584	up
1779400_at	NIT1	YIL165C	Nitrilase, member of the nitrilase branch of the nitrilase superfamily; in closely related species and other S. cerevisiae strain backgrounds YIL164C and adjacent ORF, YIL165C, likely constitute a single ORF encoding a nitrilase gene	5.974367	2.578786	up
1770902_at	AIM26	YKL037W	Putative protein of unknown function; null mutant is viable and displays elevated frequency of mitochondrial genome loss; null mutation confers sensitivity to tunicamycin and DTT	5.968315	2.577324	up
1774072_at	SFC1	YJR095W	Mitochondrial succinate-fumarate transporter, transports succinate into and fumarate out of the mitochondrion; required for ethanol and acetate utilization	5.958761	2.575012	up
1777421_at	RMD6	YEL072W	Protein required for sporulation	5.932934	2.568746	up
1775705_at		YOL024W		5.899272	2.560537	up
1771363_at	RTA1	YGR213C	Protein involved in 7-aminocholesterol resistance; has seven potential membrane-spanning regions; expression is induced under both low-heme and low-oxygen conditions; member of the fungal lipid-translocating exporter (LTE) family of protein	5.89921	2.560522	up
1775378_at	RPN4	YDL020C	Transcription factor that stimulates expression of proteasome genes; Rpn4p levels are in turn regulated by the 26S proteasome in a negative feedback control mechanism; RPN4 is transcriptionally regulated by various stress	5.868981	2.55311	up

			responses			
1776791_at	POX1	YGL205W	Fatty-acyl coenzyme A oxidase, involved in the fatty acid beta-oxidation pathway; localized to the peroxisomal matrix	5.862669	2.551558	up
1769759_at		YFL067W		5.86091	2.551125	up
1778834_at		YLL056C		5.83831	2.545551	up
1772055_at	VHS1	YDR247W	Cytoplasmic serine/threonine protein kinase; identified as a high-copy suppressor of the synthetic lethality of a sis2 sit4 double mutant, suggesting a role in G1/S phase progression; homolog of Sks1p	5.829936	2.54348	up
1780091_at	PDR12	YPL058C	Plasma membrane ATP-binding cassette (ABC) transporter, weak-acid-inducible multidrug transporter required for weak organic acid resistance; induced by sorbate and benzoate and regulated by War1p; mutants exhibit sorbate hypersensitivity	5.82814	2.543036	up
1769997_at	SEO1	YAL067C	Putative permease, member of the allantoate transporter subfamily of the major facilitator superfamily; mutation confers resistance to ethionine sulfoxide	5.82802	2.543006	up
1776546_at	BSC1	YDL037C	Protein of unconfirmed function, similar to cell surface flocculin Muc1p; ORF exhibits genomic organization compatible with a translational readthrough-dependent mode of expression	5.793179	2.534355	up
1778620_at	HXT14	YNL318C	Protein with similarity to hexose transporter family members, expression is induced in low glucose and repressed in high glucose; the authentic, non-tagged protein is detected in highly purified mitochondria in high-throughput studies	5.74559	2.522455	up
1772361_at	ANS1	YHR126C	Putative protein of unknown function; transcription dependent upon Azf1p	5.711977	2.51399	up
1776127_at		YMR102C		5.702616	2.511624	up
1778201_at		YDL129W		5.693432	2.509299	up
1775498_at		YDR182W-A		5.690315	2.508508	up
1778254_at		YEL057C		5.679267	2.505705	up
1778185_at	STL1	YDR536W	Glycerol proton symporter of the plasma membrane, subject to glucose-induced inactivation, strongly but transiently induced when cells are subjected to osmotic shock	5.653728	2.499203	up
1779999_s_at		YHL009W-B		5.653213	2.499071	up
1775996_at	RRT6	YGL146C	Putative protein of unknown function; non-essential gene identified in a screen for mutants with increased levels of rDNA transcription; contains two putative transmembrane spans, but no significant homology to other known proteins	5.60962	2.487903	up
1776542_at	PDR16	YNL231C	Phosphatidylinositol transfer protein (PITP) controlled by the multiple drug resistance regulator Pdr1p, localizes to lipid particles and microsomes, controls levels of various lipids, may regulate lipid synthesis, homologous to Pdr17p	5.605165	2.486757	up
1769723_at		YGR174W-A		5.598113	2.484941	up
1778121_s_at	PAU3	YCR104W	Protein of unknown function, member of the seripauperin multigene family encoded mainly in subtelomeric regions///Protein of unknown function, member of the seripauperin multigene family encoded mainly in subtelomeric regions///Protein of unknown function, member of the seripauperin multigene family encoded mainly in subtelomeric regions///Protein of unknown function, member of the seripauperin multigene family encoded mainly in subtelomeric regions;	5.569578	2.477568	up

			identical to Pau22p; encodes 2 proteins that are translated from 2 different start codons///Protein of unknown function, member of the seripauperin multigene family encoded mainly in subtelomeric regions; identical to Pau21p; encodes 2 proteins that are translated from 2 different start codons///Member of the seripauperin multigene family encoded mainly in subtelomeric regions, active during alcoholic fermentation, regulated by anaerobiosis, negatively regulated by oxygen, repressed by heme			
1775220_at	PRM1	YNL279W	Pheromone-regulated multispanning membrane protein involved in membrane fusion during mating; predicted to have 5 transmembrane segments and a coiled coil domain; localizes to the shmoo tip; regulated by Ste12p	5.544257	2.470994	up
1771738_at	ALP1	YNL270C	Arginine transporter; expression is normally very low and it is unclear what conditions would induce significant expression	5.496631	2.458548	up
1772566_at	IME1	YJR094C	Master regulator of meiosis that is active only during meiotic events, activates transcription of early meiotic genes through interaction with Ume6p, degraded by the 26S proteasome following phosphorylation by Ime2p	5.478154	2.45369	up
1779784_at		YJL133C-A		5.445981	2.445192	up
1772278_at	ZIP1	YDR285W	Transverse filament protein of the synaptonemal complex; required for normal levels of meiotic recombination and pairing between homologous chromosome during meiosis; potential Cdc28p substrate	5.430843	2.441176	up
1771546_at	MVP1	YMR004W	Protein required for sorting proteins to the vacuole; overproduction of Mvp1p suppresses several dominant VPS1 mutations; Mvp1p and Vps1p act in concert to promote membrane traffic to the vacuole; participates in transcription initiation and/or early elongation of specific genes; interacts with foot domain of RNA polymerase II; deletion results in abnormal CTD-Ser5 phosphorylation of RNA polymerase II at specific promoter regions	5.423694	2.439276	up
1778593_at		YKR015C		5.382972	2.428403	up
1774166_at	ECM23	YPL021W	Non-essential protein of unconfirmed function; affects pre-rRNA processing, may act as a negative regulator of the transcription of genes involved in pseudohyphal growth; homologous to Srd1p	5.377637	2.426972	up
1779410_at	PUG1	YER185W	Plasma membrane protein with roles in the uptake of protoprophyrin IX and the efflux of heme; expression is induced under both low-heme and low-oxygen conditions; member of the fungal lipid-translocating exporter (LTE) family of proteins	5.376292	2.426611	up
1773395_at	NIT1	YIL164C	Nitrilase, member of the nitrilase branch of the nitrilase superfamily; in closely related species and other S. cerevisiae strain backgrounds YIL164C and adjacent ORF, YIL165C, likely constitute a single ORF encoding a nitrilase gene	5.361274	2.422576	up
1777239_at		YGL258W-A		5.356362	2.421253	up
1769781_at	SHE9	YDR393W	Mitochondrial inner membrane protein required for normal mitochondrial morphology, may be involved in fission of the inner membrane; forms a homo-oligomeric complex	5.349562	2.419421	up
1776054_at	VHR1	YIL056W	Transcriptional activator, required for the vitamin H-responsive element (VHRE) mediated induction of VHT1 (Vitamin H transporter) and BIO5 (biotin biosynthesis intermediate transporter) in response to low biotin concentrations	5.316503	2.410478	up

1771934_at		YOR365C		5.29233	2.403903	up
1774365_at	MET14	YKL001C	Adenylylsulfate kinase, required for sulfate assimilation and involved in methionine metabolism	5.272811	2.398572	up
1778151_at	DUG3	YNL191W	Component of glutamine amidotransferase (GATase II); forms a complex with Dug2p to degrade glutathione (GSH) and other peptides containing a gamma-glu-X bond in an alternative pathway to GSH degradation by gamma-glutamyl transpeptidase (Ecm38p)	5.256684	2.394153	up
1779438_at	SET4	YJL105W	Protein of unknown function, contains a SET domain	5.251644	2.392769	up
1777665_at	RAD17	YOR368W	Checkpoint protein, involved in the activation of the DNA damage and meiotic pachytene checkpoints; with Mec3p and Ddc1p, forms a clamp that is loaded onto partial duplex DNA; homolog of human and S. pombe Rad1 and U. maydis Rec1 proteins	5.242639	2.390293	up
1771824_at	ICY2	YPL250C	Protein of unknown function; mobilized into polysomes upon a shift from a fermentable to nonfermentable carbon source; potential Cdc28p substrate	5.237261	2.388813	up
1776664_at	DAK2	YFL053W	Dihydroxyacetone kinase, required for detoxification of dihydroxyacetone (DHA); involved in stress adaptation	5.220055	2.384065	up
1769585_at		YBR285W		5.207631	2.380627	up
1771375_at	MOH1	YBL049W	Protein of unknown function, has homology to kinase Snf7p; not required for growth on nonfermentable carbon sources; essential for survival in stationary phase	5.202232	2.379131	up
1773498_at	MET3	YJR010W	ATP sulfurylase, catalyzes the primary step of intracellular sulfate activation, essential for assimilatory reduction of sulfate to sulfide, involved in methionine metabolism	5.151737	2.365059	up
1777780_at	ADH7	YCR105W	NADPH-dependent medium chain alcohol dehydrogenase with broad substrate specificity; member of the cinnamyl family of alcohol dehydrogenases; may be involved in fusel alcohol synthesis or in aldehyde tolerance	5.131182	2.359291	up
1775070_at	YIG1	YPL201C	Protein that interacts with glycerol 3-phosphatase and plays a role in anaerobic glycerol production; localizes to the nucleus and cytosol	5.119007	2.355864	up
1770279_at	LYS1	YIR034C	Saccharopine dehydrogenase (NAD+, L-lysine-forming), catalyzes the conversion of saccharopine to L-lysine, which is the final step in the lysine biosynthesis pathway; also has mRNA binding activity	5.112575	2.35405	up
1772133_at	PEX5	YDR244W	Peroxisomal membrane signal receptor for the C-terminal tripeptide signal sequence (PTS1) of peroxisomal matrix proteins, required for peroxisomal matrix protein import; also proposed to have PTS1-receptor independent functions	5.074761	2.34334	up
1770118_at	GAC1	YOR178C	Regulatory subunit for Glc7p type-1 protein phosphatase (PP1), tethers Glc7p to Gsy2p glycogen synthase, binds Hsf1p heat shock transcription factor, required for induction of some HSF-regulated genes under heat shock	5.064587	2.340445	up
1770125_at		YDL218W		5.050685	2.336479	up
1777298_at		YIL152W		5.045521	2.335003	up
1775035_at		YEL023C		5.025721	2.32933	up
1770378_at	PXA2	YKL188C	Subunit of a heterodimeric peroxisomal ATP-binding cassette transporter complex (Pxa1p-Pxa2p), required for import of long-chain fatty acids into peroxisomes; similarity to human	5.010248	2.324882	up

			adrenoleukodystrophy transporter ABCD1 and ABCD2 and ALD-related proteins; mutations in ABCD1 cause X-linked adrenoleukodystrophy (X-ALD), a peroxisomal disorder			
1774534_at	DGR1	YNL130C-A	Protein of unknown function; dgr1 null mutant is resistant to 2-deoxy-D-glucose	4.985235	2.317662	up
1779450_at		YNR014W		4.974425	2.31453	up
1773792_at	FYV10	YIL097W	Subunit of GID complex; involved in proteasome-dependent catabolite inactivation of gluconeogenic enzymes FBPase, PEPCK, and c-MDH; interacts with Rmd5p and contains a degenerate RING finger motif needed for GID complex ubiquitin ligase activity in vivo; contains CTLH domain; plays role in anti-apoptosis; required for survival upon exposure to K1 killer toxin	4.936845	2.303589	up
1772391_at	RGI2	YIL057C	Protein of unknown function involved in energy metabolism under respiratory conditions; expression induced under carbon limitation and repressed under high glucose	4.871645	2.284409	up
1773966_at	YPT53	YNL093W	Rab family GTPase, similar to Ypt51p and Ypt52p and to mammalian rab5; required for vacuolar protein sorting and endocytosis	4.859311	2.280752	up
1779332_at		YPR027C		4.838077	2.274434	up
1772192_at	DRE2	YKR071C	Conserved component of an early step in the cytosolic Fe-S protein assembly (CIA) machinery; contains an Fe-S cluster that receives electrons from NADPH via the action of Tah18p; ortholog of human Ciapin1	4.830771	2.272254	up
1769982_at	ECM8	YBR076W	Non-essential protein of unknown function	4.804988	2.264533	up
1776767_at	MHT1	YLL062C	S-methylmethionine-homocysteine methyltransferase, functions along with Sam4p in the conversion of S-adenosylmethionine (AdoMet) to methionine to control the methionine/AdoMet ratio	4.802694	2.263844	up
1778973_at	PDR15	YDR406W	Plasma membrane ATP binding cassette (ABC) transporter, multidrug transporter and general stress response factor implicated in cellular detoxification; regulated by Pdr1p, Pdr3p and Pdr8p; promoter contains a PDR responsive element	4.802429	2.263764	up
1775904_at	GAT2	YMR136W	Protein containing GATA family zinc finger motifs; similar to Gln3p and Dal80p; expression repressed by leucine	4.796346	2.261936	up
1771222_at	TOS8	YGL096W	Homeodomain-containing protein and putative transcription factor found associated with chromatin; target of SBF transcription factor; induced during meiosis and under cell-damaging conditions; similar to Cup9p transcription factor	4.793384	2.261045	up
1770580_at	ADY2	YCR010C	Acetate transporter required for normal sporulation; phosphorylated in mitochondria	4.697035	2.231751	up
1777947_at	SNX41	YDR425W	Sorting nexin, involved in the retrieval of late-Golgi SNAREs from the post-Golgi endosome to the trans-Golgi network; interacts with Snx4p	4.695629	2.231319	up
1779681_at	DIT1	YDR403W	Sporulation-specific enzyme required for spore wall maturation, involved in the production of a soluble LL-dityrosine-containing precursor of the spore wall; transcripts accumulate at the time of prospore enclosure	4.654425	2.218603	up
1777857_at		YPL014W		4.651502	2.217697	up
1773553_at	ENT1	YDL161W	Epsin-like protein involved in endocytosis and actin patch assembly and functionally redundant with Ent2p; binds clathrin via a clathrin-binding domain motif at C-terminus	4.637741	2.213422	up
1776087_at	ECM12	YHR021W-A	Putative protein of unknown function; may	4.623431	2.208964	up

			contribute to cell wall biosynthesis, mutants display zymolyase hypersensitivity			
1775868_s_at	HXT17	YNR072W	Hexose transporter, induced in the presence of non-fermentable carbon sources, induced by low levels of glucose, repressed by high levels of glucose///Hexose transporter, up-regulated in media containing raffinose and galactose at pH 7.7 versus pH 4.7, repressed by high levels of glucose	4.621659	2.208411	up
1779358_at	PKH2	YOL100W	Serine/threonine protein kinase involved in sphingolipid-mediated signaling pathway that controls endocytosis; activates Ypk1p and Ykr2p, components of signaling cascade required for maintenance of cell wall integrity; redundant with Pkh1p	4.61801	2.207271	up
1778769_at	SFK1	YKL051W	Plasma membrane protein that may act together with or upstream of Stt4p to generate normal levels of the essential phospholipid PI4P, at least partially mediates proper localization of Stt4p to the plasma membrane	4.600113	2.201669	up
1778888_at	TID3	YIL144W	Component of the evolutionarily conserved kinetochore-associated Ndc80 complex (Ndc80p-Nuf2p-Spc24p-Spc25p); conserved coiled-coil protein involved in chromosome segregation, spindle checkpoint activity, kinetochore assembly and clustering	4.529223	2.179264	up
1771192_at		YAL067W-A		4.520026	2.176331	up
1771291_at		YAL037C-A		4.516991	2.175362	up
1776153_at		YMR206W		4.512078	2.173792	up
1776107_at		YBR298C-A		4.492982	2.167673	up
1779369_at	BAG7	YOR134W	Rho GTPase activating protein (RhoGAP), stimulates the intrinsic GTPase activity of Rho1p, which plays a role in actin cytoskeleton organization and control of cell wall synthesis; structurally and functionally related to Sac7p	4.490562	2.166896	up
1778832_at		YIR042C		4.490505	2.166878	up
1774845_at	SMA2	YML066C	Meiosis-specific prospore membrane protein; required to produce bending force necessary for proper assembly of the prospore membrane during sporulation	4.480588	2.163688	up
1775678_at	PUT1	YLR142W	Proline oxidase, nuclear-encoded mitochondrial protein involved in utilization of proline as sole nitrogen source; PUT1 transcription is induced by Put3p in the presence of proline and the absence of a preferred nitrogen source	4.462654	2.157902	up
1774952_at	SUL2	YLR092W	High affinity sulfate permease; sulfate uptake is mediated by specific sulfate transporters Sul1p and Sul2p, which control the concentration of endogenous activated sulfate intermediates	4.453811	2.15504	up
1769551_at	FUN19	YAL034C	Non-essential protein of unknown function; expression induced in response to heat stress	4.450725	2.15404	up
1773511_at	YAP6	YDR259C	Basic leucine zipper (bZIP) transcription factor; physically interacts with the Tup1-Cyc8 complex and recruits Tup1p to its targets; overexpression increases sodium and lithium tolerance; computational analysis suggests a role in regulation of expression of genes involved in carbohydrate metabolism	4.445932	2.152486	up
1773980_at	SOV1	YMR066W	Mitochondrial protein of unknown function	4.42259	2.144892	up
1778246_at	TMT1	YER175C	Trans-aconitate methyltransferase, cytosolic enzyme that catalyzes the methyl esterification of 3-isopropylmalate, an intermediate of the leucine biosynthetic pathway, and trans-aconitate, which inhibits the citric acid cycle	4.388721	2.133801	up

1774011_at	PFK26	YIL107C	6-phosphofructo-2-kinase, inhibited by phosphoenolpyruvate and sn-glycerol 3-phosphate; has negligible fructose-2,6-bisphosphatase activity; transcriptional regulation involves protein kinase A	4.380598	2.131128	up
1772576_x_at	PAU7	YAR020C	Member of the seripauperin multigene family, active during alcoholic fermentation, regulated by anaerobiosis, inhibited by oxygen, repressed by heme	4.378313	2.130375	up
1771775_at		YOL047C		4.374578	2.129144	up
1770230_at		YGR204C-A		4.372266	2.128381	up
1777917_at	MTH1	YDR277C	Negative regulator of the glucose-sensing signal transduction pathway, required for repression of transcription by Rgt1p; interacts with Rgt1p and the Snf3p and Rgt2p glucose sensors; phosphorylated by Yck1p, triggering Mth1p degradation	4.347643	2.120234	up
1773363_x_at		YLL066W-B		4.343947	2.119007	up
1772073_at	OXR1	YPL196W	Protein of unknown function required for normal levels of resistance to oxidative damage, null mutants are sensitive to hydrogen peroxide; member of a conserved family of proteins found in eukaryotes	4.327579	2.11356	up
1773838_at	RAD26	YJR035W	Protein involved in transcription-coupled nucleotide excision repair of UV-induced DNA lesions; recruitment to DNA lesions is dependent on an elongating RNA polymerase II; homolog of human CSB protein	4.325272	2.112791	up
1776553_at		YOL163W		4.32167	2.111589	up
1779083_at		YFL040W		4.317884	2.110324	up
1775462_at	STR2	YJR130C	Cystathionine gamma-synthase, converts cysteine into cystathionine	4.313998	2.109026	up
1771252_at	SCM3	YDL139C	Nonhistone component of centromeric chromatin that binds stoichiometrically to CenH3-H4 histones, required for kinetochore assembly; required for G2/M progression and localization of Cse4p; may protect Cse4p from ubiquitylation	4.312759	2.108611	up
1774775_at	SSP1	YHR184W	Protein involved in the control of meiotic nuclear division and coordination of meiosis with spore formation; transcription is induced midway through meiosis	4.298058	2.103685	up
1770529_at	OPI10	YOL032W	Protein with a possible role in phospholipid biosynthesis, based on inositol-excreting phenotype of the null mutant and its suppression by exogenous choline	4.296025	2.103003	up
1777033_at	FIT3	YOR383C	Mannoprotein that is incorporated into the cell wall via a glycosylphosphatidylinositol (GPI) anchor, involved in the retention of siderophore-iron in the cell wall	4.288685	2.100535	up
1776844_at	PRM7	YDL038C	Pheromone-regulated protein, predicted to have one transmembrane segment; promoter contains Gcn4p binding elements	4.274654	2.095808	up
1779435_at		YIR024C		4.253454	2.088635	up
1775340_at	UGX2	YDL169C	Protein of unknown function, transcript accumulates in response to any combination of stress conditions	4.246817	2.086382	up
1777690_at	XBP1	YIL101C	Transcriptional repressor that binds to promoter sequences of the cyclin genes, CYS3, and SMF2; expression is induced by stress or starvation during mitosis, and late in meiosis; member of the Swi4p/Mbp1p family; potential Cdc28p substrate	4.237006	2.083045	up
1778271_at	ROX1	YPR065W	Heme-dependent repressor of hypoxic genes; contains an HMG domain that is responsible for DNA bending activity	4.236755	2.08296	up

1770009_at	MGR3	YMR115W	Subunit of the mitochondrial (mt) i-AAA protease supercomplex, which degrades misfolded mitochondrial proteins; forms a subcomplex with Mgr1p that binds to substrates to facilitate proteolysis; required for growth of cells lacking mtDNA	4.228944	2.080298	up
1771515_at		YML131W		4.228035	2.079988	up
1771632_at	DON1	YDR273W	Meiosis-specific component of the spindle pole body, part of the leading edge protein (LEP) coat, forms a ring-like structure at the leading edge of the prospore membrane during meiosis II	4.21251	2.07468	up
1776170_at		YMR034C		4.198814	2.069982	up
1777575_at		YJR115W		4.198668	2.069932	up
1775854_s_at		YHL009W-A		4.169864	2.06	up
1778862_s_at	HMLA LPHA1	YCL066W	Silenced copy of ALPHA1 at HML, encoding a transcriptional coactivator involved in the regulation of mating-type alpha-specific gene expression///Transcriptional co-activator involved in regulation of mating-type-specific gene expression; targets the transcription factor Mcm1p to the promoters of alpha-specific genes; one of two genes encoded by the MATalpha mating type cassette	4.164856	2.058266	up
1770920_at	COA2	YPL189C-A	Cytochrome oxidase assembly factor; null mutation results in respiratory deficiency with specific loss of cytochrome oxidase activity; functions downstream of assembly factors Mss51p and Coa1p and interacts with assembly factor Shy1p	4.164528	2.058153	up
1773436_x_a t	PAU23	YLR037C	Cell wall mannoprotein with similarity to Tir1p, Tir2p, Tir3p, and Tir4p; member of the seripauperin multigene family encoded mainly in subtelomeric regions; expressed under anaerobic conditions, completely repressed during aerobic growth	4.160027	2.056593	up
1773594_at	MAL31	YBR298C	Maltose permease, high-affinity maltose transporter (alpha-glucoside transporter); encoded in the MAL3 complex locus; member of the 12 transmembrane domain superfamily of sugar transporters; functional in genomic reference strain S288C	4.157418	2.055688	up
1770182_at	REC104	YHR157W	Protein involved in early stages of meiotic recombination; required for meiotic crossing over; forms a complex with Rec102p and Spo11p necessary during the initiation of recombination	4.148469	2.052579	up
1773182_at	HRP1	YOL123W	Subunit of cleavage factor I, a five-subunit complex required for the cleavage and polyadenylation of pre-mRNA 3' ends; RRM-containing heteronuclear RNA binding protein and hnRNPA/B family member that binds to poly (A) signal sequences	4.14498	2.051365	up
1771705_at	SPI1	YER150W	GPI-anchored cell wall protein involved in weak acid resistance; basal expression requires Msn2p/Msn4p; expression is induced under conditions of stress and during the diauxic shift; similar to Sed1p	4.139731	2.049537	up
1778181_at	ATG29	YPL166W	Autophagy-specific protein that is required for recruitment of other ATG proteins to the pre-autophagosomal structure (PAS); interacts with Atg17p and localizas to the PAS in a manner interdependent with Atg17p and Cis1p; not conserved	4.131736	2.046748	up
1774042_at	SPC29	YPL124W	Inner plaque spindle pole body (SPB) component, links the central plaque component Spc42p to the inner plaque component Spc110p; required for SPB duplication	4.124535	2.044231	up

1773882_at	OSW2	YLR054C	Protein of unknown function proposed to be involved in the assembly of the spore wall	4.122191	2.043411	up
1769675_at		YDL177C		4.116984	2.041588	up
1774769_at	PRM2	YIL037C	Pheromone-regulated protein, predicted to have 4 transmembrane segments and a coiled coil domain; regulated by Ste12p; required for efficient nuclear fusion	4.115704	2.041139	up
1777027_at	STP3	YLR375W	Zinc-finger protein of unknown function, possibly involved in pre-tRNA splicing and in uptake of branched-chain amino acids	4.111335	2.039607	up
1774328_at		YPR108W-A		4.110498	2.039313	up
1774360_at	ART10	YLR392C	Protein of unknown function that contains 2 PY motifs and is ubiquinated by Rsp5p; overexpression confers resistance to arsenite; green fluorescent protein (GFP)-fusion protein localizes it to the cytoplasm; non-essential gene	4.10538	2.037516	up
1776630_at	GTO1	YGR154C	Omega-class glutathione transferase; induced under oxidative stress; putative peroxisomal localization	4.102913	2.036649	up
1775663_at	MET5	YJR137C	Sulfite reductase beta subunit, involved in amino acid biosynthesis, transcription repressed by methionine	4.090857	2.032403	up
1778847_at	PGU1	YJR153W	Endo-polygalacturonase, pectolytic enzyme that hydrolyzes the alpha-1,4-glycosidic bonds in the rhamnogalacturonan chains in pectins	4.077634	2.027732	up
1771340_at	BOP2	YLR267W	Protein of unknown function	4.070522	2.025214	up
1772007_at		YIL029C		4.064892	2.023217	up
1780117_at	SPS1	YDR523C	Putative protein serine/threonine kinase expressed at the end of meiosis and localized to the prospore membrane, required for correct localization of enzymes involved in spore wall synthesis	4.064818	2.023191	up
1772919_at	LIN1	YHR156C	Non-essential component of U5 snRNP; nuclear protein; physically interacts with Irr1p of cohesin complex; may link together proteins involved in chromosome segregation, mRNA splicing and DNA replication	4.06468	2.023142	up
1770202_at	SSA4	YER103W	Heat shock protein that is highly induced upon stress; plays a role in SRP-dependent cotranslational protein-membrane targeting and translocation; member of the HSP70 family; cytoplasmic protein that concentrates in nuclei upon starvation	4.064348	2.023024	up
1769452_at	ULP1	YPL020C	Protease that specifically cleaves Smt3p protein conjugates; required for cell cycle progression; associates with nucleoporins and may interact with septin rings during telophase; sequestered to the nucleolus under stress conditions	4.045556	2.016338	up
1776294_at	HXT8	YJL214W	Protein of unknown function with similarity to hexose transporter family members, expression is induced by low levels of glucose and repressed by high levels of glucose	4.036307	2.013036	up
1770851_at	SIR1	YKR101W	Protein involved in repression of transcription at the silent mating-type loci HML and HMR; recruitment to silent chromatin requires interactions with Orc1p and with Sir4p, through a common Sir1p domain; binds to centromeric chromatin	4.036194	2.012996	up
1771326_at	SPR1	YOR190W	Sporulation-specific exo-1,3-beta-glucanase; contributes to ascospore thermoresistance	4.030902	2.011103	up
1776778_at	ISF1	YMR081C	Serine-rich, hydrophilic protein with similarity to Mbr1p; overexpression suppresses growth defects of hap2, hap3, and hap4 mutants; expression is under glucose control; cotranscribed with NAM7 in a cyp1 mutant	4.02425	2.00872	up
1771078_at	FRM2	YCL026C-A	Type II nitroreductase, using NADH as reductant; mutants are defective in fatty acid mediated	4.012	2.004322	up

			repression of genes involved in fatty acid biosynthesis indicative of a role in lipid signaling; involved in the oxidative stress response; transcription induction by cadmium and selenite indicates a possible role in the metal stress response; expression induced in cells treated with the mycotoxin patulin			
1780044_at	RNR3	YIL066C	Minor isoform of the large subunit of ribonucleotide-diphosphate reductase; the RNR complex catalyzes rate-limiting step in dNTP synthesis, regulated by DNA replication and DNA damage checkpoint pathways via localization of small subunits	4.001743	2.000629	up
1770123_at		YJL218W		3.998401	1.999423	up
1772393_at		YLR154W-E		3.98682	1.995238	up
1769713_at		YOL162W		3.979797	1.992695	up
1777129_at	YJU2	YKL095W	Essential protein required for pre-mRNA splicing; associates transiently with the spliceosomal NTC (nineteen complex) and acts after Prp2p to promote the first catalytic reaction of splicing	3.974282	1.990694	up
1777714_at	DIP5	YPL265W	Dicarboxylic amino acid permease, mediates high-affinity and high-capacity transport of L-glutamate and L-aspartate; also a transporter for Gln, Asn, Ser, Ala, and Gly	3.97013	1.989186	up
1777276_at	UBP9	YER098W	Ubiquitin carboxyl-terminal hydrolase, ubiquitin-specific protease that cleaves ubiquitin-protein fusions	3.96443	1.987114	up
1773497_at	BSC4	YNL269W	Protein of unknown function, ORF exhibits genomic organization compatible with a translational readthrough-dependent mode of expression; readthrough is increased upon depletion of Sup35p	3.951288	1.982323	up
1773681_at	VID24	YBR105C	Peripheral membrane protein located at Vid (vacuole import and degradation) vesicles; regulates fructose-1,6-bisphosphatase (FBPase) targeting to the vacuole; promotes proteasome-dependent catabolite degradation of FBPase	3.951223	1.982299	up
1778265_at	FRE4	YNR060W	Ferric reductase, reduces a specific subset of siderophore-bound iron prior to uptake by transporters; expression induced by low iron levels	3.946901	1.98072	up
1769474_at		YPL264C		3.935042	1.976379	up
1774005_at		YNL155W		3.932322	1.975381	up
1775133_at	SNX4	YJL036W	Sorting nexin, involved in retrieval of late-Golgi SNAREs from post-Golgi endosomes to the trans-Golgi network and in cytoplasm to vacuole transport; contains a PX phosphoinositide-binding domain; forms complexes with Snx41p and with Atg20p	3.901652	1.964085	up
1780054_at	ECO1	YFR027W	Acetyltransferase required for establishment of sister chromatid cohesion; modifies Smc3p at replication forks and Mcd1p in response to ds DNA breaks; has a C2H2-type zinc finger; mutations in human homolog ESCO2 cause Roberts syndrome	3.875977	1.95456	up
1771419_at	SKS1	YPL026C	Putative serine/threonine protein kinase; involved in the adaptation to low concentrations of glucose independent of the SNF3 regulated pathway	3.872973	1.953442	up
1776041_at	ATG1	YGL180W	Protein ser/thr kinase required for vesicle formation in autophagy and the cytoplasm-to-vacuole targeting (Cvt) pathway; structurally required for phagophore assembly site formation; during autophagy forms a complex with Atg13p and Atg17p	3.86633	1.950965	up
1771162_at	JJJ2	YJL162C	Protein of unknown function, contains a J-domain, which is a region with homology to the E. coli	3.858958	1.948211	up

			DnaJ protein			
1779613_at	GTT2	YLL060C	Glutathione S-transferase capable of homodimerization; functional overlap with Gtt2p, Grx1p, and Grx2p	3.843769	1.942522	up
1772997_at	GRE2	YOL151W	3-methylbutanal reductase and NADPH-dependent methylglyoxal reductase (D-lactaldehyde dehydrogenase); stress induced (osmotic, ionic, oxidative, heat shock and heavy metals); regulated by the HOG pathway	3.84213	1.941907	up
1772467_s_at	RSC30	YHR054C	Component of the RSC chromatin remodeling complex; non-essential gene required for regulation of ribosomal protein genes and the cell wall/stress response; highly similar to Rsc3p; null mutants are osmosensitive	3.82698	1.936206	up
1770156_at	CRP1	YHR146W	Protein that binds to cruciform DNA structures	3.825961	1.935822	up
1773106_at	VPS20	YMR077C	Myristoylated subunit of ESCRTIII, the endosomal sorting complex required for transport of transmembrane proteins into the multivesicular body pathway to the lysosomal/vacuolar lumen; cytoplasmic protein recruited to endosomal membranes	3.816245	1.932154	up
1772755_at	MCH1	YDL054C	Protein with similarity to mammalian monocarboxylate permeases, which are involved in transport of monocarboxylic acids across the plasma membrane; mutant is not deficient in monocarboxylate transport	3.81284	1.930866	up
1770843_at	IRC10	YOL015W	Putative protein of unknown function; null mutant displays increased levels of spontaneous Rad52p foci	3.812224	1.930633	up
1775835_at	ACM1	YPL267W	Pseudosubstrate inhibitor of the anaphase-promoting complex/cyclosome (APC/C), that suppresses APC/C [Cdh1]-mediated proteolysis of mitotic cyclins; associates with Cdh1p, Bmh1p and Bmh2p; cell cycle regulated protein	3.810336	1.929918	up
1773976_at	COS111	YBR203W	Protein required for resistance to the antifungal drug ciclopirox olamine; not related to the subtelomerically-encoded COS family; the authentic, non-tagged protein is detected in highly purified mitochondria in high-throughput studies	3.808871	1.929363	up
1777980_at	DCP1	YOL149W	Subunit of the Dcp1p-Dcp2p decapping enzyme complex, which removes the 5' cap structure from mRNAs prior to their degradation; enhances the activity of catalytic subunit Dcp2p; regulated by DEAD box protein Dhh1p	3.808659	1.929283	up
1776343_at	ATG20	YDL113C	Sorting nexin family member required for the cytoplasm-to-vacuole targeting (Cvt) pathway and for endosomal sorting; has a Phox homology domain that binds phosphatidylinositol-3-phosphate; interacts with Snx4p; potential Cdc28p substrate	3.807952	1.929015	up
1779119_at		YLR412C-A		3.793949	1.9237	up
1777414_at	RIM8	YGL045W	Protein involved in proteolytic activation of Rim101p in response to alkaline pH; interacts with ESCRT-1 subunits Stp22p and Vps28p; essential for anaerobic growth; member of the arrestin-related trafficking adaptor family	3.790827	1.922513	up
1775271_at	USV1	YPL230W	Putative transcription factor containing a C2H2 zinc finger; mutation affects transcriptional regulation of genes involved in growth on non-fermentable carbon sources, response to salt stress and cell wall biosynthesis	3.776804	1.917166	up
1770636_at	DOC1	YGL240W	Processivity factor required for the ubiquitination activity of the anaphase promoting complex (APC), mediates the activity of the APC by contributing to substrate recognition; involved in cyclin	3.775488	1.916663	up

			proteolysis; contains a conserved DOC1 homology domain			
1770367_at		YFR035C		3.757013	1.909586	up
1772347_at	CCC2	YDR270W	Cu(+2)-transporting P-type ATPase, required for export of copper from the cytosol into an extracytosolic compartment; has similarity to human proteins involved in Menkes and Wilsons diseases	3.754953	1.908795	up
1779702_at	IME4	YGL192W	Probable mRNA N6-adenosine methyltransferase required for entry into meiosis; transcribed in diploid cells; haploids repress IME4 transcription via production of antisense IME4 transcripts; antisense transcription is repressed in diploids	3.740512	1.903236	up
1778914_at		YJL160C		3.739125	1.902701	up
1771588_at	SPT21	YMR179W	Protein with a role in transcriptional silencing; required for normal transcription at several loci including HTA2-HTB2 and HHF2-HHT2, but not required at the other histone loci; functionally related to Spt10p	3.729906	1.899139	up
1770217_at	TAF12	YDR145W	Subunit (61/68 kDa) of TFIID and SAGA complexes, involved in RNA polymerase II transcription initiation and in chromatin modification, similar to histone H2A	3.717881	1.894481	up
1778969_x_at	PAU2	YEL049W	Member of the seripauperin multigene family encoded mainly in subtelomeric regions, active during alcoholic fermentation, regulated by anaerobiosis, negatively regulated by oxygen, repressed by heme	3.702479	1.888492	up
1773281_at	RAD54	YGL163C	DNA-dependent ATPase, stimulates strand exchange by modifying the topology of double-stranded DNA; involved in the recombinational repair of double-strand breaks in DNA during vegetative growth and meiosis; member of the SWI/SNF family	3.696576	1.88619	up
1779568_at	REG2	YBR050C	Regulatory subunit of the Glc7p type-1 protein phosphatase; involved with Reg1p, Glc7p, and Snf1p in regulation of glucose-repressible genes, also involved in glucose-induced proteolysis of maltose permease	3.695405	1.885732	up
1775953_s_at		YNL034W		3.694642	1.885435	up
1776944_at	MAL11	YGR289C	Inducible high-affinity maltose transporter (alpha-glucoside transporter); encoded in the MAL1 complex locus; broad substrate specificity that includes maltotriose; required for isomaltose utilization	3.674701	1.877627	up
1773191_at	UGA3	YDL170W	Transcriptional activator necessary for gamma-aminobutyrate (GABA)-dependent induction of GABA genes (such as UGA1, UGA2, UGA4); zinc-finger transcription factor of the Zn(2)-Cys(6) binuclear cluster domain type; localized to the nucleus	3.654578	1.869705	up
1775576_at	CTH1	YDR151C	Member of the CCCH zinc finger family; has similarity to mammalian Tis11 protein, which activates transcription and also has a role in mRNA degradation; may function with Tis11p in iron homeostasis	3.650616	1.86814	up
1773089_at	FET3	YMR058W	Ferro-O2-oxidoreductase required for high-affinity iron uptake and involved in mediating resistance to copper ion toxicity, belongs to class of integral membrane multicopper oxidases	3.646724	1.866601	up
1778824_at		YJL049W		3.644959	1.865902	up
1770191_at	MAG1	YER142C	3-methyl-adenine DNA glycosylase involved in protecting DNA against alkylating agents; initiates base excision repair by removing damaged bases to	3.636523	1.86256	up

			create abasic sites that are subsequently repaired			
1777198_at		YJR030C		3.634789	1.861872	up
1771776_at	CCL1	YPR025C	Cyclin associated with protein kinase Kin28p, which is the TFIIH-associated carboxy-terminal domain (CTD) kinase involved in transcription initiation at RNA polymerase II promoters	3.631802	1.860685	up
1779207_at		YKL068W-A		3.628924	1.859542	up
1769313_at	PKP2	YGL059W	Mitochondrial protein kinase that negatively regulates activity of the pyruvate dehydrogenase complex by phosphorylating the ser-133 residue of the Pda1p subunit; acts in concert with kinase Pkp1p and phosphatases Ptc5p and Ptc6p	3.612855	1.853139	up
1769607_at	KAR9	YPL269W	Karyogamy protein required for correct positioning of the mitotic spindle and for orienting cytoplasmic microtubules, localizes at the shmoo tip in mating cells and at the tip of the growing bud in small-budded cells through anaphase	3.611075	1.852428	up
1780105_at	YTP1	YNL237W	Probable type-III integral membrane protein of unknown function, has regions of similarity to mitochondrial electron transport proteins	3.601842	1.848735	up
1774717_at	YAP5	YIR018W	Basic leucine zipper (bZIP) iron-sensing transcription factor	3.576691	1.838625	up
1769342_at	POT1	YIL160C	3-ketoacyl-CoA thiolase with broad chain length specificity, cleaves 3-ketoacyl-CoA into acyl-CoA and acetyl-CoA during beta-oxidation of fatty acids	3.567726	1.835005	up
1779736_at		YER039C-A		3.562119	1.832736	up
1769681_at	WSC3	YOL105C	Partially redundant sensor-transducer of the stress-activated PKC1-MPK1 signaling pathway involved in maintenance of cell wall integrity; involved in the response to heat shock and other stressors; regulates 1,3-beta-glucan synthesis	3.557586	1.830899	up
1776321_at		YAL063C-A		3.55431	1.829569	up
1775738_at		YBR184W		3.532888	1.820848	up
1770317_at	TPO1	YLL028W	Polyamine transporter that recognizes spermine, putrescine, and spermidine; catalyzes uptake of polyamines at alkaline pH and excretion at acidic pH; phosphorylation enhances activity and sorting to the plasma membrane	3.529799	1.819586	up
1778106_x_at		YLR462W		3.529685	1.81954	up
1769666_at	APC2	YLR127C	Subunit of the Anaphase-Promoting Complex/Cyclosome (APC/C), which is a ubiquitin-protein ligase required for degradation of anaphase inhibitors, including mitotic cyclins, during the metaphase/anaphase transition; component of the catalytic core of the APC/C; has similarity to cullin Cdc53p	3.527427	1.818616	up
1773588_at	THI73	YLR004C	Putative plasma membrane permease proposed to be involved in carboxylic acid uptake and repressed by thiamine; substrate of Dbf2p/Mob1p kinase; transcription is altered if mitochondrial dysfunction occurs	3.526948	1.81842	up
1769792_at	DSD1	YGL196W	D-serine dehydratase (aka D-serine ammonia-lyase); converts D-serine to pyruvate and ammonia by a reaction dependent on pyridoxal 5'-phosphate and zinc; may play a role in D-serine detoxification; L-serine is not a substrate	3.521237	1.816083	up
1773291_at		YHL012W		3.516609	1.814185	up
1773235_at		YLR460C		3.501273	1.807879	up
1779243_at	CWC15	YDR163W	Non-essential protein involved in pre-mRNA splicing, component of a complex containing Cef1p; has similarity to S. pombe Cwf15p	3.500002	1.807356	up

1778021_at		YKR023W		3.498873	1.80689	up
1776644_at	GLG1	YKR058W	Self-glucosylating initiator of glycogen synthesis, also glucosylates n-dodecyl-beta-D-maltoside; similar to mammalian glycogenin	3.490932	1.803612	up
1774351_at	POS5	YPL188W	Mitochondrial NADH kinase, phosphorylates NADH; also phosphorylates NAD(+) with lower specificity; required for the response to oxidative stress	3.486195	1.801653	up
1771703_at	LYS20	YDL182W	Homocitrate synthase isozyme, catalyzes the condensation of acetyl-CoA and alpha-ketoglutarate to form homocitrate, which is the first step in the lysine biosynthesis pathway; highly similar to the other isozyme, Lys21p	3.485571	1.801395	up
1779202_at	MST27	YGL051W	Putative integral membrane protein, involved in vesicle formation; forms complex with Mst28p; member of DUP240 gene family; binds COPI and COPII vesicles	3.48129	1.799622	up
1771744_at	MRS4	YKR052C	Iron transporter that mediates Fe2+ transport across the inner mitochondrial membrane; mitochondrial carrier family member, similar to and functionally redundant with Mrs3p; active under low-iron conditions; may transport other cations	3.471521	1.795568	up
1774317_at	HSE1	YHL002W	Subunit of the endosomal Vps27p-Hse1p complex required for sorting of ubiquitinated membrane proteins into intralumenal vesicles prior to vacuolar degradation, as well as for recycling of Golgi proteins and formation of lumenal membranes	3.469583	1.794762	up
1778591_at	AGA1	YNR044W	Anchorage subunit of a-agglutinin of a-cells, highly O-glycosylated protein with N-terminal secretion signal and C-terminal signal for addition of GPI anchor to cell wall, linked to adhesion subunit Aga2p via two disulfide bonds	3.45828	1.790055	up
1776687_at	GAT1	YFL021W	Transcriptional activator of genes involved in nitrogen catabolite repression; contains a GATA-1-type zinc finger DNA-binding motif; activity and localization regulated by nitrogen limitation and Ure2p	3.456701	1.789396	up
1773952_at	ARG2	YJL071W	Acetylglutamate synthase (glutamate N-acetyltransferase), mitochondrial enzyme that catalyzes the first step in the biosynthesis of the arginine precursor ornithine; forms a complex with Arg5,6p	3.451122	1.787066	up
1773097_at	YPC1	YBR183W	Alkaline ceramidase that also has reverse (CoA-independent) ceramide synthase activity, catalyzes both breakdown and synthesis of phytoceramide; overexpression confers fumonisin B1 resistance	3.446208	1.78501	up
1771404_at	RTK1	YDL025C	Putative protein kinase, potentially phosphorylated by Cdc28p; interacts with ribosome biogenesis factors, Cka2, Gus1 and Arc1	3.443988	1.78408	up
1772224_at	RAV2	YDR202C	Subunit of RAVE (Rav1p, Rav2p, Skp1p), a complex that associates with the V1 domain of the vacuolar membrane (H+)-ATPase (V-ATPase) and promotes assembly and reassembly of the holoenzyme	3.432847	1.779406	up
1779649_at	RAD14	YMR201C	Protein that recognizes and binds damaged DNA during nucleotide excision repair; subunit of Nucleotide Excision Repair Factor 1 (NEF1); contains zinc finger motif; homolog of human XPA protein	3.429381	1.777948	up
1770319_at	MET10	YFR030W	Subunit alpha of assimilatory sulfite reductase, which converts sulfite into sulfide	3.420295	1.774121	up
1774546_at	SLX4	YLR135W	Endonuclease involved in processing DNA during recombination and repair; cleaves branched structures in a complex with Slx1p; involved in Rad1p/Rad10p-dependent removal of 3'-nonhomologous tails during DSBR via single-	3.417774	1.773057	up

			strand annealing			
1772443_at	PEP12	YOR036W	Target membrane receptor (t-SNARE) for vesicular intermediates traveling between the Golgi apparatus and the vacuole; controls entry of biosynthetic, endocytic, and retrograde traffic into the prevacuolar compartment; syntaxin	3.416039	1.772325	up
1778852_at	SMA1	YPL027W	Protein of unknown function involved in the assembly of the prospore membrane during sporulation; interacts with Spo14p	3.400237	1.765635	up
1772171_at		YLR152C		3.400196	1.765618	up
1779455_at	REC102	YLR329W	Protein involved in early stages of meiotic recombination; required for chromosome synapsis; forms a complex with Rec104p and Spo11p necessary during the initiation of recombination	3.398625	1.764951	up
1777536_at		YGR067C		3.393524	1.762784	up
1773664_at	RIM15	YFL033C	Glucose-repressible protein kinase involved in signal transduction during cell proliferation in response to nutrients, specifically the establishment of stationary phase; identified as a regulator of IME2; substrate of Pho80p-Pho85p kinase	3.391308	1.761842	up
1777621_at		YMR210W		3.39118	1.761787	up
1775095_at	FIG2	YCR089W	Cell wall adhesin, expressed specifically during mating; may be involved in maintenance of cell wall integrity during mating	3.383933	1.758701	up
1774523_at		YGR226C		3.364643	1.750453	up
1772642_at		YLR312C		3.360803	1.748806	up
1772907_at	SOL1	YNR034W	Protein with a possible role in tRNA export; shows similarity to 6-phosphogluconolactonase non-catalytic domains but does not exhibit this enzymatic activity; homologous to Sol2p, Sol3p, and Sol4p	3.351906	1.744982	up
1769900_at	ATG14	YBR128C	Autophagy-specific subunit of phosphatidylinositol 3-kinase complex I (with Vps34/15/30p); Atg14p targets complex I to the phagophore assembly site (PAS); required for localizing additional ATG proteins to the PAS; homolog of human Barkor	3.326752	1.734114	up
1772252_at		YOR019W		3.322428	1.732238	up
1776161_at	GGA1	YDR358W	Golgi-localized protein with homology to gamma-adaptin, interacts with and regulates Arf1p and Arf2p in a GTP-dependent manner in order to facilitate traffic through the late Golgi	3.315641	1.729288	up
1779583_at		YIL060W		3.310927	1.727235	up
1778805_at		YFL051C		3.308305	1.726092	up
1776544_at		YBL101W-C		3.306042	1.725105	up
1779142_x_at	PAU17	YLL025W	Protein of unknown function, member of the seripauperin multigene family encoded mainly in subtelomeric regions; YLL025W is not an essential gene	3.304803	1.724564	up
1770055_at	FKS3	YMR306W	Protein involved in spore wall assembly, has similarity to 1,3-beta-D-glucan synthase catalytic subunits Fks1p and Gsc2p; the authentic, non-tagged protein is detected in highly purified mitochondria in high-throughput studies	3.304549	1.724453	up
1778187_at		YKL222C		3.303019	1.723785	up
1777269_at	ISY1	YJR050W	Member of NineTeen Complex (NTC) that contains Prp19p and stabilizes U6 snRNA in catalytic forms of spliceosome containing U2, U5, and U6 snRNAs, interacts with Prp16p to modulate splicing fidelity; isy1 syf2 cells have defective spindles	3.301774	1.723241	up

1774288_at	CDC34	YDR054C	Ubiquitin-conjugating enzyme (E2) and catalytic subunit of SCF ubiquitin-protein ligase complex (together with Skp1p, Rbx1p, Cdc53p, and an F-box protein) that regulates cell cycle progression by targeting key substrates for degradation	3.295997	1.720715	up
1775988_at	NDL1	YLR254C	Homolog of nuclear distribution factor NudE, NUDEL; interacts with Pac1p and regulates dynein targeting to microtubule plus ends	3.292931	1.719372	up
1772414_at	YHC1	YLR298C	Component of the U1 snRNP complex required for pre-mRNA splicing; putative ortholog of human U1C protein, which is involved in formation of a complex between U1 snRNP and the pre-mRNA 5' splice site	3.290223	1.718185	up
1772087_at	MTC4	YBR255W	Protein of unknown function, required for normal growth rate at 15 degrees C; green fluorescent protein (GFP)-fusion protein localizes to the cytoplasm in a punctate pattern; mtc4 is synthetically sick with cdc13-1	3.290036	1.718103	up
1778585_at	GAT3	YLR013W	Protein containing GATA family zinc finger motifs	3.277226	1.712475	up
1769333_at	GRE1	YPL223C	Hydrophilin essential in dessication-rehydration process; stress induced (osmotic, ionic, oxidative, heat shock and heavy metals); regulated by the HOG pathway	3.275896	1.71189	up
1771944_at	PDR11	YIL013C	ATP-binding cassette (ABC) transporter, multidrug transporter involved in multiple drug resistance; mediates sterol uptake when sterol biosynthesis is compromisedregulated by Pdr1p; required for anaerobic growth	3.270125	1.709346	up
1773597_at	PIB1	YDR313C	RING-type ubiquitin ligase of the endosomal and vacuolar membranes, binds phosphatidylinositol(3)-phosphate; contains a FYVE finger domain	3.253789	1.702121	up
1777384_at	PRM6	YML047C	Pheromone-regulated protein, predicted to have 2 transmembrane segments; regulated by Ste12p during mating	3.248823	1.699917	up
1779467_at	FLO10	YKR102W	Member of the FLO family of cell wall flocculation proteins; not expressed in most lab strains; overproduction induces flocculation that can be inhibited by mannose, sucrose, or glucose; overproduction also promotes haploid invasive growth and diploid filamentous growth	3.248342	1.699704	up
1774584_at		YHR202W		3.244293	1.697904	up
1778877_at	PHO23	YNL097C	Probable component of the Rpd3 histone deacetylase complex, involved in transcriptional regulation of PHO5; affects termination of snoRNAs and cryptic unstable transcripts (CUTs); C-terminus has similarity to human candidate tumor suppressor p33(ING1) and its isoform ING3	3.242994	1.697326	up
1775381_at	REX3	YLR107W	RNA exonuclease; required for maturation of the RNA component of RNase MRP; functions redundantly with Rnh70p and Rex2p in processing of U5 snRNA and RNase P RNA; member of RNase D family of exonucleases	3.242013	1.69689	up
1774192_at	PSK2	YOL045W	One of two (see also PSK1) PAS domain containing S/T protein kinases; regulates sugar flux and translation in response to an unknown metabolite by phosphorylating Ugp1p and Gsy2p (sugar flux) and Caf20p, Tif11p and Sro9p (translation)	3.240364	1.696156	up
1772460_at		YJR111C		3.235609	1.694037	up
1778401_at	BUD13	YGL174W	Subunit of the RES complex, which is required for nuclear pre-mRNA retention and splicing; involved in bud-site selection; diploid mutants display a unipolar budding pattern instead of the wild-type bipolar pattern	3.232452	1.692629	up

1773293_at		YHR214C-E		3.228067	1.690671	up
1779155_at	SET3	YKR029C	Defining member of the SET3 histone deacetylase complex which is a meiosis-specific repressor of sporulation genes; necessary for efficient transcription by RNAPII; one of two yeast proteins that contains both SET and PHD domains	3.228064	1.690669	up
1779420_at	SMY1	YKL079W	Protein that interacts with Myo2p, proposed to be involved in exocytosis; N-terminal domain is related to the motor domain of kinesins	3.216472	1.685479	up
1770603_at	GUP2	YPL189W	Probable membrane protein with a possible role in proton symport of glycerol; member of the MBOAT family of putative membrane-bound O-acyltransferases; Gup1p homolog	3.214005	1.684372	up
1778304_at	UBX3	YDL091C	UBX (ubiquitin regulatory X) domain-containing protein that interacts with Cdc48p, green fluorescent protein (GFP)-fusion protein localizes to the cytoplasm in a punctate pattern	3.21118	1.683104	up
1779035_at	MTL1	YGR023W	Putative plasma membrane sensor, involved in cell integrity signaling and stress response during glucose starvation and oxidative stress; has structural and functional similarity to Mid2p	3.209845	1.682504	up
1773816_at	GPX2	YBR244W	Phospholipid hydroperoxide glutathione peroxidase induced by glucose starvation that protects cells from phospholipid hydroperoxides and nonphospholipid peroxides during oxidative stress	3.208738	1.682006	up
1776257_at		YGR250C		3.208177	1.681754	up
1777933_at	VAB2	YEL005C	Protein with a potential role in vacuolar function, as suggested by its ability to bind Vac8p; likely member of BLOC complex involved in endosomal cargo sorting; Vab2p-GFP-fusion localizes to cytoplasm in punctate patter	3.206627	1.681057	up
1776931_x_at	PAU18	YLL064C	Protein of unknown function, member of the seripauperin multigene family encoded mainly in subtelomeric regions; identical to Pau6p///Member of the seripauperin multigene family encoded mainly in subtelomeric regions, active during alcoholic fermentation, regulated by anaerobiosis, negatively regulated by oxygen, repressed by heme; identical to Pau18p	3.203095	1.679467	up
1772134_at	STE3	YKL178C	Receptor for a factor pheromone, couples to MAP kinase cascade to mediate pheromone response; transcribed in alpha cells and required for mating by alpha cells, ligand bound receptors endocytosed and recycled to the plasma membrane; GPCR	3.202656	1.679269	up
1779313_s_at		YEL075C		3.199881	1.678018	up
1770515_x_at	PAU4	YLR461W	Protein of unknown function, member of the seripauperin multigene family encoded mainly in subtelomeric regions; expression induced by low temperature and also by anaerobic conditions; induced during alcoholic fermentation///Member of the seripauperin multigene family encoded mainly in subtelomeric regions; active during alcoholic fermentation, regulated by anaerobiosis, negatively regulated by oxygen, repressed by heme	3.196494	1.67649	up
1780232_at		YMR111C		3.194913	1.675777	up
1779031_at	SER3	YER081W	3-phosphoglycerate dehydrogenase, catalyzes the first step in serine and glycine biosynthesis; isozyme of Ser33p	3.188982	1.673096	up
1777572_at	TFB1	YDR311W	Subunit of TFIIH and nucleotide excision repair factor 3 complexes, required for nucleotide excision repair, target for transcriptional activators	3.187393	1.672377	up
1770856_at		YDR090C		3.185138	1.671356	up
1778744_at	RTG3	YBL103C	Basic helix-loop-helix-leucine zipper (bHLH/Zip) transcription factor that forms a complex with	3.184675	1.671146	up

			another bHLH/Zip protein, Rtg1p, to activate the retrograde (RTG) and TOR pathways			
1769993_at	PCH2	YBR186W	Nucleolar component of the pachytene checkpoint, which prevents chromosome segregation when recombination and chromosome synapsis are defective; also represses meiotic interhomolog recombination in the rDNA	3.182457	1.670141	up
1778656_at	EMP46	YLR080W	Integral membrane component of endoplasmic reticulum-derived COPII-coated vesicles, which function in ER to Golgi transport	3.181961	1.669916	up
1779848_at	HDA2	YDR295C	Subunit of a possibly tetrameric trichostatin A-sensitive class II histone deacetylase complex containing an Hda1p homodimer and an Hda2p-Hda3p heterodimer; involved in telomere maintenance	3.181207	1.669574	up
1771073_at	DCG1	YIR030C	Protein of unknown function, expression is sensitive to nitrogen catabolite repression and regulated by Dal80p; contains transmembrane domain	3.176485	1.667431	up
1770640_at	POL4	YCR014C	DNA polymerase IV, undergoes pair-wise interactions with Dnl4p-Lif1p and Rad27p to mediate repair of DNA double-strand breaks by non-homologous end joining (NHEJ); homologous to mammalian DNA polymerase beta	3.161731	1.660714	up
1775341_at	GFD1	YMR255W	Coiled-coiled protein of unknown function, identified as a high-copy suppressor of a dbp5 mutation	3.160781	1.660281	up
1773823_at	MRPL24	YMR193W	Mitochondrial ribosomal protein of the large subunit; two mitochondrial ribosomal proteins, YmL14 and YmL24, have been assigned to the same gene	3.160225	1.660028	up
1777972_at		YGL081W		3.158808	1.65938	up
1776580_at	RAD34	YDR314C	Protein involved in nucleotide excision repair (NER); homologous to RAD4	3.150944	1.655784	up
1775118_at	SLU7	YDR088C	RNA splicing factor, required for ATP-independent portion of 2nd catalytic step of spliceosomal RNA splicing; interacts with Prp18p; contains zinc knuckle domain	3.149559	1.65515	up
1774000_s_at	HXT15	YDL245C	Protein of unknown function with similarity to hexose transporter family members, expression is induced by low levels of glucose and repressed by high levels of glucose///Protein of unknown function with similarity to hexose transporter family members, expression is repressed by high levels of glucose	3.147772	1.654331	up
1774028_at	STE11	YLR362W	Signal transducing MEK kinase involved in pheromone response and pseudohyphal/invasive growth pathways where it phosphorylates Ste7p, and the high osmolarity response pathway, via phosphorylation of Pbs2p; regulated by Ste20p and Ste50p	3.129342	1.645859	up
1773739_at	NVJ1	YHR195W	Nuclear envelope protein, anchored to the nuclear inner membrane, that interacts with the vacuolar membrane protein Vac8p to promote formation of nucleus-vacuole junctions during piecemeal microautophagy of the nucleus (PMN)	3.123111	1.642984	up
1774715_at	PFK27	YOL136C	6-phosphofructo-2-kinase, catalyzes synthesis of fructose-2,6-bisphosphate; inhibited by phosphoenolpyruvate and sn-glycerol 3-phosphate, expression induced by glucose and sucrose, transcriptional regulation involves protein kinase A	3.114312	1.638914	up
1774387_at	UBC11	YOR339C	Ubiquitin-conjugating enzyme most similar in sequence to Xenopus ubiquitin-conjugating enzyme E2-C, but not a true functional homolog of this E2; unlike E2-C, not required for the degradation of mitotic cyclin Clb2	3.11199	1.637837	up

1770913_at		YGR240C-A		3.109955	1.636894	up
1776166_at	SRY1	YKL218C	3-hydroxyaspartate dehydratase, deaminates L-threo-3-hydroxyaspartate to form oxaloacetate and ammonia; required in the presence of hydroxyaspartate; highly similar to mouse serine racemase (Srr) but has no serine racemase activity	3.108014	1.635993	up
1774560_at	SMF3	YLR034C	Putative divalent metal ion transporter involved in iron homeostasis; transcriptionally regulated by metal ions; member of the Nramp family of metal transport proteins	3.103514	1.633903	up
1778237_at	MUM3	YOR298W	Protein of unknown function involved in the organization of the outer spore wall layers; has similarity to the tafazzins superfamily of acyltransferases	3.10129	1.632869	up
1775392_at	YPI1	YFR003C	Regulatory subunit of the type I protein phosphatase (PP1) Glc7p; Glc7p participates in the regulation of a variety of metabolic processes including mitosis and glycogen metabolism; in vitro evidence suggests Ypi1p is an inhibitor of Glc7p while in vivo evidence suggests it is an activator; overproduction causes decreased cellular content of glycogen; partial depletion causes lithium sensitivity, while overproduction confers lithium-tolerance	3.097017	1.630879	up
1779066_at	NFT1	YKR103W	Putative transporter of the multidrug resistance-associated protein (MRP) subfamily; adjacent ORFs YKR103W and YKR104W are merged in different strain backgrounds.	3.091327	1.628226	up
1769442_at	VPS3	YDR495C	Component of CORVET tethering complex; cytoplasmic protein required for the sorting and processing of soluble vacuolar proteins, acidification of the vacuolar lumen, and assembly of the vacuolar H+-ATPase	3.090036	1.627624	up
1772778_at	ATG8	YBL078C	Component of autophagosomes and Cvt vesicles; undergoes conjugation to phosphatidylethanolamine (PE); Atg8p-PE is anchored to membranes, is involved in phagophore expansion, and may mediate membrane fusion during autophagosome formation	3.089136	1.627204	up
1771392_at	FRE1	YLR214W	Ferric reductase and cupric reductase, reduces siderophore-bound iron and oxidized copper prior to uptake by transporters; expression induced by low copper and iron levels	3.084817	1.625185	up
1776550_at	HSP42	YDR171W	Small heat shock protein (sHSP) with chaperone activity; forms barrel-shaped oligomers that suppress unfolded protein aggregation; involved in cytoskeleton reorganization after heat shock	3.081272	1.623526	up
1775710_at	ATG32	YIL146C	Mitochondrial outer membrane protein required to initiate mitophagy; recruits the autophagy adaptor protein Atg11p and the ubiquitin-like protein Atg8p to the mitochondrial surface to initiate mitophagy, the selective vacuolar degradation of mitochondria in response to starvation; can promote pexophagy when placed ectopically in the peroxisomal membrane	3.079093	1.622505	up
1776044_at	RCK1	YGL158W	Protein kinase involved in the response to oxidative stress; identified as suppressor of S. pombe cell cycle checkpoint mutations	3.077937	1.621964	up
1779476_at	GLC8	YMR311C	Regulatory subunit of protein phosphatase 1 (Glc7p), involved in glycogen metabolism and chromosome segregation; proposed to regulate Glc7p activity via conformational alteration; ortholog of the mammalian protein phosphatase inhibitor 2	3.075964	1.621038	up
1774888_at		YJL045W		3.073625	1.619941	up

1772528_at		YAL064W		3.073575	1.619918	up
1777812_at	SNU23	YDL098C	Component of U4/U6.U5 snRNP involved in mRNA splicing via spliceosome	3.066933	1.616797	up
1779213_at	IRC6	YFR043C	Putative protein of unknown function; null mutant displays increased levels of spontaneous Rad52p foci	3.066837	1.616752	up
1771757_at	YAT2	YER024W	Carnitine acetyltransferase; has similarity to Yat1p, which is a carnitine acetyltransferase associated with the mitochondrial outer membrane	3.062975	1.614934	up
1775158_at		YDL241W		3.060934	1.613972	up
1779057_at	UIP4	YPL186C	Protein that interacts with Ulp1p, a Ubl (ubiquitin-like protein)-specific protease for Smt3p protein conjugates; detected in a phosphorylated state in the mitochondrial outer membrane; also detected in ER and nuclear envelope	3.055898	1.611597	up
1776923_at	ISU1	YPL135W	Conserved protein of the mitochondrial matrix, performs a scaffolding function during assembly of iron-sulfur clusters, interacts physically and functionally with yeast frataxin (Yfh1p); isu1 isu2 double mutant is inviable	3.055018	1.611181	up
1779515_at	KRE28	YDR532C	Subunit of a kinetochore-microtubule binding complex with Spc105p that bridges centromeric heterochromatin and kinetochore MAPs and motors, and is also required for sister chromatid bi-orientation and kinetochore binding of SAC components	3.054436	1.610906	up
1778729_at		YAR029W		3.05373	1.610573	up
1773278_at	ARN1	YHL040C	Transporter, member of the ARN family of transporters that specifically recognize siderophore-iron chelates; responsible for uptake of iron bound to ferrirubin, ferrirhodin, and related siderophores	3.053219	1.610331	up
1780053_at	ICY1	YMR195W	Protein of unknown function, required for viability in rich media of cells lacking mitochondrial DNA; mutants have an invasive growth defect with elongated morphology; induced by amino acid starvation	3.052877	1.610169	up
1777017_at		YGR237C		3.042517	1.605265	up
1772066_at		YHL042W		3.035177	1.60178	up
1772517_at	MSN4	YKL062W	Transcriptional activator related to Msn2p; activated in stress conditions, which results in translocation from the cytoplasm to the nucleus; binds DNA at stress response elements of responsive genes, inducing gene expression	3.031973	1.600257	up
1775676_at	UPF3	YGR072W	Component of the nonsense-mediated mRNA decay (NMD) pathway, along with Nam7p and Nmd2p; involved in decay of mRNA containing nonsense codons; involved in telomere maintenance	3.031682	1.600118	up
1776673_at	NCE103	YNL036W	Carbonic anhydrase; metalloenzyme that catalyzes CO2 hydration to bicarbonate, which is an important metabolic substrate, and protons; not expressed under conditions of high CO2, such as inside a growing colony, but transcription is induced in response to low CO2 levels, such as on the colony surface in ambient air; poorly transcribed under aerobic conditions and at an undetectable level under anaerobic conditions; mutation affects non-classical protein export pathway	3.024939	1.596906	up
1779319_at	SPP381	YBR152W	mRNA splicing factor, component of U4/U6.U5 tri-snRNP; interacts genetically and physically with Prp38p	3.023361	1.596153	up
1780131_at		YFL041W-A		3.016004	1.592639	up

1779981_at	NUT2	YPR168W	Subunit of the RNA polymerase II mediator complex; associates with core polymerase subunits to form the RNA polymerase II holoenzyme; required for transcriptional activation and has a role in basal transcription	3.011166	1.590322	up
1772485_at	SLK19	YOR195W	Kinetochore-associated protein required for normal segregation of chromosomes in meiosis and mitosis; component of the FEAR regulatory network, which promotes Cdc14p release from the nucleolus during anaphase; potential Cdc28p substrate	3.0093	1.589428	up
1772622_at	SIT1	YEL065W	Ferrioxamine B transporter, member of the ARN family of transporters that specifically recognize siderophore-iron chelates; transcription is induced during iron deprivation and diauxic shift; potentially phosphorylated by Cdc28p	3.009252	1.589405	up
1775466_at		YNL018C		3.001245	1.585561	up
1769571_at	PRP3	YDR473C	Splicing factor, component of the U4/U6-U5 snRNP complex	2.996616	1.583334	up
1776772_at	GTO3	YMR251W	Omega class glutathione transferase; putative cytosolic localization	2.990618	1.580444	up
1775740_at		YDL206W		2.985491	1.577968	up
1779789_at	PDR5	YOR153W	Plasma membrane ATP-binding cassette (ABC) transporter, multidrug transporter actively regulated by Pdr1p; also involved in steroid transport, cation resistance, and cellular detoxification during exponential growth	2.982803	1.576669	up
1769322_s_at		YLR154C-H		2.980677	1.57564	up
1773831_at		YMR114C		2.978854	1.574757	up
1770795_at		YKL162C		2.972059	1.571463	up
1776528_at	GPM2	YDL021W	Homolog of Gpm1p phosphoglycerate mutase, which converts 3-phosphoglycerate to 2-phosphoglycerate in glycolysis; may be non-functional derivative of a gene duplication event	2.97111	1.571002	up
1771344_at	FIT1	YDR534C	Mannoprotein that is incorporated into the cell wall via a glycosylphosphatidylinositol (GPI) anchor, involved in the retention of siderophore-iron in the cell wall	2.964813	1.567941	up
1775207_at	ASF2	YDL197C	Anti-silencing protein that causes derepression of silent loci when overexpressed	2.952108	1.561745	up
1774181_at	SWC4	YGR002C	Component of the Swr1p complex that incorporates Htz1p into chromatin; component of the NuA4 histone acetyltransferase complex	2.951968	1.561677	up
1771184_at	VPS64	YDR200C	Protein required for cytoplasm to vacuole targeting of proteins; forms a complex with Far3p and Far7p to Far11p involved in recovery from pheromone-induced cell cycle arrest; mutant has increased aneuploidy tolerance	2.948595	1.560028	up
1777753_at	RGI1	YER067W	Protein of unknown function involved in energy metabolism under respiratory conditions; protein abundance is increased upon intracellular iron depletion	2.940999	1.556306	up
1779133_at	KEL2	YGR238C	Protein that functions in a complex with Kel1p to negatively regulate mitotic exit, interacts with Tem1p and Lte1p; localizes to regions of polarized growth; potential Cdc28p substrate	2.931514	1.551646	up
1776244_at	DGA1	YOR245C	Diacylglycerol acyltransferase, catalyzes the terminal step of triacylglycerol (TAG) formation, acylates diacylglycerol using acyl-CoA as an acyl donor, localized to lipid particles	2.93108	1.551432	up
1774946_s_at		YAR064W		2.927419	1.549629	up
1772198_at		YCR102C		2.918601	1.545277	up

1777721_at	FMS1	YMR020W	Polyamine oxidase, converts spermine to spermidine, which is required for the essential hypusination modification of translation factor eIF-5A; also involved in pantothenic acid biosynthesis	2.918062	1.545011	up
1773653_at	STP1	YDR463W	Transcription factor, undergoes proteolytic processing by SPS (Ssy1p-Ptr3p-Ssy5p)-sensor component Ssy5p in response to extracellular amino acids; activates transcription of amino acid permease genes and may have a role in tRNA processing	2.917255	1.544612	up
1779560_at	PEX18	YHR160C	Peroxin required for targeting of peroxisomal matrix proteins containing PTS2; interacts with Pex7p; partially redundant with Pex21p	2.912206	1.542112	up
1776745_at	CDC7	YDL017W	DDK (Dbf4-dependent kinase) catalytic subunit required for origin firing and replication fork progression in mitotic S phase through phosphorylation of Mcm2-7p complexes and Cdc45p; kinase activity correlates with cyclical DBF4 expression; required for pre-meiotic DNA replication, meiotic DSB formation, recruitment of the monopolin complex to kinetochores during meiosis I and as a gene-specific regulator of the meiosis-specific transcription factor Ndt80p	2.910197	1.541117	up
1778191_at		YGR042W		2.908579	1.540315	up
1776428_at	SKP2	YNL311C	F-box protein of unknown function predicted to be part of an SCF ubiquitin protease complex; involved in regulating protein levels of sulfur metabolism enzymes; may interact with ribosomes, based on co-purification experiments	2.904128	1.538105	up
1779096_at		YCL021W-A		2.904092	1.538087	up
1780085_at	FAA2	YER015W	Medium chain fatty acyl-CoA synthetase, activates imported fatty acids; accepts a wide range of fatty acid chain lengths with a preference for medium chains, C9:0-C13:0; localized to the peroxisome	2.901208	1.536654	up
1779494_at	HXT10	YFL011W	Putative hexose transporter, expressed at low levels and expression is repressed by glucose	2.900138	1.536122	up
1769726_at	SLF1	YDR515W	RNA binding protein that associates with polysomes; proposed to be involved in regulating mRNA translation; involved in the copper-dependent mineralization of copper sulfide complexes on cell surface in cells cultured in copper salts	2.896058	1.534091	up
1776434_at	ECM13	YBL043W	Non-essential protein of unknown function; induced by treatment with 8-methoxypsoralen and UVA irradiation	2.894145	1.533137	up
1778536_at	SPC72	YAL047C	Component of the cytoplasmic Tub4p (gamma-tubulin) complex, binds spindle pole bodies and links them to microtubules; has roles in astral microtubule formation and stabilization	2.887832	1.529987	up
1777758_at	BSD2	YBR290W	Heavy metal ion homeostasis protein, facilitates trafficking of Smf1p and Smf2p metal transporters to the vacuole where they are degraded, controls metal ion transport, prevents metal hyperaccumulation, functions in copper detoxification	2.885327	1.528735	up
1775781_at	MCH4	YOL119C	Protein with similarity to mammalian monocarboxylate permeases, which are involved in transport of monocarboxylic acids across the plasma membrane; mutant is not deficient in monocarboxylate transport	2.884626	1.528384	up
1777211_at	RFX1	YLR176C	Major transcriptional repressor of DNA-damage-regulated genes, recruits repressors Tup1p and Cyc8p to their promoters; involved in DNA damage and replication checkpoint pathway; similar to a family of mammalian DNA binding	2.884156	1.528149	up

			RFX1-4 proteins			
1776845_at	SNA4	YDL123W	Protein of unknown function, localized to the vacuolar outer membrane; predicted to be palmitoylated	2.882741	1.527441	up
1775389_at	CRZ1	YNL027W	Transcription factor that activates transcription of genes involved in stress response; nuclear localization is positively regulated by calcineurin-mediated dephosphorylation	2.88121	1.526675	up
1771870_at	LCL3	YGL085W	Putative protein of unknown function; mutant has long chronological lifespan; has homology to Staphylococcus aureus nuclease; GFP-fusion protein localizes to mitochondria; is induced in response to the DNA-damaging agent MMS	2.879706	1.525921	up
1778335_at	ADI1	YMR009W	Acireductone dioxygenease involved in the methionine salvage pathway; ortholog of human MTCBP-1; transcribed with YMR010W and regulated post-transcriptionally by RNase III (Rnt1p) cleavage; ADI1 mRNA is induced in heat shock conditions	2.878892	1.525514	up
1774877_at	MED2	YDL005C	Subunit of the RNA polymerase II mediator complex; associates with core polymerase subunits to form the RNA polymerase II holoenzyme; essential for transcriptional regulation	2.876814	1.524472	up
1774887_at	SNC2	YOR327C	Vesicle membrane receptor protein (v-SNARE) involved in the fusion between Golgi-derived secretory vesicles with the plasma membrane; member of the synaptobrevin/VAMP family of R-type v-SNARE proteins	2.871863	1.521987	up
1775659_at	UFE1	YOR075W	t-SNARE required for retrograde vesicular traffic and homotypic ER membrane fusion; forms a complex with the SNAREs Sec22p, Sec20p and Use1p to mediate fusion of Golgi-derived vesicles at the ER	2.869999	1.521051	up
1772955_at		YBR062C		2.868825	1.52046	up
1773234_at		YER079W		2.867875	1.519982	up
1770949_at	MCM1	YMR043W	Transcription factor involved in cell-type-specific transcription and pheromone response; plays a central role in the formation of both repressor and activator complexes	2.867269	1.519677	up
1778859_at	VPS41	YDR080W	Vacuolar membrane protein that is a subunit of the homotypic vacuole fusion and vacuole protein sorting (HOPS) complex; essential for membrane docking and fusion at the Golgi-to-endosome and endosome-to-vacuole stages of protein transport	2.864865	1.518467	up
1771348_at		YLR040C		2.86485	1.51846	up
1775401_at		YIL024C		2.863942	1.518003	up
1775599_at	NPR1	YNL183C	Protein kinase that stabilizes several plasma membrane amino acid transporters by antagonizing their ubiquitin-mediated degradation; phosphorylates Aly2p; negatively regulates Ldb19p-mediated endocytosis through phosphorylation of Ldb19p, which prevents its association with the plasma membrane; Npr1p activity is negatively regulated via phosphorylation by the TOR complex	2.863387	1.517723	up
1779265_at		YBR071W		2.862107	1.517077	up
1777711_at	ATG11	YPR049C	Adapter protein for pexophagy and the cytoplasm-to-vacuole targeting (Cvt) pathway; directs receptor-bound cargo to the phagophore assembly site (PAS) for packaging into vesicles; required for recruiting other proteins to the (PAS)	2.849923	1.510923	up
1773464_at	IRC23	YOR044W	Putative protein of unknown function; green fluorescent protein (GFP)-fusion localizes to the	2.849	1.510456	up

			ER; null mutant displays increased levels of spontaneous Rad52p foci			
1772935_at	NBL1	YHR199C-A	Subunit of the conserved chromosomal passenger complex (CPC; Ipl1p-Sli15p-Bir1p-Nbl1p), which regulates mitotic chromosome segregation; not required for the kinase activity of the complex; mediates the interaction of Sli15p and Bir1p	2.848786	1.510347	up
1779572_at	VPS71	YML041C	Nucleosome-binding component of the SWR1 complex, which exchanges histone variant H2AZ (Htz1p) for chromatin-bound histone H2A; required for vacuolar protein sorting	2.845945	1.508908	up
1770073_s_at	SNO4	YMR322C	Possible chaperone and cysteine protease with similarity to E. coli Hsp31 and S. cerevisiae Hsp31p, Hsp33p, and Sno4p; member of the DJ-1/ThiJ/PfpI superfamily, which includes human DJ-1 involved in Parkinson's diseasePossible chaperone and cysteine protease, similar to bacterial Hsp31 and yeast Hsp31p, Hsp32p, and Hsp33p; DJ-1/ThiJ/PfpI superfamily member; predicted involvement in pyridoxine metabolism; induced by mild heat stress and copper deprivation	2.843751	1.507795	up
1776423_at	CTK1	YKL139W	Catalytic (alpha) subunit of C-terminal domain kinase I (CTDK-I); phosphorylates both RNA pol II subunit Rpo21p to affect transcription and pre-mRNA 3' end processing, and ribosomal protein Rps2p to increase translational fidelity; similar to the Drosophila dCDK12 and human CDK12 and probably CDK13	2.84341	1.507622	up
1772962_at	FAR7	YFR008W	Protein involved in recovery from cell cycle arrest in response to pheromone, in a Far1p-independent pathway; interacts with Far3p, Far8p, Far9p, Far10p, and Far11p	2.834142	1.502912	up
1772356_at	TDA8	YAL064C-A	Putative protein of unknown function; null mutant is sensitive to expression of the top1-T722A allele; not an essential gene	2.83377	1.502723	up
1774491_s_at	MAL32	YBR299W	Maltase (alpha-D-glucosidase), inducible protein involved in maltose catabolism; encoded in the MAL1 complex locus; hydrolyzes the disaccharides maltose, turanose, maltotriose, and sucrose///Maltase (alpha-D-glucosidase), inducible protein involved in maltose catabolism; encoded in the MAL3 complex locus; functional in genomic reference strain S288C; hydrolyzes the disaccharides maltose, turanose, maltotriose, and sucrose	2.833175	1.50242	up
1773469_at		YDL186W		2.831917	1.501779	up
1779718_at	RNY1	YPL123C	Vacuolar RNase of the T(2) family, relocalizes to the cytosol where it cleaves tRNAs upon oxidative or stationary phase stress; promotes apoptosis under stress conditions and this function is independent of its catalytic activity	2.831033	1.501329	up
1779955_at	ATG21	YPL100W	Phosphoinositide binding protein required for vesicle formation in the cytoplasm-to-vacuole targeting (Cvt) pathway; binds both phosphatidylinositol (3,5)-bisphosphate and phosphatidylinositol 3-phosphate; WD-40 repeat protein	2.829957	1.50078	up
1777510_at		YER130C		2.828414	1.499993	up
1772386_at	NTG1	YAL015C	DNA N-glycosylase and apurinic/apyrimidinic (AP) lyase involved in base excision repair; acts in both nucleus and mitochondrion; creates a double-strand break at mtDNA origins that stimulates replication in response to oxidative stress	2.827924	1.499744	up
1774258_at	CDC27	YBL084C	Subunit of the Anaphase-Promoting Complex/Cyclosome (APC/C), which is a	2.823664	1.497568	up

			ubiquitin-protein ligase required for degradation of anaphase inhibitors, including mitotic cyclins, during the metaphase/anaphase transition			
1774339_at	RRT8	YOL048C	Putative protein of unknown function; identified in a screen for mutants with increased levels of rDNA transcription; green fluorescent protein (GFP)-fusion protein localizes to lipid particles	2.822921	1.497189	up
1773204_at	SAW1	YAL027W	Protein involved in Rad1p/Rad10p-dependent removal of 3'-nonhomologous tails during double-strand break repair via single-strand annealing; green fluorescent protein (GFP)-fusion protein localizes to the nucleus	2.822115	1.496777	up
1780130_at	PPM1	YDR435C	Carboxyl methyltransferase, methylates the C terminus of the protein phosphatase 2A catalytic subunit (Pph21p or Pph22p), which is important for complex formation with regulatory subunits	2.817052	1.494186	up
1775769_at		YAL065C		2.815619	1.493452	up
1770300_at	CNM67	YNL225C	Component of the spindle pole body outer plaque; required for spindle orientation and mitotic nuclear migration	2.812483	1.491844	up
1777677_at	YRB2	YIL063C	Protein of unknown function involved in nuclear processes of the Ran-GTPase cycle; involved in nuclear protein export; contains Ran Binding Domain and FxFG repeats; interacts with Srm1p, GTP-Gsp1p, Rna1p and Crm1p; is not essential	2.80936	1.490242	up
1774895_at	PET130	YJL023C	Protein required for respiratory growth; the authentic, non-tagged protein is detected in highly purified mitochondria in high-throughput studies	2.808911	1.490011	up
1778479_at	CHA1	YCL064C	Catabolic L-serine (L-threonine) deaminase, catalyzes the degradation of both L-serine and L-threonine; required to use serine or threonine as the sole nitrogen source, transcriptionally induced by serine and threonine	2.807443	1.489257	up
1776402_at		YMR181C		2.807185	1.489124	up
1778682_at	ASE1	YOR058C	Mitotic spindle midzone localized microtubule-associated protein (MAP) family member; required for spindle elongation and stabilization; undergoes cell cycle-regulated degradation by anaphase promoting complex; potential Cdc28p substrate	2.805589	1.488304	up
1775688_at		YDL183C		2.803744	1.487355	up
1776173_at	CSR2	YPR030W	Nuclear protein proposed to regulate utilization of nonfermentable carbon sources and endocytosis of plasma membrane proteins; overproduction suppresses chs5 spa2 lethality at high temp; ubiquitinated by Rsp5p, deubiquitinated by Ubp2p	2.79108	1.480824	up
1774742_at	TDA6	YPR157W	Putative protein of unknown function; induced by treatment with 8-methoxypsoralen and UVA irradiation; null mutant is sensitive to expression of the top1-T722A allele	2.789963	1.480246	up
1779817_at	BRR1	YPR057W	snRNP protein component of spliceosomal snRNPs, required for pre-mRNA splicing and snRNP biogenesis; in null mutant newly-synthesized snRNAs are destabilized and 3'-end processing is slowed	2.781071	1.475641	up
1776232_at	HVG1	YER039C	Protein of unknown function, has homology to Vrg4p	2.777791	1.473938	up
1779659_at		YBR056W-A		2.777505	1.47379	up
1777486_at	RIB5	YBR256C	Riboflavin synthase; catalyzes the last step of the riboflavin biosynthesis pathway	2.770578	1.470187	up
1774870_at	JHD1	YER051W	JmjC domain family histone demethylase specific for H3-K36, similar to proteins found in human, mouse, drosophila, X. laevis, C. elegans, and S. pombe	2.767566	1.468617	up
1771914_at	STB6	YKL072W	Protein that binds Sin3p in a two-hybrid assay	2.767465	1.468565	up

1774340_at	FBP26	YJL155C	Fructose-2,6-bisphosphatase, required for glucose metabolism	2.763097	1.466286	up
1776297_at	AME1	YBR211C	Essential kinetochore protein associated with microtubules and spindle pole bodies; component of the kinetochore sub-complex COMA (Ctf19p, Okp1p, Mcm21p, Ame1p); involved in spindle checkpoint maintenance	2.762347	1.465895	up
1770122_at	RFM1	YOR279C	DNA-binding protein required for vegetative repression of middle sporulation genes; specificity factor that directs the Hst1p histone deacetylase to some of the promoters regulated by Sum1p; involved in telomere maintenance	2.762168	1.465801	up
1774873_at	HEL1	YKR017C	Putative protein of unknown function; contains a RING finger motif	2.760247	1.464798	up
1773983_at	GMC2	YLR445W	Protein involved in meiotic progression; mutants are delayed in meiotic nuclear division and are defective in synaptonemal complex assembly; transcription is regulated by Ume6p and induced in response to alpha factor	2.76004	1.464689	up
1779950_at	AMD2	YDR242W	Putative amidase	2.759577	1.464447	up
1778878_s_at	IMD2	YAR073W	Inosine monophosphate dehydrogenase, catalyzes the rate-limiting step in GTP biosynthesis, expression is induced by mycophenolic acid resulting in resistance to the drug, expression is repressed by nutrient limitatio	2.750098	1.459483	up
1770758_at	PEX14	YGL153W	Peroxisomal membrane peroxin that is a central component of the peroxisomal protein import machinery; interacts with both PTS1 (Pex5p) and PTS2 (Pex7p), peroxisomal matrix protein signal recognition factors and membrane receptor Pex13p	2.744781	1.456691	up
1778414_at	BRF1	YGR246C	TFIIIB B-related factor, one of three subunits of RNA polymerase III transcription initiation factor TFIIIB, binds TFIIIC and TBP and recruits RNA pol III to promoters, amino-terminal half is homologous to TFIIB	2.744128	1.456348	up
1774146_at	EAF7	YNL136W	Subunit of the NuA4 histone acetyltransferase complex, which acetylates the N-terminal tails of histones H4 and H2A	2.741344	1.454883	up
1770150_at	YAF9	YNL107W	Subunit of both the NuA4 histone H4 acetyltransferase complex and the SWR1 complex, may function to antagonize silencing near telomeres; interacts directly with Swc4p, has homology to human leukemogenic protein AF9, contains a YEATS domain	2.739561	1.453945	up
1772974_at	IKS1	YJL057C	Protein kinase of unknown cellular role; putative serine/threonine kinase; expression is induced during mild heat stress; deletion mutants are hypersensitive to copper sulphate and resistant to sorbate; interacts with an N-terminal fragment of Sst2p	2.73771	1.45297	up
1773684_at	GSM1	YJL103C	Putative zinc cluster protein of unknown function; proposed to be involved in the regulation of energy metabolism, based on patterns of expression and sequence analysis	2.736486	1.452324	up
1777919_at	NNK1	YKL171W	Protein kinase; implicated in proteasome function; interacts with TORC1, Ure2 and Gdh2; overexpression leads to hypersensitivity to rapamycin and nuclear accumulation of Gln3; epitope-tagged protein localizes to the cytoplasm	2.735658	1.451888	up
1776312_at	MSH4	YFL003C	Protein involved in meiotic recombination, required for normal levels of crossing over, colocalizes with Zip2p to discrete foci on meiotic chromosomes, has homology to bacterial MutS protein	2.735619	1.451867	up
1778746_at	CST6	YIL036W	Basic leucine zipper (bZIP) transcription factor, in ATF/CREB family; mediates transcriptional activation of NCE103 (encoding carbonic	2.735051	1.451568	up

			anhydrase) in response to low CO2 levels such as in the ambient air; proposed to be a regulator of oleate responsive genes; involved in utilization of non-optimal carbon sources and chromosome stability			
1773685_at	SSQ1	YLR369W	Mitochondrial hsp70-type molecular chaperone, required for assembly of iron/sulfur clusters into proteins at a step after cluster synthesis, and for maturation of Yfh1p, which is a homolog of human frataxin implicated in Friedreich's ataxia	2.734404	1.451226	up
1774764_at	NRK1	YNL129W	Nicotinamide riboside kinase, catalyzes the phosphorylation of nicotinamide riboside and nicotinic acid riboside in salvage pathways for NAD+ biosynthesis	2.72519	1.446357	up
1770376_at	CAF16	YFL028C	Part of evolutionarily-conserved CCR4-NOT regulatory complex; contains single ABC-type ATPase domain but no transmembrane domain; interacts with several subunits of Mediator	2.722332	1.444843	up
1771999_at	AIM39	YOL053W	Putative protein of unknown function; null mutant displays elevated frequency of mitochondrial genome loss	2.722306	1.444829	up
1771729_at		YDR186C		2.720358	1.443797	up
1776679_at	ARN2	YHL047C	Transporter, member of the ARN family of transporters that specifically recognize siderophore-iron chelates; responsible for uptake of iron bound to the siderophore triacetylfusarinine C	2.718525	1.442824	up
1772122_s_at		YFL064C	Helicase encoded by the Y' element of subtelomeric regions, highly expressed in the mutants lacking the telomerase component TLC1; potentially phosphorylated by Cdc28p	2.715715	1.441332	up
1779853_at	PCL5	YHR071W	Cyclin, interacts with and phosphorylated by Pho85p cyclin-dependent kinase (Cdk), induced by Gcn4p at level of transcription, specifically required for Gcn4p degradation, may be sensor of cellular protein biosynthetic capacity	2.71533	1.441127	up
1769844_at	FMP32	YFL046W	Putative protein of unknown function; the authentic, non-tagged protein is detected in highly purified mitochondria in high-throughput studies	2.71476	1.440825	up
1769403_at	MIA40	YKL195W	Essential protein of the mitochondrial intermembrane space (IMS); promotes retention of newly imported proteins; may do so by stabilizing client protein folding as part of a disulfide relay system or transferring metal to client proteins	2.714116	1.440482	up
1779454_at	KHA1	YJL094C	Putative K+/H+ antiporter with a probable role in intracellular cation homeostasis, localized to Golgi vesicles and detected in highly purified mitochondria in high-throughput studies	2.712761	1.439762	up
1775507_at	SRL2	YLR082C	Protein of unknown function; overexpression suppresses the lethality caused by a rad53 null mutation	2.710022	1.438304	up
1778139_at	FYV6	YNL133C	Protein of unknown function, required for survival upon exposure to K1 killer toxin; proposed to regulate double-strand break repair via non-homologous end-joining	2.70933	1.437936	up
1773183_at	MUC1	YIR019C	GPI-anchored cell surface glycoprotein (flocculin); required for pseudohyphal formation, invasive growth, flocculation, and biofilms; transcriptionally regulated by the MAPK pathway (via Ste12p and Tec1p) and the cAMP pathway (via Flo8p); required for the formation of fibrous interconnections between cells in a colony of a wild S. cerevisiae strain	2.707017	1.436704	up
1770737_at	JNM1	YMR294W	Component of the yeast dynactin complex, consisting of Nip100p, Jnm1p, and Arp1p; required for proper nuclear migration and spindle partitioning during mitotic anaphase B	2.703694	1.434932	up

1780120_at	CRS5	YOR031W	Copper-binding metallothionein, required for wild-type copper resistance	2.703491	1.434824	up
1772029_at	RCO1	YMR075W	Essential subunit of the histone deacetylase Rpd3S complex; interacts with Eaf3p	2.698958	1.432403	up
1778278_at	RTG1	YOL067C	Transcription factor (bHLH) involved in interorganelle communication between mitochondria, peroxisomes, and nucleus	2.698943	1.432395	up
1778111_at	ATO3	YDR384C	Plasma membrane protein, regulation pattern suggests a possible role in export of ammonia from the cell; phosphorylated in mitochondria; member of the TC 9.B.33 YaaH family of putative transporters	2.696104	1.430876	up
1772597_at		YCR101C		2.695407	1.430503	up
1773094_at	SPT7	YBR081C	Subunit of the SAGA transcriptional regulatory complex, involved in proper assembly of the complex; also present as a C-terminally truncated form in the SLIK/SALSA transcriptional regulatory complex	2.691003	1.428144	up
1777525_at	RCN2	YOR220W	Protein of unknown function; green fluorescent protein (GFP)-fusion protein localizes to the cytoplasm and is induced in response to the DNA-damaging agent MMS; phosphorylated in response to alpha factor	2.690722	1.427993	up
1780036_at	UBC8	YEL012W	Ubiquitin-conjugating enzyme that negatively regulates gluconeogenesis by mediating the glucose-induced ubiquitination of fructose-1,6-bisphosphatase (FBPase); cytoplasmic enzyme that catalyzes the ubiquitination of histones in vitro	2.686225	1.42558	up
1776964_at	YPT35	YHR105W	Endosomal protein of unknown function that contains a phox (PX) homology domain and binds to both phosphatidylinositol-3-phosphate (PtdIns(3)P) and proteins involved in ER-Golgi or vesicular transport	2.683542	1.424139	up
1769544_at		YLR149C		2.675591	1.419858	up
1775424_at	EPL1	YFL024C	Subunit of NuA4, an essential histone H4/H2A acetyltransferase complex; conserved region at N-terminus is essential for interaction with the NPC (nucleosome core particle); homologous to Drosophila Enhancer of Polycomb	2.672247	1.418053	up
1774070_at	ADH2	YMR303C	Glucose-repressible alcohol dehydrogenase II, catalyzes the conversion of ethanol to acetaldehyde; involved in the production of certain carboxylate esters; regulated by ADR1	2.669838	1.416752	up
1775355_at	BST1	YFL025C	GPI inositol deacylase of the ER that negatively regulates COPII vesicle formation, prevents production of vesicles with defective subunits, required for proper discrimination between resident ER proteins and Golgi-bound cargo molecules	2.666513	1.414954	up
1771041_at		YOR152C		2.665908	1.414627	up
1769520_at	PER33	YLR064W	Protein that localizes to the endoplasmic reticulum with some nuclear pore complex association; deletion extends chronological lifespan; highly conserved across species, orthologous to human TMEM33 and paralogous to Pom33p	2.660084	1.411472	up
1771072_at	ATG16	YMR159C	Conserved protein that interacts with Atg12p-Atg5p conjugates to form Atg12p-Atg5p-Atg16p multimers, which localize to the pre-autophagosomal structure and are required for autophagy	2.659147	1.410964	up
1777366_at	PIG2	YIL045W	Putative type-1 protein phosphatase targeting subunit that tethers Glc7p type-1 protein phosphatase to Gsy2p glycogen synthase	2.658014	1.410349	up
1772756_at	ECL1	YGR146C	Protein of unknown function, affects chronological lifespan; induced by iron homeostasis transcription factor Aft2p; multicopy suppressor of temperature	2.654178	1.408265	up

			sensitive hsf1 mutant; induced by treatment with 8-methoxypsoralen and UVA irradiation			
1774122_at	FDH1	YOR388C	NAD(+)-dependent formate dehydrogenase, may protect cells from exogenous formate	2.650877	1.40647	up
1776447_at	YNG2	YHR090C	Subunit of NuA4, an essential histone acetyltransferase complex; positions Piccolo NuA4 for efficient acetylation of histone H4 or histone H2A; has similarity to the human tumor suppressor ING1 and its isoforms ING4 and ING5	2.649005	1.405451	up
1775546_at		YDL114W		2.646948	1.40433	up
1771712_at	RRT13	YER066W	Putative protein of unknown function; non-essential gene identified in a screen for mutants with decreased levels of rDNA transcription	2.641741	1.401489	up
1770412_at		YML007C-A		2.641073	1.401124	up
1777877_at	IMH1	YLR309C	Protein involved in vesicular transport, mediates transport between an endosomal compartment and the Golgi, contains a Golgi-localization (GRIP) domain that interacts with activated Arl1p-GTP to localize Imh1p to the Golgi	2.640939	1.401051	up
1773197_at		YDR034C-A		2.640693	1.400916	up
1771090_at	FRE2	YKL220C	Ferric reductase and cupric reductase, reduces siderophore-bound iron and oxidized copper prior to uptake by transporters; expression induced by low iron levels but not by low copper levels	2.640642	1.400888	up
1773096_at	GAL2	YLR081W	Galactose permease, required for utilization of galactose; also able to transport glucose	2.640257	1.400678	up
1769514_at	HAL5	YJL165C	Putative protein kinase; overexpression increases sodium and lithium tolerance, whereas gene disruption increases cation and low pH sensitivity and impairs potassium uptake, suggesting a role in regulation of Trk1p and/or Trk2p transporters	2.635594	1.398128	up
1777918_at	POG1	YIL122W	Nuclear chromatin-associated protein of unknown function; overexpression promotes recovery from pheromone induced arrest and suppresses the stress sensitivity caused by a mutation in the E3 ubiquitin ligase Rsp5p; binds upstream of BAR1 and cell cycle-related genes; potential Cdc28p substrate; SBF regulated	2.634766	1.397675	up
1772570_at	DAD2	YKR083C	Essential subunit of the Dam1 complex (aka DASH complex), couples kinetochores to the force produced by MT depolymerization thereby aiding in chromosome segregation; is transferred to the kinetochore prior to mitosis	2.632174	1.396255	up
1771940_at	PEX21	YGR239C	Peroxin required for targeting of peroxisomal matrix proteins containing PTS2; interacts with Pex7p; partially redundant with Pex18p	2.626905	1.393364	up
1777571_at	GTF1	YGR102C	Subunit of the trimeric GatFAB AmidoTransferase(AdT) complex; involved in the formation of Q-tRNAQ; transposon insertion mutant is salt sensitive and null mutant has growth defects; non-tagged protein is detected in purified mitochondria	2.623086	1.391265	up
1772104_at	YMR1	YJR110W	Phosphatidylinositol 3-phosphate (PI3P) phosphatase; involved in various protein sorting pathways, including CVT targeting and endosome to vacuole transport; has similarity to the conserved myotubularin dual specificity phosphatase family	2.618068	1.388503	up
1770071_at	THI4	YGR144W	Thiazole synthase, abundant protein involved in the formation of the thiazole moiety of thiamine during thiamine biosynthesis; acts more as a co-substrate rather than an enzyme by providing the sulphur source for thiazole formation; undergoes a single turnover only; required for mitochondrial genome stability in response to DNA damaging agents	2.614409	1.386485	up

1778697_at	ENT2	YLR206W	Epsin-like protein required for endocytosis and actin patch assembly and functionally redundant with Ent1p; contains clathrin-binding motif at C-terminus	2.613261	1.385851	up
1773559_at	YKU70	YMR284W	Subunit of the telomeric Ku complex (Yku70p-Yku80p), involved in telomere length maintenance, structure and telomere position effect; relocates to sites of double-strand cleavage to promote nonhomologous end joining during DSB repair	2.610973	1.384587	up
1775391_s_at	IMA3	YIL172C	Alpha-glucosidase with broad substrate specificity for alpha-1,4- and alpha-1,6-glucosides; member of IMA isomaltase family; not required for isomaltose utilization, but Ima3p overexpression allows the ima1 null mutant to grow on isomaltose	2.607676	1.382765	up
1779448_at	MIH1	YMR036C	Protein tyrosine phosphatase involved in cell cycle control; regulates the phosphorylation state of Cdc28p; homolog of S. pombe cdc25	2.603776	1.380605	up
1771826_at	NTG2	YOL043C	DNA N-glycosylase and apurinic/apyrimidinic (AP) lyase involved in base excision repair, localizes to the nucleus; sumoylated	2.597198	1.376956	up
1771794_at	ECM10	YEL030W	Heat shock protein of the Hsp70 family, localized in mitochondrial nucleoids, plays a role in protein translocation, interacts with Mge1p in an ATP-dependent manner; overexpression induces extensive mitochondrial DNA aggregations	2.596723	1.376692	up
1777325_at	ECM11	YDR446W	Non-essential protein apparently involved in meiosis, GFP fusion protein is present in discrete clusters in the nucleus throughout mitosis; may be involved in maintaining chromatin structure	2.594864	1.375659	up
1771081_at	ADK2	YER170W	Mitochondrial adenylate kinase, catalyzes the reversible synthesis of GTP and AMP from GDP and ADP; may serve as a back-up for synthesizing GTP or ADP depending on metabolic conditions; 3' sequence of ADK2 varies with strain background	2.593553	1.37493	up
1772626_at		YJR149W		2.591721	1.37391	up
1770323_at	RPN11	YFR004W	Metalloprotease subunit of the 19S regulatory particle of the 26S proteasome lid; couples the deubiquitination and degradation of proteasome substrates; involved, independent of catalytic activity, in fission of mitochondria and peroxisomes	2.591121	1.373576	up
1775024_at	BET4	YJL031C	Alpha subunit of Type II geranylgeranyltransferase required for vesicular transport between the endoplasmic reticulum and the Golgi; provides a membrane attachment moiety to Rab-like proteins Ypt1p and Sec4p	2.590783	1.373388	up
1774245_at		YFR012W-A		2.589369	1.372601	up
1771894_at	AIM23	YJL131C	Mitochondrial translation initiation factor 3 (IF3, mIF3); evolutionarily conserved; binds to E. coli ribosomes in vitro; null mutant displays severe respiratory growth defect and elevated frequency of mitochondrial genome loss	2.589129	1.372467	up
1780066_at	MUD1	YBR119W	U1 snRNP A protein, homolog of human U1-A; involved in nuclear mRNA splicing	2.586535	1.371021	up
1778537_at		YHR212W-A		2.586174	1.370819	up
1769808_at	EMI5	YOL071W	Subunit of succinate dehydrogenase, which couples succinate oxidation to ubiquinone reduction; required for FAD cofactor attachment to Sdh1p; mutations in human ortholog PGL2 are associated with neuroendocrine tumors (paraganglioma)	2.583749	1.369466	up
1772603_at	UBX4	YMR067C	UBX domain-containing protein that interacts with Cdc48p; involved in degradation of polyubiquitinated proteins via the ERAD (ER-associated degradation) pathway; modulates the Cdc48p-Nplp-Ufd1p AAA ATPase complex during	2.581026	1.367945	up

			its role in delivery of misfolded proteins to the proteasome			
1770088_at		YIL166C		2.578001	1.366253	up
1769979_at	STE50	YCL032W	Protein involved in mating response, invasive/filamentous growth, and osmotolerance, acts as an adaptor that links G protein-associated Cdc42p-Ste20p complex to the effector Ste11p to modulate signal transduction	2.570664	1.362141	up
1773837_at	UPS1	YLR193C	Mitochondrial protein involved in phospholipid metabolism; localizes to the intermembrane space; has a role in regulation of phospholipid metabolism by promoting conversion of phosphatidylethanolamine to phosphatidylcholine; null mutant has altered cardiolipin and phosphatidylethanolamine levels and defects in Mgm1p processing, integrity of mitochondrial inner membrane complexes, and mitochondrial morphology; ortholog of human PRELI	2.570256	1.361912	up
1773612_at	HSP82	YPL240C	Hsp90 chaperone required for pheromone signaling and negative regulation of Hsf1p; docks with Tom70p for mitochondrial preprotein delivery; promotes telomerase DNA binding and nucleotide addition; interacts with Cns1p, Cpr6p, Cpr7p, Sti1p	2.565017	1.358968	up
1776766_at	PRM7	YDL039C	Pheromone-regulated protein, predicted to have one transmembrane segment; promoter contains Gcn4p binding elements	2.564227	1.358524	up
1772032_at	DSN1	YIR010W	Essential component of the MIND kinetochore complex (Mtw1p Including Nnf1p-Nsl1p-Dsn1p) which joins kinetochore subunits contacting DNA to those contacting microtubules; important for chromosome segregation	2.56407	1.358436	up
1778375_at	TIS11	YLR136C	mRNA-binding protein expressed during iron starvation; binds to a sequence element in the 3'-untranslated regions of specific mRNAs to mediate their degradation; involved in iron homeostasis	2.560807	1.356598	up
1770203_at	STP2	YHR006W	Transcription factor, activated by proteolytic processing in response to signals from the SPS sensor system for external amino acids; activates transcription of amino acid permease genes	2.560531	1.356443	up
1773305_at	HMX1	YLR205C	ER localized heme oxygenase, involved in heme degradation during iron starvation and in the oxidative stress response; expression is regulated by AFT1 and oxidative stress; relocates to the perinuclear region in the presence of oxidants	2.555331	1.35351	up
1779014_at	GPI10	YGL142C	Integral membrane protein involved in glycosylphosphatidylinositol (GPI) anchor synthesis; putative alpha 1,2 mannosyltransferase required for addition of the third mannose onto the GPI core structure; human PIG-Bp is a functional homolog	2.553693	1.352585	up
1770891_at	PUS5	YLR165C	Pseudouridine synthase, catalyzes only the formation of pseudouridine (Psi)-2819 in mitochondrial 21S rRNA; not essential for viability	2.549913	1.350448	up
1772693_at	SWI1	YPL016W	Subunit of the SWI/SNF chromatin remodeling complex; regulates transcription by remodeling chromatin; required for transcription of many genes, including ADH1, ADH2, GAL1, HO, INO1 and SUC2; can form the prion [SWI+]; human homolog ARID1A is a candidate tumor suppressor gene in breast cancer	2.548052	1.349395	up
1778128_at	DUR3	YHL016C	Plasma membrane transporter for both urea and polyamines, expression is highly sensitive to nitrogen catabolite repression and induced by allophanate, the last intermediate of the allantoin degradative pathway	2.540496	1.34511	up

1778724_x_at		YLR466C-B		2.53856	1.34401	up
1776172_at		YBR072C-A		2.53837	1.343903	up
1774026_at	MUP1	YGR055W	High affinity methionine permease, integral membrane protein with 13 putative membrane-spanning regions; also involved in cysteine uptake	2.538031	1.34371	up
1780250_at	END3	YNL084C	EH domain-containing protein involved in endocytosis, actin cytoskeletal organization and cell wall morphogenesis; forms a complex with Sla1p and Pan1p	2.536297	1.342724	up
1774139_at	PEX15	YOL044W	Phosphorylated tail-anchored type II integral peroxisomal membrane protein required for peroxisome biogenesis, cells lacking Pex15p mislocalize peroxisomal matrix proteins to cytosol, overexpression results in impaired peroxisome assembly	2.536243	1.342693	up
1772959_at	MET8	YBR213W	Bifunctional dehydrogenase and ferrochelatase, involved in the biosynthesis of siroheme, a prosthetic group used by sulfite reductase; required for sulfate assimilation and methionine biosynthesis	2.53595	1.342526	up
1773483_at	GPI18	YBR004C	Functional ortholog of human PIG-V, which is a mannosyltransferase that transfers the second mannose in glycosylphosphatidylinositol biosynthesis; the authentic, non-tagged protein was localized to mitochondria	2.533659	1.341222	up
1769461_at		YMR317W		2.532955	1.340821	up
1772986_at	MFB1	YDR219C	Mitochondria-associated F-box protein involved in maintenance of normal mitochondrial morphology; interacts with Skp1p through the F-box motif; preferentially localizes to the mother cell during budding	2.532513	1.34057	up
1773352_at	MFM1	YPL060W	Mitochondrial inner membrane magnesium transporter, involved in maintenance of mitochondrial magnesium concentrations and membrane potential; indirectly affects splicing of group II introns; functionally and structurally related to Mrs2p	2.526243	1.336994	up
1772394_at	NGL3	YML118W	3'-5' exonuclease specific for poly-A RNAs; has a domain similar to a magnesium-dependent endonuclease motif in mRNA deadenylase Ccr4p; similar to Ngl1p and Ngl2p	2.523964	1.335692	up
1775175_at	VAM3	YOR106W	Syntaxin-like vacuolar t-SNARE that functions with Vam7p in vacuolar protein trafficking; mediates docking/fusion of late transport intermediates with the vacuole; has an acidic di-leucine sorting signal and C-terminal transmembrane region	2.523512	1.335433	up
1770718_at	SKI7	YOR076C	Coupling protein that mediates interactions between the Ski complex and the cytoplasmic exosome during 3'-5' RNA degradation; eRF3-like domain targets nonstop mRNA for degradation; null mutants have superkiller phenotype	2.523063	1.335177	up
1774234_at	PLP1	YDR183W	Protein that interacts with CCT (chaperonin containing TCP-1) complex and has a role in actin and tubulin folding; has weak similarity to phosducins, which are G-protein regulators	2.522286	1.334732	up
1780083_at	YRA1	YDR381W	RNA binding protein required for export of poly(A)+ mRNA from the nucleus; proposed to couple mRNA export with 3' end processing via its interactions with Mex67p and Pcf11p; functionally redundant with Yra2p, another REF family member	2.52145	1.334254	up
1777472_at		YOL164W-A		2.52041	1.333658	up
1777417_at	RTT103	YDR289C	Protein that interacts with exonuclease Rat1p and Rai1p and plays a role in transcription termination by RNA polymerase II, has an RPR domain	2.517805	1.332167	up

			(carboxy-terminal domain interacting domain); also involved in regulation of Ty1 transposition			
1778008_at		YPR078C		2.51676	1.331568	up
1770539_at	REH1	YLR387C	Cytoplasmic 60S subunit biogenesis factor, associates with pre-60S particles; similar to Rei1p and shares partially redundant function in cytoplasmic 60S subunit maturation; contains dispersed C2H2 zinc finger domains	2.512703	1.32924	up
1771916_at	VBA2	YBR293W	Permease of basic amino acids in the vacuolar membrane	2.512198	1.32895	up
1779703_at	UBP6	YFR010W	Ubiquitin-specific protease situated in the base subcomplex of the 26S proteasome, releases free ubiquitin from branched polyubiquitin chains; works in opposition to Hul5p polyubiquitin elongation activity; mutant has aneuploidy tolerance	2.509693	1.327511	up
1773015_at	FLO8	YER109C	Transcription factor required for flocculation, diploid filamentous growth, and haploid invasive growth; genome reference strain S288C and most laboratory strains have a mutation in this gene	2.508515	1.326834	up
1774081_at	MDH2	YOL126C	Cytoplasmic malate dehydrogenase, one of three isozymes that catalyze interconversion of malate and oxaloacetate; involved in the glyoxylate cycle and gluconeogenesis during growth on two-carbon compounds; interacts with Pck1p and Fbp1	2.506768	1.325829	up
1778034_at	TAP42	YMR028W	Essential protein involved in the TOR signaling pathway; physically associates with the protein phosphatase 2A and the SIT4 protein phosphatase catalytic subunits	2.506368	1.325598	up
1779485_at		YDR249C		2.50629	1.325553	up
1775441_at	HAP4	YKL109W	Subunit of the heme-activated, glucose-repressed Hap2p/3p/4p/5p CCAAT-binding complex, a transcriptional activator and global regulator of respiratory gene expression; provides the principal activation function of the complex	2.505743	1.325238	up
1770166_at		YHR097C		2.503143	1.323741	up
1774891_at	APC9	YLR102C	Subunit of the Anaphase-Promoting Complex/Cyclosome (APC/C), which is a ubiquitin-protein ligase required for degradation of anaphase inhibitors, including mitotic cyclins, during the metaphase/anaphase transition	2.502258	1.32323	up
1776759_at		YKL107W		2.501675	1.322895	up
1773376_at	REC114	YMR133W	Protein involved in early stages of meiotic recombination; possibly involved in the coordination of recombination and meiotic division; mutations lead to premature initiation of the first meiotic division	2.499245	1.321492	up
1775421_at		YHR138C		2.498357	1.32098	up
1776875_at	BDF1	YLR399C	Protein involved in transcription initiation at TATA-containing promoters; associates with the basal transcription factor TFIID; contains two bromodomains; corresponds to the C-terminal region of mammalian TAF1; redundant with Bdf2p	2.497967	1.320755	up
1775386_at	ATR1	YML116W	Multidrug efflux pump of the major facilitator superfamily, required for resistance to aminotriazole and 4-nitroquinoline-N-oxide	2.493773	1.31833	up
1780210_at		YOR052C		2.493012	1.31789	up
1776246_at	MDJ1	YFL016C	Co-chaperone that stimulates the ATPase activity of the HSP70 protein Ssc1p; involved in protein folding/refolding in the mitochodrial matrix; required for proteolysis of misfolded proteins; member of the HSP40 (DnaJ) family of chaperones	2.492027	1.317319	up
1770859_at	IMA1	YGR287C	Major isomaltase (alpha-1,6-glucosidase) required	2.48935	1.315769	up

			for isomaltose utilization; has specificity for isomaltose, palatinose, and methyl-alpha-glucoside; member of the IMA isomaltase family			
1770447_s_at		YEL076C		2.487604	1.314757	up
1779124_at	SPC42	YKL042W	Central plaque component of spindle pole body (SPB); involved in SPB duplication, may facilitate attachment of the SPB to the nuclear membrane	2.484738	1.313094	up
1774158_at	MLF3	YNL074C	Serine-rich protein of unknown function, predicted to be palmitoylated; overproduction suppresses the growth inhibition caused by exposure to the immunosuppressant leflunomide	2.483224	1.312214	up
1779511_at	DIA1	YMR316W	Protein of unknown function, involved in invasive and pseudohyphal growth; green fluorescent protein (GFP)-fusion protein localizes to the cytoplasm in a punctate pattern	2.479802	1.310225	up
1778795_at	MDM34	YGL219C	Mitochondrial component of the ERMES complex that links the ER to mitochondria and may promote inter-organellar calcium and phospholipid exchange as well as coordinating mitochondrial DNA replication and growth	2.478491	1.309462	up
1773494_at		YOR376W-A		2.478222	1.309305	up
1770315_at	ALG12	YNR030W	Alpha-1,6-mannosyltransferase localized to the ER; responsible for the addition of the alpha-1,6 mannose to dolichol-linked Man7GlcNAc2, acts in the dolichol pathway for N-glycosylation	2.47605	1.308041	up
1778053_at	YRO2	YBR054W	Protein of unknown function with similarity to archaeal rhodopsins; the authentic, non-tagged protein is detected in a phosphorylated state in highly purified mitochondria in high-throughput studies; transcriptionally regulated by Haa1p	2.470941	1.30506	up
1777807_at	MAD3	YJL013C	Subunit of the spindle-assembly checkpoint complex, which delays anaphase onset in cells with defects in mitotic spindle assembly; pseudosubstrate inhibitor of APC(Cdc20), the anaphase promoting complex involved in securin (Pds1p) turnover	2.469083	1.303975	up
1773195_at	YEL1	YBL060W	Guanine nucleotide exchange factor specific for Arf3p; localized to the bud neck and tip; required for localization of Arf3p to the bud neck and tip	2.467372	1.302975	up
1772131_s_at		YNL033W		2.466169	1.302272	up
1771289_at	CAB1	YDR531W	Pantothenate kinase (ATP:D-pantothenate 4'-phosphotransferase, EC 2.7.1.33); catalyzes the first committed step in the universal biosynthetic pathway for synthesis of coenzyme A (CoA); transcriptionally regulated by Upc2p via a sterol response element	2.466118	1.302242	up
1770695_at	TRX3	YCR083W	Mitochondrial thioredoxin, highly conserved oxidoreductase required to maintain the redox homeostasis of the cell, forms the mitochondrial thioredoxin system with Trr2p, redox state is maintained by both Trr2p and Glr1p	2.461514	1.299546	up
1774926_at	SDS24	YBR214W	One of two S. cerevisiae homologs (Sds23p and Sds24p) of the S. pombe Sds23 protein, which is implicated in APC/cyclosome regulation; involved in cell separation during budding; may play an indirect role in fluid-phase endocytosis	2.457856	1.297401	up
1775399_at	BAP2	YBR068C	High-affinity leucine permease, functions as a branched-chain amino acid permease involved in the uptake of leucine, isoleucine and valine; contains 12 predicted transmembrane domains	2.457124	1.296971	up
1774561_at	RMP1	YLR145W	Subunit of RNase MRP, which processes pre-rRNA and has a role in cell cycle-regulated degradation of daughter cell-specific mRNAs; unlike most subunits, not shared between RNase MRP and nuclear RNase P	2.454508	1.295434	up

1777119_at	MUS81	YDR386W	Subunit of the structure-specific Mms4p-Mus81p endonuclease that cleaves branched DNA; involved in DNA repair, replication fork stability, and joint molecule formation/resolution during meiotic recombination; helix-hairpin-helix protein	2.453928	1.295093	up
1780192_at	RNA14	YMR061W	Component of the cleavage and polyadenylation factor I (CF I); CF 1, composed of the CF 1A complex (Rna14p, Rna15p, Clp1p, Pcf11p) and Hrp1, is involved in cleavage and polyadenylation of mRNA 3' ends; bridges interaction between Rna15p and Hrp1p in the CF I complex; mutant displays reduced transcription elongation in the G-less-based run-on (GLRO) assay; required for gene looping	2.453684	1.29495	up
1777449_at		YIR014W		2.452423	1.294208	up
1774838_at	MLH3	YPL164C	Protein involved in DNA mismatch repair and crossing-over during meiotic recombination; forms a complex with Mlh1p; mammalian homolog is implicated mammalian microsatellite instability	2.450995	1.293368	up
1774353_at	UPS3	YDR185C	Mitochondrial protein of unknown function; similar to Ups1p and Ups2p which are involved in regulation of mitochondrial cardiolipin and phosphatidylethanolamine levels; null is viable but interacts synthetically with ups1 and ups2 mutations	2.449037	1.292214	up
1780013_at	ECI1	YLR284C	Peroxisomal delta3,delta2-enoyl-CoA isomerase, hexameric protein that converts 3-hexenoyl-CoA to trans-2-hexenoyl-CoA, essential for the beta-oxidation of unsaturated fatty acids, oleate-induced	2.448691	1.292011	up
1772821_at	STU1	YBL034C	Component of the mitotic spindle that binds to interpolar microtubules via its association with beta-tubulin (Tub2p); required for interpolar microtubules to provide an outward force on the spindle poles	2.446651	1.290808	up
1773127_at	RRD2	YPL152W	Activator of the phosphotyrosyl phosphatase activity of PP2A,peptidyl-prolyl cis/trans-isomerase; regulates G1 phase progression, the osmoresponse, microtubule dynamics; subunit of the Tap42p-Pph21p-Rrd2p complex	2.446365	1.290639	up
1776670_at		YFL042C		2.445355	1.290044	up
1774022_at	ENB1	YOL158C	Endosomal ferric enterobactin transporter, expressed under conditions of iron deprivation; member of the major facilitator superfamily; expression is regulated by Rcs1p and affected by chloroquine treatment	2.442119	1.288133	up
1769876_at		YOR338W		2.436543	1.284835	up
1770442_at	BNR1	YIL159W	Formin, nucleates the formation of linear actin filaments, involved in cell processes such as budding and mitotic spindle orientation which require the formation of polarized actin cables, functionally redundant with BNI1	2.435338	1.284122	up
1775384_at	BNA3	YJL060W	Kynurenine aminotransferase, catalyzes formation of kynurenic acid from kynurenine; potential Cdc28p substrate	2.435066	1.283961	up
1778556_at		YML003W		2.431242	1.281693	up
1771649_at		YGL242C		2.429872	1.280881	up
1775927_at	MSS2	YDL107W	Peripherally bound inner membrane protein of the mitochondrial matrix involved in membrane insertion of C-terminus of Cox2p, interacts genetically and physically with Cox18p	2.423757	1.277245	up
1775729_at	MLS1	YNL117W	Malate synthase, enzyme of the glyoxylate cycle, involved in utilization of non-fermentable carbon sources; expression is subject to carbon catabolite repression; localizes in peroxisomes during growth	2.421995	1.276196	up

			in oleic acid medium			
1780166_at	AAD14	YNL331C	Putative aryl-alcohol dehydrogenase; similar to P. chrysosporium aryl-alcohol dehydrogenase; mutational analysis has not yet revealed a physiological role	2.421489	1.275894	up
1774033_at	ATG13	YPR185W	Regulatory subunit of the Atg1p signaling complex; stimulates Atg1p kinase activity; required for vesicle formation during autophagy and the cytoplasm-to-vacuole targeting (Cvt) pathway; involved in Atg9p, Atg23p, and Atg27p cycling	2.421268	1.275763	up
1778510_at	CUP2	YGL166W	Copper-binding transcription factor; activates transcription of the metallothionein genes CUP1-1 and CUP1-2 in response to elevated copper concentrations	2.421111	1.275669	up
1772838_at	GCY1	YOR120W	Putative NADP(+) coupled glycerol dehydrogenase, proposed to be involved in an alternative pathway for glycerol catabolism; also has mRNA binding activity; member of the aldo-keto reductase (AKR) family	2.417238	1.273359	up
1774653_at	AIM37	YNL100W	Mitochondrial inner membrane protein; component of the mitochondrial inner membrane organizing system (MitOS, MICOS, or MINOS), a scaffold-like structure on the intermembrane space side of the inner membrane which has a role in the maintenance of crista junctions and inner membrane architecture	2.41661	1.272985	up
1777402_at		YOR186W		2.414738	1.271867	up
1770032_at	EEB1	YPL095C	Acyl-coenzymeA:ethanol O-acyltransferase responsible for the major part of medium-chain fatty acid ethyl ester biosynthesis during fermentation; possesses short-chain esterase activity; may be involved in lipid metabolism and detoxification	2.411256	1.269785	up
1776589_at	MID2	YLR332W	O-glycosylated plasma membrane protein that acts as a sensor for cell wall integrity signaling and activates the pathway; interacts with Rom2p, a guanine nucleotide exchange factor for Rho1p, and with cell integrity pathway protein Zeo1p	2.410538	1.269355	up
1773587_at	AGC1	YPR021C	Mitochondrial amino acid transporter, acts both as a glutamate uniporter and as an aspartate-glutamate exchanger; involved in nitrogen metabolism and nitrogen compound biosynthesis	2.410134	1.269113	up
1774060_at	SCS22	YBL091C-A	Protein involved in regulation of phospholipid metabolism; homolog of Scs2p; similar to D. melanogaster inturned protein	2.409053	1.268466	up
1779095_at	DAL4	YIR028W	Allantoin permease; expression sensitive to nitrogen catabolite repression and induced by allophanate, an intermediate in allantoin degradation	2.408981	1.268423	up
1770489_at	GLN3	YER040W	Transcriptional activator of genes regulated by nitrogen catabolite repression (NCR), localization and activity regulated by quality of nitrogen source	2.408013	1.267843	up
1778380_at	MGA1	YGR249W	Protein similar to heat shock transcription factor; multicopy suppressor of pseudohyphal growth defects of ammonium permease mutants	2.406877	1.267162	up
1779609_at	DDI1	YER143W	DNA damage-inducible v-SNARE binding protein with a role in suppression of protein secretion; may play a role in S-phase checkpoint control; has ubiquitin-associated (UBA), ubiquitin-like (UBL), and retroviral-like proteinase (RVP) domains	2.4023	1.264416	up
1779677_at	PEX7	YDR142C	Peroxisomal signal receptor for the N-terminal nonapeptide signal (PTS2) of peroxisomal matrix proteins; WD repeat protein; defects in human homolog cause lethal rhizomelic chondrodysplasia punctata (RCDP)	2.400046	1.263062	up

1770538_at	ARR2	YPR200C	Arsenate reductase required for arsenate resistance; converts arsenate to arsenite which can then be exported from cells by Arr3p	2.399411	1.262681	up
1776324_at	MEI4	YER044C-A	Meiosis-specific protein involved in double-strand break formation during meiotic recombination; required for chromosome synapsis and production of viable spores	2.398598	1.262191	up
1775493_at	KAR5	YMR065W	Protein required for nuclear membrane fusion during karyogamy, localizes to the membrane with a soluble portion in the endoplasmic reticulum lumen, may form a complex with Jem1p and Kar2p; expression of the gene is regulated by pheromone	2.398441	1.262097	up
1776251_at		YFR032C-B		2.39624	1.260772	up
1773936_at		YIL055C		2.393249	1.258971	up
1769958_at	GID8	YMR135C	Protein of unknown function, involved in proteasome-dependent catabolite inactivation of fructose-1,6-bisphosphatase; contains LisH and CTLH domains, like Vid30p; dosage-dependent regulator of START	2.392753	1.258672	up
1769482_at		YLR125W		2.391684	1.258027	up
1773737_at	AGP3	YFL055W	Low-affinity amino acid permease, may act to supply the cell with amino acids as nitrogen source in nitrogen-poor conditions; transcription is induced under conditions of sulfur limitation; plays a role in regulating Ty1 transposition	2.391286	1.257787	up
1771599_at	HOS4	YIL112W	Subunit of the Set3 complex, which is a meiotic-specific repressor of sporulation specific genes that contains deacetylase activity; potential Cdc28p substrate	2.390169	1.257113	up
1774707_at	RPN8	YOR261C	Essential, non-ATPase regulatory subunit of the 26S proteasome; has similarity to the human p40 proteasomal subunit and to another S. cerevisiae regulatory subunit, Rpn11p	2.386837	1.2551	up
1770261_at	SET6	YPL165C	SET domain protein of unknown function; deletion heterozygote is sensitive to compounds that target ergosterol biosynthesis, may be involved in compound availability	2.385827	1.25449	up
1774087_at	ADY3	YDL239C	Protein required for spore wall formation, thought to mediate assembly of a Don1p-containing structure at the leading edge of the prospore membrane via interaction with spindle pole body components; potentially phosphorylated by Cdc28p	2.383215	1.252909	up
1771802_at	GIP2	YER054C	Putative regulatory subunit of the protein phosphatase Glc7p, involved in glycogen metabolism; contains a conserved motif (GVNK motif) that is also found in Gac1p, Pig1p, and Pig2p	2.377975	1.249733	up
1779016_at	MMS4	YBR098W	Subunit of the structure-specific Mms4p-Mus81p endonuclease that cleaves branched DNA; involved in recombination, DNA repair, and joint molecule formation/resolution during meiotic recombination	2.376959	1.249117	up
1773386_at	AIM33	YML087C	Putative protein of unknown function, highly conserved across species and orthologous to human CYB5R4; null mutant displays reduced frequency of mitochondrial genome loss	2.373694	1.247134	up
1776860_at	GRX6	YDL010W	Cis-golgi localized monothiol glutaredoxin that binds an iron-sulfur cluster; more similar in activity to dithiol than other monothiol glutaredoxins; involved in the oxidative stress response; functional overlap with GRX7	2.373333	1.246914	up
1774315_at	PPZ2	YDR436W	Serine/threonine protein phosphatase Z, isoform of Ppz1p; involved in regulation of potassium transport, which affects osmotic stability, cell cycle progression, and halotolerance	2.372886	1.246643	up

1771574_at	UPC2	YDR213W	Sterol regulatory element binding protein, induces transcription of sterol biosynthetic genes and of DAN/TIR gene products; Ecm22p homolog; relocates from intracellular membranes to perinuclear foci on sterol depletion	2.371706	1.245925	up
1777633_at	UFD1	YGR048W	Substrate-recruiting cofactor of the Cdc48p-Npl4p-Ufd1p segregase; polyubiquitin binding protein that assists in the dislocation of misfolded, ERAD substrates that are subsequently delivered to the proteasome for degradation; involved in the regulated destruction of resident ER membrane proteins, such as HMG-CoA reductase (Hmg1/2p) and cytoplasmic proteins (Fbp1p); involved in mobilizing membrane bound transaction factors by regulated Ub/proteasome-dependent processing (RUP)	2.369788	1.244758	up
1771117_at	SNG1	YGR197C	Protein involved in resistance to nitrosoguanidine (MNNG) and 6-azauracil (6-AU); expression is regulated by transcription factors involved in multidrug resistance	2.369483	1.244572	up
1769621_at	DID2	YKR035W-A	Class E protein of the vacuolar protein-sorting (Vps) pathway; binds Vps4p and directs it to dissociate ESCRT-III complexes; forms a functional and physical complex with Ist1p; human ortholog may be altered in breast tumors	2.369285	1.244452	up
1777646_at	SPO75	YLL005C	Meiosis-specific protein of unknown function, required for spore wall formation during sporulation; dispensable for both nuclear divisions during meiosis	2.368119	1.243742	up
1778728_at	RCR2	YDR003W	Vacuolar protein that presumably functions within the endosomal-vacuolar trafficking pathway, affecting events that determine whether plasma membrane proteins are degraded or routed to the plasma membrane; similar to Rcr1p	2.366897	1.242997	up
1773771_at	OCA5	YHL029C	Cytoplasmic protein required for replication of Brome mosaic virus in S. cerevisiae, which is a model system for studying replication of positive-strand RNA viruses in their natural hosts	2.364353	1.241445	up
1778758_at	RMD5	YDR255C	Conserved protein that has an E3-like ubiquitin ligase activity necessary for polyubiquitination and degradation of the gluconeogenic enzyme fructose-1,6-bisphosphatase; also required for sporulation; has a degenerate RING finger domain	2.363627	1.241003	up
1779277_at	SMP1	YBR182C	Putative transcription factor involved in regulating the response to osmotic stress; member of the MADS-box family of transcription factors	2.360409	1.239037	up
1770850_at		YHR214C-D		2.360203	1.238911	up
1773356_at	MEP2	YNL142W	Ammonium permease involved in regulation of pseudohyphal growth; belongs to a ubiquitous family of cytoplasmic membrane proteins that transport only ammonium (NH4+); expression is under the nitrogen catabolite repression regulation	2.359432	1.23844	up
1775620_at	IES2	YNL215W	Protein that associates with the INO80 chromatin remodeling complex under low-salt conditions; essential for growth under anaerobic conditions	2.358042	1.237589	up
1772981_at	RTT107	YHR154W	Protein implicated in Mms22-dependent DNA repair during S phase; involved in recruiting the SMC5/6 complex to double-strand breaks; DNA damage induces phosphorylation by Mec1p at one or more SQ/TQ motifs; interacts with Mms22p and Slx4p; has four BRCT domains; has a role in regulation of Ty1 transposition	2.353435	1.234768	up
1769601_at	ISR1	YPR106W	Predicted protein kinase, overexpression causes sensitivity to staurosporine, which is a potent inhibitor of protein kinase C	2.352195	1.234008	up

1773552_at	PRE7	YBL041W	Beta 6 subunit of the 20S proteasome	2.352159	1.233985	up
1774330_at	JSN1	YJR091C	Member of the Puf family of RNA-binding proteins, interacts with mRNAs encoding membrane-associated proteins; involved in localizing the Arp2/3 complex to mitochondria; overexpression causes increased sensitivity to benomyl	2.351143	1.233362	up
1769487_at		YKL063C		2.351025	1.23329	up
1771643_at	HSV2	YGR223C	Phosphatidylinositol 3,5-bisphosphate-binding protein, plays a role in micronucleophagy; belongs to the PROPPIN family of proteins; predicted to fold as a seven-bladed beta-propeller; displays punctate cytoplasmic localization	2.348497	1.231738	up
1773639_at	BUG1	YDL099W	Cis-golgi localized protein involved in ER to Golgi transport; forms a complex with the mammalian GRASP65 homolog, Grh1p; mutants are compromised for the fusion of ER-derived vesicles with Golgi membranes	2.347563	1.231164	up
1775873_at	IDP3	YNL009W	Peroxisomal NADP-dependent isocitrate dehydrogenase, catalyzes oxidation of isocitrate to alpha-ketoglutarate with the formation of NADP(H+), required for growth on unsaturated fatty acids	2.347538	1.231148	up
1771977_at		YBR085C-A		2.342126	1.227819	up
1777044_at	TFB6	YOR352W	Subunit of TFIIH complex; facilities dissociation of the Ssl2p helices from TFIIH; expression levels regulated by Arg5,6p; green fluorescent protein (GFP)-fusion protein localizes to the cytoplasm and nucleus	2.339962	1.226485	up
1778344_at		YGL010W		2.338959	1.225866	up
1770013_at		YFL052W		2.337469	1.224948	up
1771902_at	SGF73	YGL066W	SAGA complex subunit; has a role in anchoring the deubiquitination module into SAGA and SLIK complexes; involved in preinitiation complex assembly at promoters; human ortholog ataxin-7 is associated with spinocerebellar ataxia diseases; mutant displays reduced transcription elongation in the G-less-based run-on (GLRO) assay	2.332331	1.221772	up
1779635_at	MCX1	YBR227C	Mitochondrial matrix protein; putative ATP-binding chaperone with non-proteolytic function; similar to bacterial ClpX proteins	2.331981	1.221556	up
1772188_at		YOR059C		2.331678	1.221368	up
1772873_at	MAL13	YGR288W	MAL-activator protein, part of complex locus MAL1; nonfunctional in genomic reference strain S288C	2.331329	1.221153	up
1770178_at		YDL119C		2.328625	1.219479	up
1775412_at	SHU2	YDR078C	Component of the Shu complex, which promotes error-free DNA repair; Shu complex mediates inhibition of Srs2p function	2.32483	1.217125	up
1773424_at	FMP10	YER182W	Putative protein of unknown function; the authentic, non-tagged protein is detected in highly purified mitochondria in high-throughput studies	2.322488	1.215671	up
1780029_at	CDC15	YAR019C	Protein kinase of the Mitotic Exit Network that is localized to the spindle pole bodies at late anaphase; promotes mitotic exit by directly switching on the kinase activity of Dbf2p; required for spindle disassembly after meiosis II	2.322311	1.215561	up
1777614_at	PIN3	YPR154W	Protein that induces appearance of [PIN+] prion when overproduced	2.31977	1.213982	up
1776270_at		YLR211C		2.31669	1.212065	up
1776449_at	PTP2	YOR208W	Phosphotyrosine-specific protein phosphatase involved in the inactivation of mitogen-activated	2.314915	1.210959	up

			protein kinase (MAPK) during osmolarity sensing; dephosporylates Hog1p MAPK and regulates its localization; localized to the nucleus			
1774545_at	SDP1	YIL113W	Stress-inducible dual-specificity MAP kinase phosphatase, negatively regulates Slt2p MAP kinase by direct dephosphorylation, diffuse localization under normal conditions shifts to punctate localization after heat shock	2.31369	1.210196	up
1772284_at		YLR225C		2.313304	1.209955	up
1769638_at		YNL095C		2.313	1.209765	up
1773050_at	CLG1	YGL215W	Cyclin-like protein that interacts with Pho85p; has sequence similarity to G1 cyclins PCL1 and PCL2	2.309044	1.207296	up
1778783_at	MET31	YPL038W	Zinc-finger DNA-binding protein, involved in transcriptional regulation of the methionine biosynthetic genes, similar to Met32p	2.308713	1.207089	up
1770559_at	ATG12	YBR217W	Conserved ubiquitin-like modifier involved in autophagy and the Cvt pathway; conjugated to Atg5p to form a complex involved in Atg8p lipidation; Atg12p-Atg5p also forms a complex with Atg16p that is required for autophagosome formation	2.306077	1.205441	up
1776355_at	VPS21	YOR089C	Rab family GTPase required for endocytic transport and for sorting of vacuolar hydrolases; localized in endocytic intermediates; detected in mitochondria; geranylgeranylation required for membrane association; mammalian Rab5 homolog	2.305548	1.20511	up
1777268_at	TES1	YJR019C	Peroxisomal acyl-CoA thioesterase likely to be involved in fatty acid oxidation rather than fatty acid synthesis; conserved protein also found in human peroxisomes; TES1 mRNA levels increase during growth on fatty acids	2.305255	1.204927	up
1776273_x_at	IMD2	YHR216W	Inosine monophosphate dehydrogenase, catalyzes the rate-limiting step in GTP biosynthesis, expression is induced by mycophenolic acid resulting in resistance to the drug, expression is repressed by nutrient limitatio	2.304225	1.204282	up
1774678_at	HST2	YPL015C	Cytoplasmic member of the silencing information regulator 2 (Sir2) family of NAD(+)-dependent protein deacetylases; modulates nucleolar (rDNA) and telomeric silencing; possesses NAD(+)-dependent histone deacetylase activity in vitro	2.303542	1.203854	up
1773805_at		YJR098C		2.287797	1.193959	up
1777467_at	IGO1	YNL157W	Protein required for initiation of G0 program; prevents degradation of nutrient-regulated mRNAs via the 5'-3' mRNA decay pathway; phosphorylated by Rim15p; GFP protein localizes to the cytoplasm and nucleus; similar to Igo2p	2.286552	1.193173	up
1771961_at	DBF20	YPR111W	Ser/Thr kinase involved in late nuclear division, one of the mitotic exit network (MEN) proteins; necessary for the execution of cytokinesis; also plays a role in regulating the stability of SWI5 and CLB2 mRNAs	2.286508	1.193146	up
1773972_at	MRC1	YCL061C	S-phase checkpoint protein required for DNA replication; interacts with and stabilizes Pol2p at stalled replication forks during stress, where it forms a pausing complex with Tof1p and is phosphorylated by Mec1p; protects uncapped telomeres	2.286201	1.192952	up
1769384_at	SPO13	YHR014W	Meiosis-specific protein, involved in maintaining sister chromatid cohesion during meiosis I as well as promoting proper attachment of kinetochores to the spindle during meiosis I and meiosis II	2.285883	1.192752	up
1773875_at	DIA3	YDL024C	Protein of unknown function, involved in invasive and pseudohyphal growth	2.284349	1.191783	up

1770558_at	NSE4	YDL105W	Component of the SMC5-SMC6 complex; this complex plays a key role in the removal of X-shaped DNA structures that arise between sister chromatids during DNA replication and repair	2.284168	1.191669	up
1776502_at	RTT105	YER104W	Protein with a role in regulation of Ty1 transposition	2.2818	1.190172	up
1771013_at		YLR271W		2.279936	1.188994	up
1773460_at	RAD4	YER162C	Protein that recognizes and binds damaged DNA (with Rad23p) during nucleotide excision repair; subunit of Nuclear Excision Repair Factor 2 (NEF2); also involved, with Rad23p, in turnover of ubiquitylated proteins	2.278589	1.18814	up
1769325_at	RRI1	YDL216C	Catalytic subunit of the COP9 signalosome (CSN) complex that acts as an isopeptidase in cleaving the ubiquitin-like protein Nedd8 from SCF ubiquitin ligases; metalloendopeptidase involved in the adaptation to pheromone signaling	2.275522	1.186197	up
1770409_at		YER137C		2.271358	1.183555	up
1776157_at	BAS1	YKR099W	Myb-related transcription factor involved in regulating basal and induced expression of genes of the purine and histidine biosynthesis pathways; also involved in regulation of meiotic recombination at specific genes	2.270773	1.183184	up
1771167_at	NBP35	YGL091C	Essential iron-sulfur cluster binding protein localized in the cytoplasm; forms a complex with Cfd1p that is involved in iron-sulfur protein assembly in the cytosol; similar to P-loop NTPases	2.269941	1.182655	up
1777394_at	ATG10	YLL042C	Conserved E2-like conjugating enzyme that mediates formation of the Atg12p-Atg5p conjugate, which is a critical step in autophagy	2.268191	1.181542	up
1772915_at	ACS1	YAL054C	Acetyl-coA synthetase isoform which, along with Acs2p, is the nuclear source of acetyl-coA for histone acetylation; expressed during growth on nonfermentable carbon sources and under aerobic conditions	2.264052	1.178907	up
1769627_at	PCD1	YLR151C	Peroxisomal nudix pyrophosphatase with specificity for coenzyme A and CoA derivatives, may function to remove potentially toxic oxidized CoA disulfide from peroxisomes to maintain the capacity for beta-oxidation of fatty acids	2.263369	1.178472	up
1774893_at		YJR151W-A		2.262881	1.178161	up
1778226_at	VPS51	YKR020W	Component of the GARP (Golgi-associated retrograde protein) complex, Vps51p-Vps52p-Vps53p-Vps54p, which is required for the recycling of proteins from endosomes to the late Golgi; links the (VFT/GARP) complex to the SNARE Tlg1p	2.261365	1.177194	up
1769654_at	SWP82	YFL049W	Member of the SWI/SNF chromatin remodeling complex in which it plays an as yet unidentified role; has identifiable counterparts in closely related yeast species; abundantly expressed in many growth conditions; paralog of Npl6p	2.252973	1.17183	up
1778434_at	SRN2	YLR119W	Component of the ESCRT-I complex, which is involved in ubiquitin-dependent sorting of proteins into the endosome; suppressor of rna1-1 mutation; may be involved in RNA export from nucleus	2.252148	1.171302	up
1774804_at	ATG3	YNR007C	E2-like enzyme involved in autophagy and the cytoplasm-to-vacuole targeting (Cvt) pathway; plays a role in formation of Atg8p-phosphatidylethanolamine conjugates, which are involved in membrane dynamics during autophagy and Cvt	2.251902	1.171144	up
1772401_at	VPS72	YDR485C	Htz1p-binding component of the SWR1 complex, which exchanges histone variant H2AZ (Htz1p) for chromatin-bound histone H2A; required for	2.251455	1.170858	up

			vacuolar protein sorting			
1777910_at	ZDS2	YML109W	Protein with a role in regulating Swe1p-dependent polarized growth; involved in maintenance of Cdc55p in the cytoplasm where it promotes mitotic entry; interacts with silencing proteins at the telomere; implicated in the mitotic exit network through regulation of Cdc14p localization; paralog of Zds1p	2.249066	1.169326	up
1779001_at	RAD7	YJR052W	Protein that recognizes and binds damaged DNA in an ATP-dependent manner (with Rad16p) during nucleotide excision repair; subunit of Nucleotide Excision Repair Factor 4 (NEF4) and the Elongin-Cullin-Socs (ECS) ligase complex	2.248899	1.169219	up
1772554_at	GAL10	YBR019C	UDP-glucose-4-epimerase, catalyzes the interconversion of UDP-galactose and UDP-D-glucose in galactose metabolism; also catalyzes the conversion of alpha-D-glucose or alpha-D-galactose to their beta-anomers	2.246459	1.167652	up
1775724_at	MUK1	YPL070W	Cytoplasmic protein of unknown function containing a Vps9 domain; computational analysis of large-scale protein-protein interaction data suggests a possible role in transcriptional regulation	2.245652	1.167134	up
1776350_at	ATG23	YLR431C	Peripheral membrane protein required for the cytoplasm-to-vacuole targeting (Cvt) pathway and efficient macroautophagy; cycles between the phagophore assembly site (PAS) and non-PAS locations; forms a complex with Atg9p and Atg27p	2.245431	1.166993	up
1775211_at	CUS1	YMR240C	Protein required for assembly of U2 snRNP into the spliceosome, forms a complex with Hsh49p and Hsh155p	2.244816	1.166597	up
1779601_at	AIM46	YHR199C	Putative protein of unknown function; the authentic, non-tagged protein is detected in highly purified mitochondria in high-throughput studies; null mutant displays elevated frequency of mitochondrial genome loss	2.244385	1.16632	up
1777245_at	SNF12	YNR023W	73 kDa subunit of the SWI/SNF chromatin remodeling complex involved in transcriptional regulation; homolog of Rsc6p subunit of the RSC chromatin remodeling complex; deletion mutants are temperature-sensitive	2.24389	1.166002	up
1779163_at	CTK3	YML112W	Gamma subunit of C-terminal domain kinase I (CTDK-I), which phosphorylates both RNA pol II subunit Rpo21p to affect transcription and pre-mRNA 3' end processing, and ribosomal protein Rps2p to increase translational fidelity	2.236864	1.161478	up
1771758_at	OCA4	YCR095C	Cytoplasmic protein required for replication of Brome mosaic virus in S. cerevisiae, which is a model system for studying replication of positive-strand RNA viruses in their natural hosts	2.235716	1.160737	up
1769541_at	PUF2	YPR042C	Member of the PUF protein family, which is defined by the presence of Pumilio homology domains that confer RNA binding activity; preferentially binds mRNAs encoding membrane-associated proteins	2.23468	1.160068	up
1777053_at	ATH1	YPR026W	Acid trehalase required for utilization of extracellular trehalose	2.234177	1.159744	up
1773769_at		YHR022C-A		2.233397	1.15924	up
1771150_at		YPL107W		2.233358	1.159215	up
1774909_at	ECM18	YDR125C	Protein of unknown function, similar to Rlp24p	2.229256	1.156562	up
1777294_at		YML054C-A		2.229241	1.156553	up
1770865_at	PEA2	YER149C	Coiled-coil polarisome protein required for polarized morphogenesis, cell fusion, and low affinity Ca2+ influx; forms polarisome complex with Bni1p, Bud6p, and Spa2p; localizes to sites of	2.227031	1.155121	up

			polarized growth			
1771931_at	KAR3	YPR141C	Minus-end-directed microtubule motor that functions in mitosis and meiosis, localizes to the spindle pole body and localization is dependent on functional Cik1p, required for nuclear fusion during mating; potential Cdc28p substrate	2.225479	1.154116	up
1775087_at	LRE1	YCL051W	Protein involved in control of cell wall structure and stress response; overproduction confers resistance to cell-wall degrading enzymes; exhibits genetic interactions with genes involved in the cell wall integrity pathway	2.225194	1.153931	up
1777551_at	MPP6	YNR024W	Nuclear exosome-associated RNA binding protein; involved in surveillance of pre-rRNAs and pre-mRNAs, and the degradation of cryptic non-coding RNAs (ncRNA); copurifies with ribosomes	2.224463	1.153457	up
1773887_at	PEX19	YDL065C	Chaperone and import receptor for newly-synthesized class I PMPs; binds peroxisomal membrane proteins (PMPs) in the cytoplasm and delivers them to the peroxisome for subsequent insertion into the peroxisomal membrane; interacts with Myo2p and contributes to peroxisome partitioning	2.223891	1.153086	up
1769665_at	SLD2	YKL108W	Single-stranded DNA origin-binding and annealing protein; required for the initiation of DNA replication; phosphorylated in S phase by cyclin-dependent kinases (Cdks), promoting origin binding, DNA replication and Dpb11p complex formation; component of the preloading complex; unphosphorylated or CDK-phosphorylated Sld2p binds to the MCM2-7 complex;required for the S phase checkpoint	2.22379	1.15302	up
1771853_at		YGL041C-B		2.221189	1.151332	up
1773145_s_at		YGR109W-A		2.221026	1.151226	up
1773974_at	PIG1	YLR273C	Putative targeting subunit for the type-1 protein phosphatase Glc7p that tethers it to the Gsy2p glycogen synthase	2.219079	1.149961	up
1777587_at		YPR159C-A		2.21897	1.14989	up
1780070_at	ZTA1	YBR046C	NADPH-dependent quinone reductase, GFP-tagged protein localizes to the cytoplasm and nucleus; has similarity to E. coli quinone oxidoreductase and to human zeta-crystallin	2.217787	1.149121	up
1769868_at	RPM2	YML091C	Protein subunit of mitochondrial RNase P, has roles in nuclear transcription, cytoplasmic and mitochondrial RNA processing, and mitochondrial translation; distributed to mitochondria, cytoplasmic processing bodies, and the nucleus	2.215379	1.147553	up
1779236_at	HAP2	YGL237C	Subunit of the heme-activated, glucose-repressed Hap2p/3p/4p/5p CCAAT-binding complex, a transcriptional activator and global regulator of respiratory gene expression; contains sequences sufficient for both complex assembly and DNA binding	2.214506	1.146985	up
1772586_at	RAD28	YDR030C	Protein involved in DNA repair, related to the human CSA protein that is involved in transcription-coupled repair nucleotide excision repair	2.21366	1.146433	up
1769478_at		YPL199C		2.213321	1.146213	up
1779578_at	UBC5	YDR059C	Ubiquitin-conjugating enzyme that mediates selective degradation of short-lived, abnormal, or excess proteins, including histone H3; central component of the cellular stress response; expression is heat inducible	2.210083	1.144101	up
1779688_at	SHH4	YLR164W	Mitochondrial inner membrane protein of unknown function; similar to Tim18p and Sdh4p; a fraction	2.208556	1.143104	up

			copurifies with Sdh3p, but Shh4p is neither a stoichiometric subunit of succinate dehydrogenase nor of the TIM22 translocase; expression induced by nitrogen limitation in a GLN3, GAT1-dependent manner			
1773262_at	SIP5	YMR140W	Protein of unknown function; interacts with both the Reg1p/Glc7p phosphatase and the Snf1p kinase	2.208491	1.143061	up
1779234_at	PEX3	YDR329C	Peroxisomal membrane protein (PMP) required for proper localization and stability of PMPs; anchors peroxisome retention factor Inp1p at the peroxisomal membrane; interacts with Pex19p	2.205757	1.141274	up
1771215_at	MET22	YOL064C	Bisphosphate-3'-nucleotidase, involved in salt tolerance and methionine biogenesis; dephosphorylates 3'-phosphoadenosine-5'-phosphate and 3'-phosphoadenosine-5'-phosphosulfate, intermediates of the sulfate assimilation pathway	2.205402	1.141042	up
1774287_at	CRD1	YDL142C	Cardiolipin synthase; produces cardiolipin, which is a phospholipid of the mitochondrial inner membrane that is required for normal mitochondrial membrane potential and function; also required for normal vacuolar ion homeostasis	2.204829	1.140667	up
1774515_at	TFC7	YOR110W	One of six subunits of the RNA polymerase III transcription initiation factor complex (TFIIIC); part of the TauA globular domain of TFIIIC that binds DNA at the BoxA promoter sites of tRNA and similar genes	2.202162	1.13892	up
1775540_at		YKL050C		2.201258	1.138328	up
1774095_at	LAG2	YOL025W	Protein that negatively regulates the SCF E3-ubiquitin ligase by interacting with and preventing neddyation of the cullin subunit, Cdc53p; longevity determinant that is preferentially expressed in young cells; similar to mammalian Cand1	2.201233	1.138312	up
1774184_at		YCR061W		2.199256	1.137015	up
1771803_at	MSS1	YMR023C	Mitochondrial protein, forms a heterodimer complex with Mto1p that performs the 5-carboxymethylaminomethyl modification of the wobble uridine base in mitochondrial tRNAs; similar to human GTPBP3	2.198163	1.136298	up
1775858_at	MBP1	YDL056W	Transcription factor involved in regulation of cell cycle progression from G1 to S phase, forms a complex with Swi6p that binds to MluI cell cycle box regulatory element in promoters of DNA synthesis genes	2.19725	1.135699	up
1772181_at		YKL133C		2.196684	1.135327	up
1779809_at	DMC1	YER179W	Meiosis-specific protein required for repair of double-strand breaks and pairing between homologous chromosomes; homolog of Rad51p and the bacterial RecA protein	2.196286	1.135066	up
1778184_at		YOL114C		2.194266	1.133739	up
1770039_at	FRE6	YLL051C	Putative ferric reductase with similarity to Fre2p; expression induced by low iron levels	2.194085	1.133619	up
1778183_at	PDR10	YOR328W	ATP-binding cassette (ABC) transporter, multidrug transporter involved in the pleiotropic drug resistance network; regulated by Pdr1p and Pdr3p	2.194019	1.133576	up
1777842_at	NAS2	YIL007C	Proteasome-interacting protein involved in the assembly of the base subcomplex of the 19S proteasomal regulatory particle (RP); similar to mammalian proteasomal modulator subunit; non-essential gene; interacts with Rpn4p	2.193049	1.132938	up
1776954_at	USE1	YGL098W	Essential SNARE protein localized to the ER, involved in retrograde traffic from the Golgi to the ER; forms a complex with the SNAREs Sec22p, Sec20p and Ufe1p	2.191651	1.132018	up

1778752_at	ARG80	YMR042W	Transcription factor involved in regulation of arginine-responsive genes; acts with Arg81p and Arg82p	2.189059	1.130311	up
1778177_at	MIG1	YGL035C	Transcription factor involved in glucose repression; sequence specific DNA binding protein containing two Cys2His2 zinc finger motifs; regulated by the SNF1 kinase and the GLC7 phosphatase	2.188601	1.130009	up
1773168_at	SUT1	YGL162W	Transcription factor of the Zn[II]2Cys6 family involved in sterol uptake; involved in induction of hypoxic gene expression	2.188558	1.129981	up
1778597_at	PCL8	YPL219W	Cyclin, interacts with Pho85p cyclin-dependent kinase (Cdk) to phosphorylate and regulate glycogen synthase, also activates Pho85p for Glc8p phosphorylation	2.186515	1.128634	up
1774154_at	YPT11	YNL304W	Rab family GTPase that interacts with the C-terminal tail domain of Myo2p; mediates distribution of mitochondria and endoplasmic reticuli to daughter cells	2.183303	1.126512	up
1776385_at	BDP1	YNL039W	Essential subunit of RNA polymerase III transcription factor (TFIIIB), which is involved in transcription of genes encoding tRNAs, 5S rRNA, U6 snRNA, and other small RNAs	2.18164	1.125413	up
1777921_at	NOP19	YGR251W	Ribosome biogenesis factor; nucleolar protein associated with pre-rRNA components of the 90S preribosome, required for cleavage of pre-rRNA at A0, A1 and A2 sites; interacts with RNA helicase Dhr2p and RNA helicase-like protein Utp25p; required for incorporation of Utp25p into preribosomes	2.18035	1.124559	up
1779764_at	ZIP2	YGL249W	Meiosis-specific protein involved in normal synaptonemal complex formation and pairing between homologous chromosomes during meiosis	2.179969	1.124308	up
1771403_at		YKR104W		2.177856	1.122909	up
1771696_at	SPT20	YOL148C	Subunit of the SAGA transcriptional regulatory complex; involved in maintaining the integrity of the complex; mutant displays reduced transcription elongation in the G-less-based run-on (GLRO) assay	2.177125	1.122424	up
1769564_at	UME6	YDR207C	Key transcriptional regulator of early meiotic genes, binds URS1 upstream regulatory sequence, couples metabolic responses to nutritional cues with initiation and progression of meiosis, forms complex with Ime1p, and also with Sin3p-Rpd3p	2.177059	1.122381	up
1775244_at	OMS1	YDR316W	Protein integral to the mitochondrial membrane; has a conserved methyltransferase motif; multicopy suppressor of respiratory defects caused by OXA1 mutations	2.176024	1.121694	up
1778578_at	IWR1	YDL115C	RNA polymerase II transport factor, conserved from yeast to humans; involved in both basal and regulated transcription from RNA polymerase II (RNAP II) promoters, but not itself a transcription factor; interacts with most of the RNAP II subunits; nucleo-cytoplasmic shuttling protein; deletion causes hypersensitivity to K1 killer toxin	2.175937	1.121637	up
1776947_at	MSL1	YIR009W	U2B component of U2 snRNP, involved in splicing, binds the U2 snRNA stem-loop IV in vitro but requires association of Lea1p for in vivo binding; does not contain the conserved C-terminal RNA binding domain found in other family members	2.174637	1.120775	up
1776083_at	VPS27	YNR006W	Endosomal protein that forms a complex with Hse1p; required for recycling Golgi proteins, forming lumenal membranes and sorting ubiquitinated proteins destined for degradation; has Ubiquitin Interaction Motifs which bind ubiquitin (Ubi4p)	2.174176	1.120469	up

1775648_at	FAD1	YDL045C	Flavin adenine dinucleotide (FAD) synthetase, performs the second step in synthesis of FAD from riboflavin	2.17409	1.120411	up
1772312_at		YLR415C		2.173476	1.120004	up
1771410_at	UBP11	YKR098C	Ubiquitin-specific protease that cleaves ubiquitin from ubiquitinated proteins	2.173095	1.119751	up
1779158_at	SSA3	YBL075C	ATPase involved in protein folding and the response to stress; plays a role in SRP-dependent cotranslational protein-membrane targeting and translocation; member of the heat shock protein 70 (HSP70) family; localized to the cytoplasm	2.172313	1.119232	up
1778899_at	HSP78	YDR258C	Oligomeric mitochondrial matrix chaperone that cooperates with Ssc1p in mitochondrial thermotolerance after heat shock; able to prevent the aggregation of misfolded proteins as well as resolubilize protein aggregates	2.171249	1.118525	up
1775918_at	BUR6	YER159C	Subunit of a heterodimeric NC2 transcription regulator complex with Ncb2p; complex binds to TBP and can repress transcription by preventing preinitiation complex assembly or stimulate activated transcription; homologous to human NC2alpha	2.170616	1.118105	up
1775766_at	AZF1	YOR113W	Zinc-finger transcription factor, involved in induction of CLN3 transcription in response to glucose; genetic and physical interactions indicate a possible role in mitochondrial transcription or genome maintenance	2.170437	1.117986	up
1774716_at	GYP7	YDL234C	GTPase-activating protein for yeast Rab family members including: Ypt7p (most effective), Ypt1p, Ypt31p, and Ypt32p (in vitro); involved in vesicle mediated protein trafficking	2.169501	1.117364	up
1774490_at	PEX10	YDR265W	Peroxisomal membrane E3 ubiquitin ligase, required for for Ubc4p-dependent Pex5p ubiquitination and peroxisomal matrix protein import; contains zinc-binding RING domain; mutations in human homolog cause various peroxisomal disorders	2.168268	1.116543	up
1775350_at	SUB1	YMR039C	Transcriptional coactivator, facilitates elongation through factors that modify RNAP II; role in peroxide resistance involving Rad2p; role in the hyperosmotic stress response through polymerase recruitment at RNAP II and RNAP III genes	2.167894	1.116294	up
1777218_at	RPI1	YIL119C	Putative transcriptional regulator; mediates fermentation stress tolerance by modulating cell wall integrity; overexpression suppresses the heat shock sensitivity of wild-type RAS2 overexpression and also suppresses the cell lysis defect of an mpk1 mutation	2.167052	1.115734	up
1769766_at	PHO89	YBR296C	Na+/Pi cotransporter, active in early growth phase; similar to phosphate transporters of Neurospora crassa; transcription regulated by inorganic phosphate concentrations and Pho4p	2.166026	1.115051	up
1780217_at	RTS3	YGR161C	Putative component of the protein phosphatase type 2A complex	2.165616	1.114777	up
1771015_at	HDA3	YPR179C	Subunit of a possibly tetrameric trichostatin A-sensitive class II histone deacetylase complex that contains an Hda1p homodimer and an Hda2p-Hda3p heterodimer; required for the activity of the complex; has similarity to Hda2p	2.165497	1.114698	up
1772091_at	PSP1	YDR505C	Asn and gln rich protein of unknown function; high-copy suppressor of POL1 (DNA polymerase alpha) and partial suppressor of CDC2 (polymerase delta) and CDC6 (pre-RC loading factor) mutations; overexpression results in growth inhibition	2.164539	1.11406	up

1770887_at		YKL187C		2.164501	1.114034	up
1779223_at	YAP1	YML007W	Basic leucine zipper (bZIP) transcription factor required for oxidative stress tolerance; activated by H2O2 through the multistep formation of disulfide bonds and transit from the cytoplasm to the nucleus; mediates resistance to cadmium	2.164127	1.113785	up
1776876_at	HAA1	YPR008W	Transcriptional activator involved in the transcription of TPO2, YRO2, and other genes putatively encoding membrane stress proteins; involved in adaptation to weak acid stress	2.162007	1.112371	up
1779195_at	CTF13	YMR094W	Subunit of the CBF3 complex, which binds to the CDE III element of centromeres, bending the DNA upon binding, and may be involved in sister chromatid cohesion during mitosis	2.161089	1.111759	up
1772185_at	COG5	YNL051W	Component of the conserved oligomeric Golgi complex (Cog1p through Cog8p), a cytosolic tethering complex that functions in protein trafficking to mediate fusion of transport vesicles to Golgi compartments	2.159074	1.110413	up
1773271_at	FRE5	YOR384W	Putative ferric reductase with similarity to Fre2p; expression induced by low iron levels; the authentic, non-tagged protein is detected in highly purified mitochondria in high-throughput studies	2.158111	1.109769	up
1776272_at	ARG7	YMR062C	Mitochondrial ornithine acetyltransferase, catalyzes the fifth step in arginine biosynthesis; also possesses acetylglutamate synthase activity, regenerates acetylglutamate while forming ornithine	2.153038	1.106374	up
1775292_at	PFA3	YNL326C	Palmitoyltransferase for Vac8p, required for vacuolar membrane fusion; contains an Asp-His-His-Cys-cysteine rich (DHHC-CRD) domain; autoacylates; required for vacuolar integrity under stress conditions	2.149763	1.104178	up
1778306_at	DEF1	YKL054C	RNAPII degradation factor, forms a complex with Rad26p in chromatin, enables ubiquitination and proteolysis of RNAPII present in an elongation complex; mutant is deficient in Zip1p loading onto chromosomes during meiosis	2.148104	1.103064	up
1779219_at	SNF7	YLR025W	One of four subunits of the endosomal sorting complex required for transport III (ESCRT-III); involved in the sorting of transmembrane proteins into the multivesicular body (MVB) pathway; recruited from the cytoplasm to endosomal membranes	2.146864	1.102231	up
1774852_at	KRE1	YNL322C	Cell wall glycoprotein involved in beta-glucan assembly; serves as a K1 killer toxin membrane receptor	2.145945	1.101613	up
1775778_at	RPN5	YDL147W	Subunit of the COP9 signalosome (CSN) and non-ATPase regulatory subunit of the 26S proteasome lid, similar to mammalian p55 subunit and to another S. cerevisiae regulatory subunit, Rpn7p; Rpn5p is an essential protein	2.144861	1.100884	up
1770864_at	RAD53	YPL153C	Protein kinase, required for cell-cycle arrest in response to DNA damage; activated by trans autophosphorylation when interacting with hyperphosphorylated Rad9p; also interacts with ARS1 and plays a role in initiation of DNA replication	2.14317	1.099746	up
1770342_at	SEC8	YPR055W	Essential 121kDa subunit of the exocyst complex (Sec3p, Sec5p, Sec6p, Sec8p, Sec10p, Sec15p, Exo70p, and Exo84p), which has the essential function of mediating polarized targeting of secretory vesicles to active sites of exocytosis	2.141808	1.098829	up
1773979_at	RPT2	YDL007W	One of six ATPases of the 19S regulatory particle of the 26S proteasome involved in the degradation of ubiquitinated substrates; required for normal	2.141516	1.098632	up

			peptide hydrolysis by the core 20S particle			
1777184_at	DCV1	YFR012W	Protein of unknown function; deletion mutant shows strong genetic interaction with cdc28-as1 mutant in the presence of 1-NM-PP1	2.14044	1.097908	up
1771068_at	PSO2	YMR137C	Nuclease required for a post-incision step in the repair of DNA single and double-strand breaks that result from interstrand crosslinks produced by a variety of mono- and bi-functional psoralen derivatives; induced by UV-irradiation	2.140014	1.09762	up
1779371_at		YIR018C-A		2.139332	1.097161	up
1773938_at	SRX1	YKL086W	Sulfiredoxin, contributes to oxidative stress resistance by reducing cysteine-sulfinic acid groups in the peroxiredoxin Tsa1p, which is formed upon exposure to oxidants; conserved in higher eukaryotes	2.137379	1.095843	up
1774693_at	KRI1	YNL308C	Essential nucleolar protein required for 40S ribosome biogenesis; associate with snR30; physically and functionally interacts with Krr1p	2.136209	1.095053	up
1771692_at	PDC6	YGR087C	Minor isoform of pyruvate decarboxylase, decarboxylates pyruvate to acetaldehyde, involved in amino acid catabolism; transcription is glucose- and ethanol-dependent, and is strongly induced during sulfur limitation	2.133999	1.093559	up
1771671_at		YDL159W-A		2.133909	1.093499	up
1771265_at	FMP46	YKR049C	Putative redox protein containing a thioredoxin fold; the authentic, non-tagged protein is detected in highly purified mitochondria in high-throughput studies	2.133368	1.093133	up
1775590_at	IML3	YBR107C	Protein with a role in kinetochore function, localizes to the outer kinetochore in a Ctf19p-dependent manner, interacts with Chl4p and Ctf19p	2.133292	1.093082	up
1769575_at	VPS60	YDR486C	Cytoplasmic and vacuolar membrane protein involved in late endosome to vacuole transport; required for normal filament maturation during pseudohyphal growth; may function in targeting cargo proteins for degradation; interacts with Vta1p	2.132042	1.092236	up
1775255_at	PRM8	YGL053W	Pheromone-regulated protein with 2 predicted transmembrane segments and an FF sequence, a motif involved in COPII binding; forms a complex with Prp9p in the ER; member of DUP240 gene family	2.130979	1.091516	up
1779618_at	GZF3	YJL110C	GATA zinc finger protein and Dal80p homolog that negatively regulates nitrogen catabolic gene expression by competing with Gat1p for GATA site binding; function requires a repressive carbon source; dimerizes with Dal80p and binds to Tor1p	2.129476	1.090498	up
1770958_at	UBX7	YBR273C	UBX (ubiquitin regulatory X) domain-containing protein that interacts with Cdc48p	2.12816	1.089606	up
1780052_at		YJR142W		2.127477	1.089143	up
1774710_at	YMC2	YBR104W	Mitochondrial protein, putative inner membrane transporter with a role in oleate metabolism and glutamate biosynthesis; member of the mitochondrial carrier (MCF) family; has similarity with Ymc1p	2.12653	1.088501	up
1769913_s_at	THI13	YDL244W	Protein involved in synthesis of the thiamine precursor hydroxymethylpyrimidine (HMP)	2.12478	1.087313	up
1774696_at	HSP31	YDR533C	Possible chaperone and cysteine protease with similarity to E. coli Hsp31; member of the DJ-1/ThiJ/PfpI superfamily, which includes human DJ-1 involved in Parkinson's disease; exists as a dimer and contains a putative metal-binding site	2.119847	1.08396	up
1776529_at	SPC105	YGL093W	Subunit of a kinetochore-microtubule binding complex with Kre28p that bridges centromeric heterochromatin and kinetochore MAPs and	2.118289	1.0829	up

			motors; required for sister chromatid bi-orientation and kinetochore binding of SAC components			
1778352_at	MFT1	YML062C	Subunit of the THO complex, which is a nuclear complex comprised of Hpr1p, Mft1p, Rlr1p, and Thp2p, that is involved in transcription elongation and mitotic recombination; involved in telomere maintenance	2.118223	1.082854	up
1769456_at	BLI1	YKL061W	Putative protein of unknown function; likely member of BLOC complex involved in endosomal cargo sorting; green fluorescent protein (GFP)-fusion protein localizes to the endosome	2.117698	1.082497	up
1771702_at	DAL5	YJR152W	Allantoate permease; ureidosuccinate permease; also transports dipeptides, though with lower affinity than for allantoate and ureidosuccinate; expression is constitutive but sensitive to nitrogen catabolite repression	2.116076	1.081391	up
1779403_at		YER158C		2.116002	1.081341	up
1769464_at		YGR130C		2.114601	1.080385	up
1777945_at	TPO2	YGR138C	Polyamine transport protein specific for spermine; localizes to the plasma membrane; transcription of TPO2 is regulated by Haa1p; member of the major facilitator superfamily	2.113282	1.079485	up
1777418_at	SMB1	YER029C	Core Sm protein Sm B; part of heteroheptameric complex (with Smd1p, Smd2p, Smd3p, Sme1p, Smx3p, and Smx2p) that is part of the spliceosomal U1, U2, U4, and U5 snRNPs; homolog of human Sm B and Sm B'	2.112955	1.079262	up
1777455_at	ARG82	YDR173C	Inositol polyphosphate multikinase (IPMK), sequentially phosphorylates Ins(1,4,5)P3 to form Ins(1,3,4,5,6)P5; also has diphosphoinositol polyphosphate synthase activity; regulates arginine-, phosphate-, and nitrogen-responsive genes	2.110735	1.077745	up
1772365_at		YLR108C		2.108676	1.076337	up
1777092_at	RNH70	YGR276C	3'-5' exoribonuclease; required for maturation of 3' ends of 5S rRNA and tRNA-Arg3 from dicistronic transcripts	2.108256	1.07605	up
1777383_at	ECM3	YOR092W	Non-essential protein of unknown function; involved in signal transduction and the genotoxic response; induced rapidly in response to treatment with 8-methoxypsoralen and UVA irradiation	2.108139	1.07597	up
1780069_at		YKR005C		2.104493	1.073473	up
1773758_at	SNF3	YDL194W	Plasma membrane low glucose sensor that regulates glucose transport; contains 12 predicted transmembrane segments and a long C-terminal tail required for induction of hexose transporters; also senses fructose and mannose; similar to Rgt2p	2.10448	1.073463	up
1778357_at	PRM4	YPL156C	Pheromone-regulated protein proposed to be involved in mating; predicted to have 1 transmembrane segment; transcriptionally regulated by Ste12p during mating and by Cat8p during the diauxic shift	2.104378	1.073394	up
1779691_at	RPN3	YER021W	Essential, non-ATPase regulatory subunit of the 26S proteasome lid, similar to the p58 subunit of the human 26S proteasome; temperature-sensitive alleles cause metaphase arrest, suggesting a role for the proteasome in cell cycle control	2.102878	1.072365	up
1769451_at	LEU5	YHR002W	Mitochondrial carrier protein involved in the accumulation of CoA in the mitochondrial matrix; homolog of human Graves disease protein; does not encode an isozyme of Leu4p, as first hypothesized	2.102765	1.072288	up
1769885_at	ELO1	YJL196C	Elongase I, medium-chain acyl elongase, catalyzes carboxy-terminal elongation of unsaturated C12-C16 fatty acyl-CoAs to C16-C18 fatty acids	2.102216	1.071911	up
1775177_at	PEX17	YNL214W	Peroxisomal membrane peroxin and subunit of the	2.099572	1.070096	up

			docking complex that facilitates the import of peroxisomal matrix proteins; required for peroxisome biogenesis			
1775851_at	GPX1	YKL026C	Phospholipid hydroperoxide glutathione peroxidase induced by glucose starvation that protects cells from phospholipid hydroperoxides and nonphospholipid peroxides during oxidative stress	2.099055	1.06974	up
1777553_s_at		YFL065C	Putative protein of unknown function; induced by treatment with 8-methoxypsoralen and UVA irradiation	2.097316	1.068544	up
1778437_at	AFR1	YDR085C	Protein required for pheromone-induced projection (shmoo) formation; regulates septin architecture during mating; has an RVXF motif that mediates targeting of Glc7p to mating projections; interacts with Cdc12p	2.095472	1.067276	up
1777617_at	TGL2	YDR058C	Triacylglycerol lipase that is localized to the mitochondria; has lipolytic activity towards triacylglycerols and diacylglycerols when expressed in E. coli	2.094338	1.066495	up
1776371_at	SKM1	YOL113W	Member of the PAK family of serine/threonine protein kinases with similarity to Ste20p and Cla4p; involved in down-regulation of sterol uptake; proposed to be a downstream effector of Cdc42p during polarized growth	2.089433	1.063111	up
1770899_at	SKO1	YNL167C	Basic leucine zipper transcription factor of the ATF/CREB family; forms a complex with Tup1p and Cyc8p to both activate and repress transcription; cytosolic and nuclear protein involved in osmotic and oxidative stress responses	2.088807	1.062679	up
1777882_at	LEA1	YPL213W	Component of U2 snRNP; disruption causes reduced U2 snRNP levels; physically interacts with Msl1p; putative homolog of human U2A' snRNP protein	2.087245	1.0616	up
1774570_at	AFT1	YGL071W	Transcription factor involved in iron utilization and homeostasis; binds the consensus site PyPuCACCCPu and activates the expression of target genes in response to changes in iron availability; in iron-replete conditions activity is negatively regulated by Grx3p, Grx4p, and Fra2p, which regulate Aft1p translocation from the nucleus to the cytoplasm	2.086705	1.061226	up
1774478_at	PCL10	YGL134W	Pho85p cyclin; recruits, activates, and targets Pho85p cyclin-dependent protein kinase to its substrate	2.086045	1.06077	up
1777350_at	BDH1	YAL060W	NAD-dependent (R,R)-butanediol dehydrogenase, catalyzes oxidation of (R,R)-2,3-butanediol to (3R)-acetoin, oxidation of meso-butanediol to (3S)-acetoin, and reduction of acetoin; enhances use of 2,3-butanediol as an aerobic carbon source	2.085122	1.060132	up
1778220_at		YPL229W		2.083129	1.058753	up
1779961_at	ERG5	YMR015C	C-22 sterol desaturase, a cytochrome P450 enzyme that catalyzes the formation of the C-22(23) double bond in the sterol side chain in ergosterol biosynthesis; may be a target of azole antifungal drugs	2.083041	1.058691	up
1769324_at		YJL147C		2.08187	1.05788	up
1773829_at	MPH2	YDL247W	Alpha-glucoside permease, transports maltose, maltotriose, alpha-methylglucoside, and turanose; identical to Mph3p; encoded in a subtelomeric position in a region likely to have undergone duplication	2.081202	1.057417	up
1771514_at	CIN1	YOR349W	Tubulin folding factor D involved in beta-tubulin (Tub2p) folding; isolated as mutant with increased chromosome loss and sensitivity to benomyl	2.080437	1.056886	up
1773891_at	EXO1	YOR033C	5'-3' exonuclease and flap-endonuclease involved in	2.079058	1.05593	up

			recombination, double-strand break repair and DNA mismatch repair; member of the Rad2p nuclease family, with conserved N and I nuclease domains			
1771230_at	NSE3	YDR288W	Component of the SMC5-SMC6 complex; this complex plays a key role in the removal of X-shaped DNA structures that arise between sister chromatids during DNA replication and repair	2.078341	1.055432	up
1776978_at		YLR290C		2.076676	1.054277	up
1779679_s_at	FLO9	YAL063C	Lectin-like protein with similarity to Flo1p, thought to be expressed and involved in flocculation	2.076027	1.053825	up
1775190_at		YMR209C		2.071656	1.050785	up
1779724_at	VFA1	YER128W	Protein that interacts with Vps4p and has a role in vacuolar sorting; localizes to endosomes in a Vps4-dependent manner; overexpression causes canavanine sensitivity and confers a partial class D vacuole morphology	2.07127	1.050516	up
1775136_at	MTG1	YMR097C	Putative GTPase peripheral to the mitochondrial inner membrane, essential for respiratory competence, likely functions in assembly of the large ribosomal subunit, has homologs in plants and animals	2.069681	1.049408	up
1772884_at		YPR013C		2.067902	1.048168	up
1775555_at	VPS52	YDR484W	Component of the GARP (Golgi-associated retrograde protein) complex, Vps51p-Vps52p-Vps53p-Vps54p, which is required for the recycling of proteins from endosomes to the late Golgi; involved in localization of actin and chitin	2.067094	1.047604	up
1775922_at	ARA2	YMR041C	NAD-dependent arabinose dehydrogenase, involved in biosynthesis of dehydro-D-arabinono-1,4-lactone; similar to plant L-galactose dehydrogenase	2.066921	1.047483	up
1774992_at		YKL105C		2.066466	1.047166	up
1778955_at	ESA1	YOR244W	Catalytic subunit of the histone acetyltransferase complex (NuA4) that acetylates four conserved internal lysines of histone H4 N-terminal tail; required for cell cycle progression and transcriptional silencing at the rDNA locus	2.066156	1.046949	up
1777609_at	CNN1	YFR046C	Kinetochore protein of unknown function; associated with the essential kinetochore proteins Nnf1p and Spc24p; phosphorylated by both Clb5-Cdk1 and, to a lesser extent, Clb2-Cdk1.	2.063877	1.045357	up
1770785_at	HPA2	YPR193C	Tetrameric histone acetyltransferase with similarity to Gcn5p, Hat1p, Elp3p, and Hpa3p; acetylates histones H3 and H4 in vitro and exhibits autoacetylation activity	2.062473	1.044375	up
1779330_at	PIF1	YML061C	DNA helicase; exists in a nuclear form that acts as a catalytic inhibitor of telomerase; and as a mitochondrial form involved in repair and recombination of mitochondrial DNA; mutations affect zinc and iron homeostasis	2.061828	1.043924	up
1778748_at	COT1	YOR316C	Vacuolar transporter that mediates zinc transport into the vacuole; overexpression confers resistance to cobalt and rhodium	2.061072	1.043395	up
1775610_s_at	HMRA2	YCR096C	Silenced copy of a2 at HMR; similarity to Alpha2p; required along with a1p for inhibiting expression of the HO endonuclease in a/alpha HO/HO diploid cells with an active mating-type interconversion system	2.060334	1.042878	up
1778871_at	DAS1	YJL149W	Putative SCF ubiquitin ligase F-box protein; interacts physically with both Cdc53p and Skp1 and genetically with CDC34; similar to putative F-box protein YDR131C	2.060197	1.042782	up
1772786_at	AIM5	YBR262C	Mitochondrial inner membrane protein; subunit of	2.060119	1.042728	up

			the mitochondrial inner membrane organizing system (MitOS, MICOS, or MINOS), a scaffold-like structure on the intermembrane space side of the inner membrane which has a role in the maintenance of crista junctions and inner membrane architecture			
1772005_at	ECM21	YBL101C	Protein involved in regulating the endocytosis of plasma membrane proteins; identified as a substrate for ubiquitination by Rsp5p and deubiquitination by Ubp2p; promoter contains several Gcn4p binding elements	2.058768	1.041781	up
1778884_at	HEF3	YNL014W	Translational elongation factor EF-3; paralog of YEF3 and member of the ABC superfamily; stimulates EF-1 alpha-dependent binding of aminoacyl-tRNA by the ribosome; normally expressed in zinc deficient cells	2.054186	1.038567	up
1779118_at	RRG8	YPR116W	Putative protein of unknown function, required for mitochondrial genome maintenance; null mutation results in a decrease in plasma membrane electron transport	2.051748	1.036853	up
1773057_at	SMC6	YLR383W	Component of the SMC5-SMC6 complex; this complex plays a key role in the removal of X-shaped DNA structures that arise between sister chromatids during DNA replication and repair; homologous to S. pombe rad18	2.051465	1.036655	up
1769467_at	ECM2	YBR065C	Pre-mRNA splicing factor, facilitates the cooperative formation of U2/U6 helix II in association with stem II in the spliceosome, function may be regulated by Slu7p	2.051437	1.036635	up
1779643_at	ARO3	YDR035W	3-deoxy-D-arabino-heptulosonate-7-phosphate (DAHP) synthase, catalyzes the first step in aromatic amino acid biosynthesis and is feedback-inhibited by phenylalanine or high concentration of tyrosine or tryptophan	2.050476	1.035959	up
1778024_at	AMA1	YGR225W	Activator of meiotic anaphase promoting complex (APC/C); Cdc20p family member; required for initiation of spore wall assembly; required for Clb1p degradation during meiosis	2.049392	1.035196	up
1778623_at	STN1	YDR082W	Telomere end-binding and capping protein, plays a key role with Pol12p in linking telomerase action with completion of lagging strand synthesis, and in a regulatory step required for telomere capping	2.049072	1.03497	up
1778931_at		YJR096W		2.048714	1.034719	up
1770773_at	PUT3	YKL015W	Transcriptional activator of proline utilization genes, constitutively binds PUT1 and PUT2 promoter sequences as a dimer and undergoes a conformational change to form the active state; differentially phosphorylated in the presence of different nitrogen sources; has a Zn(2)-Cys(6) binuclear cluster domain	2.048574	1.03462	up
1771446_at	RXT3	YDL076C	Subunit of the RPD3L complex; involved in histone deacetylation	2.048448	1.034531	up
1773524_at	GPI12	YMR281W	ER membrane protein involved in the second step of glycosylphosphatidylinositol (GPI) anchor assembly, the de-N-acetylation of the N-acetylglucosaminylphosphatidylinositol intermediate; functional homolog of human PIG-Lp	2.048091	1.03428	up
1777299_at	MPS2	YGL075C	Essential membrane protein localized at the nuclear envelope and spindle pole body (SPB), required for insertion of the newly duplicated SPB into the nuclear envelope; potentially phosphorylated by Cdc28p	2.047643	1.033964	up
1774564_at	HSP104	YLL026W	Heat shock protein that cooperates with Ydj1p (Hsp40) and Ssa1p (Hsp70) to refold and reactivate previously denatured, aggregated proteins; responsive to stresses including: heat, ethanol, and	2.044693	1.031884	up

			sodium arsenite; involved in [PSI+] propagation			
1771147_at	SGM1	YJR134C	Protein of unknown function, required for wild-type growth rate on galactose and mannose; localizes to COPI coated vesicles and the Golgi apparatus	2.043375	1.030954	up
1777692_at	RRN9	YMR270C	Protein involved in promoting high level transcription of rDNA, subunit of UAF (upstream activation factor) for RNA polymerase I	2.042282	1.030182	up
1777726_at	MER1	YNL210W	Protein with RNA-binding motifs required for meiosis-specific mRNA splicing; required for chromosome pairing and meiotic recombination; Mer1p regulon embraces four essential meiotic pre-mRNAs: REC107, HFM1, AMA1 and SPO22	2.041474	1.029611	up
1776017_at	SPT3	YDR392W	Subunit of the SAGA and SAGA-like transcriptional regulatory complexes, interacts with Spt15p to activate transcription of some RNA polymerase II-dependent genes, also functions to inhibit transcription at some promoters	2.038852	1.027757	up
1769647_at	DOS2	YDR068W	Protein of unknown function, green fluorescent protein (GFP)-fusion protein localizes to the cytoplasm	2.038472	1.027488	up
1772516_at	GIS1	YDR096W	JmjC domain-containing histone demethylase and transcription factor; involved in expression of genes during nutrient limitation; negatively regulates DPP1 and PHR1; activity is modulated by limited proteasome-mediated proteolysis; has a JmjC and a JmjN domain in the N-terminal region that interact, promoting Gis1p stability and proper transcriptional activity; contains transactivating domains TAD1 and TAD2 downstream of the Jmj domains and a C-terminal DNA binding domain	2.037299	1.026658	up
1771651_at	UBC6	YER100W	Ubiquitin-conjugating enzyme involved in ER-associated protein degradation; located at the cytosolic side of the ER membrane; tail region contains a transmembrane segment at the C-terminus; substrate of the ubiquitin-proteasome pathway	2.037184	1.026576	up
1774507_at	ROD1	YOR018W	Membrane protein that binds the ubiquitin ligase Rsp5p via its 2 PY motifs; overexpression confers resistance to the GST substrate o-dinitrobenzene,zinc, and calcium; proposed to regulate the endocytosis of plasma membrane proteins	2.035734	1.025549	up
1772635_at	DAL82	YNL314W	Positive regulator of allophanate inducible genes; binds a dodecanucleotide sequence upstream of all genes that are induced by allophanate; contains an UISALL DNA-binding, a transcriptional activation, and a coiled-coil domain	2.032322	1.023129	up
1779019_at	RIM4	YHL024W	Putative RNA-binding protein required for the expression of early and middle sporulation genes	2.031478	1.02253	up
1773654_at	AOS1	YPR180W	Subunit of a heterodimeric nuclear SUMO activating enzyme (E1) with Uba2p; activates Smt3p (SUMO) before its conjugation to proteins (sumoylation), which may play a role in protein targeting; essential for viability	2.030284	1.021682	up
1773929_at	TEP1	YNL128W	PTEN homolog with no demonstrated inositol lipid phosphatase activity; plays a role in normal sporulation; homolog of human tumor suppressor gene PTEN/MMAC1/TEP1 and fission yeast ptn1	2.029875	1.021391	up
1774403_at		YLR177W		2.02952	1.021138	up
1773640_at	BAP3	YDR046C	Amino acid permease involved in the uptake of cysteine, leucine, isoleucine and valine	2.029228	1.020931	up
1770988_at	HLJ1	YMR161W	Co-chaperone for Hsp40p, anchored in the ER membrane; with its homolog Ydj1p promotes ER-associated protein degradation (ERAD) of integral	2.027278	1.019544	up

			membrane substrates; similar to E. coli DnaJ			
1770280_at	APM1	YPL259C	Mu1-like medium subunit of the clathrin-associated protein complex (AP-1); binds clathrin; involved in clathrin-dependent Golgi protein sorting	2.025859	1.018534	up
1778920_at		YOR032W-A		2.025202	1.018066	up
1770908_at	TAF11	YML015C	TFIID subunit (40 kDa), involved in RNA polymerase II transcription initiation, similar to histone H3 with atypical histone fold motif of Spt3-like transcription factors	2.024724	1.017725	up
1770050_at		YOL036W		2.020404	1.014644	up
1773500_at		YOR012W		2.017303	1.012428	up
1779372_at	KAR1	YNL188W	Essential protein involved in karyogamy during mating and in spindle pole body duplication during mitosis, localizes to the half-bridge of the spindle pole body, interacts with Spc72p during karyogamy, also interacts with Cdc31p	2.013574	1.009759	up
1776790_at	VPS17	YOR132W	Subunit of the membrane-associated retromer complex essential for endosome-to-Golgi retrograde protein transport; peripheral membrane protein that assembles onto the membrane with Vps5p to promote vesicle formation	2.012234	1.008798	up
1777137_at		YGR109W-B		2.011825	1.008505	up
1775277_at	MEH1	YKR007W	Component of the EGO complex, which is involved in the regulation of microautophagy, and of the GSE complex, which is required for proper sorting of amino acid permease Gap1p; loss results in a defect in vacuolar acidification	2.010852	1.007807	up
1774850_at	TAF3	YPL011C	TFIID subunit (47 kDa), involved in promoter binding and RNA polymerase II transcription initiation	2.010443	1.007513	up
1777212_at	PCI8	YIL071C	Possible shared subunit of Cop9 signalosome (CSN) and eIF3, binds eIF3b subunit Prt1p, has possible dual functions in transcriptional and translational control, contains a PCI (Proteasome-COP9 signalosome (CSN)-eIF3) domain	2.01009	1.00726	up
1771536_at	AST2	YER101C	Similar to lipid raft associated protein Ast1p; similarly to Ast1p, overexpression restores Pma1p localization to lipid rafts which is required for targeting of Pma1p to the plasma membrane; sometimes classified in the medium-chain dehydrogenase/reductases (MDRs) superfamily	2.008026	1.005778	up
1778363_at	RAD5	YLR032W	DNA helicase proposed to promote replication fork regression during postreplication repair by template switching; RING finger containing ubiquitin ligase; stimulates the synthesis of free and PCNA-bound polyubiquitin chains by Ubc13p-Mms2p	2.007437	1.005355	up
1777611_at	ERP6	YGL002W	Protein with similarity to Emp24p and Erv25p, member of the p24 family involved in ER to Golgi transport; the authentic, non-tagged protein is detected in highly purified mitochondria in high-throughput studies	2.005448	1.003924	up
1770102_at	ORC6	YHR118C	Subunit of the origin recognition complex, which directs DNA replication by binding to replication origins and is also involved in transcriptional silencing; phosphorylated by Cdc28p	2.004625	1.003333	up
1775797_at	PEX2	YJL210W	RING-finger peroxin and E3 ubiquitin ligase, peroxisomal membrane protein with a C-terminal zinc-binding RING domain, forms translocation subcomplex with Pex10p and Pex12p which functions in peroxisomal matrix protein import	2.003038	1.00219	up
1772376_at	ARL3	YPL051W	GTPase of the Ras superfamily required to recruit Arl1p to the Golgi; similar to ADP-ribosylation factor	2.002512	1.001811	up

1770397_at		YDR262W		2.0022	1.001586	up
1771800_at		YPL260W		2.001245	1.000897	up
1776473_at	SEC9	YGR009C	t-SNARE protein important for fusion of secretory vesicles with the plasma membrane; similar to but not functionally redundant with Spo20p; SNAP-25 homolog	2.000636	1.000459	up
1776139_at		YOR131C		2.000245	1.000177	up
1772870_at	URK1	YNR012W	Uridine/cytidine kinase, component of the pyrimidine ribonucleotide salvage pathway that converts uridine into UMP and cytidine into CMP; involved in the pyrimidine deoxyribonucleotide salvage pathway, converting deoxycytidine into dCMP	-2.0005	-1.00036	down
1772921_at		YLR352W		-2.00168	-1.00121	down
1775764_at		YBL055C		-2.00197	-1.00142	down
1770776_at	SBH2	YER019C-A	Ssh1p-Sss1p-Sbh2p complex component, involved in protein translocation into the endoplasmic reticulum; homologous to Sbh1p	-2.00215	-1.00155	down
1769776_at	GPA1	YHR005C	GTP-binding alpha subunit of the heterotrimeric G protein that couples to pheromone receptors; negatively regulates the mating pathway by sequestering G(beta)gamma and by triggering an adaptive response; activates Vps34p at the endosome	-2.00443	-1.00319	down
1772947_at	RPL20B	YOR312C	Protein component of the large (60S) ribosomal subunit, nearly identical to Rpl20Ap and has similarity to rat L18a ribosomal protein	-2.00516	-1.00372	down
1779942_at	SOL2	YCR073W-A	Protein with a possible role in tRNA export; shows similarity to 6-phosphogluconolactonase non-catalytic domains but does not exhibit this enzymatic activity; homologous to Sol1p, Sol3p, and Sol4p	-2.00621	-1.00447	down
1770569_at	RPS21A	YKR057W	Protein component of the small (40S) ribosomal subunit; nearly identical to Rps21Bp and has similarity to rat S21 ribosomal protein	-2.00622	-1.00448	down
1778954_at	RPE1	YJL121C	D-ribulose-5-phosphate 3-epimerase, catalyzes a reaction in the non-oxidative part of the pentose-phosphate pathway; mutants are sensitive to oxidative stress	-2.00894	-1.00643	down
1770637_at	UIP5	YKR044W	Protein of unknown function that interacts with Ulp1p, a Ubl (ubiquitin-like protein)-specific protease for Smt3p protein conjugates	-2.00922	-1.00664	down
1770297_at	UTP14	YML093W	Subunit of U3-containing Small Subunit (SSU) processome complex involved in production of 18S rRNA and assembly of small ribosomal subunit	-2.0094	-1.00676	down
1776379_at	WWM1	YFL010C	WW domain containing protein of unknown function; binds to Mca1p, a caspase-related protease that regulates H2O2-induced apoptosis; overexpression causes G1 phase growth arrest and clonal death that is suppressed by overexpression of MCA1	-2.01048	-1.00754	down
1771816_at	MRI1	YPR118W	5'-methylthioribose-1-phosphate isomerase; catalyzes the isomerization of 5-methylthioribose-1-phosphate to 5-methylthioribulose-1-phosphate in the methionine salvage pathway	-2.01108	-1.00797	down
1771954_at	PKC1	YBL105C	Protein serine/threonine kinase essential for cell wall remodeling during growth; localized to sites of polarized growth and the mother-daughter bud neck; homolog of the alpha, beta, and gamma isoforms of mammalian protein kinase C (PKC)	-2.0135	-1.0097	down
1777567_at	HMT1	YBR034C	Nuclear SAM-dependent mono- and asymmetric arginine dimethylating methyltransferase that modifies hnRNPs, including Npl3p and Hrp1p, affecting their activity and nuclear export;	-2.0158	-1.01135	down

			methylates U1 snRNP protein Snp1p and ribosomal protein Rps2p			
1769628_at	SEY1	YOR165W	Dynamin-like GTPase that mediates homotypic ER fusion; has a role in ER morphology; interacts physically and genetically with Yop1p and Rtn1p; functional ortholog of the human atlastin ATL1, defects in which cause a form of the human disease hereditary spastic paraplegia; homolog of Arabidopsis RHD3	-2.01621	-1.01165	down
1772606_at	MRS6	YOR370C	Rab escort protein, forms a complex with the Ras-like small GTPase Ypt1p that is required for the prenylation of Ypt1p by protein geranylgeranyltransferase type II (Bet2p-Bet4p); sequence similarity to mammalian choroideraemia gene	-2.01713	-1.01231	down
1779468_at	URA4	YLR420W	Dihydroorotase, catalyzes the third enzymatic step in the de novo biosynthesis of pyrimidines, converting carbamoyl-L-aspartate into dihydroorotate	-2.01886	-1.01354	down
1775560_at	ECM27	YJR106W	Putative protein of unknown function; may play a role in cell wall biosynthesis, mutants are hypersensitive to Papulacandin B; null mutants have increased plasmid loss; displays a two-hybrid interaction with Pdr5p	-2.02043	-1.01466	down
1774468_at	GCN2	YDR283C	Protein kinase, phosphorylates the alpha-subunit of translation initiation factor eIF2 (Sui2p) in response to starvation; activated by uncharged tRNAs and the Gcn1p-Gcn20p complex; contributes to DNA damage checkpoint control	-2.02276	-1.01632	down
1778095_at	SPR6	YER115C	Protein of unknown function, expressed during sporulation; not required for sporulation, but gene exhibits genetic interactions with other genes required for sporulation	-2.02365	-1.01696	down
1771502_at	RPL43A	YPR043W	Protein component of the large (60S) ribosomal subunit, identical to Rpl43Bp and has similarity to rat L37a ribosomal protein; null mutation confers a dominant lethal phenotype	-2.02466	-1.01768	down
1771312_at	ACE2	YLR131C	Transcription factor required for septum destruction after cytokinesis; phosphorylation by Cbk1p blocks nuclear exit of Ace2p during the M-to-G1 transition, causing its specific localization to daughter cell nuclei, and also increases Ace2p activity; phosphorylation by Cdc28p and Pho85p prevents nuclear import during cell cycle phases other than cytokinesis; part of the RAM network that regulates cellular polarity and morphogenesis	-2.02596	-1.01861	down
1770690_at	SLM3	YDL033C	tRNA-specific 2-thiouridylase, responsible for 2-thiolation of the wobble base of mitochondrial tRNAs; human ortholog is implicated in myoclonus epilepsy associated with ragged red fibers (MERRF)	-2.02697	-1.01932	down
1773902_at	IRC22	YEL001C	Putative protein of unknown function; green fluorescent protein (GFP)-fusion localizes to the ER; YEL001C is non-essential; null mutant displays increased levels of spontaneous Rad52p foci	-2.02714	-1.01944	down
1770572_at	BCH1	YMR237W	Member of the ChAPs family (Chs5p-Arf1p-binding proteins: Bch1p, Bch2p, Bud7p, Chs6p), that forms the exomer complex with Chs5p to mediate export of specific cargo proteins from the Golgi to the plasma membrane; may interact with ribosomes	-2.02715	-1.01945	down
1769959_at	NDE2	YDL085W	Mitochondrial external NADH dehydrogenase, catalyzes the oxidation of cytosolic NADH; Nde1p and Nde2p are involved in providing the cytosolic NADH to the mitochondrial respiratory chain	-2.02767	-1.01982	down

1770820_at	WRS1	YOL097C	Cytoplasmic tryptophanyl-tRNA synthetase, aminoacylates tryptophanyl-tRNA	-2.02921	-1.02092	down
1773353_at	PAB1	YER165W	Poly(A) binding protein, part of the 3'-end RNA-processing complex, mediates interactions between the 5' cap structure and the 3' mRNA poly(A) tail, involved in control of poly(A) tail length, interacts with translation factor eIF-4G	-2.02962	-1.02121	down
1775982_at		YHR045W		-2.02976	-1.02131	down
1774111_at	CLU1	YMR012W	eIF3 component of unknown function; deletion causes defects in mitochondrial organization but not in growth or translation initiation, can rescue cytokinesis and mitochondrial organization defects of the Dictyostelium cluA- mutant	-2.03058	-1.02189	down
1775518_at	QNS1	YHR074W	Glutamine-dependent NAD(+) synthetase, essential for the formation of NAD(+) from nicotinic acid adenine dinucleotide	-2.031	-1.02219	down
1775594_s_at	RPL42B	YHR141C	Protein component of the large (60S) ribosomal subunit, identical to Rpl42Ap and has similarity to rat L44; required for propagation of the killer toxin-encoding M1 double-stranded RNA satellite of the L-A double-stranded RNA virus	-2.03116	-1.0223	down
1774703_at	DIB1	YPR082C	17-kDa component of the U4/U6aU5 tri-snRNP, plays an essential role in pre-mRNA splicing, orthologue of hDIM1, the human U5-specific 15-kDa protein	-2.03196	-1.02287	down
1771227_at	GBP2	YCL011C	Poly(A+) RNA-binding protein, involved in the export of mRNAs from the nucleus to the cytoplasm; similar to Hrb1p and Npl3p; also binds single-stranded telomeric repeat sequence in vitro	-2.03272	-1.02341	down
1774670_at		YPL108W		-2.03338	-1.02388	down
1775492_at	NAT1	YDL040C	Subunit of the N-terminal acetyltransferase NatA (Nat1p, Ard1p, Nat5p); N-terminally acetylates many proteins, which influences multiple processes such as the cell cycle, heat-shock resistance, mating, sporulation, and telomeric silencing	-2.0337	-1.02411	down
1778696_at	SEC11	YIR022W	18kDa catalytic subunit of the Signal Peptidase Complex (SPC; Spc1p, Spc2p, Spc3p, and Sec11p) which cleaves the signal sequence of proteins targeted to the endoplasmic reticulum	-2.03574	-1.02555	down
1771125_at	PAD1	YDR538W	Phenylacrylic acid decarboxylase, confers resistance to cinnamic acid, decarboxylates aromatic carboxylic acids to the corresponding vinyl derivatives; also has mRNA binding activity; homolog of E. coli UbiX	-2.03692	-1.02639	down
1772594_at	TCB2	YNL087W	Bud-specific protein with a potential role in membrane trafficking; localizes to endoplasmic reticulum and is enriched at ER-plasma membrane contact sites; GFP-fusion protein migrates from the cell surface to intracellular vesicles near vacuole; contains 3 calcium and lipid binding domains; mRNA is targeted to the bud	-2.03802	-1.02717	down
1772617_at	GPI16	YHR188C	Transmembrane protein subunit of the glycosylphosphatidylinositol transamidase complex that adds GPIs to newly synthesized proteins; human PIG-Tp homolog	-2.04012	-1.02866	down
1775706_at	NUP188	YML103C	Subunit of the inner ring of the nuclear pore complex (NPC); contributes to NPC organization and nucleocytoplasmic transport; homologous to human NUP188	-2.04354	-1.03107	down
1775856_at	TRM1	YDR120C	tRNA methyltransferase; two forms of the protein are made by alternative translation starts; localizes to both the nucleus and mitochondrion to produce the modified base N2,N2-dimethylguanosine in tRNAs in both compartments	-2.04375	-1.03122	down
1772703_at	TAF13	YML098W	TFIID subunit (19 kDa), involved in RNA	-2.04399	-1.03139	down

			polymerase II transcription initiation, similar to histone H4 with atypical histone fold motif of Spt3-like transcription factors			
1770256_at	EMC2	YJR088C	Member of a transmembrane complex required for efficient folding of proteins in the ER; null mutant displays induction of the unfolded protein response	-2.04473	-1.03191	down
1777126_at	BEM2	YER155C	Rho GTPase activating protein (RhoGAP) involved in the control of cytoskeleton organization and cellular morphogenesis; required for bud emergence	-2.04651	-1.03317	down
1776702_at	TAO3	YIL129C	Component of the RAM signaling network that is involved in regulation of Ace2p activity and cellular morphogenesis, interacts with protein kinase Cbk1p and also with Kic1p	-2.04906	-1.03496	down
1777988_at	PXR1	YGR280C	Essential protein involved in rRNA and snoRNA maturation; competes with TLC1 RNA for binding to Est2p, suggesting a role in negative regulation of telomerase; human homolog inhibits telomerase; contains a G-patch RNA interacting domain	-2.04951	-1.03528	down
1778823_at	HOP1	YIL072W	Meiosis-specific DNA binding protein that displays Red1p dependent localization to the unsynapsed axial-lateral elements of the synaptonemal complex; required for homologous chromosome synapsis and chiasma formation	-2.05	-1.03562	down
1777799_at	COA3	YJL062W-A	Mitochondrial inner membrane protein that participates in regulation of COX1 translation, Cox1p stabilization, and cytochrome oxidase assembly	-2.05023	-1.03578	down
1778444_at	LCL1	YPL056C	Putative protein of unknown function; deletion mutant is fluconazole resistant and has long chronological lifespan	-2.0525	-1.03738	down
1771069_at		YLR063W		-2.05277	-1.03757	down
1778495_at	YPT7	YML001W	Rab family GTPase; GTP-binding protein of the rab family; required for homotypic fusion event in vacuole inheritance, for endosome-endosome fusion, similar to mammalian Rab7	-2.05341	-1.03802	down
1772231_at	PPX1	YHR201C	Exopolyphosphatase, hydrolyzes inorganic polyphosphate (poly P) into Pi residues; located in the cytosol, plasma membrane, and mitochondrial matrix	-2.05341	-1.03802	down
1774666_at	SEC66	YBR171W	Non-essential subunit of Sec63 complex (Sec63p, Sec62p, Sec66p and Sec72p); with Sec61 complex, Kar2p/BiP and Lhs1p forms a channel competent for SRP-dependent and post-translational SRP-independent protein targeting and import into the ER	-2.05439	-1.03871	down
1773420_at		YMR147W		-2.05665	-1.04029	down
1772826_at	LAS1	YKR063C	Protein required for pre-rRNA processing at both ends of ITS2; may coordinate the action of the Rat1p-Rai1p exoRNAse; required for the G1/S transition of the cell cycle; human ortholog is Las1L; mutants require the SSD1-v allele for viability	-2.05683	-1.04042	down
1775403_at	RPO21	YDL140C	RNA polymerase II largest subunit B220, part of central core; phosphorylation of C-terminal heptapeptide repeat domain regulates association with transcription and splicing factors; similar to bacterial beta-prime	-2.05708	-1.0406	down
1776825_at	CDS1	YBR029C	Phosphatidate cytidylyltransferase (CDP-diglyceride synthetase); an enzyme that catalyzes that conversion of CTP + phosphate into diphosphate + CDP-diaclglyerol, a critical step in the synthesis of all major yeast phospholipids	-2.0576	-1.04096	down
1770276_at	RPC34	YNR003C	RNA polymerase III subunit C34; interacts with TFIIIB70 and is a key determinant in pol III	-2.0579	-1.04117	down

			recruitment by the preinitiation complex			
1779075_at	OPT2	YPR194C	Oligopeptide transporter; member of the OPT family, with potential orthologs in S. pombe and C. albicans; also plays a role in formation of mature vacuoles	-2.05886	-1.04184	down
1771725_at		YLR243W		-2.05945	-1.04226	down
1779680_at		YMR310C		-2.05965	-1.0424	down
1772748_at	TMA7	YLR262C-A	Protein of unknown that associates with ribosomes; null mutant exhibits translation defects, altered polyribosome profiles, and resistance to the translation inhibitor anisomcyin	-2.05968	-1.04242	down
1771007_at	ECM32	YER176W	DNA dependent ATPase/DNA helicase belonging to the Dna2p- and Nam7p-like family of helicases that is involved in modulating translation termination; interacts with the translation termination factors, localized to polysomes	-2.06036	-1.0429	down
1774160_at	GAL83	YER027C	One of three possible beta-subunits of the Snf1 kinase complex, allows nuclear localization of the Snf1 kinase complex in the presence of a nonfermentable carbon source; contains glycogen-binding domain	-2.06071	-1.04314	down
1778624_at	NIF3	YGL221C	Protein of unknown function, similar to Listeria monocytogenes major sigma factor (rpoD gene product); the authentic, non-tagged protein is detected in highly purified mitochondria in high-throughput studies	-2.06085	-1.04324	down
1776407_at	ALK1	YGL021W	Protein kinase; accumulation and phosphorylation are periodic during the cell cycle; phosphorylated in response to DNA damage; contains characteristic motifs for degradation via the APC pathway; similar to Alk2p and to mammalian haspins	-2.06203	-1.04407	down
1779535_at	IMP4	YNL075W	Component of the SSU processome, which is required for pre-18S rRNA processing; interacts with Mpp10p; member of a superfamily of proteins that contain a sigma(70)-like motif and associate with RNAs	-2.06205	-1.04408	down
1779850_at	GPH1	YPR160W	Non-essential glycogen phosphorylase required for the mobilization of glycogen, activity is regulated by cyclic AMP-mediated phosphorylation, expression is regulated by stress-response elements and by the HOG MAP kinase pathway	-2.06433	-1.04567	down
1776077_at	TMA10 8	YIL137C	Protein that associates with ribosomes and is involved in ribosome biogenesis; putative metalloprotease	-2.06691	-1.04747	down
1772232_at		YLR179C		-2.06751	-1.0479	down
1772239_at	RLP7	YNL002C	Nucleolar protein with similarity to large ribosomal subunit L7 proteins; constituent of 66S pre-ribosomal particles; plays an essential role in processing of precursors to the large ribosomal subunit RNAs	-2.06848	-1.04857	down
1774134_at	ADE1	YAR015W	N-succinyl-5-aminoimidazole-4-carboxamide ribotide (SAICAR) synthetase, required for 'de novo' purine nucleotide biosynthesis; red pigment accumulates in mutant cells deprived of adenine	-2.0686	-1.04865	down
1770047_at	RRP3	YHR065C	Protein involved in rRNA processing; required for maturation of the 35S primary transcript of pre-rRNA and for cleavage leading to mature 18S rRNA; homologous to eIF-4a, which is a DEAD box RNA-dependent ATPase with helicase activity	-2.06904	-1.04896	down
1778277_at	SST2	YLR452C	GTPase-activating protein for Gpa1p, regulates desensitization to alpha factor pheromone; also required to prevent receptor-independent signaling of the mating pathway; member of the RGS (regulator of G-protein signaling) family	-2.07026	-1.04981	down

1775928_at	ADK1	YDR226W	Adenylate kinase, required for purine metabolism; localized to the cytoplasm and the mitochondria; lacks cleavable signal sequence	-2.07055	-1.05002	down
1771214_at	PYK2	YOR347C	Pyruvate kinase that appears to be modulated by phosphorylation; PYK2 transcription is repressed by glucose, and Pyk2p may be active under low glycolytic flux	-2.07232	-1.05125	down
1775056_at	CKB1	YGL019W	Beta regulatory subunit of casein kinase 2 (CK2), a Ser/Thr protein kinase with roles in cell growth and proliferation; CK2, comprised of CKA1, CKA2, CKB1 and CKB2, has many substrates including transcription factors and all RNA polymerases	-2.07403	-1.05244	down
1779018_at	URA8	YJR103W	Minor CTP synthase isozyme (see also URA7), catalyzes the ATP-dependent transfer of the amide nitrogen from glutamine to UTP, forming CTP, the final step in de novo biosynthesis of pyrimidines; involved in phospholipid biosynthesis	-2.07617	-1.05392	down
1779186_at	MRPL3	YMR024W	Mitochondrial ribosomal protein of the large subunit	-2.08301	-1.05867	down
1774903_at	DBP3	YGL078C	RNA-Dependent ATPase, member of DExD/H-box family; involved in cleavage of site A3 within the ITS1 spacer during rRNA processing; not essential for growth, but deletion causes severe slow-growth phenotype	-2.08477	-1.05989	down
1775701_at	AKR1	YDR264C	Palmitoyl transferase involved in protein palmitoylation; acts as a negative regulator of pheromone response pathway; required for endocytosis of pheromone receptors; involved in cell shape control; contains ankyrin repeats	-2.08566	-1.0605	down
1775282_at	PTI1	YGR156W	Essential protein that is a component of CPF (cleavage and polyadenylation factor); involved in 3' end formation of snoRNA and mRNA; interacts directly with Pta1p; has similarity to mammalian Cleavage-Stimulation Factor CstF-64	-2.08774	-1.06194	down
1774766_s_at		YBL112C	Putative protein of unknown function; YBL112C is contained within TEL02L	-2.09132	-1.06442	down
1778155_at		YJL118W		-2.0933	-1.06578	down
1770384_at		YDR415C		-2.09409	-1.06632	down
1779051_at	CDC11	YJR076C	Component of the septin ring that is required for cytokinesis; septins are GTP-binding proteins that assemble into rod-like hetero-oligomers that can associate with other rods to form filaments; septin rings at the mother-bud neck act as scaffolds for recruiting cell division factors and as barriers to prevent diffusion of specific proteins between mother and daughter cells	-2.09462	-1.06669	down
1776678_at	CAF20	YOR276W	Phosphoprotein of the mRNA cap-binding complex involved in translational control, repressor of cap-dependent translation initiation, competes with eIF4G for binding to eIF4E	-2.09537	-1.0672	down
1779840_at	SKI2	YLR398C	Ski complex component and putative RNA helicase, mediates 3'-5' RNA degradation by the cytoplasmic exosome; null mutants have superkiller phenotype of increased viral dsRNAs and are synthetic lethal with mutations in 5'-3' mRNA decay	-2.09587	-1.06755	down
1778928_s_at	RPS6B	YBR181C	Protein component of the small (40S) ribosomal subunit; homologous to mammalian ribosomal protein S6; phosphorylated on S233 by Ypk3p in a TORC1-dependent manner, and on S232 in a TORC1/2-dependent manner by Ypk1/2/3p	-2.09597	-1.06762	down
1774218_at	NUP133	YKR082W	Subunit of the Nup84p subcomplex of the nuclear pore complex (NPC); contributes to nucleocytoplasmic transport and NPC biogenesis and is involved in establishment of a normal	-2.09647	-1.06796	down

			nucleocytoplasmic concentration gradient of the GTPase Gsp1p; also plays roles in several processes that may require localization of genes or chromosomes at the nuclear periphery, including double-strand break repair, transcription and chromatin silencing; homologous to human NUP133			
1774513_at	MNN11	YJL183W	Subunit of a Golgi mannosyltransferase complex that also contains Anp1p, Mnn9p, Mnn10p, and Hoc1p, and mediates elongation of the polysaccharide mannan backbone; has homology to Mnn10p	-2.09935	-1.06994	down
1774776_at	PPT2	YPL148C	Phosphopantetheine:protein transferase (PPTase), activates mitochondrial acyl carrier protein (Acp1p) by phosphopantetheinylation	-2.10034	-1.07062	down
1773030_at	BUD9	YGR041W	Protein involved in bud-site selection; mutant has increased aneuploidy tolerance; diploid mutants display a unipolar budding pattern instead of the wild-type bipolar pattern, and bud at the distal pole	-2.10249	-1.0721	down
1776285_at	PUT4	YOR348C	Proline permease, required for high-affinity transport of proline; also transports the toxic proline analog azetidine-2-carboxylate (AzC); PUT4 transcription is repressed in ammonia-grown cells	-2.10608	-1.07456	down
1776092_at	GEA1	YJR031C	Guanine nucleotide exchange factor for ADP ribosylation factors (ARFs), involved in vesicular transport between the Golgi and ER, Golgi organization, and actin cytoskeleton organization; similar to but not functionally redundant with Gea2p	-2.10684	-1.07508	down
1778765_at	CEX1	YOR112W	Cytoplasmic component of the nuclear aminoacylation-dependent tRNA export pathway; interacts with nuclear pore component Nup116p; copurifies with tRNA export receptors Los1p and Msn5p, as well as eIF-1a and the RAN GTPase Gsp1p	-2.11209	-1.07867	down
1778014_at	FSH2	YMR222C	Putative serine hydrolase that localizes to the cytoplasm; sequence is similar to S. cerevisiae Fsh1p and Fsh3p and the human candidate tumor suppressor OVCA2	-2.11436	-1.08022	down
1778794_at	RPP0	YLR340W	Conserved ribosomal protein P0 of the ribosomal stalk, which is involved in interaction between translational elongation factors and the ribosome; similar to rat P0, human P0, and E. coli L10e; phosphorylated on serine 302	-2.11585	-1.08124	down
1778055_at	TAF14	YPL129W	Subunit of TFIID, TFIIF, INO80, SWI/SNF, and NuA3 complexes, involved in RNA polymerase II transcription initiation and in chromatin modification; contains a YEATS domain	-2.12879	-1.09004	down
1771212_at	UBC12	YLR306W	Enzyme that mediates the conjugation of Rub1p, a ubiquitin-like protein, to other proteins; related to E2 ubiquitin-conjugating enzymes	-2.12934	-1.0904	down
1772463_at	NUP170	YBL079W	Subunit of the inner ring of the nuclear pore complex (NPC); contributes to NPC assembly and nucleocytoplasmic transport; has similarity to Nup157p; both Nup170p and NUP157p are similar to human Nup155p	-2.1298	-1.09072	down
1779544_at	LCP5	YER127W	Essential protein involved in maturation of 18S rRNA; depletion leads to inhibited pre-rRNA processing and reduced polysome levels; localizes primarily to the nucleolus	-2.13086	-1.09143	down
1776010_at	RPS22A	YJL190C	Protein component of the small (40S) ribosomal subunit; nearly identical to Rps22Bp and has similarity to E. coli S8 and rat S15a ribosomal proteins	-2.13287	-1.0928	down
1772275_at	JJJ3	YJR097W	Protein of unknown function, contains a CSL Zn	-2.1358	-1.09477	down

			finger and a DnaJ-domain; involved in diphthamide biosynthesis; ortholog human Dph4			
1772923_at	TMA64	YDR117C	Protein of unknown function that associates with ribosomes; has a putative RNA binding domain; in mammals the corresponding protein, eIF2D, has been shown to possess translation initiation factor activity	-2.13818	-1.09638	down
1778504_at	VMA13	YPR036W	Subunit H of the eight-subunit V1 peripheral membrane domain of the vacuolar H+-ATPase (V-ATPase), an electrogenic proton pump found throughout the endomembrane system; serves as an activator or a structural stabilizer of the V-ATPase	-2.1391	-1.097	down
1779129_at	INO1	YJL153C	Inositol-3-phosphate synthase, involved in synthesis of inositol phosphates and inositol-containing phospholipids; transcription is coregulated with other phospholipid biosynthetic genes by Ino2p and Ino4p, which bind the UASINO DNA element	-2.14135	-1.09852	down
1770417_at	PEX6	YNL329C	AAA-peroxin that heterodimerizes with AAA-peroxin Pex1p and participates in the recycling of peroxisomal signal receptor Pex5p from the peroxisomal membrane to the cystosol	-2.14167	-1.09873	down
1779460_at	TPM1	YNL079C	Major isoform of tropomyosin; binds to and stabilizes actin cables and filaments, which direct polarized cell growth and the distribution of several organelles; acetylated by the NatB complex and acetylated form binds actin most efficiently	-2.14359	-1.10003	down
1772846_at	TOR2	YKL203C	PIK-related protein kinase and rapamycin target; subunit of TORC1, a complex that regulates growth in response to nutrients and TORC2, a complex that regulates cell-cycle dependent polarization of the actin cytoskeleton; involved in meiosis	-2.14517	-1.10109	down
1775971_at	GAA1	YLR088W	Subunit of the GPI (glycosylphosphatidylinositol):protein transamidase complex, removes the GPI-anchoring signal and attaches GPI to proteins in the ER	-2.14665	-1.10209	down
1777700_at	POM15 2	YMR129W	Glycoprotein subunit of the transmembrane ring of the nuclear pore complex (NPC); contributes to nucleocytoplasmic transport, NPC biogenesis and spindle pole body duplication; homologous to human NUP210	-2.14995	-1.10431	down
1778512_at	YSY6	YBR162W-A	Protein whose expression suppresses a secretory pathway mutation in E. coli; has similarity to the mammalian RAMP4 protein involved in secretion	-2.1531	-1.10642	down
1771650_at	REI1	YBR267W	Cytoplasmic pre-60S factor; required for the correct recycling of shuttling factors Alb1, Arx1 and Tif6 at the end of the ribosomal large subunit biogenesis; involved in bud growth in the mitotic signaling network	-2.15464	-1.10745	down
1778932_at	URB1	YKL014C	Nucleolar protein required for the normal accumulation of 25S and 5.8S rRNAs, associated with the 27SA2 pre-ribosomal particle; proposed to be involved in the biogenesis of the 60S ribosomal subunit	-2.15476	-1.10753	down
1776912_at		YPL162C		-2.15477	-1.10753	down
1776218_at	MFA1	YDR461W	Mating pheromone a-factor, made by a cells; interacts with alpha cells to induce cell cycle arrest and other responses leading to mating; biogenesis involves C-terminal modification, N-terminal proteolysis, and export; also encoded by MFA2	-2.15507	-1.10774	down
1779099_at	IOC2	YLR095C	Member of a complex (Isw1b) with Isw1p and Ioc4p that exhibits nucleosome-stimulated ATPase activity and acts within coding regions to coordinate transcription elongation with termination and processing, contains a PHD finger motif	-2.15633	-1.10858	down

1779329_at	GRH1	YDR517W	Acetylated, cis-golgi localized protein involved in ER to Golgi transport; homolog of human GRASP65; forms a complex with the coiled-coil protein Bug1p; mutants are compromised for the fusion of ER-derived vesicles with Golgi membranes	-2.15832	-1.10991	down
1774482_at	TRX1	YLR043C	Cytoplasmic thioredoxin isoenzyme of the thioredoxin system which protects cells against oxidative and reductive stress, forms LMA1 complex with Pbi2p, acts as a cofactor for Tsa1p, required for ER-Golgi transport and vacuole inheritance	-2.16252	-1.11271	down
1769850_at	HHO1	YPL127C	Histone H1, a linker histone required for nucleosome packaging at restricted sites; suppresses DNA repair involving homologous recombination; not required for telomeric silencing, basal transcriptional repression, or efficient sporulation	-2.16412	-1.11378	down
1772362_at	HOC1	YJR075W	Alpha-1,6-mannosyltransferase involved in cell wall mannan biosynthesis; subunit of a Golgi-localized complex that also contains Anp1p, Mnn9p, Mnn11p, and Mnn10p; identified as a suppressor of a cell lysis sensitive pkc1-371 allele	-2.16415	-1.1138	down
1775314_at	SFM1	YOR021C	SPOUT methyltransferase; catalyzes omega-monomethylation of Rps3p on Arg-146; not an essential gene; predicted to be involved in rRNA processing and ribosome biogenesis and in biopolymer catabolism	-2.16438	-1.11395	down
1775669_at	RIO2	YNL207W	Essential serine kinase involved in the processing of the 20S pre-rRNA into mature 18S rRNA; has similarity to Rio1p	-2.16468	-1.11415	down
1775031_at	SEC24	YIL109C	Component of the Sec23p-Sec24p heterodimer of the COPII vesicle coat, required for cargo selection during vesicle formation in ER to Golgi transport; homologous to Sfb2p and Sfb3p	-2.16632	-1.11525	down
1769558_at	MHP1	YJL042W	Microtubule-associated protein involved in assembly and stabilization of microtubules; overproduction results in cell cycle arrest at G2 phase; similar to Drosophila protein MAP and to mammalian MAP4 proteins	-2.16685	-1.1156	down
1778173_at	MET7	YOR241W	Folylpolyglutamate synthetase, catalyzes extension of the glutamate chains of the folate coenzymes, required for methionine synthesis and for maintenance of mitochondrial DNA	-2.17104	-1.11838	down
1777630_at	URA1	YKL216W	Dihydroorotate dehydrogenase, catalyzes the fourth enzymatic step in the de novo biosynthesis of pyrimidines, converting dihydroorotic acid into orotic acid	-2.17194	-1.11899	down
1775859_at	SAH1	YER043C	S-adenosyl-L-homocysteine hydrolase, catabolizes S-adenosyl-L-homocysteine which is formed after donation of the activated methyl group of S-adenosyl-L-methionine (AdoMet) to an acceptor	-2.17271	-1.1195	down
1770407_at	TRM2	YKR056W	tRNA methyltransferase, 5-methylates the uridine residue at position 54 of tRNAs and may also have a role in tRNA stabilization or maturation; endo-exonuclease with a role in DNA repair	-2.17312	-1.11977	down
1778403_at	NST1	YNL091W	Protein of unknown function, mediates sensitivity to salt stress; interacts physically with the splicing factor Msl1p and also displays genetic interaction with MSL1	-2.17754	-1.1227	down
1774222_at	AIM29	YKR074W	Putative protein of unknown function; epitope-tagged protein localizes to the cytoplasm; YKR074W is not an essential gene; null mutant displays elevated frequency of mitochondrial genome loss	-2.17884	-1.12356	down
1774643_at	ATS1	YAL020C	Protein required, with Elongator complex, Kti11p,	-2.17989	-1.12426	down

			and Kti12p, for modification of wobble nucleosides in tRNA; has a potential role in regulatory interactions between microtubules and the cell cycle			
1770106_at	SLX9	YGR081C	Protein required for pre-rRNA processing; associated with the 90S pre-ribosome and 43S small ribosomal subunit precursor; interacts with U3 snoRNA; deletion mutant has synthetic fitness defect with an sgs1 deletion mutant	-2.18128	-1.12517	down
1779633_at	PPH22	YDL188C	Catalytic subunit of protein phosphatase 2A (PP2A), functionally redundant with Pph21p; methylated at C terminus; forms alternate complexes with several regulatory subunits; involved in signal transduction and regulation of mitosis	-2.18373	-1.12679	down
1770155_at	RPS25A	YGR027C	Protein component of the small (40S) ribosomal subunit; nearly identical to Rps25Bp and has similarity to rat S25 ribosomal protein	-2.18686	-1.12886	down
1771497_at	RPS24B	YIL069C	Protein component of the small (40S) ribosomal subunit; identical to Rps24Ap and has similarity to rat S24 ribosomal protein	-2.18804	-1.12964	down
1779159_at	MPD1	YOR288C	Member of the protein disulfide isomerase (PDI) family; interacts with and inhibits the chaperone activity of Cne1p; MPD1 overexpression in a pdi1 null mutant suppresses defects in Pdi1p functions such as carboxypeptidase Y maturation	-2.18921	-1.13041	down
1772400_at	SEC14	YMR079W	Phosphatidylinositol/phosphatidylcholine transfer protein; involved in regulating PtdIns, PtdCho, and ceramide metabolism, products of which regulate intracellular transport and UPR; functionally homologous to mammalian PITPs	-2.19037	-1.13117	down
1773022_at	CDC73	YLR418C	Component of the Paf1p complex; binds to and modulates the activity of RNA polymerases I and II; required for expression of certain genes, modification of some histones, and telomere maintenance; involved in transcription elongation as demonstrated by the G-less-based run-on (GLRO) assay	-2.19213	-1.13234	down
1776846_at	SEC72	YLR292C	Non-essential subunit of Sec63 complex (Sec63p, Sec62p, Sec66p and Sec72p); with Sec61 complex, Kar2p/BiP and Lhs1p forms a channel competent for SRP-dependent and post-translational SRP-independent protein targeting and import into the ER	-2.1922	-1.13238	down
1772475_at	PMT5	YDL093W	Protein O-mannosyltransferase, transfers mannose residues from dolichyl phosphate-D-mannose to protein serine/threonine residues; acts in a complex with Pmt3p, can instead interact with Pmt2p in some conditions; target for new antifungals	-2.19737	-1.13578	down
1777320_at	HFI1	YPL254W	Adaptor protein required for structural integrity of the SAGA complex, a histone acetyltransferase-coactivator complex that is involved in global regulation of gene expression through acetylation and transcription functions	-2.19878	-1.1367	down
1772654_at	ALG3	YBL082C	Dolichol-P-Man dependent alpha(1-3) mannosyltransferase, involved in the synthesis of dolichol-linked oligosaccharide donor for N-linked glycosylation of proteins	-2.19917	-1.13696	down
1773646_at	LRG1	YDL240W	Putative GTPase-activating protein (GAP) involved in the Pkc1p-mediated signaling pathway that controls cell wall integrity; appears to specifically regulate 1,3-beta-glucan synthesis	-2.19989	-1.13743	down
1774290_at	PYC2	YBR218C	Pyruvate carboxylase isoform, cytoplasmic enzyme that converts pyruvate to oxaloacetate; highly similar to isoform Pyc1p but differentially regulated; mutations in the human homolog are	-2.20123	-1.13831	down

			associated with lactic acidosis			
1769809_at		YDL012C		-2.20204	-1.13884	down
1769637_at	POP8	YBL018C	Subunit of both RNase MRP and nuclear RNase P; RNase MRP cleaves pre-rRNA, while nuclear RNase P cleaves tRNA precursors to generate mature 5' ends and facilitates turnover of nuclear RNAs	-2.20327	-1.13965	down
1773809_at	AQR1	YNL065W	Plasma membrane multidrug transporter of the major facilitator superfamily, confers resistance to short-chain monocarboxylic acids and quinidine; involved in the excretion of excess amino acids	-2.20408	-1.14018	down
1778673_at	DED81	YHR019C	Cytosolic asparaginyl-tRNA synthetase, required for protein synthesis, catalyzes the specific attachment of asparagine to its cognate tRNA	-2.20697	-1.14207	down
1774880_at	SFT1	YKL006C-A	Intra-Golgi v-SNARE, required for transport of proteins between an early and a later Golgi compartment	-2.20849	-1.14306	down
1774150_at	VAC14	YLR386W	Protein involved in regulated synthesis of PtdIns(3,5)P(2), in control of trafficking of some proteins to the vacuole lumen via the MVB, and in maintenance of vacuole size and acidity; interacts with Fig4p; activator of Fab1p	-2.21346	-1.1463	down
1778104_at	ERG24	YNL280C	C-14 sterol reductase, acts in ergosterol biosynthesis; mutants accumulate the abnormal sterol ignosterol (ergosta-8,14 dienol), and are viable under anaerobic growth conditions but inviable on rich medium under aerobic conditions	-2.21346	-1.1463	down
1780041_at	EGD1	YPL037C	Subunit beta1 of the nascent polypeptide-associated complex (NAC) involved in protein targeting, associated with cytoplasmic ribosomes; enhances DNA binding of the Gal4p activator; homolog of human BTF3b	-2.21467	-1.14709	down
1769374_at	MOB2	YFL034C-B	Activator of Cbk1p kinase; component of the RAM signaling network that regulates cellular polarity and morphogenesis; activation of Cbk1p facilitates the Ace2p-dependent daughter cell-specific transcription of genes involved in cell separation; similar to Mob1p	-2.21482	-1.14719	down
1774844_at	PRO2	YOR323C	Gamma-glutamyl phosphate reductase, catalyzes the second step in proline biosynthesis	-2.21561	-1.1477	down
1778791_at	DCD1	YHR144C	Deoxycytidine monophosphate (dCMP) deaminase; involved in dUMP and dTMP biosynthesis; expression is NOT cell cycle regulated	-2.21889	-1.14984	down
1774263_at	EGT2	YNL327W	Glycosylphosphatidylinositol (GPI)-anchored cell wall endoglucanase required for proper cell separation after cytokinesis, expression is activated by Swi5p and tightly regulated in a cell cycle-dependent manner	-2.21907	-1.14995	down
1776003_at	SEC63	YOR254C	Essential subunit of Sec63 complex (Sec63p, Sec62p, Sec66p and Sec72p); with Sec61 complex, Kar2p/BiP and Lhs1p forms a channel competent for SRP-dependent and post-translational SRP-independent protein targeting and import into the ER	-2.22442	-1.15343	down
1773413_at	NUM1	YDR150W	Protein required for nuclear migration, localizes to the mother cell cortex and the bud tip; may mediate interactions of dynein and cytoplasmic microtubules with the cell cortex	-2.22446	-1.15346	down
1778943_at	RIM2	YBR192W	Mitochondrial pyrimidine nucleotide transporter; imports pyrimidine nucleoside triphosphates and exports pyrimidine nucleoside monophosphates; member of the mitochondrial carrier family	-2.22707	-1.15515	down
1779599_at	RPL31A	YDL075W	Protein component of the large (60S) ribosomal subunit, nearly identical to Rpl31Bp and has similarity to rat L31 ribosomal protein; associates	-2.22728	-1.15528	down

			with the karyopherin Sxm1p; loss of both Rpl31p and Rpl39p confers lethality			
1776610_at	URA5	YML106W	Major orotate phosphoribosyltransferase (OPRTase) isozyme that catalyzes the fifth enzymatic step in de novo biosynthesis of pyrimidines, converting orotate into orotidine-5'-phosphate; minor OPRTase encoded by URA10	-2.22848	-1.15606	down
1774765_at	SEH1	YGL100W	Component of two distinct complexes; subunit of the Nup84 nuclear pore sub-complex (NPC) and the Seh1-associated (SEA) complex; the the NUP84 subcomplex contributes to nucleocytoplasmic transport and NPC biogenesis; the SEA complex is a coatamer-related complex that associates dynamically with the vacuole; homologous to human SEH1	-2.22855	-1.1561	down
1774780_at	SLC1	YDL052C	1-acyl-sn-glycerol-3-phosphate acyltransferase, catalyzes the acylation of lysophosphatidic acid to form phosphatidic acid, a key intermediate in lipid metabolism; enzymatic activity detected in lipid particles and microsomes	-2.2291	-1.15646	down
1772675_at	ISW1	YBR245C	ATPase subunit of imitation-switch (ISWI) class chromatin remodelers; ATPase; forms a complex with Ioc3p called Isw1a and a complex with Ioc2p and Ioc4p called Isw1b; Isw1a and Isw1b have both partially overlapping and distinct roles with Isw1a involved in repression of transcription initiation and Isw1b involved in regulation of transcription elongation	-2.23064	-1.15746	down
1779854_at	NUP120	YKL057C	Subunit of the Nup84p subcomplex of the nuclear pore complex (NPC); contributes to nucleocytoplasmic transport and NPC biogenesis and is involved in establishment of a normal nucleocytoplasmic concentration gradient of the GTPase Gsp1p; also plays roles in several processes that may require localization of genes or chromosomes at the nuclear periphery, including double-strand break repair, transcription and chromatin silencing; homologous to human NUP160	-2.23388	-1.15955	down
1769756_at	KAP104	YBR017C	Transportin or cytosolic karyopherin beta 2; functions in the rg-nuclear localization signal-mediated nuclear import/reimport of mRNA-binding proteins Nab2p and Hrp1p; regulates asymmetric protein synthesis in daughter cells during mitosis	-2.23452	-1.15996	down
1773002_at	TAF9	YMR236W	Subunit (17 kDa) of TFIID and SAGA complexes, involved in RNA polymerase II transcription initiation and in chromatin modification, similar to histone H3	-2.23467	-1.16006	down
1774604_at	THS1	YIL078W	Threonyl-tRNA synthetase, essential cytoplasmic protein	-2.23511	-1.16035	down
1770063_at	DIS3	YOL021C	Exosome core complex catalytic subunit; possesses both endonuclease and 3'-5' exonuclease activity; involved in 3'-5' RNA processing and degradation in both the nucleus and the cytoplasm; has similarity to E. coli RNase R and to human DIS3	-2.23559	-1.16066	down
1779387_at		YML079W		-2.2362	-1.16105	down
1771652_at		YML096W		-2.23682	-1.16145	down
1775448_at	RPL37B	YDR500C	Protein component of the large (60S) ribosomal subunit, has similarity to Rpl37Ap and to rat L37 ribosomal protein	-2.23709	-1.16162	down
1771495_at	HLR1	YDR528W	Protein involved in regulation of cell wall composition and integrity and response to osmotic stress; overproduction suppresses a lysis sensitive PKC mutation; similar to Lre1p, which functions	-2.24563	-1.16712	down

			antagonistically to protein kinase A			
1778452_at	MRP4	YHL004W	Mitochondrial ribosomal protein of the small subunit	-2.24775	-1.16848	down
1771664_at		YMR315W		-2.25178	-1.17107	down
1769568_at	NMA2	YGR010W	Nicotinic acid mononucleotide adenylyltransferase, involved in de novo and salvage synthesis of NAD(+)	-2.25392	-1.17244	down
1772510_at	VMA1	YDL185W	Subunit A of the eight-subunit V1 peripheral membrane domain of the vacuolar H+-ATPase; protein precursor undergoes self-catalyzed splicing to yield the extein Tfp1p and the intein Vde (PI-SceI), which is a site-specific endonuclease	-2.25412	-1.17256	down
1772611_at	FLD1	YLR404W	Seipin protein involved in lipid droplet morphology, number, and size; proposed to be involved in lipid metabolism; related to the human BSCL2 which is associated with lipodystrophy	-2.25484	-1.17302	down
1775807_at	GCD1	YOR260W	Gamma subunit of the translation initiation factor eIF2B, the guanine-nucleotide exchange factor for eIF2; activity subsequently regulated by phosphorylated eIF2; first identified as a negative regulator of GCN4 expression	-2.25485	-1.17303	down
1773973_at	BUD8	YLR353W	Protein involved in bud-site selection; diploid mutants display a unipolar budding pattern instead of the wild-type bipolar pattern, and bud at the proximal pole	-2.25998	-1.17631	down
1776697_at	TRM7	YBR061C	2'-O-ribose methyltransferase, methylates the 2'-O-ribose of nucleotides at positions 32 and 34 of the tRNA anticodon loop	-2.26127	-1.17713	down
1771920_at	ASC1	YMR116C	G-protein beta subunit and guanine nucleotide dissociation inhibitor for Gpa2p; ortholog of RACK1 that inhibits translation; core component of the small (40S) ribosomal subunit; represses Gcn4p in the absence of amino acid starvation	-2.26768	-1.18122	down
1773088_at	RRP5	YMR229C	RNA binding protein with preference for single stranded tracts of U's involved in synthesis of both 18S and 5.8S rRNAs; component of both the ribosomal small subunit (SSU) processosome and the 90S preribosome	-2.27048	-1.183	down
1778322_at	SRM1	YGL097W	Nucleotide exchange factor for Gsp1p, localizes to the nucleus, required for nucleocytoplasmic trafficking of macromolecules; suppressor of the pheromone response pathway; potentially phosphorylated by Cdc28p	-2.27169	-1.18376	down
1774576_at	PSY2	YNL201C	Subunit of protein phosphatase PP4 complex; active complex is composed of catalytic subunit Pph3p and Psy2p, with Psy4p apparently providing additional substrate specificity in some cases; regulates recovery from the DNA damage checkpoint and also the gene conversion- and single-strand annealing-mediated pathways of meiotic double-strand break repair; Pph3p and Psy2p localize to foci on meiotic chromosomes; putative homolog of mammalian R3	-2.27279	-1.18446	down
1773984_at	SLH1	YGR271W	Putative RNA helicase related to Ski2p, involved in translation inhibition of non-poly(A) mRNAs; required for repressing propagation of dsRNA viruses	-2.27336	-1.18482	down
1776614_at	CLN3	YAL040C	G1 cyclin involved in cell cycle progression; activates Cdc28p kinase to promote the G1 to S phase transition; plays a role in regulating transcription of the other G1 cyclins, CLN1 and CLN2; regulated by phosphorylation and proteolysis	-2.27435	-1.18545	down
1775775_at	DPH1	YIL103W	Protein required, along with Dph2p, Kti11p, Jjj3p, and Dph5p, for synthesis of diphthamide, which is	-2.27456	-1.18559	down

			a modified histidine residue of translation elongation factor 2 (Eft1p or Eft2p); may act in a complex with Dph2p and Kti11p			
1777524_at	VMA6	YLR447C	Subunit d of the five-subunit V0 integral membrane domain of vacuolar H+-ATPase (V-ATPase), an electrogenic proton pump found in the endomembrane system; stabilizes VO subunits; required for V1 domain assembly on the vacuolar membrane	-2.27633	-1.18671	down
1778919_at	RNR1	YER070W	Major isoform of the large subunit of ribonucleotide-diphosphate reductase; the RNR complex catalyzes rate-limiting step in dNTP synthesis, regulated by DNA replication and DNA damage checkpoint pathways via localization of small subunits	-2.27683	-1.18703	down
1772719_at	AFG2	YLR397C	ATPase of the CDC48/PAS1/SEC18 (AAA) family, forms a hexameric complex; is essential for pre-60S maturation and release of several preribosome maturation factors; may be involved in degradation of aberrant mRNAs	-2.27854	-1.18811	down
1771841_at	APS3	YJL024C	Small subunit of the clathrin-associated adaptor complex AP-3, which is involved in vacuolar protein sorting; related to the sigma subunit of the mammalian clathrin AP-3 complex; suppressor of loss of casein kinase 1 function	-2.28037	-1.18927	down
1772372_at	PUB1	YNL016W	Poly (A)+ RNA-binding protein, abundant mRNP-component protein that binds mRNA and is required for stability of many mRNAs; component of glucose deprivation induced stress granules, involved in P-body-dependent granule assembly	-2.2854	-1.19245	down
1769576_at	AIM34	YMR003W	Protein of unknown function; GFP-fusion protein localizes to the mitochondria; null mutant is viable and displays reduced frequency of mitochondrial genome loss	-2.28679	-1.19333	down
1778906_at	SPE2	YOL052C	S-adenosylmethionine decarboxylase, required for the biosynthesis of spermidine and spermine; cells lacking Spe2p require spermine or spermidine for growth in the presence of oxygen but not when grown anaerobically	-2.28843	-1.19436	down
1778423_at	TRM11 2	YNR046W	Protein involved in methylation of tRNA, rRNA, and translation factors; subunit of tRNA methyltransferase (MTase) complexes in combination with Trm9p and Trm11p; interacts with and stabilizes 18S rRNA methyltransferase Bud23p; subunit of complex with Mtq2p that methylates Sup45p (eRF1) in the ternary complex eRF1-eRF3-GTP; deletion confers resistance to zymocin	-2.29028	-1.19552	down
1779917_at	CGI121	YML036W	Component of the EKC/KEOPS complex with Bud32p, Kae1p, Pcc1p, and Gon7p; EKC/KEOPS complex is required for t6A tRNA modification and may have roles in telomere maintenance and transcription; Cgi121p is dispensable for tRNA modification	-2.29908	-1.20106	down
1777724_at		YLR278C		-2.30405	-1.20417	down
1776921_at	RAD3	YER171W	5' to 3' DNA helicase, involved in nucleotide excision repair and transcription; subunit of RNA polII initiation factor TFIIH and of Nucleotide Excision Repair Factor 3 (NEF3); homolog of human XPD protein; mutant has aneuploidy tolerance	-2.307	-1.20602	down
1772116_at	CRN1	YLR429W	Coronin, cortical actin cytoskeletal component that associates with the Arp2p/Arp3p complex to regulate its activity; plays a role in regulation of actin patch assembly	-2.3073	-1.20621	down

1769317_at	DAT1	YML113W	DNA binding protein that recognizes oligo(dA).oligo(dT) tracts; Arg side chain in its N-terminal pentad Gly-Arg-Lys-Pro-Gly repeat is required for DNA-binding; not essential for viability	-2.30873	-1.2071	down
1770979_at	FLX1	YIL134W	Protein required for transport of flavin adenine dinucleotide (FAD), a synthesis product of riboflavin, across the mitochondrial membrane	-2.31138	-1.20875	down
1770967_at		YIL096C		-2.31182	-1.20903	down
1779154_s_at	RPS4B	YHR203C	Protein component of the small (40S) ribosomal subunit; homologous to mammalian ribosomal protein S4	-2.31333	-1.20997	down
1779552_at	TUM1	YOR251C	Rhodanese domain sulfur transferase, accepts persulfite from Nfs1p and transfers it to Uba4p in the pathway for 2-thiolation of the wobble uridine base of tRNAs; also stimulates sulfur transfer by Nfs1p; may be mitochondrially localized	-2.31445	-1.21067	down
1776193_at	TAT1	YBR069C	Amino acid transport protein for valine, leucine, isoleucine, and tyrosine, low-affinity tryptophan and histidine transporter; overexpression confers FK506 and FTY720 resistance	-2.31487	-1.21093	down
1777012_at	ARP4	YJL081C	Nuclear actin-related protein involved in chromatin remodeling, component of chromatin-remodeling enzyme complexes	-2.31545	-1.21129	down
1773258_at	MAK21	YDR060W	Constituent of 66S pre-ribosomal particles, required for large (60S) ribosomal subunit biogenesis; involved in nuclear export of pre-ribosomes; required for maintenance of dsRNA virus; homolog of human CAATT-binding protein	-2.31606	-1.21167	down
1772524_at		YML108W		-2.31727	-1.21243	down
1775829_at	NDC1	YML031W	Subunit of the transmembrane ring of the nuclear pore complex (NPC); contributes to nucleocytoplasmic transport, NPC biogenesis and spindle pole body duplication; homologous to human NDC1	-2.31802	-1.21289	down
1770062_at	PBP1	YGR178C	Component of glucose deprivation induced stress granules, involved in P-body-dependent granule assembly; similar to human ataxin-2; interacts with Pab1p to regulate mRNA polyadenylation; interacts with Mkt1p to regulate HO translation	-2.31892	-1.21345	down
1771693_at	SHU1	YHL006C	Component of the Shu complex, which promotes error-free DNA repair; Shu complex mediates inhibition of Srs2p function	-2.31996	-1.2141	down
1777963_at	MKT1	YNL085W	Protein that forms a complex with Pbp1p that may mediate posttranscriptional regulation of HO; involved in propagation of M2 dsRNA satellite of L-A virus; allelic variation affects mitochondrial genome stability, drug resistance, and more	-2.32102	-1.21476	down
1769895_at	MET18	YIL128W	DNA repair and TFIIH regulator, required for both nucleotide excision repair (NER) and RNA polymerase II (RNAP II) transcription; involved in telomere maintenance	-2.32185	-1.21527	down
1777863_at	ARP7	YPR034W	Component of both the SWI/SNF and RSC chromatin remodeling complexes; actin-related protein involved in transcriptional regulation	-2.32423	-1.21675	down
1770766_at	EST3	YIL009C-A	Component of the telomerase holoenzyme, involved in telomere replication	-2.32488	-1.21716	down
1777020_at	CIC1	YHR052W	Essential protein that interacts with proteasome components and has a potential role in proteasome substrate specificity; also copurifies with 66S pre-ribosomal particles	-2.32974	-1.22017	down
1770662_at	TMA22	YJR014W	Protein of unknown function; associates with ribosomes and has a putative RNA binding domain; interacts with Tma20p; similar to human GRAP and human DRP1, which interacts with human	-2.3309	-1.22089	down

			Tma20p homolog MCT-1			
1773173_at	RET2	YFR051C	Delta subunit of the coatomer complex (COPI), which coats Golgi-derived transport vesicles; involved in retrograde transport between Golgi and ER	-2.33109	-1.22101	down
1771862_at	NUP145	YGL092W	Essential protein with distinct roles in two nuclear pore subcomplexes; catalyzes its own proteolytic cleavage in vivo to generate a C-terminal fragment that is a structural component of the Nup84p subcomplex (with roles in NPC biogenesis and localization of genes to the nuclear periphery), and an N-terminal fragment that is one of several FG-nucleoporins within the NPC central core directly responsible for nucleocytoplasmic transport; homologous to human NUP98	-2.33253	-1.22189	down
1776344_at	RPL14 A	YKL006W	N-terminally acetylated protein component of the large (60S) ribosomal subunit, nearly identical to Rpl14Bp and has similarity to rat L14 ribosomal protein; rpl14a csh5 double null mutant exhibits synthetic slow growth	-2.33427	-1.22297	down
1771577_at	MNN2	YBR015C	Alpha-1,2-mannosyltransferase, responsible for addition of the first alpha-1,2-linked mannose to form the branches on the mannan backbone of oligosaccharides, localizes to an early Golgi compartment	-2.33446	-1.22309	down
1776621_at	RPC25	YKL144C	RNA polymerase III subunit C25, required for transcription initiation; forms a heterodimer with Rpc17p; paralog of Rpb7p	-2.33539	-1.22366	down
1771109_at	WSC4	YHL028W	ER membrane protein involved in the translocation of soluble secretory proteins and insertion of membrane proteins into the ER membrane; may also have a role in the stress response but has only partial functional overlap with WSC1-3	-2.33661	-1.22442	down
1779425_at	CYC3	YAL039C	Cytochrome c heme lyase (holocytochrome c synthase), attaches heme to apo-cytochrome c (Cyc1p or Cyc7p) in the mitochondrial intermembrane space; human ortholog may have a role in microphthalmia with linear skin defects (MLS)	-2.33787	-1.22519	down
1775078_at	SPB4	YFL002C	Putative ATP-dependent RNA helicase, nucleolar protein required for synthesis of 60S ribosomal subunits at a late step in the pathway; sediments with 66S pre-ribosomes in sucrose gradients	-2.33832	-1.22547	down
1777214_at		YNL162W-A		-2.33989	-1.22644	down
1769635_at	ADD37	YMR184W	Protein of unknown function involved in ER-associated protein degradation; green fluorescent protein (GFP)-fusion protein localizes to the cytoplasm and is induced in response to the DNA-damaging agent MMS; YMR184W is not an essential gene	-2.33989	-1.22644	down
1777041_at	FIR1	YER032W	Protein involved in 3' mRNA processing, interacts with Ref2p; potential Cdc28p substrate	-2.34045	-1.22679	down
1777293_at	KTI11	YBL071W-A	Zn-ribbon protein that co-purifies with Dph1 and Dph2 in a complex required for synthesis of diphthamide on translation factor eEF2 and with Elongator subunits Iki3p, Elp2p, and Elp3p involved in modification of wobble nucleosides in tRNAs	-2.34314	-1.22845	down
1776143_at	STH1	YIL126W	ATPase component of the RSC chromatin remodeling complex; required for expression of early meiotic genes; essential helicase-related protein homologous to Snf2p	-2.34328	-1.22853	down
1774224_at	GTB1	YDR221W	Glucosidase II beta subunit, forms a complex with alpha subunit Rot2p, involved in removal of two glucose residues from N-linked glycans during	-2.34647	-1.23049	down

			glycoprotein biogenesis in the ER			
1771813_at	LAP2	YNL045W	Leucyl aminopeptidase yscIV (leukotriene A4 hydrolase) with epoxide hydrolase activity, metalloenzyme containing one zinc atom; green fluorescent protein (GFP)-fusion protein localizes to the cytoplasm and nucleus	-2.35032	-1.23285	down
1774275_at	RPS17A	YML024W	Ribosomal protein 51 (rp51) of the small (40s) subunit; nearly identical to Rps17Bp and has similarity to rat S17 ribosomal protein	-2.35115	-1.23337	down
1778096_at	SPC97	YHR172W	Component of the microtubule-nucleating Tub4p (gamma-tubulin) complex; interacts with Spc110p at the spindle pole body (SPB) inner plaque and with Spc72p at the SPB outer plaque	-2.35185	-1.2338	down
1775633_at	DER1	YBR201W	Endoplasmic reticulum membrane protein, required for ER-associated protein degradation of misfolded or unassembled proteins; N- and C- termini protrude into the cytoplasm, has similarity to Dfm1p	-2.35294	-1.23447	down
1773348_at		YBR137W		-2.35385	-1.23502	down
1773619_at	TOM20	YGR082W	Component of the TOM (translocase of outer membrane) complex responsible for recognition and initial import steps for all mitochondrially directed proteins; acts as a receptor for incoming precursor proteins	-2.35834	-1.23777	down
1776088_at	NRM1	YNR009W	Transcriptional co-repressor of MBF (MCB binding factor)-regulated gene expression; Nrm1p associates stably with promoters via MBF to repress transcription upon exit from G1 phase	-2.35896	-1.23815	down
1773712_at	NUP53	YMR153W	FG-nucleoporin component of central core of the nuclear pore complex (NPC); also part of the NPC nuclear basket; contributes directly to nucleocytoplasmic transport; involved in regulation of transcription and mitosis	-2.36134	-1.2396	down
1770410_at		YGR210C		-2.36343	-1.24088	down
1772167_at		YKL018C-A		-2.36591	-1.24239	down
1770067_at	HIR3	YJR140C	Subunit of the HIR complex; a nucleosome assembly complex involved in regulation of histone gene transcription; involved in position-dependent gene silencing and nucleosome reassembly; ortholog of human CABIN1 protein	-2.36671	-1.24288	down
1773620_at	TYW3	YGL050W	tRNA methyltransferase required for synthesis of wybutosine, a modified guanosine found at the 3'-position adjacent to the anticodon of phenylalanine tRNA which supports reading frame maintenance by stabilizing codon-anticodon interactions	-2.36739	-1.2433	down
1773060_at	SPO73	YER046W	Meiosis-specific protein of unknown function, required for spore wall formation during sporulation; dispensible for both nuclear divisions during meiosis	-2.36774	-1.24351	down
1771126_at	PUF6	YDR496C	Pumilio-homology domain protein that binds the 3' UTR of ASH1 mRNA and represses its translation, resulting in proper asymmetric localization of ASH1 mRNA; also co-sediments with the 60S ribosomal subunit and is required for its biogenesis	-2.36878	-1.24414	down
1777955_at	RPS23A	YGR118W	Ribosomal protein 28 (rp28) of the small (40S) ribosomal subunit, required for translational accuracy; nearly identical to Rps23Bp and similar to E. coli S12 and rat S23 ribosomal proteins; deletion of both RPS23A and RPS23B is lethal	-2.369	-1.24428	down
1773332_at	SCO1	YBR037C	Copper-binding protein of the mitochondrial inner membrane, required for cytochrome c oxidase activity and respiration; may function to deliver copper to cytochrome c oxidase; has similarity to thioredoxins	-2.36929	-1.24446	down

1773520_at		YDR514C		-2.37077	-1.24536	down
1774225_at	MOS1	YCL057C-A	Mitochondrial protein essential for proper inner membrane organization; conserved component of the mitochondrial inner membrane organizing system (MICOS, MINOS, or MitOS), a scaffold-like structure on the intermembrane space side of the inner membrane which has a role in the maintenance of crista junctions and inner membrane architecture; ortholog of human MINOS1	-2.37285	-1.24662	down
1776626_at	SCW11	YGL028C	Cell wall protein with similarity to glucanases; may play a role in conjugation during mating based on its regulation by Ste12p	-2.37293	-1.24667	down
1778439_at	RPL23B	YER117W	Protein component of the large (60S) ribosomal subunit, identical to Rpl23Ap and has similarity to E. coli L14 and rat L23 ribosomal proteins	-2.37578	-1.2484	down
1777409_at		YCR015C		-2.37608	-1.24858	down
1777134_at	RPL28	YGL103W	Ribosomal protein of the large (60S) ribosomal subunit, has similarity to E. coli L15 and rat L27a ribosomal proteins; may have peptidyl transferase activity; can mutate to cycloheximide resistance	-2.37849	-1.25005	down
1771187_at	VPS62	YGR141W	Vacuolar protein sorting (VPS) protein required for cytoplasm to vacuole targeting of proteins	-2.38194	-1.25214	down
1772796_at	SEN54	YPL083C	Subunit of the tRNA splicing endonuclease, which is composed of Sen2p, Sen15p, Sen34p, and Sen54p	-2.38647	-1.25488	down
1775468_at	NHP2	YDL208W	Nuclear protein related to mammalian high mobility group (HMG) proteins, essential for function of H/ACA-type snoRNPs, which are involved in 18S rRNA processing	-2.38681	-1.25509	down
1770109_at	RPL14B	YHL001W	Protein component of the large (60S) ribosomal subunit, nearly identical to Rpl14Ap and has similarity to rat L14 ribosomal protein	-2.38868	-1.25621	down
1775969_at	UBX2	YML013W	Bridging factor involved in ER-associated protein degradation (ERAD); bridges the cytosolic Cdc48p-Npl1p-Ufd1p ATPase complex and the membrane associated Ssm4p and Hrd1p ubiquitin ligase complexes; contains a UBX (ubiquitin regulatory X) domain and a ubiquitin-associated (UBA) domain; redistributes from the ER to lipid droplets during the diauxic shift and stationary phase; required for the maintenance of lipid homeostasis	-2.39051	-1.25732	down
1777795_at	PTA1	YAL043C	Subunit of holo-CPF, a multiprotein complex and functional homolog of mammalian CPSF, required for the cleavage and polyadenylation of mRNA and snoRNA 3' ends; involved in pre-tRNA processing; binds to the phosphorylated CTD of RNAPII	-2.39109	-1.25767	down
1778491_at	TRZ1	YKR079C	tRNA 3'-end processing endonuclease tRNase Z; also localized to mitochondria and interacts genetically with Rex2 exonuclease; homolog of the human candidate prostate cancer susceptibility gene ELAC2	-2.39347	-1.2591	down
1774035_at	NOC3	YLR002C	Protein that forms a nuclear complex with Noc2p that binds to 66S ribosomal precursors to mediate their intranuclear transport; also binds to chromatin to promote the association of DNA replication factors and replication initiation	-2.39525	-1.26018	down
1776429_at	UAF30	YOR295W	Subunit of UAF (upstream activation factor), which is an RNA polymerase I specific transcription stimulatory factor composed of Uaf30p, Rrn5p, Rrn9p, Rrn10p, histones H3 and H4; deletion decreases cellular growth rate	-2.39549	-1.26032	down
1775593_at	CDC5	YMR001C	Polo-like kinase with multiple functions in mitosis and cytokinesis through substrate phosphorylation,	-2.39652	-1.26094	down

			also functions in adaptation to DNA damage during meiosis; has similarity to Xenopus Plx1 and S. pombe Plo1p; possible Cdc28p substrate			
1775076_at	RNH201	YNL072W	Ribonuclease H2 catalytic subunit, removes RNA primers during Okazaki fragment synthesis and errant ribonucleotides misincorporated during DNA replication; homolog of RNAse HI; related to human AGS4 which causes Aicardi-Goutieres syndrome	-2.39678	-1.2611	down
1774334_at	RPS28A	YOR167C	Protein component of the small (40S) ribosomal subunit; nearly identical to Rps28Bp and has similarity to rat S28 ribosomal protein	-2.3978	-1.26171	down
1771321_at	RSC6	YCR052W	Component of the RSC chromatin remodeling complex; essential for mitotic growth; homolog of SWI/SNF subunit Swp73p	-2.39986	-1.26295	down
1769519_at	CWP1	YKL096W	Cell wall mannoprotein that localizes specifically to birth scars of daughter cells, linked to a beta-1,3- and beta-1,6-glucan heteropolymer through a phosphodiester bond; required for propionic acid resistance	-2.4008	-1.26351	down
1774551_at	TOM71	YHR117W	Mitochondrial outer membrane protein with similarity to Tom70p; probable minor component of the TOM (translocase of outer membrane) complex responsible for recognition and import of mitochondrially directed proteins	-2.40397	-1.26542	down
1775374_at		YMR099C		-2.4044	-1.26568	down
1771766_at	MAP1	YLR244C	Methionine aminopeptidase, catalyzes the cotranslational removal of N-terminal methionine from nascent polypeptides; function is partially redundant with that of Map2p	-2.41279	-1.2707	down
1779910_at	ZRT2	YLR130C	Low-affinity zinc transporter of the plasma membrane; transcription is induced under low-zinc conditions by the Zap1p transcription factor	-2.41331	-1.27101	down
1780161_at	RPL16A	YIL133C	N-terminally acetylated protein component of the large (60S) ribosomal subunit, binds to 5.8 S rRNA; has similarity to Rpl16Bp, E. coli L13 and rat L13a ribosomal proteins; transcriptionally regulated by Rap1p	-2.4149	-1.27197	down
1773696_at	PAM18	YLR008C	Constituent of the import motor (PAM complex) component of the Translocase of the Inner Mitochondrial membrane (TIM23 complex); essential J-protein cochaperone that stimulates Ssc1p ATPase activity to drive import; inhibited by Pam16p	-2.41843	-1.27407	down
1771778_at	PMT4	YJR143C	Protein O-mannosyltransferase, transfers mannose residues from dolichyl phosphate-D-mannose to protein serine/threonine residues; appears to form homodimers in vivo and does not complex with other Pmt proteins; target for new antifungals	-2.41967	-1.27481	down
1772442_at	CBF5	YLR175W	Pseudouridine synthase catalytic subunit of box H/ACA small nucleolar ribonucleoprotein particles (snoRNPs), acts on both large and small rRNAs and on snRNA U2; mutations in human ortholog dyskerin cause the disorder dyskeratosis congenita	-2.41982	-1.2749	down
1774470_at	ENV10	YLR065C	Protein proposed to be involved in vacuolar functions; mutant shows defect in CPY processing; YLR065C is not an essential gene	-2.42032	-1.2752	down
1779029_at	BUD20	YLR074C	Protein involved in bud-site selection; diploid mutants display a random budding pattern instead of the wild-type bipolar pattern	-2.42033	-1.2752	down
1774383_at	SUB2	YDL084W	Component of the TREX complex required for nuclear mRNA export; member of the DEAD-box RNA helicase superfamily and is involved in early and late steps of spliceosome assembly; homolog of the human splicing factor hUAP56	-2.42066	-1.2754	down

1769955_at	CTT1	YGR088W	Cytosolic catalase T, has a role in protection from oxidative damage by hydrogen peroxide	-2.42231	-1.27638	down
1779674_at	TIM50	YPL063W	Essential component of the Translocase of the Inner Mitochondrial membrane (TIM23 complex); acts as receptor for the TIM23 complex guiding incoming precursors from the TOM complex; may control the gating of the Tim23p-Tim17p channel	-2.42466	-1.27778	down
1771193_at	TAT2	YOL020W	High affinity tryptophan and tyrosine permease, overexpression confers FK506 and FTY720 resistance	-2.42503	-1.278	down
1776877_at	SES1	YDR023W	Cytosolic seryl-tRNA synthetase, class II aminoacyl-tRNA synthetase that aminoacylates tRNA(Ser), displays tRNA-dependent amino acid recognition which enhances discrimination of the serine substrate, interacts with peroxin Pex21p	-2.42957	-1.2807	down
1778980_at	UTP4	YDR324C	Subunit of U3-containing 90S preribosome and Small Subunit (SSU) processome complexes involved in production of 18S rRNA and assembly of small ribosomal subunit; member of t-Utp subcomplex involved with transcription of 35S rRNA transcript	-2.43266	-1.28253	down
1776262_at	HPT1	YDR399W	Dimeric hypoxanthine-guanine phosphoribosyltransferase, catalyzes the transfer of the phosphoribosyl portion of 5-phosphoribosyl-alpha-1-pyrophosphate to a purine base (either guanine or hypoxanthine) to form pyrophosphate and a purine nucleotide (either guanosine monophosphate or inosine monophosphate); mutations in the human homolog HPRT1 can cause Lesch-Nyhan syndrome and Kelley-Seegmiller syndrome	-2.43883	-1.28619	down
1777458_at	RPL16B	YNL069C	N-terminally acetylated protein component of the large (60S) ribosomal subunit, binds to 5.8 S rRNA; has similarity to Rpl16Ap, E. coli L13 and rat L13a ribosomal proteins; transcriptionally regulated by Rap1p	-2.43941	-1.28653	down
1771966_at	CNE1	YAL058W	Calnexin; integral membrane ER chaperone involved in folding and quality control of glycoproteins; chaperone activity is inhibited by Mpd1p, with which Cne1p interacts; 24% identical to mammalian calnexin; Ca+ binding not yet shown in yeast	-2.44028	-1.28705	down
1770971_at	RPS13	YDR064W	Protein component of the small (40S) ribosomal subunit; has similarity to E. coli S15 and rat S13 ribosomal proteins	-2.44077	-1.28734	down
1774932_at	RTT10	YPL183C	WD40 domain-containing protein involved in endosomal recycling; forms a complex with Rrt2p that functions in the retromer-mediated pathway for recycling internalized cell-surface proteins; has a role in regulation of Ty1 transposition; human ortholog is WDR6	-2.44213	-1.28814	down
1774721_at	ENP1	YBR247C	Protein associated with U3 and U14 snoRNAs, required for pre-rRNA processing and 40S ribosomal subunit synthesis; localized in the nucleus and concentrated in the nucleolus	-2.44511	-1.2899	down
1772827_at	TRM8	YDL201W	Noncatalytic subunit of a tRNA methyltransferase complex; Trm8p and Trm82p comprise an enzyme that catalyzes a methyl-transfer from S-adenosyl-l-methionine to the N(7) atom of guanine at position 46 in tRNA; Trm8 lacks catalytic activity if not bound to Trm82p	-2.44886	-1.29211	down
1776774_at	CLB2	YPR119W	B-type cyclin involved in cell cycle progression; activates Cdc28p to promote the transition from G2 to M phase; accumulates during G2 and M, then targeted via a destruction box motif for ubiquitin-mediated degradation by the proteasome	-2.44887	-1.29212	down

1776068_at	SVS1	YPL163C	Cell wall and vacuolar protein, required for wild-type resistance to vanadate	-2.45163	-1.29374	down
1771820_at	RPC10	YHR143W-A	RNA polymerase subunit ABC10-alpha, found in RNA polymerase complexes I, II, and III	-2.45291	-1.29449	down
1770173_at	SSB1	YDL229W	Cytoplasmic ATPase that is a ribosome-associated molecular chaperone, functions with J-protein partner Zuo1p; may be involved in folding of newly-made polypeptide chains; member of the HSP70 family; interacts with phosphatase subunit Reg1p	-2.45569	-1.29613	down
1771763_at		YER163C		-2.45677	-1.29676	down
1773465_at	CSF1	YLR087C	Protein required for fermentation at low temperature; the authentic, non-tagged protein is detected in highly purified mitochondria in high-throughput studies	-2.45828	-1.29765	down
1775293_at	TIF34	YMR146C	eIF3i subunit of the core complex of translation initiation factor 3 (eIF3), which is essential for translation; stimulates rate of ribosomal scanning during translation reinitiation	-2.45863	-1.29785	down
1776049_at	RPS19B	YNL302C	Protein component of the small (40S) ribosomal subunit, required for assembly and maturation of pre-40 S particles; mutations in human RPS19 are associated with Diamond Blackfan anemia; nearly identical to Rps19Ap	-2.46036	-1.29887	down
1775532_at	NHP6A	YPR052C	High-mobility group (HMG) protein that binds to and remodels nucleosomes; involved in recruiting FACT and other chromatin remodelling complexes to the chromosomes; functionally redundant with Nhp6Bp; homologous to mammalian HMGB1 and HMGB2	-2.46287	-1.30034	down
1771956_at	APT1	YML022W	Adenine phosphoribosyltransferase, catalyzes the formation of AMP from adenine and 5-phosphoribosylpyrophosphate; involved in the salvage pathway of purine nucleotide biosynthesis	-2.4637	-1.30083	down
1778252_at	ADE5,7	YGL234W	Bifunctional enzyme of the 'de novo' purine nucleotide biosynthetic pathway, contains aminoimidazole ribotide synthetase and glycinamide ribotide synthetase activities	-2.46479	-1.30147	down
1774289_at	FUN26	YAL022C	Vacuolar membrane transporter with broad nucleoside selectivity; may regulate balance of nicotinamide riboside (NmR) levels between cytosol and vacuole, contributing to salvage of NmR for use in cytosolic NAD+ synthesis	-2.46961	-1.30428	down
1771992_at	NOP4	YPL043W	Nucleolar protein, essential for processing and maturation of 27S pre-rRNA and large ribosomal subunit biogenesis; constituent of 66S pre-ribosomal particles; contains four RNA recognition motifs (RRMs)	-2.47127	-1.30525	down
1776199_at	RPS23B	YPR132W	Ribosomal protein 28 (rp28) of the small (40S) ribosomal subunit, required for translational accuracy; nearly identical to Rps23Ap and similar to E. coli S12 and rat S23 ribosomal proteins; deletion of both RPS23A and RPS23B is lethal	-2.4732	-1.30638	down
1770677_at	BUD31	YCR063W	Component of the SF3b subcomplex of the U2 snRNP; diploid mutants display a random budding pattern instead of the wild-type bipolar pattern	-2.47373	-1.30669	down
1776501_at	CIN4	YMR138W	GTP-binding protein involved in beta-tubulin (Tub2p) folding; isolated as mutant with increased chromosome loss and sensitivity to benomyl; regulated by the GTPase-activating protein, Cin2p, the human retinitis pigmentosa 2 (RP2) homolog	-2.47996	-1.31032	down
1772317_at	MES1	YGR264C	Methionyl-tRNA synthetase, forms a complex with glutamyl-tRNA synthetase (Gus1p) and Arc1p, which increases the catalytic efficiency of both tRNA synthetases; also has a role in nuclear export of tRNAs	-2.48252	-1.31181	down

1770136_at	RPS5	YJR123W	Protein component of the small (40S) ribosomal subunit, the least basic of the non-acidic ribosomal proteins; phosphorylated in vivo; essential for viability; has similarity to E. coli S7 and rat S5 ribosomal proteins	-2.48267	-1.31189	down
1779773_at	CMS1	YLR003C	Putative subunit of the 90S preribosome processome complex; overexpression rescues supressor mutant of mcm10; null mutant is viable	-2.48664	-1.3142	down
1774993_at	CHC1	YGL206C	Clathrin heavy chain, subunit of the major coat protein involved in intracellular protein transport and endocytosis; two heavy chains form the clathrin triskelion structural component; the light chain (CLC1) is thought to regulate function	-2.4873	-1.31458	down
1773828_at	SUN4	YNL066W	Cell wall protein related to glucanases, possibly involved in cell wall septation; member of the SUN family	-2.4901	-1.3162	down
1770113_at	SEC21	YNL287W	Gamma subunit of coatomer, a heptameric protein complex that together with Arf1p forms the COPI coat; involved in ER to Golgi transport of selective cargo	-2.49089	-1.31666	down
1773669_at	UTP23	YOR004W	Component of the small subunit processome, involved in 40S ribosomal subunit biogenesis; interacts with snR30 and is required for dissociation of snR30 from large pre-ribosomal particles; has homology to PINc domain protein Fcf1p, although the PINc domain of Utp23p is not required for function; essential protein	-2.49195	-1.31727	down
1778316_at	GSH2	YOL049W	Glutathione synthetase, catalyzes the ATP-dependent synthesis of glutathione (GSH) from gamma-glutamylcysteine and glycine; induced by oxidative stress and heat shock	-2.49273	-1.31773	down
1770680_at	SUA5	YGL169W	Single-stranded telomeric DNA-binding protein, required for normal telomere length; null mutant lacks N6-threonylcarbamoyl adenosine (t6A) modification in the anticodon loop of ANN-decoding tRNA; member of conserved YrdC/Sua5 family	-2.49302	-1.31789	down
1774720_at	NAF1	YNL124W	RNA-binding protein required for the assembly of box H/ACA snoRNPs and thus for pre-rRNA processing, forms a complex with Shq1p and interacts with H/ACA snoRNP components Nhp2p and Cbf5p; similar to Gar1p	-2.49783	-1.32068	down
1777206_at	NOP9	YJL010C	Essential subunit of U3-containing 90S preribosome involved in production of 18S rRNA and assembly of small ribosomal subunit; also part of pre-40S ribosome and required for its export into cytoplasm; binds RNA and contains pumilio domain	-2.49833	-1.32097	down
1777295_at	RRP42	YDL111C	Exosome non-catalytic core component; involved in 3'-5' RNA processing and degradation in both the nucleus and the cytoplasm; has similarity to E. coli RNase PH and to human hRrp42p (EXOSC7)	-2.50442	-1.32448	down
1776452_at	TIM44	YIL022W	Essential component of the Translocase of the Inner Mitochondrial membrane (TIM23 complex); tethers the import motor and regulatory factors (PAM complex) to the translocation channel (Tim23p-Tim17p core complex)	-2.50465	-1.32461	down
1779920_at		YER034W		-2.50501	-1.32481	down
1777021_at	RCF2	YNR018W	Cytochrome c oxidase subunit; has a role in assembly of respiratory supercomplexes; similar to Rcf1p, and either Rcf1p or Rcf2p is required for late-stage assembly of the Cox12p and Cox13p subunits and for cytochrome c oxidase activity; associates with the cytochrome c oxidase - cytochrome bc1 supercomplex; null mutant accumulates reactive oxygen species; member of	-2.50672	-1.3258	down

			the conserved hypoxia induced gene family; C. elegans homolog is functional in yeast			
1769743_s_at	RPL35 A	YDL191W	Ribosomal 60S subunit protein L35A; homologous to mammalian ribosomal protein L35 and bacterial L29	-2.51219	-1.32895	down
1775844_at	NMT1	YLR195C	N-myristoyl transferase, catalyzes the cotranslational, covalent attachment of myristic acid to the N-terminal glycine residue of several proteins involved in cellular growth and signal transduction	-2.51479	-1.33044	down
1774265_at	SUP35	YDR172W	Translation termination factor eRF3, has a role in mRNA deadenylation and decay; altered protein conformation creates the [PSI(+)] prion that alters translational fidelity and results in a nonsense suppressor phenotype	-2.51638	-1.33135	down
1775057_at	MF(AL PHA)1	YPL187W	Mating pheromone alpha-factor, made by alpha cells; interacts with mating type a cells to induce cell cycle arrest and other responses leading to mating; also encoded by MF(ALPHA)2, although MF(ALPHA)1 produces most alpha-factor	-2.51783	-1.33218	down
1769448_at	SPO12	YHR152W	Nucleolar protein of unknown function, positive regulator of mitotic exit; involved in regulating release of Cdc14p from the nucleolus in early anaphase, may play similar role in meiosis	-2.51904	-1.33287	down
1773327_at	PFA4	YOL003C	Palmitoyltransferase with autoacylation activity, required for palmitoylation of amino acid permeases containing a C-terminal Phe-Trp-Cys site; required for modification of Chs3p; member of the DHHC family of putative palmitoyltransferases	-2.52148	-1.33427	down
1779272_at	SDS23	YGL056C	One of two S. cerevisiae homologs (Sds23p and Sds24p) of the S. pombe Sds23 protein, which is implicated in APC/cyclosome regulation; involved in cell separation during budding	-2.52205	-1.3346	down
1780001_at	GDA1	YEL042W	Guanosine diphosphatase located in the Golgi, involved in the transport of GDP-mannose into the Golgi lumen by converting GDP to GMP after mannose is transferred its substrate	-2.52231	-1.33474	down
1780247_at	WTM2	YOR229W	Transcriptional modulator involved in regulation of meiosis, silencing, and expression of RNR genes; involved in response to replication stress; contains WD repeats	-2.52276	-1.335	down
1775699_at	HOG1	YLR113W	Mitogen-activated protein kinase involved in osmoregulation; acts via three independent osmosensors; mitophagy-specific regulator; mediates the recruitment and activation of RNA Pol II at Hot1p-dependent promoters; localization regulated by Ptp2p and Ptp3p	-2.52515	-1.33637	down
1780116_at	RPS21B	YJL136C	Protein component of the small (40S) ribosomal subunit; nearly identical to Rps21Ap and has similarity to rat S21 ribosomal protein	-2.52691	-1.33737	down
1776314_at		YDR124W		-2.52761	-1.33777	down
1772638_at	RPL26 A	YLR344W	Protein component of the large (60S) ribosomal subunit, nearly identical to Rpl26Bp and has similarity to E. coli L24 and rat L26 ribosomal proteins; binds to 5.8S rRNA	-2.52763	-1.33779	down
1776151_at	MTO1	YGL236C	Mitochondrial protein, forms a heterodimer complex with Mss1p that performs the 5-carboxymethylaminomethyl modification of the wobble uridine base in mitochondrial tRNAs; required for respiration in paromomycin-resistant 15S rRNA mutants	-2.52785	-1.33791	down
1772186_at	NUP157	YER105C	Subunit of the inner ring of the nuclear pore complex (NPC); contributes to NPC assembly and tethering of DNA to the nuclear periphery; has similarity to Nup170p; both Nup170p and NUP157p are similar to human Nup155p	-2.52864	-1.33836	down

1776601_at	TOM5	YPR133W-A	Component of the TOM (translocase of outer membrane) complex responsible for recognition and initial import of all mitochondrially directed proteins; involved in transfer of precursors from the Tom70p and Tom20p receptors to the Tom40p pore	-2.52875	-1.33843	down
1778303_at	RPL29	YFR032C-A	Protein component of the large (60S) ribosomal subunit, has similarity to rat L29 ribosomal protein; not essential for translation, but required for proper joining of the large and small ribosomal subunits and for normal translation rate	-2.53152	-1.34	down
1775553_at	KAP95	YLR347C	Karyopherin beta, forms a complex with Srp1p/Kap60p; interacts with nucleoporins to mediate nuclear import of NLS-containing cargo proteins via the nuclear pore complex; regulates PC biosynthesis; GDP-to-GTP exchange factor for Gsp1p	-2.53237	-1.34049	down
1771271_at	SHB17	YKR043C	Sedoheptulose bisphosphatase involved in riboneogenesis; dephosphorylates sedoheptulose 1,7-bisphosphate, which is converted via the nonoxidative pentose phosphate pathway to ribose-5-phosphate; facilitates the conversion of glycolytic intermediates to pentose phosphate units; also has fructose 1,6-bisphosphatase activity but this is probably not biologically relevant, since deletion does not affect FBP levels; GFP-fusion protein localizes to the cytoplasm and nucleus	-2.53248	-1.34055	down
1773506_at		YLR173W		-2.53329	-1.34101	down
1773910_at	TIM12	YBR091C	Essential protein of the inner mitochondrial membrane, peripherally localized; component of the TIM22 complex, which is a twin-pore translocase that mediates insertion of numerous multispanning inner membrane proteins	-2.53751	-1.34341	down
1776097_at	PRS3	YHL011C	5-phospho-ribosyl-1(alpha)-pyrophosphate synthetase, synthesizes PRPP, which is required for nucleotide, histidine, and tryptophan biosynthesis; one of five related enzymes, which are active as heteromultimeric complexes	-2.53865	-1.34406	down
1775832_at	RPS20	YHL015W	Protein component of the small (40S) ribosomal subunit; overproduction suppresses mutations affecting RNA polymerase III-dependent transcription; has similarity to E. coli S10 and rat S20 ribosomal proteins	-2.5406	-1.34517	down
1773941_at	RRT14	YIL127C	Putative protein of unknown function; identified in a screen for mutants with decreased levels of rDNA transcription; green fluorescent protein (GFP)-fusion protein localizes to the nucleolus; predicted to be involved in ribosome biogenesis	-2.54101	-1.3454	down
1780226_at	USA1	YML029W	Scaffold subunit of the Hrd1p ubiquitin ligase that also promotes ligase oligomerization; involved in ER-associated protein degradation (ERAD); interacts with the U1 snRNP-specific protein, Snp1p	-2.54709	-1.34885	down
1780055_at	VAS1	YGR094W	Mitochondrial and cytoplasmic valyl-tRNA synthetase	-2.5481	-1.34942	down
1774600_at	XRN1	YGL173C	Evolutionarily-conserved 5'-3' exonuclease component of cytoplasmic processing (P) bodies involved in mRNA decay; plays a role in microtubule-mediated processes, filamentous growth, ribosomal RNA maturation, and telomere maintenance; activated by the scavenger decapping enzyme Dcs1p	-2.55383	-1.35266	down
1774332_at	SEC31	YDL195W	Component of the Sec13p-Sec31p complex of the COPII vesicle coat, required for vesicle formation in ER to Golgi transport; mutant has increased aneuploidy tolerance	-2.55756	-1.35477	down

1775213_at	MNS1	YJR131W	Alpha-1,2-mannosidase involved in ER-associated protein degradation (ERAD); catalyzes the removal of one mannose residue from a glycosylated protein, converting the modification from Man9GlcNAc to Man8GlcNAc; catalyzes the last step in glycoprotein maturation in the ER and is critical for ER protein degradation	-2.55839	-1.35524	down
1772888_at	YBT1	YLL048C	Transporter of the ATP-binding cassette (ABC) family involved in bile acid transport; similar to mammalian bile transporters	-2.55975	-1.35601	down
1778653_at	RIX7	YLL034C	Putative ATPase of the AAA family, required for export of pre-ribosomal large subunits from the nucleus; distributed between the nucleolus, nucleoplasm, and nuclear periphery depending on growth conditions	-2.56296	-1.35781	down
1774556_at	HCH1	YNL281W	Heat shock protein regulator that binds to Hsp90p and may stimulate ATPase activity; originally identified as a high-copy number suppressor of a HSP90 loss-of-function mutation; GFP-fusion protein localizes to the cytoplasm and nucleus	-2.56428	-1.35856	down
1773209_at	ORC5	YNL261W	Subunit of the origin recognition complex, which directs DNA replication by binding to replication origins and is also involved in transcriptional silencing	-2.56528	-1.35912	down
1771566_at	ERP1	YAR002C-A	Protein that forms a heterotrimeric complex with Erp2p, Emp24p, and Erv25p; member, along with Emp24p and Erv25p, of the p24 family involved in ER to Golgi transport and localized to COPII-coated vesicles	-2.56622	-1.35965	down
1770571_at	MVD1	YNR043W	Mevalonate pyrophosphate decarboxylase, essential enzyme involved in the biosynthesis of isoprenoids and sterols, including ergosterol; acts as a homodimer	-2.57024	-1.3619	down
1771579_at	RPL25	YOL127W	Primary rRNA-binding ribosomal protein component of the large (60S) ribosomal subunit, has similarity to E. coli L23 and rat L23a ribosomal proteins; binds to 25S rRNA via a conserved C-terminal motif	-2.57149	-1.36261	down
1779536_at	MAK3	YPR051W	Catalytic subunit of N-terminal acetyltransferase of the NatC type; required for replication of dsRNA virus	-2.5715	-1.36261	down
1779576_at	ERV29	YGR284C	Protein localized to COPII-coated vesicles, involved in vesicle formation and incorporation of specific secretory cargo	-2.57264	-1.36325	down
1776365_s_at	RPS24A	YER074W	Protein component of the small (40S) ribosomal subunit; homologous to mammalian ribosomal protein S24	-2.5739	-1.36396	down
1774627_at	PMI40	YER003C	Mannose-6-phosphate isomerase, catalyzes the interconversion of fructose-6-P and mannose-6-P; required for early steps in protein mannosylation	-2.57511	-1.36463	down
1775694_at	PSE1	YMR308C	Karyopherin/importin that interacts with the nuclear pore complex; acts as the nuclear import receptor for specific proteins, including Pdr1p, Yap1p, Ste12p, and Aft1p	-2.57559	-1.3649	down
1780184_at		YNR021W		-2.57561	-1.36492	down
1777456_at	OXP1	YKL215C	5-oxoprolinase; enzyme is ATP-dependent and functions as a dimer; similar to mouse Oplah gene; green fluorescent protein (GFP)-fusion protein localizes to the cytoplasm	-2.57682	-1.36559	down
1774621_at	RPL36 A	YMR194W	N-terminally acetylated protein component of the large (60S) ribosomal subunit, nearly identical to Rpl36Bp and has similarity to rat L36 ribosomal protein; binds to 5.8 S rRNA	-2.57685	-1.36561	down
1769378_at	SEC53	YFL045C	Phosphomannomutase, involved in synthesis of GDP-mannose and dolichol-phosphate-mannose; required for folding and glycosylation of secretory	-2.5794	-1.36704	down

			proteins in the ER lumen			
1773369_at	ALO1	YML086C	D-Arabinono-1,4-lactone oxidase, catalyzes the final step in biosynthesis of dehydro-D-arabinono-1,4-lactone, which is protective against oxidative stress	-2.57955	-1.36712	down
1775186_at	RPS14B	YJL191W	Ribosomal protein 59 of the small subunit, required for ribosome assembly and 20S pre-rRNA processing; mutations confer cryptopleurine resistance; nearly identical to Rps14Ap and similar to E. coli S11 and rat S14 ribosomal proteins	-2.57961	-1.36715	down
1774101_at	THO2	YNL139C	Subunit of the THO complex, which is required for efficient transcription elongation and involved in transcriptional elongation-associated recombination; required for LacZ RNA expression from certain plasmids	-2.58526	-1.37031	down
1769945_at	SRP40	YKR092C	Nucleolar, serine-rich protein with a role in preribosome assembly or transport; may function as a chaperone of small nucleolar ribonucleoprotein particles (snoRNPs); immunologically and structurally to rat Nopp140	-2.58814	-1.37192	down
1777509_at	CAF120	YNL278W	Part of the evolutionarily-conserved CCR4-NOT transcriptional regulatory complex involved in controlling mRNA initiation, elongation, and degradation	-2.58846	-1.37209	down
1779447_at	SKI6	YGR195W	Exosome non-catalytic core component; involved in 3'-5' RNA processing and degradation in both the nucleus and the cytoplasm; has similarity to E. coli RNase PH and to human hRrp41p (EXOSC4)	-2.59023	-1.37308	down
1769640_at	ELP4	YPL101W	Subunit of hexameric RecA-like ATPase Elp456 Elongator subcomplex; which is required for modification of wobble nucleosides in tRNA; required for Elongator structural integrity	-2.59029	-1.37312	down
1774053_at	EFM2	YBR271W	S-adenosylmethionine-dependent methyltransferase; methylates translation elongation factors EF2 (Eft1p and Eft2p) and EF3A (Yef3p); belongs to the seven beta-strand family; green fluorescent protein (GFP)-fusion protein localizes to the cytoplasm; predicted to be involved in ribosome biogenesis	-2.59611	-1.37635	down
1772207_at	APL3	YBL037W	Alpha-adaptin, large subunit of the clathrin associated protein complex (AP-2); involved in vesicle mediated transport	-2.59673	-1.3767	down
1777386_at	PAT1	YCR077C	Topoisomerase II-associated deadenylation-dependent mRNA-decapping factor; also required for faithful chromosome transmission, maintenance of rDNA locus stability, and protection of mRNA 3'-UTRs from trimming; functionally linked to Pab1p	-2.59831	-1.37757	down
1778199_at	JEM1	YJL073W	DnaJ-like chaperone required for nuclear membrane fusion during mating, localizes to the ER membrane; exhibits genetic interactions with KAR2	-2.59967	-1.37833	down
1772089_at	VPS8	YAL002W	Membrane-binding component of the CORVET complex; involved in endosomal vesicle tethering and fusion in the endosome to vacuole protein targeting pathway; interacts with Vps21p; contains RING finger motif	-2.60093	-1.37903	down
1779491_at	NEW1	YPL226W	ATP binding cassette protein that cosediments with polysomes and is required for biogenesis of the small ribosomal subunit; Asn/Gln-rich rich region supports [NU+] prion formation and susceptibility to [PSI+] prion induction	-2.6039	-1.38067	down
1778711_at		YER152C		-2.60486	-1.38121	down
1773276_at	TAF5	YBR198C	Subunit (90 kDa) of TFIID and SAGA complexes, involved in RNA polymerase II transcription	-2.60827	-1.38309	down

			initiation and in chromatin modification			
1778046_at	ROT2	YBR229C	Glucosidase II catalytic subunit required for normal cell wall synthesis; mutations in rot2 suppress tor2 mutations, and are synthetically lethal with rot1 mutations	-2.60851	-1.38322	down
1780076_at	TIF11	YMR260C	Translation initiation factor eIF1A, essential protein that forms a complex with Sui1p (eIF1) and the 40S ribosomal subunit and scans for the start codon; C-terminus associates with Fun12p (eIF5B); N terminus interacts with eIF2 and eIF3	-2.61411	-1.38632	down
1769903_at	TUB4	YLR212C	Gamma-tubulin, involved in nucleating microtubules from both the cytoplasmic and nuclear faces of the spindle pole body	-2.61449	-1.38653	down
1776115_at	TRM9	YML014W	tRNA methyltransferase, catalyzes esterification of modified uridine nucleotides in tRNA(Arg3) and tRNA(Glu), likely as part of a complex with Trm112p; deletion confers resistance to zymocin	-2.61647	-1.38762	down
1773086_at	TRM82	YDR165W	Catalytic subunit of a tRNA methyltransferase complex; Trm8p and Trm82p comprise an enzyme that catalyzes a methyl-transfer from S-adenosyl-l-methionine to the N(7) atom of guanine at position 46 in tRNA; Trm8 lacks catalytic activity if not bound to Trm82p	-2.61724	-1.38804	down
1769845_at	GUT1	YHL032C	Glycerol kinase, converts glycerol to glycerol-3-phosphate; glucose repression of expression is mediated by Adr1p and Ino2p-Ino4p; derepression of expression on non-fermentable carbon sources is mediated by Opi1p and Rsf1p	-2.61784	-1.38838	down
1769534_at	EFR3	YMR212C	Protein required for Stt4-containing phosphoinositide kinase patch assembly at the plasma membrane; exhibits synthetic lethal genetic interactions with PHO85; has sequence similarity to the Drosophila rolling blackout (RBO) gene	-2.61963	-1.38936	down
1773295_at	INP52	YNL106C	Polyphosphatidylinositol phosphatase, dephosphorylates a number of phosphatidylinositols (PIs) to PI; involved in endocytosis; hyperosmotic stress causes translocation to actin patches; synaptojanin-like protein with a Sac1 domain	-2.61964	-1.38937	down
1772590_at	DNF1	YER166W	Aminophospholipid translocase (flippase) that localizes primarily to the plasma membrane; contributes to endocytosis, protein transport and cell polarity; type 4 P-type ATPase	-2.62422	-1.39189	down
1777661_at	STT4	YLR305C	Phosphatidylinositol-4-kinase that functions in the Pkc1p protein kinase pathway; required for normal vacuole morphology, cell wall integrity, and actin cytoskeleton organization	-2.62492	-1.39227	down
1773461_at	VAN1	YML115C	Component of the mannan polymerase I, which contains Van1p and Mnn9p and is involved in the first steps of mannan synthesis; mutants are vanadate-resistant	-2.62664	-1.39322	down
1778031_at	TGS1	YPL157W	Trimethyl guanosine synthase, conserved nucleolar methyl transferase that converts the m(7)G cap structure of snRNAs, snoRNAs, and telomerase TLC1 RNA to m(2,2,7)G; also required for nucleolar assembly and splicing of meiotic pre-mRNAs	-2.62752	-1.3937	down
1773300_at	SNU13	YEL026W	RNA binding protein, part of U3 snoRNP involved in rRNA processing, part of U4/U6-U5 tri-snRNP involved in mRNA splicing, similar to human 15.5K protein	-2.63545	-1.39805	down
1770267_at	ATM1	YMR301C	Mitochondrial inner membrane ATP-binding cassette (ABC) transporter, exports mitochondrially synthesized precursors of iron-sulfur (Fe/S) clusters to the cytosol	-2.64073	-1.40094	down

1771085_at	RPL18 A	YOL120C	Protein component of the large (60S) ribosomal subunit, identical to Rpl18Bp and has similarity to rat L18 ribosomal protein; intron of RPL18A pre-mRNA forms stem-loop structures that are a target for Rnt1p cleavage leading to degradation	-2.64298	-1.40216	down
1777016_at		YBL081W		-2.64429	-1.40288	down
1777153_at	MRD1	YPR112C	Essential conserved protein that is part of the 90S preribosome; required for production of 18S rRNA and small ribosomal subunit; contains five consensus RNA-binding domains	-2.64458	-1.40304	down
1771658_at	NIP1	YMR309C	eIF3c subunit of the eukaryotic translation initiation factor 3 (eIF3), involved in the assembly of preinitiation complex and start codon selection	-2.64481	-1.40317	down
1774175_at	ESF2	YNR054C	Essential nucleolar protein involved in pre-18S rRNA processing; binds to RNA and stimulates ATPase activity of Dbp8; involved in assembly of the small subunit (SSU) processome	-2.64764	-1.4047	down
1778270_at	HO	YDL227C	Site-specific endonuclease required for gene conversion at the MAT locus (homothallic switching) through the generation of a ds DNA break; expression restricted to mother cells in late G1 as controlled by Swi4p-Swi6p, Swi5p and Ash1p	-2.65154	-1.40683	down
1769383_at	MRS1	YIR021W	Protein required for the splicing of two mitochondrial group I introns (BI3 in COB and AI5beta in COX1); forms a splicing complex, containing four subunits of Mrs1p and two subunits of the BI3-encoded maturase, that binds to the BI3 RNA	-2.65207	-1.40712	down
1773892_at	NCS2	YNL119W	Protein required for thiolation of the uridine at the wobble position of Lys(UUU) and Glu(UUC) tRNAs; has a role in urmylation and in invasive and pseudohyphal growth; inhibits replication of Brome mosaic virus in S. cerevisiae	-2.65321	-1.40774	down
1779304_at	BPL1	YDL141W	Biotin:apoprotein ligase, covalently modifies proteins with the addition of biotin, required for acetyl-CoA carboxylase (Acc1p) holoenzyme formation	-2.65528	-1.40887	down
1774613_at	IMP2'	YIL154C	Transcriptional activator involved in maintenance of ion homeostasis and protection against DNA damage caused by bleomycin and other oxidants, contains a C-terminal leucine-rich repeat	-2.6584	-1.41056	down
1770935_at	LAA1	YJL207C	AP-1 accessory protein; colocalizes with clathrin to the late-Golgi apparatus; involved in TGN-endosome transport; physically interacts with AP-1; similar to the mammalian p200; may interact with ribosomes; YJL207C is a non-essential gene	-2.66103	-1.41198	down
1772978_at	CYM1	YDR430C	Lysine-specific metalloprotease of the mitochondrial intermembrane space, member of the pitrilysin family; degrades proteins and presequence peptides cleaved from imported proteins; required for normal mitochondrial morphology	-2.66179	-1.4124	down
1779982_at	RPL32	YBL092W	Protein component of the large (60S) ribosomal subunit, has similarity to rat L32 ribosomal protein; overexpression disrupts telomeric silencing	-2.66508	-1.41418	down
1774718_at	TIF4631	YGR162W	Translation initiation factor eIF4G, subunit of the mRNA cap-binding protein complex (eIF4F) that also contains eIF4E (Cdc33p); interacts with Pab1p and with eIF4A (Tif1p); also has a role in biogenesis of the large ribosomal subunit	-2.6779	-1.4211	down
1770792_at	THI7	YLR237W	Plasma membrane transporter responsible for the uptake of thiamine, member of the major facilitator superfamily of transporters; mutation of human ortholog causes thiamine-responsive megaloblastic anemia	-2.67863	-1.42149	down

1773582_at	MDS3	YGL197W	Putative component of the TOR regulatory pathway; negative regulator of early meiotic gene expression; required, with Pmd1p, for growth under alkaline conditions; has an N-terminal kelch-like domain	-2.68111	-1.42283	down
1771027_at	TIF3	YPR163C	Translation initiation factor eIF-4B, has RNA annealing activity; contains an RNA recognition motif and binds to single-stranded RNA	-2.68603	-1.42548	down
1772824_at	STB3	YDR169C	Ribosomal RNA processing element (RRPE)-binding protein involved in the glucose-induced transition from quiescence to growth; restricted to nucleus in quiescent cells, released into cytoplasm after glucose repletion; binds Sin3p	-2.68666	-1.42581	down
1773285_at	RHO2	YNL090W	Non-essential small GTPase of the Rho/Rac subfamily of Ras-like proteins, involved in the establishment of cell polarity and in microtubule assembly	-2.69054	-1.42789	down
1770281_at	ANP1	YEL036C	Subunit of the alpha-1,6 mannosyltransferase complex; type II membrane protein; has a role in retention of glycosyltransferases in the Golgi; involved in osmotic sensitivity and resistance to aminonitrophenyl propanediol	-2.69194	-1.42865	down
1769779_at	ABP1	YCR088W	Actin-binding protein of the cortical actin cytoskeleton, important for activation of the Arp2/3 complex that plays a key role actin in cytoskeleton organization; phosphorylation within its PRR (Proline-Rich Region), mediated by Cdc28p and Pho85p, protects Abp1p from proteolysis mediated by its own PEST sequences	-2.69347	-1.42946	down
1770683_at	SLD5	YDR489W	Subunit of the GINS complex (Sld5p, Psf1p, Psf2p, Psf3p), which is localized to DNA replication origins and implicated in assembly of the DNA replication machinery	-2.6957	-1.43066	down
1778219_at	RPB8	YOR224C	RNA polymerase subunit ABC14.5, common to RNA polymerases I, II, and III	-2.69609	-1.43087	down
1779268_at	SRB2	YHR041C	Subunit of the RNA polymerase II mediator complex; associates with core polymerase subunits to form the RNA polymerase II holoenzyme; general transcription factor involved in telomere maintenance	-2.69728	-1.43151	down
1776278_at	RPS7B	YNL096C	Protein component of the small (40S) ribosomal subunit, nearly identical to Rps7Ap; interacts with Kti11p; deletion causes hypersensitivity to zymocin; has similarity to rat S7 and Xenopus S8 ribosomal proteins	-2.70365	-1.43491	down
1779080_at	SEC20	YDR498C	Membrane glycoprotein v-SNARE involved in retrograde transport from the Golgi to the ER; required for N- and O-glycosylation in the Golgi but not in the ER; interacts with the Dsl1p complex through Tip20p	-2.70438	-1.4353	down
1772251_at	RPL39	YJL189W	Protein component of the large (60S) ribosomal subunit, has similarity to rat L39 ribosomal protein; required for ribosome biogenesis; loss of both Rpl31p and Rpl39p confers lethality; also exhibits genetic interactions with SIS1 and PAB1	-2.70858	-1.43754	down
1771012_at	TSR1	YDL060W	Protein required for processing of 20S pre-rRNA in the cytoplasm; associates with pre-40S ribosomal particles; inhibits the premature association of 60S subunits with assembling 40S subunits in the cytoplasm; similar to Bms1p	-2.71015	-1.43838	down
1771324_at	KRR1	YCL059C	Essential nucleolar protein required for the synthesis of 18S rRNA and for the assembly of 40S ribosomal subunit	-2.71066	-1.43864	down
1777051_at	ELP3	YPL086C	Subunit of Elongator complex, which is required for modification of wobble nucleosides in tRNA; exhibits histone acetyltransferase activity that is	-2.71291	-1.43984	down

			directed to histones H3 and H4; disruption confers resistance to K. lactis zymotoxin			
1779959_at	AVT6	YER119C	Vacuolar aspartate and glutamate exporter; member of a family of seven genes (AVT1-7) related to vesicular GABA-glycine transporters; involved in compartmentalizing acidic amino acids in response to nitrogen starvation	-2.71389	-1.44036	down
1775436_at	SCT1	YBL011W	Glycerol 3-phosphate/dihydroxyacetone phosphate dual substrate-specific sn-1 acyltransferase of the glycerolipid biosynthesis pathway, prefers 16-carbon fatty acids, similar to Gpt2p, gene is constitutively transcribed	-2.7152	-1.44106	down
1773028_at	RSM7	YJR113C	Mitochondrial ribosomal protein of the small subunit, has similarity to E. coli S7 ribosomal protein	-2.7157	-1.44133	down
1771499_at	CYB5	YNL111C	Cytochrome b5, involved in the sterol and lipid biosynthesis pathways; acts as an electron donor to support sterol C5-6 desaturation	-2.71611	-1.44154	down
1770709_at	TMA20	YER007C-A	Protein of unknown function that associates with ribosomes and has a putative RNA binding domain; interacts with Tma22p; null mutant exhibits translation defects; has homology to human oncogene MCT-1	-2.71831	-1.44271	down
1771353_at	GRS1	YBR121C	Cytoplasmic and mitochondrial glycyl-tRNA synthase that ligates glycine to the cognate anticodon bearing tRNA; transcription termination factor that may interact with the 3'-end of pre-mRNA to promote 3'-end formation	-2.71949	-1.44334	down
1774276_at	SSB2	YNL209W	Cytoplasmic ATPase that is a ribosome-associated molecular chaperone, functions with J-protein partner Zuo1p; may be involved in the folding of newly-synthesized polypeptide chains; member of the HSP70 family; homolog of SSB1	-2.7197	-1.44345	down
1778488_at	CCR4	YAL021C	Component of the CCR4-NOT transcriptional complex, which is involved in regulation of gene expression; component of the major cytoplasmic deadenylase, which is involved in mRNA poly(A) tail shortening	-2.72838	-1.44804	down
1770837_at		YGR266W		-2.73038	-1.4491	down
1771246_at	DUT1	YBR252W	deoxyuridine triphosphate diphosphatase (dUTPase); catalyzes hydrolysis of dUTP to dUMP and PPi, thereby preventing incorporation of uracil into DNA during replication; critical for the maintenance of genetic stability; also has diphosphatase activity on deoxyinosine triphosphate	-2.7304	-1.44911	down
1773960_at	RPS16B	YDL083C	Protein component of the small (40S) ribosomal subunit; identical to Rps16Ap and has similarity to E. coli S9 and rat S16 ribosomal proteins	-2.7329	-1.45043	down
1776353_at	DML1	YMR211W	Essential protein involved in mtDNA inheritance, may also function in the partitioning of the mitochondrial organelle or in the segregation of chromosomes, exhibits regions similar to members of a GTPase family	-2.73537	-1.45174	down
1773708_at	PIK1	YNL267W	Phosphatidylinositol 4-kinase; catalyzes first step in the biosynthesis of phosphatidylinositol-4,5-biphosphate; may control cytokinesis through the actin cytoskeleton	-2.7375	-1.45286	down
1770963_at	TRM5	YHR070W	tRNA(m(1)G37)methyltransferase, methylates a tRNA base adjacent to the anticodon that has a role in prevention of frameshifting; highly conserved across Archaea, Bacteria, and Eukarya	-2.73862	-1.45345	down
1769800_at	COX20	YDR231C	Mitochondrial inner membrane protein, required for proteolytic processing of Cox2p and its assembly into cytochrome c oxidase	-2.74521	-1.45692	down

1779333_at	MMR1	YLR190W	Phosphorylated protein of the mitochondrial outer membrane, localizes only to mitochondria of the bud; interacts with Myo2p to mediate mitochondrial distribution to buds; mRNA is targeted to the bud via the transport system involving She2p	-2.75048	-1.45968	down
1776363_at	SUR4	YLR372W	Elongase, involved in fatty acid and sphingolipid biosynthesis; synthesizes very long chain 20-26-carbon fatty acids from C18-CoA primers; involved in regulation of sphingolipid biosynthesis	-2.75229	-1.46063	down
1769331_at	STE20	YHL007C	Cdc42p-activated signal transducing kinase of the PAK (p21-activated kinase) family; involved in pheromone response, pseudohyphal/invasive growth, vacuole inheritance, down-regulation of sterol uptake; GBB motif binds Ste4p	-2.75831	-1.46378	down
1769703_at	PHS1	YJL097W	Essential 3-hydroxyacyl-CoA dehydratase of the ER membrane, involved in elongation of very long-chain fatty acids; evolutionarily conserved, similar to mammalian PTPLA and PTPLB; involved in sphingolipid biosynthesis and protein trafficking	-2.75929	-1.4643	down
1772384_at	PCM1	YEL058W	Essential N-acetylglucosamine-phosphate mutase; converts GlcNAc-6-P to GlcNAc-1-P, which is a precursor for the biosynthesis of chitin and for the formation of N-glycosylated mannoproteins and glycosylphosphatidylinositol anchors	-2.76152	-1.46546	down
1775407_at	RPL38	YLR325C	Protein component of the large (60S) ribosomal subunit, has similarity to rat L38 ribosomal protein	-2.76179	-1.4656	down
1779003_at	CDC14	YFR028C	Protein phosphatase required for mitotic exit; located in the nucleolus until liberated by the FEAR and Mitotic Exit Network in anaphase, enabling it to act on key substrates to effect a decrease in CDK/B-cyclin activity and mitotic exit; required for meiosis I spindle disassembly; released from nucleolus upon entry into anaphase I of meiosis, resequestered in metaphase II, then released again upon entry into anaphase II; maintained in nucleolus by Cdc55p in early meiosis	-2.76262	-1.46604	down
1777853_at	FTR1	YER145C	High affinity iron permease involved in the transport of iron across the plasma membrane; forms complex with Fet3p; expression is regulated by iron	-2.7635	-1.4665	down
1777774_at	SRO77	YBL106C	Protein with roles in exocytosis and cation homeostasis; functions in docking and fusion of post-Golgi vesicles with plasma membrane; homolog of Sro7p and Drosophila lethal giant larvae tumor suppressor; interacts with SNARE protein Sec9p	-2.76535	-1.46746	down
1774974_at	IMD4	YML056C	Inosine monophosphate dehydrogenase, catalyzes the first step of GMP biosynthesis, member of a four-gene family in S. cerevisiae, constitutively expressed	-2.76859	-1.46915	down
1770016_at	RIA1	YNL163C	Cytoplasmic GTPase involved in ribosomal biogenesis; with Sdo1p, promotes release of Tif6p from 60S ribosomal subunits in the cytoplasm so that they can assemble with 40S subunits to generate mature ribosomes	-2.77188	-1.47087	down
1772459_at	INH1	YDL181W	Protein that inhibits ATP hydrolysis by the F1F0-ATP synthase; inhibitory function is enhanced by stabilizing proteins Stf1p and Stf2p; has similarity to Stf1p; has a calmodulin-binding motif and binds calmodulin in vitro	-2.77614	-1.47308	down
1779516_at	RRS1	YOR294W	Essential protein that binds ribosomal protein L11; required for nuclear export of the 60S pre-ribosomal subunit during ribosome biogenesis; localizes to the nucleolus and in foci along nuclear periphery; cooperates with Ebp2p and Mps3p to	-2.77619	-1.47311	down

			mediate telomere clustering by binding Sir4p, but is not involved in telomere tethering; mouse homolog shows altered expression in Huntington's disease model mice			
1777542_at	FAS1	YKL182W	Beta subunit of fatty acid synthetase, which catalyzes the synthesis of long-chain saturated fatty acids; contains acetyltransacylase, dehydratase, enoyl reductase, malonyl transacylase, and palmitoyl transacylase activities	-2.77724	-1.47365	down
1778802_at	NNF1	YJR112W	Essential component of the MIND kinetochore complex (Mtw1p Including Nnf1p-Nsl1p-Dsn1p) which joins kinetochore subunits contacting DNA to those contacting microtubules; required for accurate chromosome segregation	-2.7777	-1.47389	down
1772830_at	PMT6	YGR199W	Protein O-mannosyltransferase, transfers mannose from dolichyl phosphate-D-mannose to protein serine/threonine residues of secretory proteins; reaction is essential for cell wall rigidity; member of a family of mannosyltransferases	-2.77853	-1.47432	down
1772250_at	ERV25	YML012W	Protein that forms a heterotrimeric complex with Erp1, Erp2p, and Emp24, member of the p24 family involved in endoplasmic reticulum to Golgi transport	-2.78374	-1.47702	down
1777543_at	EMP24	YGL200C	Component of the p24 complex; binds to GPI anchor proteins and mediates their efficient transport from the ER to the Golgi; integral membrane protein that associates with endoplasmic reticulum-derived COPII-coated vesicles	-2.78631	-1.47835	down
1775999_at	SAM35	YHR083W	Essential component of the sorting and assembly machinery (SAM complex or TOB complex) of the mitochondrial outer membrane, which binds precursors of beta-barrel proteins and facilitates their insertion into the outer membrane	-2.78807	-1.47927	down
1779559_at	RPL43B	YJR094W-A	Protein component of the large (60S) ribosomal subunit, identical to Rpl43Ap and has similarity to rat L37a ribosomal protein	-2.78878	-1.47964	down
1769919_at	RPL5	YPL131W	Protein component of the large (60S) ribosomal subunit with similarity to E. coli L18 and rat L5 ribosomal proteins; binds 5S rRNA and is required for 60S subunit assembly	-2.79478	-1.48274	down
1770026_at	PEP1	YBL017C	Type I transmembrane sorting receptor for multiple vacuolar hydrolases; cycles between the late-Golgi and prevacuolar endosome-like compartments	-2.79624	-1.48349	down
1777130_at	FKH1	YIL131C	Forkhead family transcription factor with a minor role in the expression of G2/M phase genes; negatively regulates transcriptional elongation; positive role in chromatin silencing at HML and HMR; regulates donor preference during switching	-2.79795	-1.48437	down
1774806_at	SHM1	YBR263W	Mitochondrial serine hydroxymethyltransferase, converts serine to glycine plus 5,10 methylenetetrahydrofolate; involved in generating precursors for purine, pyrimidine, amino acid, and lipid biosynthesis; reverse reaction generates serine	-2.79879	-1.4848	down
1778720_at	AHA1	YDR214W	Co-chaperone that binds to Hsp82p and activates its ATPase activity; similar to Hch1p; expression is regulated by stresses such as heat shock	-2.7992	-1.48501	down
1777227_at	RPL23 A	YBL087C	Protein component of the large (60S) ribosomal subunit, identical to Rpl23Bp and has similarity to E. coli L14 and rat L23 ribosomal proteins	-2.80126	-1.48607	down
1773886_at	PCL2	YDL127W	Cyclin, interacts with cyclin-dependent kinase Pho85p; member of the Pcl1,2-like subfamily, involved in the regulation of polarized growth and morphogenesis and progression through the cell cycle; localizes to sites of polarized cell growth	-2.80227	-1.4866	down
1779928_at	DYN1	YKR054C	Cytoplasmic heavy chain dynein, microtubule motor protein, required for anaphase spindle	-2.80609	-1.48856	down

			elongation; involved in spindle assembly, chromosome movement, and spindle orientation during cell division, targeted to microtubule tips by Pac1p			
1772508_at	NOP7	YGR103W	Component of several different pre-ribosomal particles; forms a complex with Ytm1p and Erb1p that is required for maturation of the large ribosomal subunit; required for exit from G₀ and the initiation of cell proliferation	-2.80791	-1.4895	down
1778541_at	PCL9	YDL179W	Cyclin, forms a functional kinase complex with Pho85p cyclin-dependent kinase (Cdk), expressed in late M/early G1 phase, activated by Swi5p	-2.80907	-1.49009	down
1771438_at	RFC1	YOR217W	Subunit of heteropentameric Replication factor C (RF-C), which is a DNA binding protein and ATPase that acts as a clamp loader of the proliferating cell nuclear antigen (PCNA) processivity factor for DNA polymerases delta and epsilon	-2.80909	-1.4901	down
1770074_at		YBR141C		-2.81077	-1.49097	down
1775258_at	MDL1	YLR188W	Mitochondrial inner membrane half-type ATP-binding cassette (ABC) transporter, mediates export of peptides generated upon proteolysis of mitochondrial proteins, plays a role in the regulation of cellular resistance to oxidative stress	-2.81493	-1.4931	down
1778133_s_at	RPL7A	YGL076C	Ribosomal 60S subunit protein L7A; required for processing of 27SA3 pre-rRNA to 27SB pre-rRNA during assembly of large ribosomal subunit; depletion leads to a turnover of pre-rRNA; contains a conserved C-terminal Nucleic acid Binding Domain (NDB2); binds to Domain II of 25S and 5.8S rRNAs; homologous to mammalian ribosomal protein L7 and bacterial L30	-2.81519	-1.49323	down
1773288_at	RPC40	YPR110C	RNA polymerase subunit AC40, common to RNA polymerase I and III	-2.81569	-1.49349	down
1776633_at		YNL247W		-2.81812	-1.49473	down
1773631_at	MIM1	YOL026C	Mitochondrial protein required for outer membrane protein import; cooperates with Tom70p to import the subset of proteins with multiple alpha-helical transmembrane segments, including Ugo1p, Tom20p, and others; present in a complex with Mim2p in the outer membrane that may create a local environment to facilitate membrane insertion of substrate proteins; also has a role in assembly of Tom20p into the TOM complex	-2.81951	-1.49545	down
1774550_at	ISC1	YER019W	Mitochondrial membrane localized inositol phosphosphingolipid phospholipase C, hydrolyzes complex sphingolipids to produce ceramide; activated by phosphatidylserine, cardiolipin, and phosphatidylglycerol; mediates Na+ and Li+ halotolerance	-2.81971	-1.49555	down
1774025_at	HEM3	YDL205C	Porphobilinogen deaminase, catalyzes the conversion of 4-porphobilinogen to hydroxymethylbilane, the third step in heme biosynthesis; localizes to the cytoplasm and nucleus; expression is regulated by Hap2p-Hap3p, but not by levels of heme	-2.82237	-1.4969	down
1771569_at	BNA7	YDR428C	Formylkynurenine formamidase, involved in the de novo biosynthesis of NAD from tryptophan via kynurenine	-2.82253	-1.49699	down
1780080_at	HSL7	YBR133C	Protein arginine N-methyltransferase that exhibits septin and Hsl1p-dependent bud neck localization and periodic Hsl1p-dependent phosphorylation; required along with Hsl1p for bud neck recruitment, phosphorylation, and degradation of	-2.83028	-1.50095	down

			Swe1p			
1773997_at	CEP3	YMR168C	Essential kinetochore protein, component of the CBF3 complex that binds the CDEIII region of the centromere; contains an N-terminal Zn2Cys6 type zinc finger domain, a C-terminal acidic domain, and a putative coiled coil dimerization domain	-2.83201	-1.50182	down
1779156_x_at	RPS16A	YMR143W	Protein component of the small (40S) ribosomal subunit; identical to Rps16Bp and has similarity to E. coli S9 and rat S16 ribosomal proteins	-2.83435	-1.50302	down
1769877_at	RPL34B	YIL052C	Protein component of the large (60S) ribosomal subunit, nearly identical to Rpl34Ap and has similarity to rat L34 ribosomal protein	-2.84074	-1.50627	down
1772975_at	RPS3	YNL178W	Protein component of the small (40S) ribosomal subunit, has apurinic/apyrimidinic (AP) endonuclease activity; essential for viability; has similarity to E. coli S3 and rat S3 ribosomal proteins	-2.84101	-1.5064	down
1778685_at	UTP6	YDR449C	Nucleolar protein, component of the small subunit (SSU) processome containing the U3 snoRNA that is involved in processing of pre-18S rRNA	-2.84342	-1.50763	down
1771921_at	DOP1	YDR141C	Golgi-localized, leucine-zipper domain containing protein; involved in endosome to Golgi transport, organization of the ER, establishing cell polarity, and morphogenesis; detected in highly purified mitochondria in high-throughput studies	-2.84788	-1.50989	down
1770777_at	RPO26	YPR187W	RNA polymerase subunit ABC23, common to RNA polymerases I, II, and III; part of central core; similar to bacterial omega subunit	-2.85204	-1.51199	down
1777604_at	NTC20	YBR188C	Member of the NineTeen Complex (NTC) that contains Prp19p and stabilizes U6 snRNA in catalytic forms of the spliceosome containing U2, U5, and U6 snRNAs	-2.85606	-1.51403	down
1773430_at	ARH1	YDR376W	Oxidoreductase of the mitochondrial inner membrane, involved in cytoplasmic and mitochondrial iron homeostasis and required for activity of Fe-S cluster-containing enzymes; one of the few mitochondrial proteins essential for viability	-2.86077	-1.5164	down
1776909_at	UTP20	YBL004W	Component of the small-subunit (SSU) processome, which is involved in the biogenesis of the 18S rRNA	-2.86606	-1.51907	down
1773292_at	RPL26B	YGR034W	Protein component of the large (60S) ribosomal subunit, nearly identical to Rpl26Ap and has similarity to E. coli L24 and rat L26 ribosomal proteins; binds to 5.8S rRNA	-2.87083	-1.52147	down
1778145_at	RRM3	YHR031C	DNA helicase involved in rDNA replication and Ty1 transposition; relieves replication fork pauses at telomeric regions; structurally and functionally related to Pif1p	-2.87083	-1.52147	down
1779705_at	FMP41	YNL168C	Putative protein of unknown function; GFP-fusion protein is induced in response to the DNA-damaging agent MMS; the authentic, non-tagged protein is detected in highly purified mitochondria in high-throughput studies	-2.87151	-1.52181	down
1769549_at	FSF1	YOR271C	Putative protein, predicted to be an alpha-isopropylmalate carrier; belongs to the sideroblastic-associated protein family; non-tagged protein is detected in purified mitochondria; likely to play a role in iron homeostasis	-2.87217	-1.52214	down
1771814_at	HTD2	YHR067W	Mitochondrial 3-hydroxyacyl-thioester dehydratase involved in fatty acid biosynthesis, required for respiratory growth and for normal mitochondrial morphology	-2.87571	-1.52392	down
1770448_at	CDC21	YOR074C	Thymidylate synthase, required for de novo biosynthesis of pyrimidine deoxyribonucleotides; expression is induced at G1/S	-2.88224	-1.52719	down

1769785_at	EFB1	YAL003W	Translation elongation factor 1 beta; stimulates nucleotide exchange to regenerate EF-1 alpha-GTP for the next elongation cycle; part of the EF-1 complex, which facilitates binding of aminoacyl-tRNA to the ribosomal A site	-2.88754	-1.52984	down
1774719_at	CGR1	YGL029W	Protein involved in nucleolar integrity and processing of the pre-rRNA for the 60S ribosome subunit; transcript is induced in response to cytotoxic stress but not genotoxic stress	-2.89017	-1.53115	down
1773687_at	TYW1	YPL207W	Iron-sulfer protein required for synthesis of Wybutosine modified tRNA; Wybutosine is a modified guanosine found at the 3'-position adjacent to the anticodon of phenylalanine tRNA which supports reading frame maintenance by stabilizing codon-anticodon interactions; induction by Yap5p in response to iron provides protection from high iron toxicity; overexpression results in increased cellular iron	-2.89205	-1.53209	down
1778816_at	GDH1	YOR375C	NADP(+)-dependent glutamate dehydrogenase, synthesizes glutamate from ammonia and alpha-ketoglutarate; rate of alpha-ketoglutarate utilization differs from Gdh3p; expression regulated by nitrogen and carbon sources	-2.89798	-1.53505	down
1777369_at	SHO1	YER118C	Transmembrane osmosensor involved in activation of the Cdc42p- and MAP kinase-dependent filamentous growth pathway and the high-osmolarity glycerol response pathway; phosphorylated by Hog1p; interacts with Pbs2p, Msb2p, Hkr1p, and Ste11p	-2.90223	-1.53716	down
1776399_at	MGS1	YNL218W	Protein with DNA-dependent ATPase and ssDNA annealing activities involved in maintenance of genome; interacts functionally with DNA polymerase delta; homolog of human Werner helicase interacting protein (WHIP)	-2.90885	-1.54045	down
1769942_at	RPB11	YOL005C	RNA polymerase II subunit B12.5; part of central core; similar to Rpc19p and bacterial alpha subunit	-2.91392	-1.54296	down
1776686_at	ISM1	YPL040C	Mitochondrial isoleucyl-tRNA synthetase, null mutant is deficient in respiratory growth	-2.91592	-1.54395	down
1770339_at	TEF4	YKL081W	Gamma subunit of translational elongation factor eEF1B, stimulates the binding of aminoacyl-tRNA (AA-tRNA) to ribosomes by releasing eEF1A (Tef1p/Tef2p) from the ribosomal complex	-2.91921	-1.54558	down
1778003_at	NIS1	YNL078W	Protein localized in the bud neck at G2/M phase; physically interacts with septins; possibly involved in a mitotic signaling network	-2.91998	-1.54596	down
1769697_at	RER2	YBR002C	Cis-prenyltransferase involved in dolichol synthesis; participates in endoplasmic reticulum (ER) protein sorting	-2.92578	-1.54882	down
1775821_at	TAD2	YJL035C	Subunit of tRNA-specific adenosine-34 deaminase, forms a heterodimer with Tad3p that converts adenosine to inosine at the wobble position of several tRNAs	-2.93137	-1.55157	down
1771158_at		YLR342W-A		-2.93159	-1.55168	down
1772041_at	NPL3	YDR432W	RNA-binding protein that promotes elongation, regulates termination, and carries poly(A) mRNA from nucleus to cytoplasm; has a role in repressing translation initiation by binding eIF4G; required for pre-mRNA splicing; dissociation from mRNAs promoted by Mtr10p; phosphorylated by Sky1p in the cytoplasm	-2.93843	-1.55505	down
1775789_at		YDL144C		-2.94461	-1.55808	down
1774342_at	MYO2	YOR326W	One of two type V myosin motors (along with MYO4) involved in actin-based transport of cargos; required for the polarized delivery of secretory vesicles, the vacuole, late Golgi elements,	-2.94467	-1.55811	down

			peroxisomes, and the mitotic spindle			
1778651_at	FAA4	YMR246W	Long chain fatty acyl-CoA synthetase; activates imported fatty acids with a preference for C12:0-C16:0 chain lengths; functions in long chain fatty acid import; important for survival during stationary phase; localized to lipid particles; involved in sphingolipid-to-glycerolipid metabolism	-2.9458	-1.55866	down
1778279_at	TSC10	YBR265W	3-ketosphinganine reductase, catalyzes the second step in phytosphingosine synthesis, essential for growth in the absence of exogenous dihydrosphingosine or phytosphingosine, member of short chain dehydrogenase/reductase protein family	-2.95774	-1.5645	down
1771743_at	NUC1	YJL208C	Major mitochondrial nuclease, has RNAse and DNA endo- and exonucleolytic activities; has roles in mitochondrial recombination, apoptosis and maintenance of polyploidy	-2.96199	-1.56657	down
1776125_at	GIC1	YHR061C	Protein of unknown function involved in initiation of budding and cellular polarization, interacts with Cdc42p via the Cdc42/Rac-interactive binding (CRIB) domain	-2.96497	-1.56802	down
1769511_at	DBP6	YNR038W	Essential protein involved in ribosome biogenesis; putative ATP-dependent RNA helicase of the DEAD-box protein family	-2.96675	-1.56888	down
1773885_at		YDL085C-A		-2.97431	-1.57255	down
1779734_at	GUA1	YMR217W	GMP synthase; highly conserved enzyme that catalyzes the second step in the biosynthesis of GMP from inosine 5'-phosphate (IMP); transcription is not subject to regulation by guanine but is negatively regulated by nutrient starvation; reduction-of-function mutation gua1-G388D causes changes in cellular guanine nucleotide pools, defects in general protein synthesis, and impaired translation of GCN4 mRNA	-2.98149	-1.57603	down
1770848_at	DPH2	YKL191W	Protein required, along with Dph1p, Kti11p, Jjj3p, and Dph5p, for synthesis of diphthamide, which is a modified histidine residue of translation elongation factor 2 (Eft1p or Eft2p); may act in a complex with Dph1p and Kti11p	-2.98348	-1.577	down
1775295_at	ASP1	YDR321W	Cytosolic L-asparaginase, involved in asparagine catabolism; catalyzes hydrolysis of L-asparagine to aspartic acid and ammonia, has an important role in therapy of acute lymphoblastic leukemia; synthesized constitutively	-2.98618	-1.5783	down
1769377_at		YBR238C		-2.98637	-1.57839	down
1772065_at	RPL12B	YDR418W	Protein component of the large (60S) ribosomal subunit, nearly identical to Rpl12Ap; rpl12a rpl12b double mutant exhibits slow growth and slow translation; has similarity to E. coli L11 and rat L12 ribosomal proteins	-2.98888	-1.57961	down
1771440_at	ROK1	YGL171W	RNA-dependent ATPase; involved in pre-rRNA processing at sites A0, A1, and A2, and in control of cell cycle progression; contains two upstream open reading frames (uORFs) in 5' untranslated region which regulate translation	-2.98997	-1.58013	down
1779787_at	RPL21 A	YBR191W	Protein component of the large (60S) ribosomal subunit, nearly identical to Rpl21Bp and has similarity to rat L21 ribosomal protein	-2.99752	-1.58377	down
1777989_at	RPL31B	YLR406C	Protein component of the large (60S) ribosomal subunit, nearly identical to Rpl31Ap and has similarity to rat L31 ribosomal protein; associates with the karyopherin Sxm1p; loss of both Rpl31p and Rpl39p confers lethality	-2.99755	-1.58378	down

1778886_at	RPS15	YOL040C	Protein component of the small (40S) ribosomal subunit; has similarity to E. coli S19 and rat S15 ribosomal proteins	-3.00218	-1.58601	down
1770345_at	TIM23	YNR017W	Essential component of the Translocase of the Inner Mitochondrial membrane (TIM23 complex); involved in protein import into mitochondrial matrix and inner membrane; with Tim17p, contributes to architecture and function of the import channel	-3.01003	-1.58978	down
1771444_at	RPG1	YBR079C	eIF3a subunit of the core complex of translation initiation factor 3 (eIF3), essential for translation; part of a Prt1p-Rpg1p-Nip1p subcomplex that stimulates binding of mRNA and tRNA(i)Met to ribosomes; involved in translation reinitiation	-3.01552	-1.59241	down
1769366_at	DAS2	YDR020C	Putative protein of unknown function; non-essential gene identified in a screen for mutants with increased levels of rDNA transcription; weak similarity with uridine kinases and with phosphoribokinases	-3.02401	-1.59646	down
1775631_at	BLM10	YFL007W	Proteasome activator; binds the core proteasome and stimulates proteasome-mediated protein degradation by inducing gate opening; required for resistance to bleomycin, may be involved in protecting against oxidative damage; similar to mammalian PA200	-3.0274	-1.59808	down
1775023_at		YJR003C		-3.03483	-1.60162	down
1776792_at	TAE1	YBR261C	AdoMet-dependent proline methyltransferase; catalyzes the dimethylation of ribosomal proteins Rpl12 and Rps25 at N-terminal proline residues; has a role in protein synthesis; fusion protein localizes to the cytoplasm	-3.03846	-1.60334	down
1779965_at	DSE2	YHR143W	Daughter cell-specific secreted protein with similarity to glucanases, degrades cell wall from the daughter side causing daughter to separate from mother; expression is repressed by cAMP	-3.04735	-1.60756	down
1778486_at	SIM1	YIL123W	Protein of the SUN family (Sim1p, Uth1p, Nca3p, Sun4p) that may participate in DNA replication, promoter contains SCB regulation box at -300 bp indicating that expression may be cell cycle-regulated	-3.05036	-1.60898	down
1776523_at	DPS1	YLL018C	Aspartyl-tRNA synthetase, primarily cytoplasmic; homodimeric enzyme that catalyzes the specific aspartylation of tRNA(Asp); class II aminoacyl tRNA synthetase; binding to its own mRNA may confer autoregulation	-3.05161	-1.60957	down
1777555_at	RPL27 A	YHR010W	Protein component of the large (60S) ribosomal subunit, nearly identical to Rpl27Bp and has similarity to rat L27 ribosomal protein	-3.06889	-1.61772	down
1770284_at	RPL37 A	YLR185W	Protein component of the large (60S) ribosomal subunit, has similarity to Rpl37Bp and to rat L37 ribosomal protein	-3.07099	-1.61871	down
1773091_at	RPL2B	YIL018W	Protein component of the large (60S) ribosomal subunit, identical to Rpl2Ap and has similarity to E. coli L2 and rat L8 ribosomal proteins; expression is upregulated at low temperatures	-3.07586	-1.62099	down
1777301_at	KIC1	YHR102W	Protein kinase of the PAK/Ste20 family, required for cell integrity; physically interacts with Cdc31p (centrin), which is a component of the spindle pole body; part of the RAM network that regulates cellular polarity and morphogenesis	-3.08267	-1.62418	down
1771052_at	FMP45	YDL222C	Integral membrane protein localized to mitochondria (untagged protein); required for sporulation and maintaining sphingolipid content; has sequence similarity to SUR7 and YNL194C	-3.09094	-1.62805	down
1771788_at	ERP2	YAL007C	Protein that forms a heterotrimeric complex with Erp1p, Emp24p, and Erv25p; member, along with	-3.09346	-1.62922	down

			Emp24p and Erv25p, of the p24 family involved in ER to Golgi transport and localized to COPII-coated vesicles			
1770577_at	RPL11B	YGR085C	Protein component of the large (60S) ribosomal subunit, nearly identical to Rpl11Ap; involved in ribosomal assembly; depletion causes degradation of proteins and RNA of the 60S subunit; has similarity to E. coli L5 and rat L11	-3.09639	-1.63059	down
1778759_at	EGD2	YHR193C	Alpha subunit of the heteromeric nascent polypeptide-associated complex (NAC) involved in protein sorting and translocation, associated with cytoplasmic ribosomes	-3.09808	-1.63137	down
1776104_at	RPS28B	YLR264W	Protein component of the small (40S) ribosomal subunit; nearly identical to Rps28Ap and has similarity to rat S28 ribosomal protein	-3.10481	-1.6345	down
1773302_at	ESS1	YJR017C	Peptidylprolyl-cis/trans-isomerase (PPIase) specific for phosphorylated serine and threonine residues N-terminal to proline; regulates phosphorylation of the RNA polymerase II large subunit (Rpo21p) C-terminal domain	-3.11999	-1.64154	down
1776315_at	DFM1	YDR411C	Endoplasmic reticulum (ER) localized protein involved in ER-associated protein degradation (ERAD), ER stress and homeostasis; interacts with components of ERAD-L and ERAD-C and Cdc48p; derlin-like family member similar to Der1p	-3.12254	-1.64272	down
1778408_at	BAR1	YIL015W	Aspartyl protease secreted into the periplasmic space of mating type a cells, helps cells find mating partners, cleaves and inactivates alpha factor allowing cells to recover from alpha-factor-induced cell cycle arrest	-3.12468	-1.64371	down
1772945_at		YBR230W-A		-3.13069	-1.64648	down
1773992_at	TUB2	YFL037W	Beta-tubulin; associates with alpha-tubulin (Tub1p and Tub3p) to form tubulin dimer, which polymerizes to form microtubules	-3.13124	-1.64673	down
1778929_at	ADO1	YJR105W	Adenosine kinase, required for the utilization of S-adenosylmethionine (AdoMet); may be involved in recycling adenosine produced through the methyl cycle	-3.13708	-1.64942	down
1780138_at	LEU9	YOR108W	Alpha-isopropylmalate synthase II (2-isopropylmalate synthase), catalyzes the first step in the leucine biosynthesis pathway; the minor isozyme, responsible for the residual alpha-IPMS activity detected in a leu4 null mutant	-3.14643	-1.65371	down
1772875_at	RPL4B	YDR012W	Protein component of the large (60S) ribosomal subunit, nearly identical to Rpl4Ap and has similarity to E. coli L4 and rat L4 ribosomal proteins	-3.14652	-1.65376	down
1776498_at	UTP15	YMR093W	Nucleolar protein, component of the small subunit (SSU) processome containing the U3 snoRNA that is involved in processing of pre-18S rRNA	-3.15763	-1.65884	down
1771367_at	RPP1	YHR062C	Subunit of both RNase MRP and nuclear RNase P; RNase MRP cleaves pre-rRNA, while nuclear RNase P cleaves tRNA precursors to generate mature 5' ends and facilitates turnover of nuclear RNAs	-3.16175	-1.66072	down
1780004_at	ADE6	YGR061C	Formylglycinamidine-ribonucleotide (FGAM)-synthetase, catalyzes a step in the 'de novo' purine nucleotide biosynthetic pathway	-3.16548	-1.66242	down
1774488_at		YOL107W		-3.17434	-1.66646	down
1771911_at	RPL20A	YMR242C	Protein component of the large (60S) ribosomal subunit, nearly identical to Rpl20Bp and has similarity to rat L18a ribosomal protein	-3.17874	-1.66846	down
1773840_at	MDM20	YOL076W	Non-catalytic subunit of the NatB N-terminal acetyltransferase, which catalyzes N-acetylation of proteins with specific N-terminal sequences;	-3.18375	-1.67073	down

			involved in mitochondrial inheritance and actin assembly			
1779214_at	LHS1	YKL073W	Molecular chaperone of the endoplasmic reticulum lumen, involved in polypeptide translocation and folding; nucleotide exchange factor for the ER lumenal Hsp70 chaperone Kar2p; regulated by the unfolded protein response pathway	-3.18544	-1.67149	down
1770020_at	NCS6	YGL211W	Protein required for thiolation of the uridine at the wobble position of Gln, Lys, and Glu tRNAs; has a role in urmylation and in invasive and pseudohyphal growth; inhibits replication of Brome mosaic virus in S. cerevisiae	-3.18686	-1.67214	down
1769880_at	RKM5	YLR137W	Protein lysine methyltransferase; monomethylates Lys-46 of the ribosomal large subunit Rpl1a/Rpl1b; member of the seven beta-strand methyltransferase superfamily; orthologs only found among fungal species	-3.18719	-1.67228	down
1769834_at	HST3	YOR025W	Member of the Sir2 family of NAD(+)-dependent protein deacetylases; involved along with Hst4p in telomeric silencing, cell cycle progression, radiation resistance, genomic stability and short-chain fatty acid metabolism	-3.18795	-1.67263	down
1779152_at	SDA1	YGR245C	Highly conserved nuclear protein required for actin cytoskeleton organization and passage through Start, plays a critical role in G1 events, binds Nap1p, also involved in 60S ribosome biogenesis	-3.19093	-1.67398	down
1772310_at	HRD3	YLR207W	Resident protein of the ER membrane that plays a central role in ER-associated protein degradation (ERAD), forms HRD complex with Hrd1p and ERAD determinants that engages in lumen to cytosol communication and coordination of ERAD events	-3.20102	-1.67853	down
1778044_at	YCH1	YGR203W	Phosphatase with sequence similarity to Cdc25p, Arr2p and Mih1p; member of the single-domain rhodanese homology superfamily; green fluorescent protein (GFP)-fusion protein localizes to both the cytoplasm and the nucleus	-3.20721	-1.68132	down
1776027_at	CDC60	YPL160W	Cytosolic leucyl tRNA synthetase, ligates leucine to the appropriate tRNA	-3.21237	-1.68364	down
1770485_at	RPA34	YJL148W	RNA polymerase I subunit A34.5	-3.21487	-1.68476	down
1770882_at	RPL13B	YMR142C	Protein component of the large (60S) ribosomal subunit, nearly identical to Rpl13Ap; not essential for viability; has similarity to rat L13 ribosomal protein	-3.22392	-1.68881	down
1774856_at	YFH1	YDL120W	Mitochondrial matrix iron chaperone; oxidizes and stores iron; interacts with Isu1p to promote Fe-S cluster assembly; mutation results in multiple Fe/S-dependent enzyme deficiencies; human frataxin homolog is mutated in Friedrich's ataxia	-3.22833	-1.69079	down
1778194_at	CHS2	YBR038W	Chitin synthase II; catalyzes transfer of N-acetylglucosamine (GlcNAc) to chitin upon activation of zymogenic form; required for chitin synthesis in the primary septum during cytokinesis; localization regulated by Cdk1p during mitosis	-3.22878	-1.69099	down
1769988_at	DSE4	YNR067C	Daughter cell-specific secreted protein with similarity to glucanases, degrades cell wall from the daughter side causing daughter to separate from mother	-3.24229	-1.69701	down
1771832_at	RPL12 A	YEL054C	Protein component of the large (60S) ribosomal subunit, nearly identical to Rpl12Bp; rpl12a rpl12b double mutant exhibits slow growth and slow translation; has similarity to E. coli L11 and rat L12 ribosomal proteins	-3.24243	-1.69707	down
1777358_at	TIF5	YPR041W	Translation initiation factor eIF5; functions both as a GTPase-activating protein to mediate hydrolysis of ribosome-bound GTP and as a GDP dissociation	-3.24589	-1.69862	down

			inhibitor to prevent recycling of eIF2			
1770861_at	UTP10	YJL109C	Nucleolar protein, component of the small subunit (SSU) processome containing the U3 snoRNA that is involved in processing of pre-18S rRNA; mutant has increased aneuploidy tolerance	-3.25377	-1.70211	down
1775452_at	ABP140	YOR239W	AdoMet-dependent tRNA methyltransferase and actin binding protein; C-terminal domain is responsible for 3-methylcytidine modification of residue 32 of the tRNA anticodon loop of tRNA-Thr and tRNA-Ser and contains an S-adenosylmethionine (AdoMet) binding motif; N-terminal actin binding sequence interacts with actin filaments and localizes to actin patches and cables; N- and C-terminal domains are encoded in separate ORFs that are translated into one protein via a +1 frameshift	-3.25828	-1.70411	down
1773053_at	OXA1	YER154W	Mitochondrial inner membrane insertase, mediates the insertion of both mitochondrial- and nuclear-encoded proteins from the matrix into the inner membrane, interacts with mitochondrial ribosomes; conserved from bacteria to animals	-3.26644	-1.70772	down
1779073_at	UTP18	YJL069C	Possible U3 snoRNP protein involved in maturation of pre-18S rRNA, based on computational analysis of large-scale protein-protein interaction data	-3.26729	-1.7081	down
1774495_at	GAD1	YMR250W	Glutamate decarboxylase, converts glutamate into gamma-aminobutyric acid (GABA) during glutamate catabolism; involved in response to oxidative stress	-3.28108	-1.71417	down
1778202_at	RRP6	YOR001W	Nuclear exosome exonuclease component; has 3'-5' exonuclease activity; involved in RNA processing, maturation, surveillance, degradation, tethering, and export; has similarity to E. coli RNase D and to human PM-Sc1 100 (EXOSC10); mutant displays reduced transcription elongation in the G-less-based run-on (GLRO) assay	-3.29311	-1.71945	down
1774757_at	RRP12	YPL012W	Protein required for export of the ribosomal subunits; associates with the RNA components of the pre-ribosomes; has a role in nuclear import in association with Pse1p; contains HEAT-repeats	-3.3078	-1.72587	down
1778739_at	SFG1	YOR315W	Nuclear protein, putative transcription factor required for growth of superficial pseudohyphae (which do not invade the agar substrate) but not for invasive pseudohyphal growth; may act together with Phd1p; potential Cdc28p substrate	-3.30964	-1.72667	down
1775759_at	KTR3	YBR205W	Putative alpha-1,2-mannosyltransferase involved in O- and N-linked protein glycosylation; member of the KRE2/MNT1 mannosyltransferase family; Svp26p mediates uptake of Ktr3p into COPII vesicles	-3.31896	-1.73073	down
1773254_at	STE6	YKL209C	Plasma membrane ATP-binding cassette (ABC) transporter required for the export of a-factor, catalyzes ATP hydrolysis coupled to a-factor transport; contains 12 transmembrane domains and two ATP binding domains; expressed only in MATa cells	-3.32323	-1.73259	down
1777990_at	RPS10A	YOR293W	Protein component of the small (40S) ribosomal subunit; nearly identical to Rps10Bp and has similarity to rat ribosomal protein S10	-3.32875	-1.73498	down
1776853_at	RPS10B	YMR230W	Protein component of the small (40S) ribosomal subunit; nearly identical to Rps10Ap and has similarity to rat ribosomal protein S10	-3.32896	-1.73507	down
1778412_at	PRO3	YER023W	Delta 1-pyrroline-5-carboxylate reductase, catalyzes the last step in proline biosynthesis	-3.33331	-1.73695	down
1773475_at	TCB1	YOR086C	Lipid-binding ER protein, enriched at ER-plasma membrane contact sites; contains three calcium and lipid binding domains; non-tagged protein also	-3.33736	-1.73871	down

			localizes to mitochondria; C-termini of Tcb1p, Tcb2p and Tcb3p interact			
1773125_at		YGR283C		-3.34285	-1.74108	down
1779105_at	GSY2	YLR258W	Glycogen synthase, similar to Gsy1p; expression induced by glucose limitation, nitrogen starvation, heat shock, and stationary phase; activity regulated by cAMP-dependent, Snf1p and Pho85p kinases as well as by the Gac1p-Glc7p phosphatase	-3.35159	-1.74484	down
1777387_at	SFB3	YHR098C	Component of the Sec23p-Sfb3p heterodimer of the COPII vesicle coat, required for cargo selection during vesicle formation in ER to Golgi transport; homologous to Sec24p and Sfb2p	-3.3555	-1.74653	down
1776091_at	RPA43	YOR340C	RNA polymerase I subunit A43	-3.35688	-1.74712	down
1772882_at	ACC1	YNR016C	Acetyl-CoA carboxylase, biotin containing enzyme that catalyzes the carboxylation of acetyl-CoA to form malonyl-CoA; required for de novo biosynthesis of long-chain fatty acids	-3.36086	-1.74883	down
1769829_at	RPS18A	YDR450W	Protein component of the small (40S) ribosomal subunit; nearly identical to Rps18Bp and has similarity to E. coli S13 and rat S18 ribosomal proteins	-3.3612	-1.74898	down
1776884_at		YPR063C		-3.36496	-1.75059	down
1779426_at	SEC26	YDR238C	Essential beta-coat protein of the COPI coatomer, involved in ER-to-Golgi protein trafficking and maintenance of normal ER morphology; shares 43% sequence identity with mammalian beta-coat protein (beta-COP)	-3.36608	-1.75107	down
1779254_at	BUD23	YCR047C	Methyltransferase, methylates residue G1575 of 18S rRNA; required for rRNA processing and nuclear export of 40S ribosomal subunits independently of methylation activity; diploid mutant displays random budding pattern	-3.36811	-1.75194	down
1771510_at	PSD2	YGR170W	Phosphatidylserine decarboxylase of the Golgi and vacuolar membranes, converts phosphatidylserine to phosphatidylethanolamine	-3.37435	-1.75461	down
1776370_at	RPL2A	YFR031C-A	Protein component of the large (60S) ribosomal subunit, identical to Rpl2Bp and has similarity to E. coli L2 and rat L8 ribosomal proteins	-3.38582	-1.7595	down
1769388_at	DUS3	YLR401C	Dihydrouridine synthase, member of a widespread family of conserved proteins including Smm1p, Dus1p, and Dus4p; contains a consensus oleate response element (ORE) in its promoter region	-3.38679	-1.75992	down
1776080_at	GUS1	YGL245W	Glutamyl-tRNA synthetase (GluRS), forms a complex with methionyl-tRNA synthetase (Mes1p) and Arc1p; complex formation increases the catalytic efficiency of both tRNA synthetases and ensures their correct localization to the cytoplasm	-3.38754	-1.76024	down
1777928_at	FAR1	YJL157C	Cyclin-dependent kinase inhibitor that mediates cell cycle arrest in response to pheromone; also forms a complex with Cdc24p, Ste4p, and Ste18p that may specify the direction of polarized growth during mating; potential Cdc28p substrate	-3.38761	-1.76027	down
1778332_at	GNT1	YOR320C	N-acetylglucosaminyltransferase capable of modification of N-linked glycans in the Golgi apparatus	-3.39722	-1.76436	down
1771975_at	BUB2	YMR055C	Mitotic exit network regulator, forms GTPase-activating Bfa1p-Bub2p complex that binds Tem1p and spindle pole bodies, blocks cell cycle progression before anaphase in response to spindle and kinetochore damage	-3.39761	-1.76452	down
1779175_at	TMA46	YOR091W	Protein of unknown function that associates with translating ribosomes; interacts with GTPase Rbg1p	-3.40201	-1.76639	down
1772416_at	MNT2	YGL257C	Mannosyltransferase involved in adding the 4th and 5th mannose residues of O-linked glycans	-3.40776	-1.76882	down

1774947_at	TOS4	YLR183C	Forkhead Associated domain containing protein and putative transcription factor found associated with chromatin; target of SBF transcription factor; expression is periodic and peaks in G1; similar to PLM2	-3.41153	-1.77042	down
1779751_s_at		YBR219C		-3.41171	-1.7705	down
1777971_at	PGM1	YKL127W	Phosphoglucomutase, minor isoform; catalyzes the conversion from glucose-1-phosphate to glucose-6-phosphate, which is a key step in hexose metabolism	-3.4131	-1.77108	down
1770043_at	NMD3	YHR170W	Protein involved in nuclear export of the large ribosomal subunit; acts as a Crm1p-dependent adapter protein for export of nascent ribosomal subunits through the nuclear pore complex	-3.41799	-1.77315	down
1773378_at	HPA3	YEL066W	D-Amino acid N-acetyltransferase, catalyzes N-acetylation of D-amino acids through ordered bi-bi mechanism in which acetyl-CoA is first substrate bound and CoA is last product liberated; similar to Hpa2p, acetylates histones weakly in vitro	-3.42211	-1.77489	down
1774186_at	SSF1	YHR066W	Constituent of 66S pre-ribosomal particles, required for ribosomal large subunit maturation; functionally redundant with Ssf2p; member of the Brix family	-3.43014	-1.77827	down
1769805_at	ALD3	YMR169C	Cytoplasmic aldehyde dehydrogenase, involved in beta-alanine synthesis; uses NAD+ as the preferred coenzyme; very similar to Ald2p; expression is induced by stress and repressed by glucose	-3.43326	-1.77958	down
1771817_at	PSD1	YNL169C	Phosphatidylserine decarboxylase of the mitochondrial inner membrane, converts phosphatidylserine to phosphatidylethanolamine	-3.44031	-1.78254	down
1777639_at	PPT1	YGR123C	Protein serine/threonine phosphatase, regulates Hsp90 chaperone by affecting its ATPase and cochaperone binding activities; has similarity to human phosphatase PP5; present in both the nucleus and cytoplasm; expressed during logarithmic growth	-3.44077	-1.78273	down
1778331_at		YDL211C		-3.44247	-1.78344	down
1776524_at		YBL028C		-3.44409	-1.78412	down
1778167_at	SEC62	YPL094C	Essential subunit of Sec63 complex (Sec63p, Sec62p, Sec66p and Sec72p); with Sec61 complex, Kar2p/BiP and Lhs1p forms a channel competent for SRP-dependent and post-translational SRP-independent protein targeting and import into the ER	-3.44495	-1.78448	down
1777942_at		YBR220C		-3.44564	-1.78477	down
1773056_at		YOL019W		-3.45673	-1.78941	down
1771580_at	KDX1	YKL161C	Protein kinase implicated in the Slt2p mitogen-activated (MAP) kinase signaling pathway; interacts with numerous components in the mating pheromone and CWI MAPK pathways; associates with Rlm1p	-3.45993	-1.79074	down
1777093_at	TRS130	YMR218C	One of 10 subunits of the transport protein particle (TRAPP) complex of the cis-Golgi which mediates vesicle docking and fusion; involved in ER to Golgi membrane traffic; mutation activates transcription of OCH1	-3.46108	-1.79122	down
1772407_at	YIP3	YNL044W	Protein localized to COPII vesicles, proposed to be involved in ER to Golgi transport; interacts with members of the Rab GTPase family and Yip1p; also interacts with Rtn1p	-3.46209	-1.79165	down
1778038_at	RLI1	YDR091C	Essential iron-sulfur protein required for ribosome biogenesis and translation initiation and termination; facilitates binding of a multifactor complex (MFC) of initiation factors to the small ribosomal subunit; predicted ABC family ATPase	-3.46314	-1.79208	down

1774541_at	PWP1	YLR196W	Protein with WD-40 repeats involved in rRNA processing; associates with trans-acting ribosome biogenesis factors; similar to beta-transducin superfamily	-3.46682	-1.79361	down
1778611_at	ZUO1	YGR285C	Ribosome-associated chaperone, functions in ribosome biogenesis and, in partnership with Ssz1p and SSb1/2, as a chaperone for nascent polypeptide chains; contains a DnaJ domain and functions as a J-protein partner for Ssb1p and Ssb2p	-3.46885	-1.79446	down
1774942_at		YBR200W-A		-3.47757	-1.79808	down
1779373_at	POC4	YPL144W	Component of a heterodimeric Poc4p-Irc25p chaperone involved in assembly of alpha subunits into the 20S proteasome; may regulate formation of proteasome isoforms with alternative subunits under different conditions	-3.48269	-1.8002	down
1778192_at	SEC7	YDR170C	Guanine nucleotide exchange factor (GEF) for ADP ribosylation factors involved in proliferation of the Golgi, intra-Golgi transport and ER-to-Golgi transport; found in the cytoplasm and on Golgi-associated coated vesicles	-3.50227	-1.80829	down
1778731_at	RPL8B	YLL045C	Ribosomal protein L4 of the large (60S) ribosomal subunit, nearly identical to Rpl8Ap and has similarity to rat L7a ribosomal protein; mutation results in decreased amounts of free 60S subunits	-3.50466	-1.80927	down
1780102_at	ERC1	YHR032W	Member of the multi-drug and toxin extrusion (MATE) family of the multidrug/oligosaccharidyl-lipid/polysaccharide (MOP) exporter superfamily; overproduction confers ethionine resistance and accumulation of S-adenosylmethionine	-3.50571	-1.80971	down
1778347_at	NOP15	YNL110C	Constituent of 66S pre-ribosomal particles, involved in 60S ribosomal subunit biogenesis; localizes to both nucleolus and cytoplasm	-3.50851	-1.81086	down
1776290_at	AIM20	YIL158W	Putative protein of unknown function; overexpression causes a cell cycle delay or arrest; green fluorescent protein (GFP)-fusion protein localizes to the vacuole; null mutant displays elevated frequency of mitochondrial genome loss	-3.51651	-1.81414	down
1771320_at	NUG1	YER006W	GTPase that associates with nuclear 60S pre-ribosomes, required for export of 60S ribosomal subunits from the nucleus	-3.52033	-1.81571	down
1778487_at	RPA12	YJR063W	RNA polymerase I subunit A12.2; contains two zinc binding domains, and the N terminal domain is responsible for anchoring to the RNA pol I complex	-3.52141	-1.81615	down
1770502_x_at	RPS26A	YGL189C	Protein component of the small (40S) ribosomal subunit; nearly identical to Rps26Bp and has similarity to rat S26 ribosomal protein	-3.5282	-1.81893	down
1771884_at	PUS2	YGL063W	Mitochondrial tRNA:pseudouridine synthase; acts at positions 27 and 28, but not at position 72; efficiently and rapidly targeted to mitochondria, specifically dedicated to mitochondrial tRNA modification	-3.53013	-1.81972	down
1779399_at	SAM3	YPL274W	High-affinity S-adenosylmethionine permease, required for utilization of S-adenosylmethionine as a sulfur source; has similarity to S-methylmethionine permease Mmp1p	-3.53146	-1.82026	down
1776992_at	AGA2	YGL032C	Adhesion subunit of a-agglutinin of a-cells, C-terminal sequence acts as a ligand for alpha-agglutinin (Sag1p) during agglutination, modified with O-linked oligomannosyl chains, linked to anchorage subunit Aga1p via two disulfide bonds	-3.53266	-1.82075	down
1770889_at	VPS13	YLL040C	Protein of unknown function; heterooligomeric or homooligomeric complex; peripherally associated with membranes; involved in sporulation, vacuolar protein sorting, prospore membrane formation and protein-Golgi retention; homologous to human	-3.5358	-1.82204	down

			CHAC and COH1 which are involved in chorea acanthocytosis and Cohen syndrome, respectively			
1778381_at	DUS4	YLR405W	Dihydrouridine synthase, member of a widespread family of conserved proteins including Smm1p, Dus1p, and Dus3p	-3.53745	-1.82271	down
1769434_at	RMT2	YDR465C	Arginine N5 methyltransferase; methylates ribosomal protein Rpl12 (L12) on Arg67	-3.53836	-1.82308	down
1779169_at	MAK16	YAL025C	Essential nuclear protein, constituent of 66S pre-ribosomal particles; required for maturation of 25S and 5.8S rRNAs; required for maintenance of M1 satellite double-stranded RNA of the L-A virus	-3.53893	-1.82331	down
1774927_at	MTR3	YGR158C	Exosome non-catalytic core component; involved in 3'-5' RNA processing and degradation in both the nucleus and the cytoplasm; has similarity to E. coli RNase PH and to human hMtr3p (EXOSC6)	-3.54173	-1.82446	down
1773157_at	STE2	YFL026W	Receptor for alpha-factor pheromone; seven transmembrane-domain GPCR that interacts with both pheromone and a heterotrimeric G protein to initiate the signaling response that leads to mating between haploid a and alpha cells	-3.55776	-1.83097	down
1774367_at	NUP192	YJL039C	Essential subunit of the inner ring of the nuclear pore complex (NPC); contributes to nucleocytoplasmic transport; homologous to human NUP205	-3.56024	-1.83198	down
1771500_at	CTF18	YMR078C	Subunit of a complex with Ctf8p that shares some subunits with Replication Factor C and is required for sister chromatid cohesion; may have overlapping functions with Rad24p in the DNA damage replication checkpoint	-3.56029	-1.83199	down
1771190_at	ATF2	YGR177C	Alcohol acetyltransferase, may play a role in steroid detoxification; forms volatile esters during fermentation, which is important for brewing and winemaking	-3.5712	-1.83641	down
1772805_at	UTP22	YGR090W	Possible U3 snoRNP protein involved in maturation of pre-18S rRNA, based on computational analysis of large-scale protein-protein interaction data	-3.57288	-1.83709	down
1780244_at	RBG2	YGR173W	Protein with a role in translation; forms a complex with Gir2p; has similarity to mammalian developmentally regulated GTP-binding protein	-3.57702	-1.83876	down
1772294_at	RPS29A	YLR388W	Protein component of the small (40S) ribosomal subunit; nearly identical to Rps29Bp and has similarity to rat S29 and E. coli S14 ribosomal proteins	-3.58301	-1.84117	down
1779888_at	PRY2	YKR013W	Protein of unknown function	-3.59732	-1.84692	down
1772646_at	GTT3	YEL017W	Protein of unknown function with a possible role in glutathione metabolism, as suggested by computational analysis of large-scale protein-protein interaction data; GFP-fusion protein localizes to the nuclear periphery	-3.61984	-1.85593	down
1770706_at	ERV46	YAL042W	Protein localized to COPII-coated vesicles, forms a complex with Erv41p; involved in the membrane fusion stage of transport	-3.64075	-1.86424	down
1774064_at		YDL121C		-3.64188	-1.86468	down
1769932_at	BTT1	YDR252W	Beta3 subunit of the heterotrimeric nascent polypeptide-associated complex which binds ribosomes via its beta-subunits in close proximity to nascent polypeptides; interacts with Caf130p of the CCR4-NOT complex; similar to human BTF3	-3.64593	-1.86629	down
1770258_at	RPL17 A	YKL180W	Protein component of the large (60S) ribosomal subunit, nearly identical to Rpl17Bp and has similarity to E. coli L22 and rat L17 ribosomal proteins; copurifies with the Dam1 complex (aka DASH complex)	-3.65065	-1.86815	down
1775454_at	LHP1	YDL051W	RNA binding protein required for maturation of tRNA and U6 snRNA precursors; acts as a	-3.65194	-1.86866	down

			molecular chaperone for RNAs transcribed by polymerase III; homologous to human La (SS-B) autoantigen			
1770257_at	EMW1	YNL313C	Essential conserved protein with a role in maintaining cell wall integrity; contains six TPR (tetratricopeptide repeat) domains clustered in the C-terminal region; conditional mutant is suppressed by overexpression of GFA1	-3.65246	-1.86887	down
1778655_at	CKI1	YLR133W	Choline kinase, catalyzing the first step in phosphatidylcholine synthesis via the CDP-choline (Kennedy pathway); exhibits some ethanolamine kinase activity contributing to phosphatidylethanolamine synthesis via the CDP-ethanolamine pathway	-3.65916	-1.87151	down
1772264_at	RLP24	YLR009W	Essential protein with similarity to Rpl24Ap and Rpl24Bp, associated with pre-60S ribosomal subunits and required for ribosomal large subunit biogenesis	-3.67124	-1.87627	down
1772172_at	MPP10	YJR002W	Component of the SSU processome and 90S preribosome, required for pre-18S rRNA processing, interacts with and controls the stability of Imp3p and Imp4p, essential for viability; similar to human Mpp10p	-3.67338	-1.87711	down
1770990_at	VHR2	YER064C	Non-essential nuclear protein; null mutation has global effects on transcription	-3.67718	-1.8786	down
1779256_at	FUI1	YBL042C	High affinity uridine permease, localizes to the plasma membrane; also mediates low but significant transport of the cytotoxic nucleoside analog 5-fluorouridine; not involved in uracil transport	-3.67827	-1.87903	down
1769765_at	RPL19 A	YBR084C-A	Protein component of the large (60S) ribosomal subunit, nearly identical to Rpl19Bp and has similarity to rat L19 ribosomal protein; rpl19a and rpl19b single null mutations result in slow growth, while the double null mutation is lethal	-3.68174	-1.88039	down
1774509_at	SCJ1	YMR214W	One of several homologs of bacterial chaperone DnaJ, located in the ER lumen where it cooperates with Kar2p to mediate maturation of proteins	-3.69069	-1.88389	down
1779808_at	NAN1	YPL126W	U3 snoRNP protein, component of the small (ribosomal) subunit (SSU) processosome containing U3 snoRNA; required for the biogenesis of18S rRNA	-3.70023	-1.88762	down
1777477_at	FEN1	YCR034W	Fatty acid elongase, involved in sphingolipid biosynthesis; acts on fatty acids of up to 24 carbons in length; mutations have regulatory effects on 1,3-beta-glucan synthase, vacuolar ATPase, and the secretory pathway	-3.7149	-1.89332	down
1777528_at	RPL11 A	YPR102C	Protein of the large 60S ribosomal subunit, nearly identical to Rpl11Bp but expressed at twice the level; involved in ribosomal assembly; depletion causes degradation of 60S proteins and RNA; similar to E. coli L5 and rat L11	-3.71504	-1.89338	down
1775002_at	LOT5	YKL183W	Protein of unknown function; gene expression increases in cultures shifted to a lower temperature	-3.7169	-1.8941	down
1775611_at	NMD5	YJR132W	Karyopherin, a carrier protein involved in nuclear import of proteins; importin beta homolog	-3.71953	-1.89512	down
1772855_at	MPA43	YNL249C	Putative protein of unknown function; the authentic, non-tagged protein is detected in highly purified mitochondria in high-throughput studies	-3.72487	-1.89719	down
1775480_at	CAR2	YLR438W	L-ornithine transaminase (OTAse), catalyzes the second step of arginine degradation, expression is dually-regulated by allophanate induction and a specific arginine induction process; not nitrogen catabolite repression sensitive	-3.74215	-1.90387	down
1777944_at	RPC31	YNL151C	RNA polymerase III subunit C31	-3.75573	-1.90909	down

1776021_at	HTZ1	YOL012C	Histone variant H2AZ, exchanged for histone H2A in nucleosomes by the SWR1 complex; involved in transcriptional regulation through prevention of the spread of silent heterochromatin	-3.75647	-1.90938	down
1775046_at	FAS2	YPL231W	Alpha subunit of fatty acid synthetase, which catalyzes the synthesis of long-chain saturated fatty acids; contains the acyl-carrier protein domain and beta-ketoacyl reductase, beta-ketoacyl synthase and self-pantetheinylation activities	-3.76103	-1.91113	down
1771542_at	RPC19	YNL113W	RNA polymerase subunit AC19, common to RNA polymerases I and III	-3.76126	-1.91122	down
1776913_at		YOL057W		-3.7844	-1.92006	down
1777632_at	DIP2	YLR129W	Nucleolar protein, specifically associated with the U3 snoRNA, part of the large ribonucleoprotein complex known as the small subunit (SSU) processome, required for 18S rRNA biogenesis, part of the active pre-rRNA processing complex	-3.78737	-1.92119	down
1773855_at	USB1	YLR132C	Essential protein that localizes to the nucleus and mitochondria; overexpression suppresses the respiratory defects of oxa1 and mtf2 mutants	-3.7921	-1.923	down
1769550_at	RIX1	YHR197W	Essential component of the Rix1 complex (Rix1p, Ipi1p, Ipi3p) that is required for processing of ITS2 sequences from 35S pre-rRNA; Rix1 complex associates with Mdn1p in pre-60S ribosomal particles	-3.80014	-1.92605	down
1772468_at	RPL40B	YKR094C	Fusion protein, identical to Rpl40Ap, that is cleaved to yield ubiquitin and a ribosomal protein of the large (60S) ribosomal subunit with similarity to rat L40; ubiquitin may facilitate assembly of the ribosomal protein into ribosomes	-3.80073	-1.92628	down
1778073_at	TRM10	YOL093W	tRNA methyltransferase, methylates the N-1 position of guanosine in tRNAs	-3.80664	-1.92852	down
1777403_at	IMP3	YHR148W	Component of the SSU processome, which is required for pre-18S rRNA processing, essential protein that interacts with Mpp10p and mediates interactions of Imp4p and Mpp10p with U3 snoRNA	-3.80875	-1.92932	down
1772333_at	KEL3	YPL263C	Cytoplasmic protein of unknown function	-3.8395	-1.94092	down
1773564_at	RBG1	YAL036C	Member of the DRG family of GTP-binding proteins; associates with translating ribosomes; interacts with Tma46p, Ygr250cp, Gir2p and Yap1p via two-hybrid	-3.84027	-1.94121	down
1770413_at	RPL24 A	YGL031C	Ribosomal protein L30 of the large (60S) ribosomal subunit, nearly identical to Rpl24Bp and has similarity to rat L24 ribosomal protein; not essential for translation but may be required for normal translation rate	-3.8409	-1.94144	down
1774349_at	MRT4	YKL009W	Protein involved in mRNA turnover and ribosome assembly, localizes to the nucleolus	-3.84957	-1.9447	down
1776241_at	ERV2	YPR037C	Flavin-linked sulfhydryl oxidase localized to the endoplasmic reticulum lumen, involved in disulfide bond formation within the ER	-3.85478	-1.94665	down
1769741_at	VBA4	YDR119W	Protein of unknown function with proposed role as a basic amino acid permease based on phylogeny; GFP-fusion protein localizes to vacuolar membrane; physical interaction with Atg27p suggests a possible role in autophagy; non-essential gene	-3.85579	-1.94702	down
1773605_at	NOP13	YNL175C	Nucleolar protein found in preribosomal complexes; contains an RNA recognition motif (RRM)	-3.85806	-1.94788	down
1775720_at	YOX1	YML027W	Homeodomain-containing transcriptional repressor, binds to Mcm1p and to early cell cycle boxes (ECBs) in the promoters of cell cycle-regulated genes expressed in M/G1 phase; expression is cell	-3.8601	-1.94864	down

			cycle-regulated; potential Cdc28p substrate			
1772003_at	LYS9	YNR050C	Saccharopine dehydrogenase (NADP+, L-glutamate-forming); catalyzes the formation of saccharopine from alpha-aminoadipate 6-semialdehyde, the seventh step in lysine biosynthesis pathway; exhibits genetic and physical interactions with TRM112	-3.87174	-1.95298	down
1771795_at	COP1	YDL145C	Alpha subunit of COPI vesicle coatomer complex, which surrounds transport vesicles in the early secretory pathway	-3.87526	-1.95429	down
1778245_at	MOD5	YOR274W	Delta 2-isopentenyl pyrophosphate:tRNA isopentenyl transferase; required for biosynthesis of the modified base isopentenyladenosine in mitochondrial and cytoplasmic tRNAs; gene is nuclear and encodes two isozymic forms; converts to a prion form, and prion conversion contributes to azole antifungal resistance by upregulating ergosterol biosynthesis	-3.87881	-1.95561	down
1769406_at	MKC7	YDR144C	GPI-anchored aspartyl protease, member of the yapsin family of proteases involved in cell wall growth and maintenance; shares functions with Yap3p and Kex2p	-3.88274	-1.95707	down
1773099_at	KRE33	YNL132W	Essential protein, required for biogenesis of the small ribosomal subunit; heterozygous mutant shows haploinsufficiency in K1 killer toxin resistance	-3.88291	-1.95714	down
1776525_at	KIN3	YAR018C	Nonessential serine/threonine protein kinase; possible role in DNA damage response	-3.88394	-1.95752	down
1779445_at	YCF1	YDR135C	Vacuolar glutathione S-conjugate transporter; member of the ATP-binding cassette family; has a role in detoxifying metals such as cadmium, mercury, and arsenite; also transports unconjugated bilirubin, selenodigluthatione, and oxidized glutathione; similar to human cystic fibrosis protein CFTR	-3.89616	-1.96205	down
1770157_at	BCP1	YDR361C	Essential protein involved in nuclear export of Mss4p, which is a lipid kinase that generates phosphatidylinositol 4,5-biphosphate and plays a role in actin cytoskeleton organization and vesicular transport	-3.90953	-1.967	down
1778120_at	MEU1	YLR017W	Methylthioadenosine phosphorylase (MTAP), catalyzes the initial step in the methionine salvage pathway; affects polyamine biosynthesis through regulation of ornithine decarboxylase (Spe1p) activity; regulates ADH2 gene expression	-3.91196	-1.96789	down
1777242_at	FRS1	YLR060W	Beta subunit of cytoplasmic phenylalanyl-tRNA synthetase, forms a tetramer with Frs2p to generate active enzyme; able to hydrolyze mis-aminoacylated tRNA-Phe, which could contribute to translational quality control	-3.91666	-1.96962	down
1771468_at	PKR1	YMR123W	V-ATPase assembly factor, functions with other V-ATPase assembly factors in the ER to efficiently assemble the V-ATPase membrane sector (V0)	-3.92087	-1.97117	down
1771892_at	FPR2	YDR519W	Membrane-bound peptidyl-prolyl cis-trans isomerase (PPIase), binds to the drugs FK506 and rapamycin; expression pattern suggests possible involvement in ER protein trafficking	-3.9249	-1.97266	down
1779257_at		YOR342C		-3.92596	-1.97305	down
1770500_at	RPL19B	YBL027W	Protein component of the large (60S) ribosomal subunit, nearly identical to Rpl19Ap and has similarity to rat L19 ribosomal protein; rpl19a and rpl19b single null mutations result in slow growth, while the double null mutation is lethal	-3.93032	-1.97465	down
1778099_at	SSZ1	YHR064C	Hsp70 protein that interacts with Zuo1p (a DnaJ homolog) to form a ribosome-associated complex	-3.94515	-1.98008	down

			that binds the ribosome via the Zuo1p subunit; also involved in pleiotropic drug resistance via sequential activation of PDR1 and PDR5; binds ATP			
1775457_at	RPL21B	YPL079W	Protein component of the large (60S) ribosomal subunit, nearly identical to Rpl21Ap and has similarity to rat L21 ribosomal protein	-3.95992	-1.98547	down
1771906_at	RPS18B	YML026C	Protein component of the small (40S) ribosomal subunit; nearly identical to Rps18Ap and has similarity to E. coli S13 and rat S18 ribosomal proteins	-3.96278	-1.98651	down
1772853_at	FET4	YMR319C	Low-affinity Fe(II) transporter of the plasma membrane	-3.97364	-1.99046	down
1779541_at	GFD2	YCL036W	Protein of unknown function, identified as a high-copy suppressor of a dbp5 mutation	-3.98852	-1.99585	down
1773444_at	GSY1	YFR015C	Glycogen synthase with similarity to Gsy2p, the more highly expressed yeast homolog; expression induced by glucose limitation, nitrogen starvation, environmental stress, and entry into stationary phase	-3.99098	-1.99674	down
1777215_at		YGR054W		-4.01221	-2.0044	down
1770383_at	SPE3	YPR069C	Spermidine synthase, involved in biosynthesis of spermidine and also in biosynthesis of pantothenic acid; spermidine is required for growth of wild-type cells	-4.01455	-2.00524	down
1773741_at	SEE1	YIL064W	Probable lysine methyltransferase involved in the dimethylation of eEF1A (Tef1p/Tef2p); sequence similarity to S-adenosylmethionine-dependent methyltransferases of the seven beta-strand family; role in vesicular transport	-4.02766	-2.00994	down
1770147_at	DBP8	YHR169W	ATPase, putative RNA helicase of the DEAD-box family; component of 90S preribosome complex involved in production of 18S rRNA and assembly of 40S small ribosomal subunit; ATPase activity stimulated by association with Esf2p	-4.0359	-2.01289	down
1773591_at	CLN1	YMR199W	G1 cyclin involved in regulation of the cell cycle; activates Cdc28p kinase to promote the G1 to S phase transition; late G1 specific expression depends on transcription factor complexes, MBF (Swi6p-Mbp1p) and SBF (Swi6p-Swi4p)	-4.04006	-2.01438	down
1773154_at	RCL1	YOL010W	Endonuclease that cleaves pre-rRNA at site A2 for 18S rRNA biogenesis; subunit of U3-containing 90S preribosome processome complex involved in small ribosomal subunit assembly; stimulates Bms1p GTPase and U3 binding activity; similar to RNA cyclase-like proteins but no cyclase activity detected	-4.04522	-2.01622	down
1770154_at	CAC2	YML102W	Subunit of chromatin assembly factor I (CAF-1), with Rlf2p and Msi1p; chromatin assembly by CAF-1 is important for multiple processes including silencing at telomeres, mating type loci, and rDNA; maintenance of kinetochore structure; deactivation of the DNA damage checkpoint after DNA repair; and chromatin dynamics during transcription	-4.0513	-2.01838	down
1774976_at	NOC4	YPR144C	Nucleolar protein, forms a complex with Nop14p that mediates maturation and nuclear export of 40S ribosomal subunits	-4.0581	-2.0208	down
1779816_at	RPL33B	YOR234C	Ribosomal protein L37 of the large (60S) ribosomal subunit, nearly identical to Rpl33Ap and has similarity to rat L35a; rpl33b null mutant exhibits normal growth while rpl33a rpl33b double null mutant is inviable	-4.07734	-2.02763	down
1772208_at	RPS11B	YBR048W	Protein component of the small (40S) ribosomal subunit; identical to Rps11Ap and has similarity to E. coli S17 and rat S11 ribosomal proteins	-4.08333	-2.02975	down

1776927_at	RPL9A	YGL147C	Protein component of the large (60S) ribosomal subunit, nearly identical to Rpl9Bp and has similarity to E. coli L6 and rat L9 ribosomal proteins	-4.10515	-2.03743	down
1770374_at	PMT3	YOR321W	Protein O-mannosyltransferase, transfers mannose residues from dolichyl phosphate-D-mannose to protein serine/threonine residues; acts in a complex with Pmt5p, can instead interact with Pmt1p in some conditions; target for new antifungals	-4.10657	-2.03793	down
1775178_at	KSS1	YGR040W	Mitogen-activated protein kinase (MAPK) involved in signal transduction pathways that control filamentous growth and pheromone response; the KSS1 gene is nonfunctional in S288C strains and functional in W303 strains	-4.11531	-2.041	down
1773989_at	GWT1	YJL091C	Protein involved in the inositol acylation of glucosaminyl phosphatidylinositol (GlcN-PI) to form glucosaminyl(acyl)phosphatidylinositol (GlcN(acyl)PI), an intermediate in the biosynthesis of glycosylphosphatidylinositol (GPI) anchors	-4.1178	-2.04187	down
1771463_at	RPA135	YPR010C	RNA polymerase I second largest subunit A135	-4.12452	-2.04423	down
1773719_at	NOC2	YOR206W	Protein that forms a nucleolar complex with Mak21p that binds to 90S and 66S pre-ribosomes, as well as a nuclear complex with Noc3p that binds to 66S pre-ribosomes; both complexes mediate intranuclear transport of ribosomal precursors	-4.12571	-2.04464	down
1775732_at	PUS7	YOR243C	Pseudouridine synthase, catalyzes pseudouridylation at positions 35 and 56 in U2 snRNA, position 50 in 5S rRNA, position 13 in cytoplasmic tRNAs, and position 35 in pre-tRNA(Tyr); conserved in archaea, vertebrates, and some bacteria	-4.13329	-2.04729	down
1774779_at	YPT31	YER031C	Rab family GTPase, very similar to Ypt32p; involved in the exocytic pathway; mediates intra-Golgi traffic or the budding of post-Golgi vesicles from the trans-Golgi	-4.13647	-2.0484	down
1772742_at	RPS0B	YLR048W	Protein component of the small (40S) ribosomal subunit, nearly identical to Rps0Ap; required for maturation of 18S rRNA along with Rps0Ap; deletion of either RPS0 gene reduces growth rate, deletion of both genes is lethal	-4.13756	-2.04878	down
1777922_at	TOS6	YNL300W	Glycosylphosphatidylinositol-dependent cell wall protein, expression is periodic and decreases in respone to ergosterol perturbation or upon entry into stationary phase; depletion increases resistance to lactic acid	-4.14562	-2.05159	down
1772676_at	GAR1	YHR089C	Protein component of the H/ACA snoRNP pseudouridylase complex, involved in the modification and cleavage of the 18S pre-rRNA	-4.16133	-2.05705	down
1776735_at	RPL34 A	YER056C-A	Protein component of the large (60S) ribosomal subunit, nearly identical to Rpl34Bp and has similarity to rat L34 ribosomal protein	-4.16513	-2.05836	down
1775866_at	BFR2	YDR299W	Essential protein that is a component of 90S preribosomes; may be involved in rRNA processing; multicopy suppressor of sensitivity to Brefeldin A; expression is induced during lag phase and also by cold shock	-4.16865	-2.05958	down
1777847_at		YKR045C		-4.17025	-2.06013	down
1776014_at	RRP1	YDR087C	Essential evolutionarily conserved nucleolar protein necessary for biogenesis of 60S ribosomal subunits and processing of pre-rRNAs to mature rRNAs, associated with several distinct 66S pre-ribosomal particles	-4.17568	-2.06201	down
1779350_at	SPC25	YER018C	Component of the evolutionarily conserved kinetochore-associated Ndc80 complex (Ndc80p-Nuf2p-Spc24p-Spc25p); involved in chromosome	-4.17924	-2.06324	down

			segregation, spindle checkpoint activity and kinetochore clustering			
1772456_at	IRC24	YIR036C	Putative benzil reductase;(GFP)-fusion protein localizes to the cytoplasm and is induced by the DNA-damaging agent MMS; sequence similarity with short-chain dehydrogenase/reductases; null mutant has increased spontaneous Rad52p foci	-4.18713	-2.06596	down
1777982_at	RPS7A	YOR096W	Protein component of the small (40S) ribosomal subunit, nearly identical to Rps7Bp; interacts with Kti11p; deletion causes hypersensitivity to zymocin; has similarity to rat S7 and Xenopus S8 ribosomal proteins	-4.18889	-2.06657	down
1770501_at	RPS8B	YER102W	Protein component of the small (40S) ribosomal subunit; identical to Rps8Ap and has similarity to rat S8 ribosomal protein	-4.19466	-2.06856	down
1775351_at	GCS1	YDL226C	ADP-ribosylation factor GTPase activating protein (ARF GAP), involved in ER-Golgi transport; shares functional similarity with Glo3p	-4.19973	-2.0703	down
1775052_at	UTP8	YGR128C	Nucleolar protein required for export of tRNAs from the nucleus; also copurifies with the small subunit (SSU) processome containing the U3 snoRNA that is involved in processing of pre-18S rRNA	-4.20021	-2.07046	down
1774347_at	IKI3	YLR384C	Subunit of Elongator complex, which is required for modification of wobble nucleosides in tRNA; maintains structural integrity of Elongator; homolog of human IKAP, mutations in which cause familial dysautonomia (FD)	-4.20422	-2.07184	down
1770139_at	TCB3	YML072C	Lipid-binding ER protein, enriched at ER-plasma membrane contact sites; localized to the bud via specific mRNA transport; non-tagged protein detected in a phosphorylated state in mitochondria; C-termini of Tcb1p, Tcb2p and Tcb3p interact	-4.2188	-2.07683	down
1773123_at	ALG7	YBR243C	UDP-N-acetyl-glucosamine-1-P transferase, transfers Glc-Nac-P from UDP-GlcNac to Dol-P in the ER in the first step of the dolichol pathway of protein asparagine-linked glycosylation; inhibited by tunicamycin	-4.22575	-2.07921	down
1769858_at	RPL15 A	YLR029C	Protein component of the large (60S) ribosomal subunit, nearly identical to Rpl15Bp and has similarity to rat L15 ribosomal protein; binds to 5.8 S rRNA	-4.24714	-2.08649	down
1775762_at	NOP56	YLR197W	Essential evolutionarily-conserved nucleolar protein component of the box C/D snoRNP complexes that direct 2'-O-methylation of pre-rRNA during its maturation; overexpression causes spindle orientation defects	-4.25541	-2.0893	down
1775512_at		YCR051W		-4.26588	-2.09284	down
1775966_at		YNL010W		-4.29077	-2.10124	down
1770871_at	CKS1	YBR135W	Cyclin-dependent protein kinase regulatory subunit and adaptor; modulates proteolysis of M-phase targets through interactions with the proteasome; role in transcriptional regulation, recruiting proteasomal subunits to target gene promoters	-4.29565	-2.10288	down
1777104_at	RPL6A	YML073C	N-terminally acetylated protein component of the large (60S) ribosomal subunit, has similarity to Rpl6Bp and to rat L6 ribosomal protein; binds to 5.8S rRNA	-4.30151	-2.10484	down
1776705_at	IMD3	YLR432W	Inosine monophosphate dehydrogenase, catalyzes the first step of GMP biosynthesis, member of a four-gene family in S. cerevisiae, constitutively expressed	-4.3074	-2.10682	down
1775737_at		YER156C		-4.3244	-2.1125	down
1771863_at	GNA1	YFL017C	Evolutionarily conserved glucosamine-6-phosphate acetyltransferase required for multiple cell cycle	-4.3295	-2.1142	down

			events including passage through START, DNA synthesis, and mitosis; involved in UDP-N-acetylglucosamine synthesis, forms GlcNAc6P from AcCoA			
1770349_at	SWI5	YDR146C	Transcription factor that recruits the Mediator and Swi/Snf complexes; activates transcription of genes expressed at the M/G1 phase boundary and in G1 phase; required for expression of the HO gene controlling mating type switching; localization to the nucleus occurs during G1 and appears to be regulated by phosphorylation by Cdc28p kinase	-4.33181	-2.11497	down
1776015_at	KAP123	YER110C	Karyopherin beta, mediates nuclear import of ribosomal proteins prior to assembly into ribosomes and import of histones H3 and H4; localizes to the nuclear pore, nucleus, and cytoplasm; exhibits genetic interactions with RAI1	-4.3366	-2.11656	down
1769954_at	GCN1	YGL195W	Positive regulator of the Gcn2p kinase activity, forms a complex with Gcn20p; proposed to stimulate Gcn2p activation by an uncharged tRNA	-4.35233	-2.12179	down
1779509_at	HUA2	YOR284W	Cytoplasmic protein of unknown function; computational analysis of large-scale protein-protein interaction data suggests a possible role in actin patch assembly	-4.36744	-2.12679	down
1779979_at	ZRT1	YGL255W	High-affinity zinc transporter of the plasma membrane, responsible for the majority of zinc uptake; transcription is induced under low-zinc conditions by the Zap1p transcription factor	-4.38424	-2.13233	down
1772892_at	NCL1	YBL024W	S-adenosyl-L-methionine-dependent tRNA: m5C-methyltransferase, methylates cytosine to m5C at several positions in tRNAs and intron-containing pre-tRNAs; similar to Nop2p and human proliferation associated nucleolar protein p120	-4.40163	-2.13804	down
1772948_at	POL5	YEL055C	DNA Polymerase phi; has sequence similarity to the human MybBP1A and weak sequence similarity to B-type DNA polymerases, not required for chromosomal DNA replication; required for the synthesis of rRNA	-4.46113	-2.15741	down
1774525_at	RPL36B	YPL249C-A	Protein component of the large (60S) ribosomal subunit, nearly identical to Rpl36Ap and has similarity to rat L36 ribosomal protein; binds to 5.8 S rRNA	-4.46556	-2.15884	down
1776009_at	PRT1	YOR361C	eIF3b subunit of the core complex of translation initiation factor 3 (eIF3), essential for translation; part of a subcomplex (Prt1p-Rpg1p-Nip1p) that stimulates binding of mRNA and tRNA(i)Met to ribosomes	-4.47733	-2.16264	down
1778368_at	YHP1	YDR451C	One of two homeobox transcriptional repressors (see also Yox1p), that bind to Mcm1p and to early cell cycle box (ECB) elements of cell cycle regulated genes, thereby restricting ECB-mediated transcription to the M/G1 interval	-4.4819	-2.16411	down
1774239_at		YGR079W		-4.50615	-2.1719	down
1770749_at	DSE1	YER124C	Daughter cell-specific protein, may regulate cross-talk between the mating and filamentation pathways; deletion affects cell separation after division and sensitivity to alpha-factor and drugs affecting the cell wall	-4.51825	-2.17576	down
1769685_at	EUG1	YDR518W	Protein disulfide isomerase of the endoplasmic reticulum lumen, function overlaps with that of Pdi1p; may interact with nascent polypeptides in the ER	-4.51995	-2.17631	down
1772876_at	STT3	YGL022W	Subunit of the oligosaccharyltransferase complex of the ER lumen, which catalyzes asparagine-linked glycosylation of newly synthesized proteins; forms a subcomplex with Ost3p and Ost4p and is directly involved in catalysis	-4.52938	-2.17932	down

1771626_at	RPF2	YKR081C	Essential protein involved in the processing of pre-rRNA and the assembly of the 60S ribosomal subunit; interacts with ribosomal protein L11; localizes predominantly to the nucleolus; constituent of 66S pre-ribosomal particles	-4.53225	-2.18023	down
1779360_at	FOL1	YNL256W	Multifunctional enzyme of the folic acid biosynthesis pathway, has dihydropteroate synthetase, dihydro-6-hydroxymethylpterin pyrophosphokinase, and dihydroneopterin aldolase activities	-4.54292	-2.18362	down
1770824_at	RPA190	YOR341W	RNA polymerase I largest subunit A190	-4.54525	-2.18436	down
1779632_at	SEC27	YGL137W	Essential beta'-coat protein of the COPI coatomer, involved in ER-to-Golgi and Golgi-to-ER transport; contains WD40 domains that mediate cargo selective interactions; 45% sequence identity to mammalian beta'-COP	-4.546	-2.1846	down
1778811_at	TRM11	YOL124C	Catalytic subunit of an adoMet-dependent tRNA methyltransferase complex (Trm11p-Trm112p), required for the methylation of the guanosine nucleotide at position 10 (m2G10) in tRNAs; contains a THUMP domain and a methyltransferase domain	-4.56395	-2.19028	down
1776391_at	MSB1	YOR188W	Protein involved in positive regulation of both 1,3-beta-glucan synthesis and the Pkc1p-MAPK pathway, potential Cdc28p substrate; multicopy suppressor of temperature-sensitive mutations in CDC24 and CDC42, and of mutations in BEM4	-4.58276	-2.19622	down
1776025_at	RPS9B	YBR189W	Protein component of the small (40S) ribosomal subunit; nearly identical to Rps9Ap and has similarity to E. coli S4 and rat S9 ribosomal proteins	-4.62194	-2.2085	down
1770200_at	YTM1	YOR272W	Constituent of 66S pre-ribosomal particles, forms a complex with Nop7p and Erb1p that is required for maturation of the large ribosomal subunit; has seven C-terminal WD repeats	-4.6303	-2.21111	down
1776507_at	RAX1	YOR301W	Protein involved in bud site selection during bipolar budding; localization requires Rax2p; has similarity to members of the insulin-related peptide superfamily	-4.63722	-2.21326	down
1774860_at	DPM1	YPR183W	Dolichol phosphate mannose (Dol-P-Man) synthase of the ER membrane, catalyzes the formation of Dol-P-Man from Dol-P and GDP-Man; required for glycosyl phosphatidylinositol membrane anchoring, O mannosylation, and protein glycosylation	-4.66127	-2.22072	down
1776118_at	UTP21	YLR409C	Subunit of U3-containing 90S preribosome and Small Subunit (SSU) processome complexes involved in production of 18S rRNA and assembly of small ribosomal subunit; synthetic defect with STI1 Hsp90 cochaperone; human homolog linked to glaucoma	-4.68066	-2.22671	down
1771302_at	ENV9	YOR246C	Protein proposed to be involved in vacuolar functions; mutant shows defect in CPY processing and defects in vacuolar morphology; has similarity to oxidoreductases, found in lipid particles; required for replication of Brome mosaic virus in S. cerevisiae, a model system for studying replication of positive-strand RNA viruses in their natural hosts	-4.68789	-2.22894	down
1773397_at	UTP5	YDR398W	Subunit of U3-containing Small Subunit (SSU) processome complex involved in production of 18S rRNA and assembly of small ribosomal subunit	-4.69486	-2.23108	down
1773827_s_at	RPL8A	YHL033C	Ribosomal protein L4 of the large (60S) Ribosomal 60S subunit protein L8A; required for processing of 27SA3 pre-rRNA to 27SB pre-rRNA during assembly of large ribosomal subunit; depletion leads to a turnover of pre-rRNA; L8 binds to	-4.71255	-2.23651	down

			Domain I of 25S and 5.8 S rRNAs; mutation results in decreased amounts of free 60S subunits; homologous to mammalian ribosomal protein L7A			
1770624_at	PAC10	YGR078C	Part of the heteromeric co-chaperone GimC/prefoldin complex, which promotes efficient protein folding	-4.72273	-2.23962	down
1773307_at	URA6	YKL024C	Uridylate kinase, catalyzes the seventh enzymatic step in the de novo biosynthesis of pyrimidines, converting uridine monophosphate (UMP) into uridine-5'-diphosphate (UDP)	-4.75008	-2.24795	down
1772857_at	RPS8A	YBL072C	Protein component of the small (40S) ribosomal subunit; identical to Rps8Bp and has similarity to rat S8 ribosomal protein	-4.76611	-2.25281	down
1772366_at	RPS1B	YML063W	Ribosomal protein 10 (rp10) of the small (40S) subunit; nearly identical to Rps1Ap and has similarity to rat S3a ribosomal protein	-4.78399	-2.25821	down
1769597_at	ERB1	YMR049C	Constituent of 66S pre-ribosomal particles, forms a complex with Nop7p and Ytm1p that is required for maturation of the large ribosomal subunit; required for maturation of the 25S and 5.8S ribosomal RNAs; homologous to mammalian Bop1	-4.80548	-2.26468	down
1771396_at	BRX1	YOL077C	Nucleolar protein, constituent of 66S pre-ribosomal particles; depletion leads to defects in rRNA processing and a block in the assembly of large ribosomal subunits; possesses a sigma(70)-like RNA-binding motif	-4.83974	-2.27493	down
1775536_at	RPL22 A	YLR061W	Protein component of the large (60S) ribosomal subunit, has similarity to Rpl22Bp and to rat L22 ribosomal protein	-4.84707	-2.27711	down
1778092_at	ELP2	YGR200C	Subunit of Elongator complex, which is required for modification of wobble nucleosides in tRNA; target of Kluyveromyces lactis zymocin	-4.89041	-2.28996	down
1771513_at	PTC2	YER089C	Type 2C protein phosphatase (PP2C); dephosphorylates Hog1p to limit maximal osmostress induced kinase activity; dephosphorylates Ire1p to downregulate the unfolded protein response; dephosphorylates Cdc28p; inactivates the DNA damage checkpoint	-4.89754	-2.29206	down
1774802_at	RPS11A	YDR025W	Protein component of the small (40S) ribosomal subunit; identical to Rps11Bp and has similarity to E. coli S17 and rat S11 ribosomal proteins	-4.92286	-2.2995	down
1779615_at	PHO3	YBR092C	Constitutively expressed acid phosphatase similar to Pho5p; brought to the cell surface by transport vesicles; hydrolyzes thiamin phosphates in the periplasmic space, increasing cellular thiamin uptake; expression is repressed by thiamin	-4.99738	-2.32117	down
1775109_at	ALF1	YNL148C	Alpha-tubulin folding protein, similar to mammalian cofactor B; Alf1p-GFP localizes to cytoplasmic microtubules; required for the folding of alpha-tubulin and may play an additional role in microtubule maintenance	-5.01159	-2.32527	down
1779952_at	SEC16	YPL085W	COPII vesicle coat protein required for ER transport vesicle budding; Sec16p is bound to the periphery of ER membranes and may act to stabilize initial COPII complexes; interacts with Sec23p, Sec24p and Sec31p	-5.0135	-2.32582	down
1776763_at	CLN2	YPL256C	G1 cyclin involved in regulation of the cell cycle; activates Cdc28p kinase to promote the G1 to S phase transition; late G1 specific expression depends on transcription factor complexes, MBF (Swi6p-Mbp1p) and SBF (Swi6p-Swi4p)	-5.03437	-2.33181	down
1774335_at	NOP58	YOR310C	Protein involved in pre-rRNA processing, 18S rRNA synthesis, and snoRNA synthesis; component of the small subunit processome complex, which is required for processing of pre-18S rRNA	-5.04841	-2.33583	down

1769973_at	CLB1	YGR108W	B-type cyclin involved in cell cycle progression; activates Cdc28p to promote the transition from G2 to M phase; accumulates during G2 and M, then targeted via a destruction box motif for ubiquitin-mediated degradation by the proteasome	-5.08582	-2.34648	down
1770205_at	YVH1	YIR026C	Protein phosphatase involved in vegetative growth at low temperatures, sporulation, and glycogen accumulation; mutants are defective in 60S ribosome assembly; member of the dual-specificity family of protein phosphatases	-5.09206	-2.34825	down
1773459_at	ARX1	YDR101C	Shuttling pre-60S factor; involved in the biogenesis of ribosomal large subunit biogenesis; interacts directly with Alb1; responsible for Tif6 recycling defects in absence of Rei1; associated with the ribosomal export complex	-5.09421	-2.34886	down
1775645_at	REX2	YLR059C	3'-5' RNA exonuclease; involved in 3'-end processing of U4 and U5 snRNAs, 5S and 5.8S rRNAs, and RNase P and RNase MRP RNA; localized to mitochondria and null suppresses escape of mtDNA to nucleus in yme1 yme2 mutants; RNase D exonuclease	-5.11396	-2.35444	down
1780155_at	PAN6	YIL145C	Pantothenate synthase, also known as pantoate-beta-alanine ligase, required for pantothenic acid biosynthesis, deletion causes pantothenic acid auxotrophy, homologous to E. coli panC	-5.12365	-2.35717	down
1769503_at	RPS17B	YDR447C	Ribosomal protein 51 (rp51) of the small (40s) subunit; nearly identical to Rps17Ap and has similarity to rat S17 ribosomal protein	-5.13186	-2.35948	down
1778317_at	ADH4	YGL256W	Alcohol dehydrogenase isoenzyme type IV, dimeric enzyme demonstrated to be zinc-dependent despite sequence similarity to iron-activated alcohol dehydrogenases; transcription is induced in response to zinc deficiency	-5.1324	-2.35963	down
1772514_at	SCP160	YJL080C	Essential RNA-binding G protein effector of mating response pathway, mainly associated with nuclear envelope and ER, interacts in mRNA-dependent manner with translating ribosomes via multiple KH domains, similar to vertebrate vigilins	-5.13596	-2.36063	down
1779287_at	NOG2	YNR053C	Putative GTPase that associates with pre-60S ribosomal subunits in the nucleolus and is required for their nuclear export and maturation	-5.13752	-2.36107	down
1776859_at	NOP1	YDL014W	Nucleolar protein, component of the small subunit processome complex, which is required for processing of pre-18S rRNA; has similarity to mammalian fibrillarin	-5.20674	-2.38038	down
1774084_at	INO2	YDR123C	Component of the heteromeric Ino2p/Ino4p basic helix-loop-helix transcription activator that binds inositol/choline-responsive elements (ICREs), required for derepression of phospholipid biosynthetic genes in response to inositol depletion	-5.21602	-2.38295	down
1778592_at	RPS1A	YLR441C	Ribosomal protein 10 (rp10) of the small (40S) subunit; nearly identical to Rps1Bp and has similarity to rat S3a ribosomal protein	-5.22189	-2.38457	down
1777241_at	RPS19A	YOL121C	Protein component of the small (40S) ribosomal subunit, required for assembly and maturation of pre-40 S particles; mutations in human RPS19 are associated with Diamond Blackfan anemia; nearly identical to Rps19Bp	-5.22409	-2.38518	down
1770812_at	ZAP1	YJL056C	Zinc-regulated transcription factor; binds to zinc-responsive promoters to induce transcription of certain genes in presence of zinc, represses other genes in low zinc; regulates its own transcription; contains seven zinc-finger domains	-5.28536	-2.402	down
1769539_at	SSU72	YNL222W	Transcription/RNA-processing factor that associates with TFIIB and cleavage/polyadenylation factor Pta1p; exhibits	-5.32158	-2.41186	down

			phosphatase activity on serine-5 of the RNA polymerase II C-terminal domain; affects start site selection in vivo			
1779514_at	ALB1	YJL122W	Shuttling pre-60S factor; involved in the biogenesis of ribosomal large subunit; interacts directly with Arx1p; responsible for Tif6p recycling defects in absence of Rei1p	-5.33199	-2.41467	down
1771957_at	IMO32	YGR031W	Conserved mitochondrial protein of unknown function; processed by both mitochondrial processing peptidase and mitochondrial octapeptidyl aminopeptidase; gene contains the nested antisense gene NAG1	-5.3611	-2.42253	down
1770597_at		YKL027W		-5.43988	-2.44357	down
1778912_at	NOP2	YNL061W	Probable RNA m(5)C methyltransferase, essential for processing and maturation of 27S pre-rRNA and large ribosomal subunit biogenesis; localized to the nucleolus; constituent of 66S pre-ribosomal particles	-5.44448	-2.44479	down
1772817_at	NSR1	YGR159C	Nucleolar protein that binds nuclear localization sequences, required for pre-rRNA processing and ribosome biogenesis	-5.45689	-2.44808	down
1771764_at	RAS1	YOR101W	GTPase involved in G-protein signaling in the adenylate cyclase activating pathway, plays a role in cell proliferation; localized to the plasma membrane; homolog of mammalian RAS proto-oncogenes	-5.50135	-2.45979	down
1772022_at	STM1	YLR150W	Protein required for optimal translation under nutrient stress; perturbs association of Yef3p with ribosomes; involved in TOR signaling; binds G4 quadruplex and purine motif triplex nucleic acid; helps maintain telomere structure	-5.50138	-2.45979	down
1775939_at	SIL1	YOL031C	Nucleotide exchange factor for the endoplasmic reticulum (ER) lumenal Hsp70 chaperone Kar2p, required for protein translocation into the ER; homolog of Yarrowia lipolytica SLS1; GrpE-like protein	-5.51326	-2.46291	down
1773510_at	RPL27B	YDR471W	Protein component of the large (60S) ribosomal subunit, nearly identical to Rpl27Ap and has similarity to rat L27 ribosomal protein	-5.51704	-2.46389	down
1770780_at	SRO9	YCL037C	Cytoplasmic RNA-binding protein that associates with translating ribosomes; involved in heme regulation of Hap1p as a component of the HMC complex, also involved in the organization of actin filaments; contains a La motif	-5.56074	-2.47528	down
1770452_at		YKL069W		-5.58814	-2.48237	down
1774694_at		YDL063C		-5.60885	-2.48771	down
1771819_at	PWP2	YCR057C	Conserved 90S pre-ribosomal component essential for proper endonucleolytic cleavage of the 35 S rRNA precursor at A0, A1, and A2 sites; contains eight WD-repeats; PWP2 deletion leads to defects in cell cycle and bud morphogenesis	-5.70945	-2.51335	down
1772374_at		YJR054W		-5.72105	-2.51628	down
1771304_at	UTP13	YLR222C	Nucleolar protein, component of the small subunit (SSU) processome containing the U3 snoRNA that is involved in processing of pre-18S rRNA	-5.72248	-2.51664	down
1777706_at	EMG1	YLR186W	Member of the alpha/beta knot fold methyltransferase superfamily; required for maturation of 18S rRNA and for 40S ribosome production; interacts with RNA and with S-adenosylmethionine; associates with spindle/microtubules; forms homodimers	-5.74525	-2.52237	down
1771736_at	PMT1	YDL095W	Protein O-mannosyltransferase, transfers mannose from dolichyl phosphate-D-mannose to protein Ser/Thr residues; 1 of 7 related proteins involved in	-5.78438	-2.53216	down

			O-glycosylation which is essential for cell wall rigidity; involved in ER quality control			
1771206_at	MCD4	YKL165C	Protein involved in glycosylphosphatidylinositol (GPI) anchor synthesis; multimembrane-spanning protein that localizes to the endoplasmic reticulum; highly conserved among eukaryotes	-5.83368	-2.54441	down
1773667_at	DOG2	YHR043C	2-deoxyglucose-6-phosphate phosphatase, member of a family of low molecular weight phosphatases, similar to Dog1p, induced by oxidative and osmotic stress, confers 2-deoxyglucose resistance when overexpressed	-5.86062	-2.55105	down
1772199_at	BIO3	YNR058W	7,8-diamino-pelargonic acid aminotransferase (DAPA), catalyzes the second step in the biotin biosynthesis pathway; BIO3 is in a cluster of 3 genes (BIO3, BIO4, and BIO5) that mediate biotin synthesis	-5.91593	-2.56461	down
1779532_at	RPL17B	YJL177W	Protein component of the large (60S) ribosomal subunit, nearly identical to Rpl17Ap and has similarity to E. coli L22 and rat L17 ribosomal proteins	-5.9393	-2.57029	down
1774223_at	HAS1	YMR290C	ATP-dependent RNA helicase; localizes to both the nuclear periphery and nucleolus; highly enriched in nuclear pore complex fractions; constituent of 66S pre-ribosomal particles	-6.05581	-2.59832	down
1769890_at	RRB1	YMR131C	Essential nuclear protein involved in early steps of ribosome biogenesis; physically interacts with the ribosomal protein Rpl3p	-6.08534	-2.60534	down
1773427_at	RPS22B	YLR367W	Protein component of the small (40S) ribosomal subunit; nearly identical to Rps22Ap and has similarity to E. coli S8 and rat S15a ribosomal proteins	-6.11228	-2.61171	down
1770925_at	FSH1	YHR049W	Putative serine hydrolase that localizes to both the nucleus and cytoplasm; sequence is similar to S. cerevisiae Fsh2p and Fsh3p and the human candidate tumor suppressor OVCA2	-6.16871	-2.62497	down
1776958_at		YNL058C		-6.26772	-2.64794	down
1771490_at	NSA2	YER126C	Protein constituent of 66S pre-ribosomal particles, contributes to processing of the 27S pre-rRNA	-6.30177	-2.65576	down
1769730_at	FUR1	YHR128W	Uracil phosphoribosyltransferase, synthesizes UMP from uracil; involved in the pyrimidine salvage pathway	-6.33424	-2.66317	down
1776516_at	RPL13 A	YDL082W	Protein component of the large (60S) ribosomal subunit, nearly identical to Rpl13Bp; not essential for viability; has similarity to rat L13 ribosomal protein	-6.37416	-2.67223	down
1775167_at	RPS26B	YER131W	Protein component of the small (40S) ribosomal subunit; nearly identical to Rps26Ap and has similarity to rat S26 ribosomal protein	-6.37839	-2.67319	down
1775150_at	NIP7	YPL211W	Nucleolar protein required for 60S ribosome subunit biogenesis, constituent of 66S pre-ribosomal particles; physically interacts with Nop8p and the exosome subunit Rrp43p	-6.4493	-2.68914	down
1777228_at		YMR230W-A		-6.60164	-2.72282	down
1779813_at	RPA49	YNL248C	RNA polymerase I subunit A49	-6.60681	-2.72395	down
1771781_at	DPH5	YLR172C	Methyltransferase required for synthesis of diphthamide, which is a modified histidine residue of translation elongation factor 2 (Eft1p or Eft2p); not essential for viability; GFP-Dph5p fusion protein localizes to the cytoplasm	-6.6276	-2.72849	down
1778296_at	CSI2	YOL007C	Protein of unknown function; green fluorescent protein (GFP)- fusion protein localizes to the mother side of the bud neck and the vacuole; YOL007C is not an essential gene	-6.64372	-2.73199	down
1769937_at	MIS1	YBR084W	Mitochondrial C1-tetrahydrofolate synthase, involved in interconversion between different	-6.66123	-2.73579	down

			oxidation states of tetrahydrofolate (THF); provides activities of formyl-THF synthetase, methenyl-THF cyclohydrolase, and methylene-THF dehydrogenase			
1779549_at	DFR1	YOR236W	Dihydrofolate reductase involved in tetrahydrofolate biosynthesis; required for respiratory metabolism	-6.7433	-2.75345	down
1779336_at	FRS2	YFL022C	Alpha subunit of cytoplasmic phenylalanyl-tRNA synthetase, forms a tetramer with Frs1p to form active enzyme; evolutionarily distant from mitochondrial phenylalanyl-tRNA synthetase based on protein sequence, but substrate binding is similar	-6.80011	-2.76556	down
1774174_at	RPL6B	YLR448W	Protein component of the large (60S) ribosomal subunit, has similarity to Rpl6Ap and to rat L6 ribosomal protein; binds to 5.8S rRNA	-6.80348	-2.76627	down
1770353_at	GIT1	YCR098C	Plasma membrane permease, mediates uptake of glycerophosphoinositol and glycerophosphocholine as sources of the nutrients inositol and phosphate; expression and transport rate are regulated by phosphate and inositol availability	-7.16964	-2.8419	down
1779136_at	RSA4	YCR072C	WD-repeat protein involved in ribosome biogenesis; may interact with ribosomes; required for maturation and efficient intra-nuclear transport or pre-60S ribosomal subunits, localizes to the nucleolus	-7.17847	-2.84368	down
1779097_at	RKI1	YOR095C	Ribose-5-phosphate ketol-isomerase, catalyzes the interconversion of ribose 5-phosphate and ribulose 5-phosphate in the pentose phosphate pathway; participates in pyridoxine biosynthesis	-7.18669	-2.84533	down
1772412_at	PMT2	YAL023C	Protein O-mannosyltransferase, transfers mannose residues from dolichyl phosphate-D-mannose to protein Ser/Thr residues; involved in ER quality control; acts in a complex with Pmt1p, can instead interact with Pmt5; target for new antifungal	-7.20482	-2.84896	down
1775626_at	URA7	YBL039C	Major CTP synthase isozyme (see also URA8), catalyzes the ATP-dependent transfer of the amide nitrogen from glutamine to UTP, forming CTP, the final step in de novo biosynthesis of pyrimidines; involved in phospholipid biosynthesis	-7.3307	-2.87395	down
1770298_at	RPS9A	YPL081W	Protein component of the small (40S) ribosomal subunit; nearly identical to Rps9Bp and has similarity to E. coli S4 and rat S9 ribosomal proteins	-7.36357	-2.8804	down
1770653_at	RPS0A	YGR214W	Protein component of the small (40S) ribosomal subunit, nearly identical to Rps0Bp; required for maturation of 18S rRNA along with Rps0Bp; deletion of either RPS0 gene reduces growth rate, deletion of both genes is lethal	-7.43701	-2.89472	down
1778063_at	NOG1	YPL093W	Putative GTPase that associates with free 60S ribosomal subunits in the nucleolus and is required for 60S ribosomal subunit biogenesis; constituent of 66S pre-ribosomal particles; member of the ODN family of nucleolar G-proteins	-7.62757	-2.93122	down
1776856_at	VPS75	YNL246W	NAP family histone chaperone; binds to histones and Rtt109p, stimulating histone acetyltransferase activity; possesses nucleosome assembly activity in vitro; proposed role in vacuolar protein sorting and in double-strand break repair	-7.67886	-2.94089	down
1775352_at	PRP43	YGL120C	RNA helicase in the DEAH-box family, functions in both RNA polymerase I and polymerase II transcript metabolism, involved in release of the lariat-intron from the spliceosome	-7.68376	-2.94181	down
1775342_at	LIA1	YJR070C	Deoxyhypusine hydroxylase, a HEAT-repeat containing metalloenzyme that catalyzes hypusine formation; binds to and is required for the modification of Hyp2p (eIF5A); complements S.	-7.8415	-2.97113	down

			pombe mmd1 mutants defective in mitochondrial positioning			
1777152_at	BIO5	YNR056C	Putative transmembrane protein involved in the biotin biosynthesis pathway; responsible for uptake of 7-keto 8-aminopelargonic acid; BIO5 is in a cluster of 3 genes (BIO3, BIO4, and BIO5) that mediate biotin synthesis	-7.9105	-2.98377	down
1779946_at	AAH1	YNL141W	Adenine deaminase (adenine aminohydrolase), converts adenine to hypoxanthine; involved in purine salvage; transcriptionally regulated by nutrient levels and growth phase; Aah1p degraded upon entry into quiescence via SCF and the proteasome	-8.03582	-3.00645	down
1771341_at	ZPS1	YOL154W	Putative GPI-anchored protein; transcription is induced under low-zinc conditions, as mediated by the Zap1p transcription factor, and at alkaline pH	-8.10144	-3.01818	down
1777399_at	ARD1	YHR013C	Subunit of N-terminal acetyltransferase NatA (Nat1p, Ard1p, Nat5p); acetylates many proteins and thus affects telomeric silencing, cell cycle, heat-shock resistance, mating, and sporulation; human Ard1p levels are elevated in cancer cells	-8.21894	-3.03895	down
1778319_at	PCL1	YNL289W	Cyclin, interacts with cyclin-dependent kinase Pho85p; member of the Pcl1,2-like subfamily, involved in the regulation of polarized growth and morphogenesis and progression through the cell cycle; localizes to sites of polarized cell growth	-8.6333	-3.10991	down
1774259_at		YOR387C		-9.18904	-3.19991	down
1776309_at	ULI1	YFR026C	Putative protein of unknown function involved in and induced by the endoplasmic reticulum unfolded protein response	-9.26689	-3.21208	down
1772723_at	GCD10	YNL062C	Subunit of tRNA (1-methyladenosine) methyltransferase with Gcd14p, required for the modification of the adenine at position 58 in tRNAs, especially tRNAi-Met; first identified as a negative regulator of GCN4 expression	-9.31988	-3.22031	down
1774987_at	SCS3	YGL126W	Protein required for inositol prototrophy, identified as an ortholog of the FIT family of proteins involved in triglyceride droplet biosynthesis; disputed role in the synthesis of inositol phospholipids from inositol	-9.56511	-3.25778	down
1773996_at	VEL1	YGL258W	Protein of unknown function; highly induced in zinc-depleted conditions and has increased expression in NAP1 deletion mutants	-9.76725	-3.28795	down
1772916_at	BIO4	YNR057C	Dethiobiotin synthetase, catalyzes the third step in the biotin biosynthesis pathway; BIO4 is in a cluster of 3 genes (BIO3, BIO4, and BIO5) that mediate biotin synthesis; expression appears to be repressed at low iron levels	-9.96664	-3.31711	down
1771055_at	RPL18B	YNL301C	Ribosomal 60S subunit protein L18B; homologous to mammalian ribosomal protein L18	-10.5065	-3.39321	down
1779645_at	DBP2	YNL112W	Essential ATP-dependent RNA helicase of the DEAD-box protein family, involved in nonsense-mediated mRNA decay and rRNA processing	-10.8572	-3.44058	down
1775443_at		YHR033W		-11.5945	-3.53537	down

Table S4: Complete list of significantly (p<0.05) over-represented MIPS (The Munich Information Center for Protein Sequences) functional categories in the dataset of VA induced transcriptome (≥2 fold) obtained by FunSpec bioinformatics tool.

Category	p-value	In Category from Cluster	k	f
Sulfate assimilation [01.02.03.01]	3.85E-07	MET8 MET10 MET3 MET5 MET14 MET1 MET22 MET16	8	8
Peroxisomal transport [20.09.03]	3.88E-07	PEX19 PEX7 PEX5 PEX3 FAA2 PEX14 PEX21 PEX18 PEX2 PXA2 CAT2 PEX17 PEX15	13	19
Development of asco- basidio- or zygospore [43.01.03.09]	8.60E-07	YSW1 DTR1 SPS22 ADY3 UBC5 ARG82 SPR28 DON1 SPT3 DIT2 DIT1 SPS2 SPS1 MEI4 SHC1 SPO74 ATG1 IME4 SPR3 AMA1 RIM4 SPS100 SSP1 PFS1 IME2 IME1 SPO75 SNF7 OSW2 CDA1 CDA2 BDF1 SMA2 SPO20 MCM1 TEP1 YNL194C SLZ1 CNM67 EMI5 SPO21 UFE1 MPC54 SPR1 PTP2 SSP2 OSW1 MUM3 SMA1 HSP82 REC8	51	166
Meiosis [10.03.02]	8.47E-06	PCH2 SDS24 SPS22 ADY2 CDC7 YDL114W IWR1 SPR28 DON1 ZIP1 SPS2 SPS1 MAM1 DMC1 MSH4 RIM15 CNN1 RIM8 RCK1 RAD54 IME4 ZIP2 RIM4 SPO13 SSP1 HOS4 IME2 IME1 SET3 SLZ1 MER1 SPO21 MPC54 GAC1 SLK19 PTP2 MUM3 MEK1 RAD17 SRL4 HSP82 REC8 YPR078C KAR3	44	146
Sugar transport [20.01.03.01]	2.17E-05	MAL31 SNF3 HXT15 MPH2 MTH1 HXT13 HXT10 MAL11 HXT8 HXT9 GAL2 HXT14 HXT17 HXT11 SKS1	15	31
Siderophore-iron transport [20.01.01.01.01.01]	0.000104	VPS41 SIT1 AFT1 ARN1 ARN2 FET3 FRE4 ENB1	8	12
Drug/toxin transport [20.01.27]	0.00014	DTR1 SIT1 TPO2 SNG1 AZR1 ARN1 ARN2 PDR11 TPO1 ATR1 YMR279C ENB1 PDR5 PDR10 YOR378W PDR12	16	39
Transcriptional control [11.02.03.04]	0.0011	RTG3 REG2 SPT7 SMP1 LRE1 HMLALPHA1 MED2 MBP1 UGA3 ASF2 GIS1 TAF12 CTH1 ARG82 PLP1 UME6 UPC2 MET32 YAP6 HDA2 TFB1 SPT3 STP1 FLO8 YER130C BUR6 EPL1 CAF16 SWP82 YFL052W MIG1 AFT1 TOS8 SUT1 CUP2 IME4 MGA1 MAL13 STP2 ORC6 RTT107 CST6 XBP1 HOS4 YAP5 SIP4 GSM1 GZF3 IME1 PUT3 PHD1 MSN4 YKL070W STB6 CTK1 YKL222C DAL80 BAS1 SIR1 GAT3 BDF1 YAP1 TAF11 CTK3 SUB1 ARG80 MCM1 RNA14 GAT2 SPT21 CRZ1 PHO23 YAF9 EAF7 SKO1 SNF12 RTG1 SPT20 CIN5 GAC1 ESA1 TAF3 HST2 SWI1 SKS1 MET31 MUK1 CCL1 CSR2 NUT2 HDA3	91	426
Autoproteolytic processing [14.07.11.01]	0.001147	ATG8 ATG14 ATG12 ATG20 ATG1 SNX4 ATG10 ATG23 ATG16 ATG3 ATG13	11	26
Regulation of glycolysis and gluconeogenesis [02.01.03]	0.001232	VID24 RMD5 UBC8 FYV10 PFK26 SIP4 FBP26 GID8 PFK27	9	19
Cellular import [20.09.18]	0.002621	BAP2 SUL1 MAL31 SNF3 UGA4 BAP3 SIT1 HXT13 HXT10 MUP1 MUP3 DAL4 HXT8 HXT9 YMR1 DAL5 FRE2 GAL2 FRE1 FET3 MEP2 ALP1 HXT14 HXT17 HXT11	25	90
Allantoin and allantoate transport [20.01.23]	0.003622	YIL166C DAL4 DAL5 YCT1 THI73	5	8
Regulation of nitrogen metabolism [01.02.07.01]	0.003622	GLN3 GAT1 GZF3 DAL80 NPR1	5	8
Proton driven symporter [20.03.02.02.01]	0.003954	MAL31 UGA4 MAL11	3	3

Chemical agent resistance [32.05.01.03]	0.004238	PDR3 MAG1 SNG1 YAP1 MLF3 PDR16 ROD1 CIN5 PDR5	9	22
Regulation of nitrogen, sulfur and selenium metabolism [01.02.07]	0.004238	MET8 UGA3 UME6 MET32 DCG1 GAT3 ARG80 MCM1 MET31	9	22
Lysosomal and vacuolar protein degradation [14.13.04]	0.004864	ATG8 VID24 ATG12 ATG1 NVJ1 ATG16 ATG3	7	15
Detoxification [32.07]	0.005254	PDR3 DTR1 TRX3 SIT1 PUG1 ROG3 TPO2 SNG1 AZR1 ARN1 ARN2 NFT1 YKR104W TPO1 ATR1 MCM1 YMR279C ENB1 ROD1 CRS5 PDR5 YOR378W	22	80
Regulation of C-compound and carbohydrate metabolism [01.05.25]	0.006608	RTG3 REG2 ADY2 SNF3 NRG1 CDC34 MTH1 GIP2 YFL052W MIG1 PCL10 HAP2 MAL13 PFK26 RPI1 FBP26 SFC1 MSN4 HAP4 SNF7 PIG1 MCM1 GLC8 ATO2 RTG1 PFK27 TFC7 GAC1 SWI1 SKS1 PCL8	31	126
Metabolism of arginine [01.01.03.05]	0.007156	VBA2 VBA3 ARG82 ARG80	4	6
Amino acid/amino acid derivatives transport [20.01.07]	0.007647	BAP2 VBA2 VBA3 UGA4 BAP3 AGP3 MUP1 MUP3 MMP1 STP3 ALP1 CRC1 DIP5 AGC1	14	45
ABC transporters [20.03.25]	0.008094	PDR15 PDR11 PXA2 NFT1 YKR104W ATR1 ENB1 PDR5 PDR10 PDR12	10	28
Meiosis I [10.03.02.01]	0.009757	UME6 MEI4 AMA1 REC104 REC102 REC114	6	13
Meiotic recombination [10.01.05.03.01]	0.011554	MMS4 MUS81 MEI4 DMC1 MSH4 MND1 RIM4 REC104 REC102 REC114 RAD17 MLH3	12	38
C-compound and carbohydrate transport [20.01.03]	0.012667	ADY2 MCH1 STL1 YAT2 YFL040W DUR3 MCH2 CAT2 MCH4 PDR12 GUP2	11	34
Detoxification involving cytochrome P450 [32.07.01]	0.013945	DIT2 JLP1 ERG5	3	4
Proton driven antiporter [20.03.02.03.01]	0.014616	VBA2 VBA3 KHA1 ATO2	4	7
Ion transport [20.01.01]	0.014616	FIT1 YCT1 FIT2 FIT3	4	7
Peroxisome [42.19]	0.015901	PEX19 PEX7 PEX5 PEX10 PEX3 PEX21 PEX18 PEX2 PEX17 PEX15 ATG11	11	35
General transcription activities [11.02.03.01]	0.018827	MMS4 HMLALPHA1 MED2 UGA3 UPC2 MET32 YAP6 SPT3 STP1 FLO8 BUR6 MIG1 SUT1 BRF1 STP2 POG1 MET28 YAP5 SIP4 GZF3 PUT3 PHD1 BAS1 ARG80 MCM1 RRN9 BDP1 SKO1 SPT20 CIN5 TFC7 MET31 NUT2	33	146
Stress response [32.01]	0.018863	NTG1 PAU7 SSA3 YRO2 HSP30 PAU3 RTK1 PPZ2 ECM10 CYC7 PAU2 TIR1 DDI1 PAU5 RIM15 DAK2 MGA1 TIR3 XBP1 SDP1 YJL144W YJL160C HAL5 MSN4 HSP104 PAU4 MCM1 SNO1 SNZ1 CRZ1 TIR4 TIR2 PTP2 HSP82 ATH1 KAR3	36	162
Mating (fertilization) [41.01.01]	0.018997	FIG1 SPT7 HMLALPHA1 FIG2 PRM7 SPT3 PRM8 RAD54 SPR3 KEL2 PRM2 MID2 PRM6 KAR5 KAR1 AGA1 PRM4 KAR3	18	69
METABOLISM [01]	0.025027	DIT2 DIT1	2	2
Regulation of amino acid metabolism [01.01.13]	0.027156	CDC34 ARG82 UME6 MET32 MET28 PUT3 BAS1 ARG80 MCM1 MET31	10	33
Vacuolar/lysosomal transport [20.09.13]	0.035408	ATG8 VID24 ATG12 BSD2 VBA3 ATG20 GYP7 UBC5 VPS64 VPS72 VPS3 DDI1 ATG1 HSE1 VPS51 DID2 ATG10 SNF7 SRN2 IMH1 ATG23 VPS71 MVP1 VPS20 YPT53 VPS27 ATG3 PEP12 VPS21 VAM3 APM1 ATG11 ATG13	33	153
DNA repair [10.01.05.01]	0.037065	NTG1 MMS4 POL4 RAD59 NSE4 RAD28 GIS1 NSE3 TFB1 RAD34 MUS81 MAG1 RAD4 PES4 ECO1 RAD54 MND1 THI4 RNR3 RAD26 RAD7 RAD5 SMC6 PIF1 PSO2 RAD14 YKU70 FYV6 NTG2 EXO1	34	159

		RAD17 RAD53 MLH3 CCL1		
Metabolism of methionine [01.01.06.05]	0.037155	MET32 MET3 MET1 MHT1 ADI1 MET31 MET16	7	21
Modification by acetylation, deacetylation [14.07.04]	0.037544	SPT7 RXT3 TAF12 HDA2 SPT3 EPL1 SGF73 SWC4 HOS4 SET3 PHO23 EAF7 SPT20 ESA1 HST2 HDA3 HPA2	17	69
Metabolism of cysteine [01.01.09.03]	0.040424	MET32 STR2 MHT1 MET31	4	9
Oxidation of fatty acids [02.25]	0.040424	POX1 POT1 TES1 ECI1	4	9
DNA processing [10.01]	0.040424	SDS24 ADY2 SRL4 YPR078C	4	9
DNA degradation [01.03.16.03]	0.041868	SDS24 ADY2 CST6 SRL4 YPR078C	5	13

‘k’ indicates the number of genes from input cluster in given category whereas ‘f’ indicates the number of genes total in given category.

Table S5: Complete list of significantly ($p<0.05$) over-represented MIPS (The Munich Information Center for Protein Sequences) functional categories in the dataset of VA repressed transcriptome (≥2 fold) obtained by FunSpec bioinformatics tool.

Category	p-value	In Category from Cluster	k	f
Ribosomal proteins [12.01.01]	1.00E-14	RPL19B RPS8A RPL23A RPL32 RPS11B RPL19A RPS6B RPS9B RPL21A NOP1 RPL31A RPL13A RPS16B RPL35A RPL4B RPS11A MAK21 RPS13 RLI1 RPL12B RPS17B RPS18A RPL27B RPL37B RPL12A RPL34A RPS24A RPS8B RPL23B RPS26B SPB4 RPL2A RPL29 RPL24A RPL7A DBP3 RPL28 RPL9A RPS26A RPS25A RPL26B YGR054W RPL11B RPS23A NSR1 RPS0A RPL14B MRP4 RPS20 RPL8A RPL27A SSF1 RPL42B NMD3 RPS4B RPL2B RPL34B RPS24B RPL16A RPS21B RPL17B RPL39 RPS22A RPS14B RPL43B RSM7 RPS5 RPL14A RPL17A RPS21A RPF2 RPL40B RPL8B RPL15A RPS0B RPL22A RPL37A RPS28B RPL38 RPP0 RPL26A RPS22B RPS29A RPL31B RPS1A RPL6B RPS17A RPS18B RPS1B RPL6A MRPL3 RPL13B RPS16A RPL36A RPS10B RPL20A RPL16B RPS7B RPS3 RPL18B RPS19B DBP6 RPS15 BRX1 RPL18A RPS19A RPL25 RPS7A RPS28A RPL33B RPS10A RPL20B RPL21B RPS9A RPL5 NIP7 RPL36B RPL43A RPL11A RPS23B	120	246
RNA binding [16.03.03]	1.00E-14	PTA1 UTP20 ENP1 GBP2 SRO9 PWP2 LHP1 NHP2 TMA64 UTP4 UTP5 NPL3 UTP6 TMA20 LCP5 PAB1 RPL24A RPL28 RPL26B UTP22 UTP8 PTI1 NSR1 PXR1 RPL14B MRP4 SSF1 GAR1 IMP3 NMD3 RPL16A MRS1 NOP9 UTP18 SCP160 UTP10 RPS14B TMA22 RPL14A RPF2 RPL15A DIP2 EMG1 UTP13 RPL26A UTP21 RPL6B RPL6A UTP14 UTP15 RPL36A RRP5 RLP7 RPL16B RNH201 IMP4 NAF1 NOP13 BRX1 TRM11 RPL25 UAF30 NOP4 NAN1 RPL5 RPL36B MRD1 NOC4	68	189
rRNA processing [11.04.01]	1.14E-14	UTP20 POP8 ENP1 KRR1 PWP2 RSA4 NOP1 TSR1 RRP42 NHP2 RRP1 UTP4 UTP5 NPL3 UTP6 SNU13 NUG1 LCP5 SPB4 CGR1 DBP3 SRM1 ROK1 KEM1 UTP22 NOP7 UTP8 MTR3 NSR1 SKI6 PXR1 RPP1 RRP3 GAR1 IMP3 DBP8 RIX1 NOP9 UTP18 UTP10 MPP10 MRT4 URB1 RPF2 NOC3 REX2 DIP2 CBF5 EMG1 NOP56 UTP13 UTP21 UTP14 ERB1 UTP15 RRP5 HAS1 RLP7 NOP2 IMP4 DBP2 RIO2 SSU72 DBP6 ESF2 RCL1 DIS3 RRP6 UTP23 RRS1 NOP58 RRP12 NOP4 NAN1 NIP7 MRD1 NOC4	77	169
Ribosome biogenesis [12.01]	2.37E-13	MAK16 KRR1 RSA4 TSR1 ARX1 NUG1 NSA2 CGR1 SRM1 NOP7 SDA1 IMP3 RIX1 MRT4 TOR2 RIX7 RLP24 EMG1 ASC1 RRB1 RLP7 NOP15 NCS2 RIA1 NOG2 ESF2 NOC2 YTM1 RRS1 RRP12 NOG1 TIF5 NOC4	33	64
tRNA modification [11.06.02]	2.04E-08	NCL1 TRM7 MIS1 SLM3 TRM8 TRM1 TRM82 PUS2 TRM5 TAD2 TRM2 DUS3 DUS4 TRM9 GCD10 TRM112 RCL1 TRM10 TRM11 PUS7 MOD5	21	43
Nuclear transport [20.09.01]	2.08E-05	NUP170 KAP104 RSA4 NPL3 NUG1 NUP157 KAP123 NUP145 SRM1 SEH1 NUP192 NMD5 NUP120 NUP133 SRP40 RIX7 KAP95 NDC1 NUP188 POM152 NUP53 PSE1 RIO2 ACC1 RPS15 NOC2	26	84

rRNA modification [11.06.01]	2.76E-05	NOP1 NHP2 LCP5 GAR1 IMP3 MPP10 CBF5 NOP56 IMP4 NOP58	10	18
O-directed glycosylation, deglycosylation [14.07.02.01]	3.09E-05	PMT2 KTR3 PMT5 PMT1 MNT2 PMT6 PMT4 PMT3 DPM1	9	15
Mitotic cell cycle and cell cycle control [10.03.01]	4.27E-05	MAK16 PKC1 RPG1 CKS1 YBR238C ALG7 RSC6 PCL2 GCS1 NUM1 SUP35 SPC25 BEM2 GNA1 MOB2 CDC14 SDS23 KEM1 PAC10 ARD1 KIC1 SPC97 SIM1 STH1 FKH1 SCP160 CDC11 KDX1 LAS1 TRX1 DIP2 CBF5 YLR179C TUB4 STT4 NDC1 VAN1 CDC5 CIN4 CLN1 VPS75 PCL1 EGT2 DIS3 MDM20 RFC1 YTM1 PRT1 NAN1 DIB1	50	215
rRNA synthesis [11.02.01]	5.03E-05	POL5 CKB1 RPC10 RPA34 RPA12 RPC25 RRB1 RPC19 RPC31 RPA49 RPC34 RPB8 RRS1 UAF30 RPA43 RPA190 RPA135 RPC40 RPO26	19	55
Protein transport [20.01.10]	6.74E-05	NUP170 TIM12 YSY6 SEC66 SBH2 NUP157 KAP123 OXA1 DNF1 SEC53 NUP145 SEH1 TOM20 SAM35 TOM71 TIM44 NMD5 NUP120 LHS1 NUP133 PAM18 SEC72 STT4 KAP95 UBX2 NUP188 POM152 NUP53 PSE1 TIM23 RPS15 SEC63 MRS6 TIM50 SEC62 TOM5	36	141
Translational control [12.07]	0.000175	RPS11B RPS9B PAT1 RPS11A GCN2 PAB1 ECM32 GCN1 MTO1 RPS23A SLH1 SKI2 TRM9 THO2 CAF20 RPS9A TIF5 RPS23B	18	55
Metabolism of secondary products derived from L-lysine, L-arginine and L-histidine [01.20.31]	0.000226	KTI11 DPH1 JJJ3 DPH2 DPH5	5	6
G1 phase of mitotic cell cycle [10.03.01.01.01]	0.000245	CCR4 KTI11 TAF5 SWI5 SDA1 TOR2 ACE2 TAF13 TAF9 TAF14	10	22
Aminoacyl-tRNA-synthetases [12.10]	0.00031	GRS1 SES1 FRS2 GUS1 VAS1 MES1 DED81 THS1 DPS1 FRS1 YNL247W WRS1 ISM1 CDC60	14	39
ER to Golgi transport [20.09.07.03]	0.000597	ERP2 ERV46 ERP1 COP1 SEC31 GCS1 SEC7 SEC26 BFR2 RET2 SEC27 EMP24 ERV29 SFB3 SEC24 GEA1 ERV25 TRS130 YIP3 SEC21	20	70
Nuclear membrane [42.10.05]	0.00111	NUP170 NUP157 NUP145 SEH1 NUP192 NUP120 NUP133 NUP188 POM152 NUP53 ACC1	11	30
BIOGENESIS OF CELLULAR COMPONENTS [42]	0.00142	MAK21 CGR1 RRB1 NOC2	4	5
Translation initiation [12.04.01]	0.001476	RPG1 RLI1 YGR054W TIF4631 CLU1 TIF34 TIF11 NIP1 GCD10 GCD1 PRT1 TIF5 TIF3	13	40
RNA transport [20.01.21]	0.001652	NUP170 KAP104 HMT1 GBP2 SUB2 NPL3 NUP157 PAB1 NUP145 SRM1 SEH1 MTR3 SCP160 NUP120 NUP133 NDC1 NUP188 POM152 NUP53 PSE1 TPM1 THO2	22	86
Cell growth / morphogenesis [40.01]	0.00324	CLN3 KIN3 SRO77 MNN2 HSL7 ENP1 FEN1 ABP1 PPH22 DOP1 BFR2 SPR6 DSE1 CKB1 SUA5 NCS6 STE20 HTD2 TRM5 KIC1 TAO3 FAR1 HOC1 CDC11 TOR2 CBF5 BUD8 CRN1 BUB2 EFR3 TPM1 NCS2 EGT2 SPE2 MSB1 HUA2 MRS6 CLN2 NHP6A	39	189
Aminosaccharide anabolism [01.05.09.04]	0.003809	PCM1 GNA1 PGM1 GNT1	4	6
Aminosaccharide catabolism [01.05.09.07]	0.003809	PCM1 GNA1 PGM1 GNT1	4	6
Purin nucleotide/nucleoside/nucleobase metabolism [01.03.01]	0.004165	MIS1 SHM1 DAS2 ADK1 PPX1 ADO1 MEU1 IMD4 GUA1 DFR1	10	30
Posttranslational modification of amino acids (e.g. hydroxylation, methylation) [14.07.09]	0.004882	KTI11 HMT1 HSL7 BPL1 RMT2 DPH1 JJJ3 DPH2 DPH5	9	26

Pyrimidine nucleotide/nucleoside/nucleobase metabolism [01.03.04]	0.005427	FUI1 GDA1 GNA1 PUS2 DCD1 URA8 URA6 URA1 CBF5 URA5	10	31
Pyrimidine nucleotide/nucleoside/nucleobase anabolism [01.03.04.03]	0.006491	URA7 PRS3 FUR1 URA4 URK1 CDC21	6	14
Fatty acid derivatives mediated signal transduction [30.01.09.09]	0.008584	PKC1 CDS1 ISC1	3	4
tRNA synthesis [11.02.02]	0.008922	LHP1 CKB1 RPC10 RPC25 RPC19 RPC31 RPC34 RPB8 NHP6A RPC40 RPO26	11	38
Non-vesicular ER transport [20.09.05]	0.009613	SEC66 SBH2 SEC72 SIL1 SEC63 SEC62	6	15
Protein targeting, sorting and translocation [14.04]	0.012544	VPS8 ERP2 ERP1 PEP1 NUP170 KAP104 TIM12 YSY6 SEC66 TMA64 CYM1 NPL3 SEC20 SBH2 NUP157 KAP123 SEC53 NUP145 SEH1 CHC1 TOM20 VPS62 SAM35 TIM44 NMD5 NUP120 LHS1 NUP133 VPS13 PAM18 YLR243W SEC72 STT4 KAP95 UBX2 NDC1 NUP188 POM152 NUP53 SCJ1 VPS75 ACC1 TIM23 MIM1 SIL1 RPS15 SEC63 EGD1 TIM50 SEC62 TOM5	51	281
RNA modification [11.06]	0.017865	NOP2 TGS1	2	2
Microtubule cytoskeleton [42.04.05]	0.018136	ATS1 NUM1 SPC25 BEM2 SPC97 SIM1 MHP1 TUB4 CRN1 NDC1 CIN4 RHO2	12	47
PROTEIN SYNTHESIS [12]	0.020639	TRM7 SRO9 NAT1 PPH22 SSB1 SSZ1 SSB2	7	22
Mitotic cell cycle [10.03.01.01]	0.021554	REI1 SPO12 YOX1 NIS1 CLN2	5	13
N-directed glycosylation, deglycosylation [14.07.02.02]	0.022857	ALG3 KTR3 ALG7 ANP1 STT3 HOC1 MNS1 VAN1 SCJ1 GNT1 DPM1	11	43
Translation termination [12.04.03]	0.02294	SUP35 BTT1 EGD2 EGD1	4	9
Amino acid metabolism [01.01]	0.028879	MIS1 GCN2 RMT2 YHR033W TRM5 DPH2 OXP1 DPH5 YML096W DFR1	10	39
Vitamine/cofactor transport [20.01.25]	0.029922	PHO3 YCF1 FLX1 THI7 BIO5	5	14
Transcription termination [11.02.03.01.07]	0.029922	PTA1 GRS1 PTI1 ESS1 SSU72	5	14
Structural protein binding [16.07]	0.030499	NUP170 SEC31 NUP157 NUP145 SEH1 CHC1 NUP120 NUP133 NUP188 POM152 NUP53 DML1 SEC16	13	56
Utilization of vitamins, cofactors, and prosthetic groups [01.07.04]	0.03485	CYC3 PHO3 BPL1	3	6
tRNA processing [11.04.02]	0.036558	PTA1 POP8 SOL2 LHP1 NUP145 SRM1 RPP1 TRZ1 GCD10 RCL1 SEN54	11	46
RNA degradation [01.03.16.01]	0.038374	CCR4 RRP42 SUP35 KEM1 MTR3 SKI6 NUC1 MRT4 SKI2 PUB1 DBP2 DIS3	12	52
mRNA processing (splicing, 5'-, 3'-end processing) [11.04.03]	0.038936	PAT1 NPL3 PAB1 SRM1 KEM1 MTR3 ESS1 MRT4	8	30
DNA binding [16.03.01]	0.044865	GBP2 RPO21 SLD5 POL5 NSR1 RPC10 HOP1 ZAP1 RPA34 RPA12 RPC25 STM1 YOX1 DAT1 CEP3 RNH201 RPC19 RPC31 RPA49 RPC34 RPB11 HST3 RPB8 RPA43 RPA190 HHO1 RPA135 RPC40 RPO26	29	158
Peptide transport [20.01.09]	0.048281	DNF1 STE6 MDL1 OPT2	4	11
GTP binding [16.19.05]	0.048281	RBG1 RBG2 GPA1 CIN4	4	11

'k' indicates the number of genes from input cluster in given category whereas 'f' indicates the number of genes total in given category.

Table S6: Ranking of Transcriptional Factors (TF's) that were induced and repressed (≥2 fold) in VA transcriptome using YEASTRACT tool.

Regulation of Entire VA transcriptome by Induced TF's				S.No.	Regulation of Entire VA transcriptome by Repressed TF's			
Transcription Factor	% in user set	% in Yeastract	p-value		Transcription Factor	% in user set	% in Yeastract	p-value
BAS1	53.28%	37.42%	0	1	ACE2	82.02%	33.24%	0
CST6	45.68%	32.80%	0	2	SWI5	42.33%	42.13%	0
MSN4	44.50%	33.57%	0	3	YOX1	28.17%	38.17%	0
YAP1	42.84%	28.02%	0.106245706	4	ZAP1	26.51%	33.53%	$3.53609E^{-10}$
CIN5	38.09%	33.81%	0	5	YHP1	25.43%	38.23%	0
SPT20	32.61%	32.58%	$4.33162E^{-10}$	6	HFI1	24.29%	32.55%	$2.45163E^{-07}$
RPN4	31.94%	34.66%	0	7	FKH1	18.40%	44.11%	0
MCM1	28.68%	33.23%	$2.44901E^{-10}$	8	TAF14	14.47%	30.80%	0.004805878
AFT1	26.56%	37.11%	0	9	SRB2	13.23%	31.68%	0.001310951
HAP2	24.81%	29.74%	0.005088448	10	CAC2	13.07%	37.65%	$2.42588E^{-10}$
GLN3	20.05%	32.88%	$1.17832E^{-06}$	11	CDC73	12.61%	30.93%	0.00666202
SPT3	19.38%	30.49%	0.002445978	12	YLR278C	7.39%	33.65%	0.001135734
YAP6	18.09%	35.90%	$7.0422E^{-11}$	13	ISW1	7.34%	33.18%	0.002208848
SKO1	17.00%	35.57%	$1.0354E^{-09}$	14	INO2	7.24%	32.48%	0.005636704
PDR3	16.95%	27.87%	0.289022929	15	HIR3	4.86%	23.21%	0.967433963
YAP5	15.40%	34.10%	$7.93891E^{-07}$	16	MET18	4.03%	32.23%	0.034316049
UME6	15.30%	33.94%	$1.36248E^{-06}$	17	DAT1	3.77%	31.06%	0.080871631
ROX1	15.19%	32.67%	$5.13527E^{-05}$	18	HOG1	3.41%	31.28%	0.080470026
PHD1	14.63%	37.09%	$1.23544E^{-10}$	19	WTM2	3.26%	37.50%	0.001274797
DAL82	14.06%	33.50%	$1.25862E^{-05}$	20	KSS1	2.95%	30.65%	0.128572995
HAP4	13.64%	29.97%	0.025005554	21	TOS4	2.79%	26.73%	0.530711148
XBP1	12.56%	33.94%	$1.33438E^{-05}$	22	HST3	2.17%	27.81%	0.396912776
NRG1	12.30%	33.06%	0.00012058	23	THO2	1.40%	36.99%	0.02491288
RTG3	12.20%	35.17%	8.89109E-07	24	STB3	0.72%	41.18%	0.025268705
MGA1	12.09%	33.29%	8.37552E-05	25	MDS3	0.41%	20.00%	0.801660634
PUT3	11.63%	34.19%	1.59034E-05	26	IMP2'	0.05%	20.00%	0.414652757
RFX1	11.63%	32.01%	0.001414063	27				
MBP1	11.37%	31.84%	0.002139071	28				
FLO8	10.49%	32.85%	0.000550179	29				
STP1	10.49%	28.31%	0.236251701	30				
MET32	10.23%	36.07%	$9.9106E^{-07}$	31				
HAA1	9.92%	28.79%	0.163659197	32				
STP2	9.87%	29.70%	0.066397422	33				
CUP2	9.66%	32.35%	0.001990379	34				
RTT107	8.89%	33.86%	0.000275611	35				
GAT3	8.68%	37.67%	$2.78326E^{-07}$	36				
MET31	8.11%	38.57%	$1.16452E^{-07}$	37				
GIS1	7.70%	40.16%	$1.13944E^{-08}$	38				

CRZ1	7.39%	28.60%	0.224727354	39	
MIG1	7.34%	31.63%	0.014660325	40	
RCO1	6.46%	26.65%	0.595674864	41	
SUT1	6.41%	29.74%	0.10997118	42	
ARG80	6.10%	29.87%	0.104334723	43	
UGA3	5.58%	34.07%	0.002573186	44	
TOS8	5.58%	26.54%	0.60661358	45	
GZF3	5.48%	36.81%	0.000116242	46	
TIS11	5.37%	32.60%	0.012969578	47	
MTH1	5.37%	30.06%	0.104121223	48	
UPC2	4.86%	29.94%	0.124213234	49	
SMP1	4.65%	31.69%	0.038933342	50	
AZF1	4.34%	42.42%	$1.06441E^{-06}$	51	
MET28	4.29%	36.24%	0.000966397	52	
SIP4	4.29%	34.30%	0.005751923	53	
RTG1	4.03%	28.16%	0.336550435	54	
SIR1	4.03%	25.41%	0.749286906	55	
RPI1	3.51%	39.31%	0.000180453	56	
GAT1	3.51%	31.78%	0.058031345	57	
DAL80	3.10%	29.85%	0.178484545	58	
YER130C	2.74%	30.46%	0.147547759	59	
GSM1	2.64%	30.18%	0.170339498	60	
YPR015C	2.38%	33.33%	0.045255873	61	
ARG82	2.27%	30.99%	0.135289105	62	
PHO23	2.17%	33.33%	0.052015524	63	
USV1	2.17%	31.58%	0.110677075	64	
YFL052W	1.96%	44.71%	0.000166103	65	
IME1	1.81%	28.69%	0.318031014	66	
JHD1	1.60%	29.52%	0.259067983	67	
POG1	1.55%	31.91%	0.128340699	68	
STB6	1.24%	26.97%	0.469617874	69	
MAL13	1.09%	30.43%	0.229219827	70	
YKL222C	1.03%	33.33%	0.115097669	71	
HPA2	0.83%	27.12%	0.442365641	72	
ESA1	0.16%	100.00%	0	73	

REFERENCES:

1 Azad, G. K. *et al.* Ebselen induces reactive oxygen species (ROS)-mediated cytotoxicity in Saccha-romyces cerevisiae with inhibition of glutamate dehydrogenase being a target. *Febs Open Bio* **4**, 77-89, doi:10.1016/j.fob.2014.01.002 (2014).

2 Ruepp, A. *et al.* The FunCat, a functional annotation scheme for systematic classification of proteins from whole genomes. *Nucleic Acids Res* **32**, 5539-5545, doi:10.1093/nar/gkh894 (2004).

3 Robinson, M. D., Grigull, J., Mohammad, N. & Hughes, T. R. FunSpec: a web-based cluster interpreter for yeast. *BMC Bioinformatics* **3**, 35 (2002).

4 Maere, S., Heymans, K. & Kuiper, M. BiNGO: a Cytoscape plugin to assess overrepresentation of gene ontology categories in biological networks. *Bioinformatics* **21**, 3448-3449, doi:10.1093/bioinformatics/bti551 (2005).

5 Zheng, Q. & Wang, X. J. GOEAST: a web-based software toolkit for Gene Ontology enrichment analysis. *Nucleic Acids Res* **36**, W358-363, doi:10.1093/nar/gkn276 (2008).

6 Shannon, P. *et al.* Cytoscape: a software environment for integrated models of biomolecular interaction networks. *Genome Res* **13**, 2498-2504, doi:10.1101/gr.1239303 (2003).

7 Priebe, S., Kreisel, C., Horn, F., Guthke, R. & Linde, J. FungiFun2: a comprehensive online resource for systematic analysis of gene lists from fungal species. *Bioinformatics* **31**, 445-446, doi:10.1093/bioinformatics/btu627 (2015).

8 Teixeira, M. C. *et al.* The YEASTRACT database: an upgraded information system for the analysis of gene and genomic transcription regulation in Saccharomyces cerevisiae. *Nucleic Acids Res* **42**, D161-166, doi:10.1093/nar/gkt1015 (2014).

9 O'Connor, T. R. & Wyrick, J. J. ChromatinDB: a database of genome-wide histone modification patterns for Saccharomyces cerevisiae. *Bioinformatics* **23**, 1828-1830, doi:10.1093/bioinformatics/btm236 (2007).

10 Tsaponina, O., Barsoum, E., Astrom, S. U. & Chabes, A. Ixr1 is required for the expression of the ribonucleotide reductase Rnr1 and maintenance of dNTP pools. *PLoS Genet* **7**, e1002061, doi:10.1371/journal.pgen.1002061 (2011).

11 Capaldi, A. P. *et al.* Structure and function of a transcriptional network activated by the MAPK Hog1. *Nat Genet* **40**, 1300-1306, doi:10.1038/ng.235 (2008).

12 Suresh, H. G. *et al.* Prolonged starvation drives reversible sequestration of lipid biosynthetic enzymes and organelle reorganization in Saccharomyces cerevisiae. *Mol Biol Cell* **26**, 1601-1615, doi:10.1091/mbc.E14-11-1559 (2015).

13 Kurtz, S. & Shore, D. RAP1 protein activates and silences transcription of mating-type genes in yeast. *Genes Dev* **5**, 616-628 (1991).

14 Sandager, L. *et al.* Storage lipid synthesis is non-essential in yeast. *Journal of Biological Chemistry* **277**, 6478-6482, doi:10.1074/jbc.M109109200 (2002).
